# Supplementary figures and images for: Prion propagation is controlled by a hierarchical network involving the nuclear Tfap2c and hnRNP K factors and the cytosolic mTORC1 complex
Source: PLoS Pathog. 2026 Apr 20;22(4):e1014056. doi: 10.1371/journal.ppat.1014056 (PMC13108870; doi:10.1371/journal.ppat.1014056)

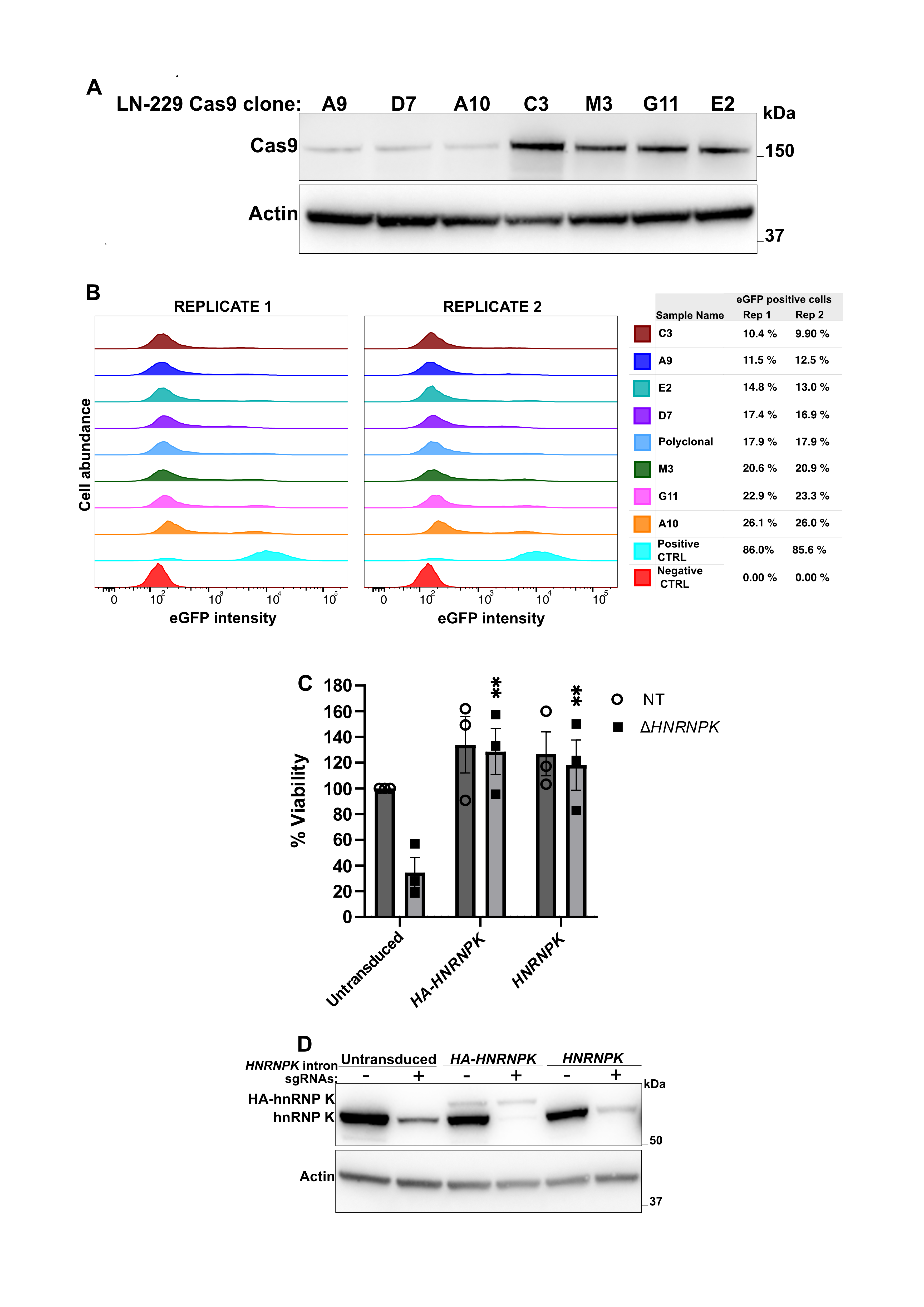

Supplement: S1 Fig — A. Cas9 protein in isolated LN-229 Cas9 clones. B. Flow cytometry-based determination of Cas9 activity in the LN-229 Cas9 clones by an eGFP reporter assay. Cas9 activity was estimated from the percentage of the eGFP-negative cells. LN-229 not expressing Cas9 or the eGFP reporter were used as positive and negative controls, respectively. C. Viability of LN-229 C3 cells upon ablation of the HNRNPK endogenous gene (CellTiter-Glo assay). LN-229 C3 cells expressed a vector carrying either the HA-HNRNPK or the HNRNPK coding sequence. Untransduced cells were used for control. Results are normalized on the untransduced non-targeting condition (NT). n = 3, each with 3 individually treated wells. D. The western blot refers to the data shown in C. -: NT, + : HNRNPK sgRNAs. Data information: n represents independent experiments. Mean ± SEM. **: p < 0.01 (Two-way ANOVA Dunnett’s test). (TIFF) [file ppat.1014056.s001.tiff]

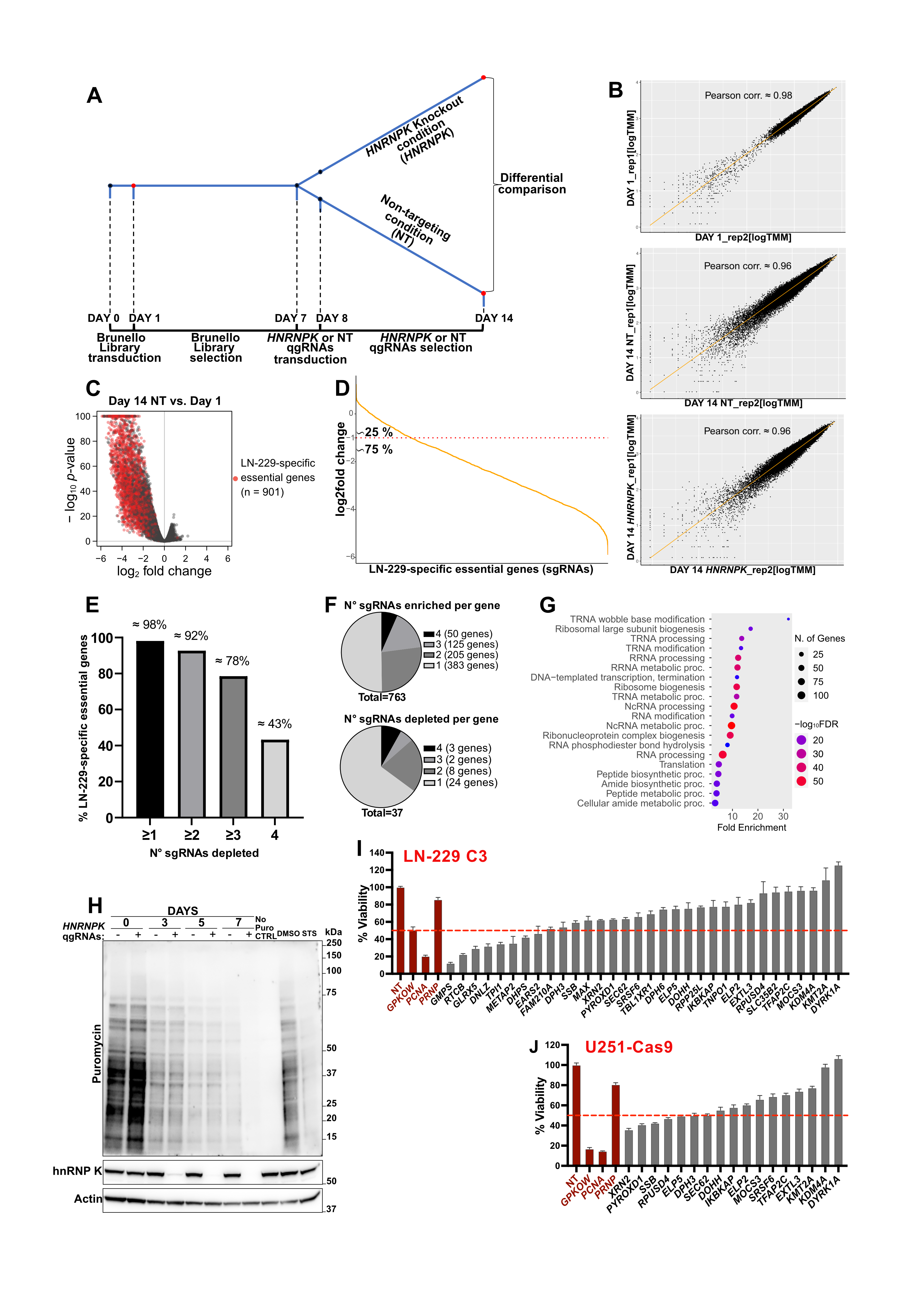

Supplement: S2 Fig — A. Workflow of the genome-wide CRISPR deletion screen. The red dots highlight the three time points subjected to next-generation sequencing (NGS) and analysis (Day 1, Day 14 NT, Day 14 HNRNPK). B. Correlation between the two experimental replicates of the screen for the three analyzed conditions: Day 1, Day 14 NT, and Day 14 HNRNPK. C. Volcano plot showing the differential sgRNAs abundance in Day 14 NT vs. Day 1. Red-filled circles indicate the sgRNAs targeting LN-229 essential genes. D. Distribution of sgRNAs targeting LN-229 essential genes in the Day 14 NT vs. Day 1 comparison. E. Percentage of LN-229 essential genes with at least one, two, three, or four sgRNAs depleted in the Day 14 NT vs. Day 1 comparison. F. Distribution of the number of sgRNAs per gene significantly enriched or depleted. G. Gene enrichment biological process analysis of the genes with ≥2 sgRNAs enriched in HNRNPK vs. NT at day 14. H. Puromycin labeling and detection of global protein synthesis in LN-229 C3 cells transduced with HNRNPK (+) or NT (-) qgRNAs. 4 hours, 1 μM staurosoprine (STS) was used for control. I, J. Cell viability upon individual deletion of each of the candidate genes obtained from the screen (CellTiter-Glo assay). Results are normalized against the seeded cell density and compared to the NT condition. Red columns indicate the control groups: non-targeting control (NT), non-specific genes (PCNA, GPKOW, PRNP). The red dashed line highlights the viability threshold set at 50% of the NT condition. Mean ± SEM, n ≥ 3 independent experiments. (TIFF) [file ppat.1014056.s002.tiff]

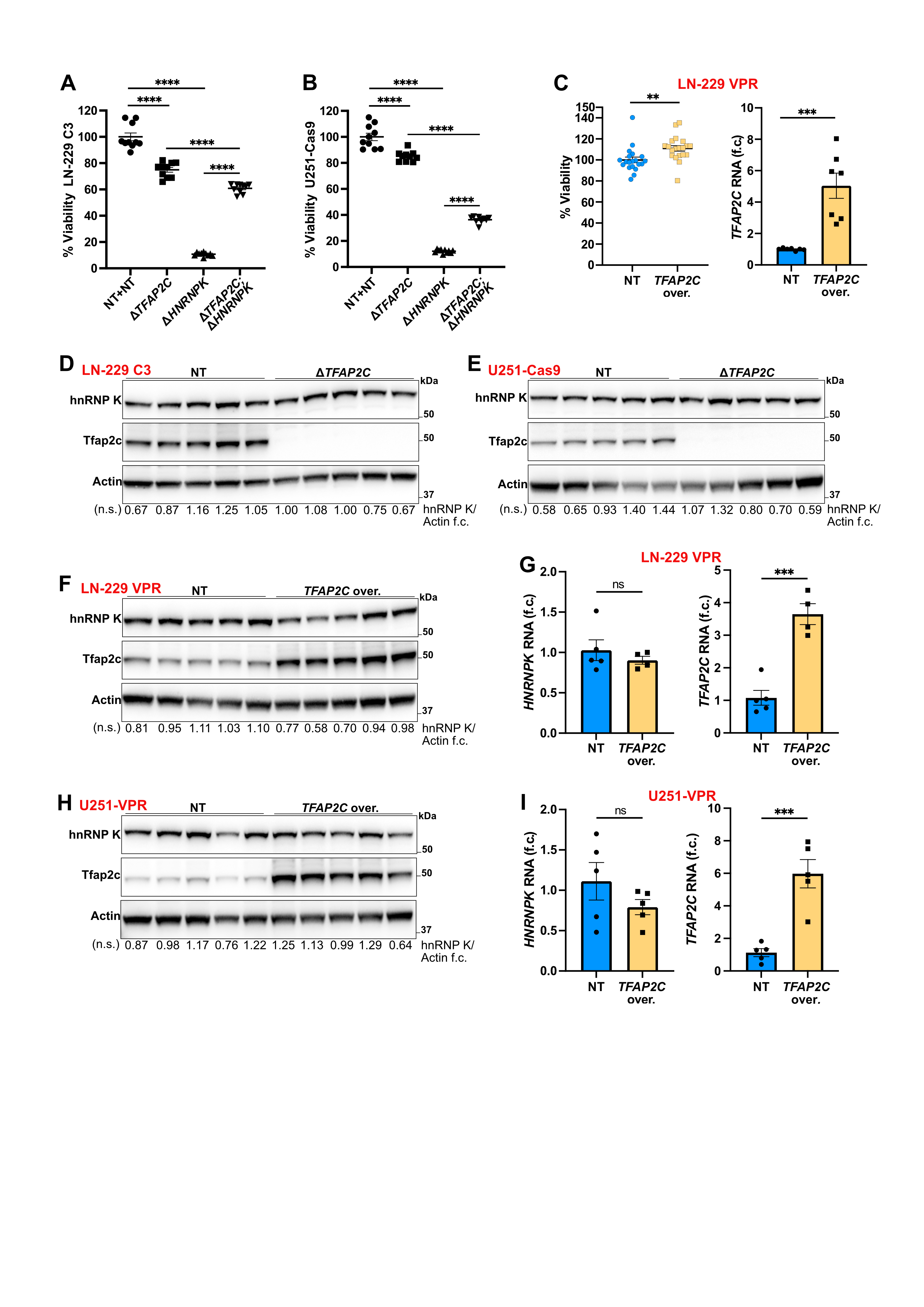

Supplement: S3 Fig — A-B. Viability of ΔTFAP2C or NT-unmodified cells 7 (A) and 10 days (B) upon delivering HNRNPK or NT qgRNAs (CellTiter-Glo assay). Results are normalized against the seeded cell density before HNRNPK ablation. 10 individually treated wells. C. Viability of LN-229 dCas9-VPR cells upon TFAP2C overexpression (CellTiter-Glo assay). 20 individually treated wells. qRT-PCR: n = 7. D-E. hnRNP K protein upon TFAP2C ablation. n = 5. F-I. hnRNP K protein (F, H) and RNA (G, I) after TFAP2C overexpression in dCas9-VPR cells. WB: n = 5. qRT-PCR: n ≥ 4. J-K. Confocal images showing hnRNP K and Tfap2c proteins in ΔTFAP2C and NT-unmodified cells. hnRNP K and Tfap2c proteins were also imaged in cells transduced with HNRNPK or NT qgRNAs for 4 (J) or 6 (K) days. L. Co-immunoprecipitation of Tfap2c and hnRNP K in ΔTFAP2C and WT LN-229 C3 cells. IP: Immunoprecipitated Protein; FT: Flow Through after immunoprecipitation. Data information: qRT-PCR results are normalized against GAPDH expression. n represents independent experiments. f.c.: fold change. Mean ± SEM. ns: p > 0.05, **: p < 0.01, ***: p < 0.001, ****: p < 0.0001 (Two-way ANOVA Uncorrected Fisher’s LSD in A-B. Unpaired t-test in C-I.). (TIFF) [file ppat.1014056.s003.tiff]

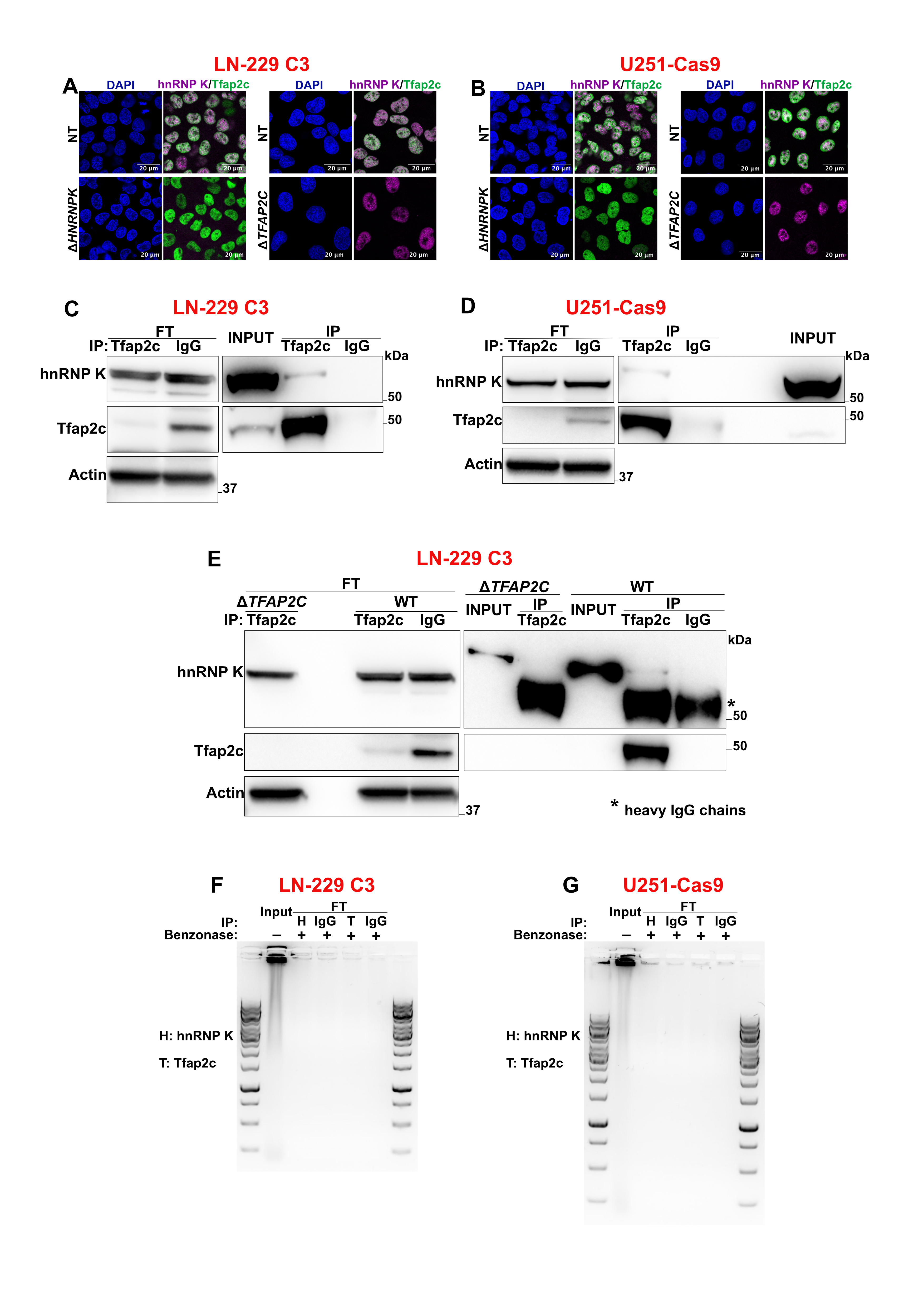

Supplement: S4 Fig — A-B. Confocal images showing hnRNP K and Tfap2c proteins in ΔTFAP2C and NT-unmodified cells. hnRNP K and Tfap2c proteins were also imaged in cells transduced with HNRNPK or NT qgRNAs for 4 (A) or 6 (B) days. C-E. Co-immunoprecipitation of Tfap2c and hnRNP K in no-detergent conditions in WT LN-229 C3 (C), WT U251-Cas9 (D), and ΔTFAP2C LN-229 C3 cells (E). IP: Immunoprecipitated Protein; FT: Flow-Through after immunoprecipitation. F-G. 1% agarose gel electrophoresis of DNA from the FT of co-immunoprecipitated samples shown in Fig 2H and 2I, incubated overnight with benzonase nuclease. The original input, not digested with benzonase nuclease and diluted to match the final protein concentration of the FT, was included as a control. (TIFF) [file ppat.1014056.s004.tiff]

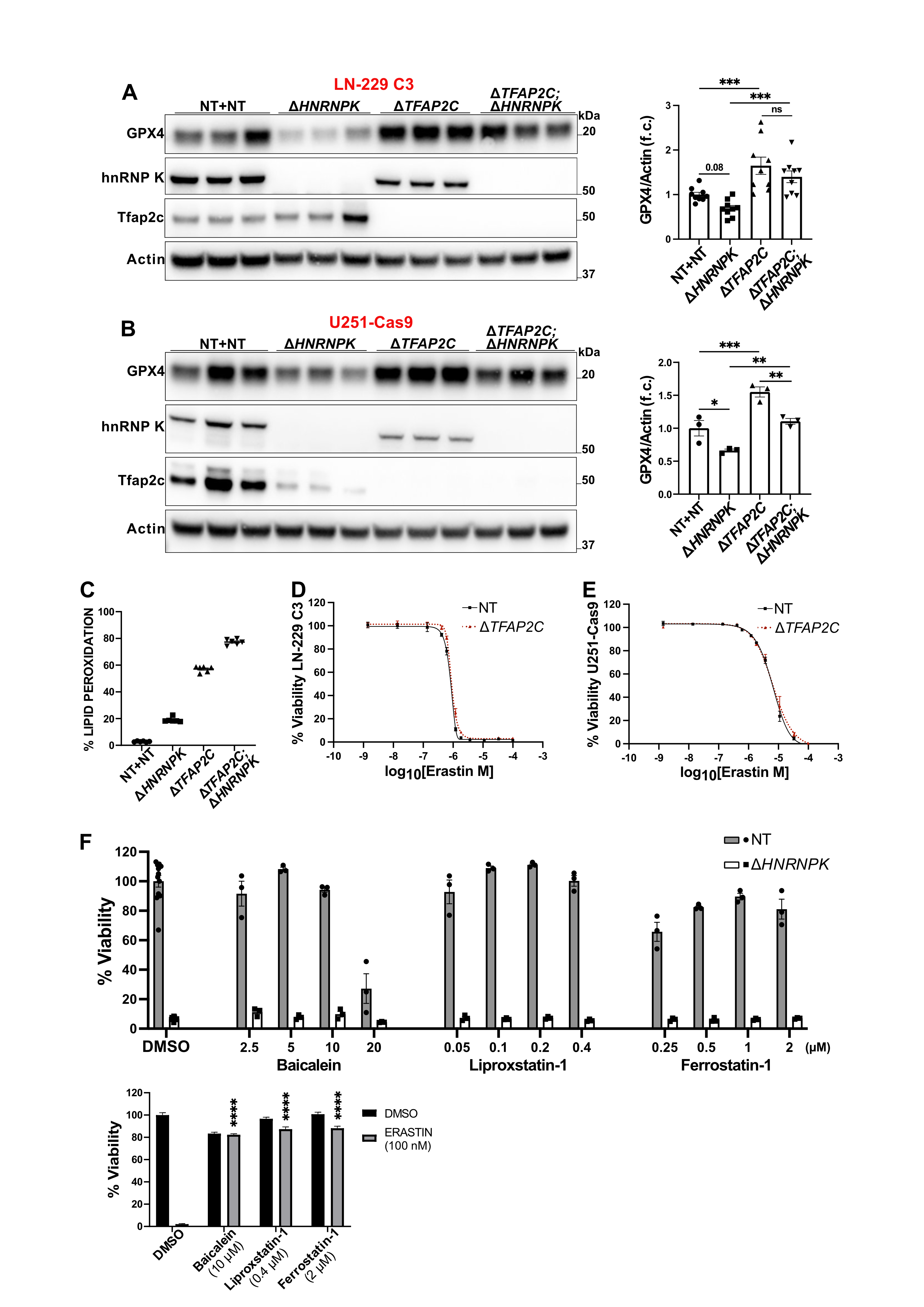

Supplement: S5 Fig — A-B. GPX4 protein after HNRNPK and TFAP2C ablation. n = 3 or 9. C. Percentage of ΔTFAP2C or NT-unmodified LN-229 C3 cells showing lipid peroxidation 4 days after delivering HNRNPK and NT qgRNAs (Liperfluo staining). 6 individually treated wells. D-E. Viability of ΔTFAP2C cells treated with erastin or DMSO (CellTiter-Glo assay). Results are normalized against the DMSO-treated cells. 4 individually treated wells. F. Viability of LN-229 C3 cells treated with erastin as a control (bottom) or transduced with HNRNPK or NT qgRNAs and supplemented with anti-ferroptosis drugs (top) (CellTiter-Glo assay). Results are normalized on the DMSO/NT condition. ≥ 3 individually treated wells. Data information: n represents independent experiments. f.c.: fold change. Mean ± SEM. ns: p > 0.05, *: p < 0.05, **: p < 0.01, ***: p < 0.001, ****: p < 0.0001 (Two-way ANOVA Uncorrected Fisher’s LSD in A-B and Dunnett’s test in F). (TIFF) [file ppat.1014056.s005.tiff]

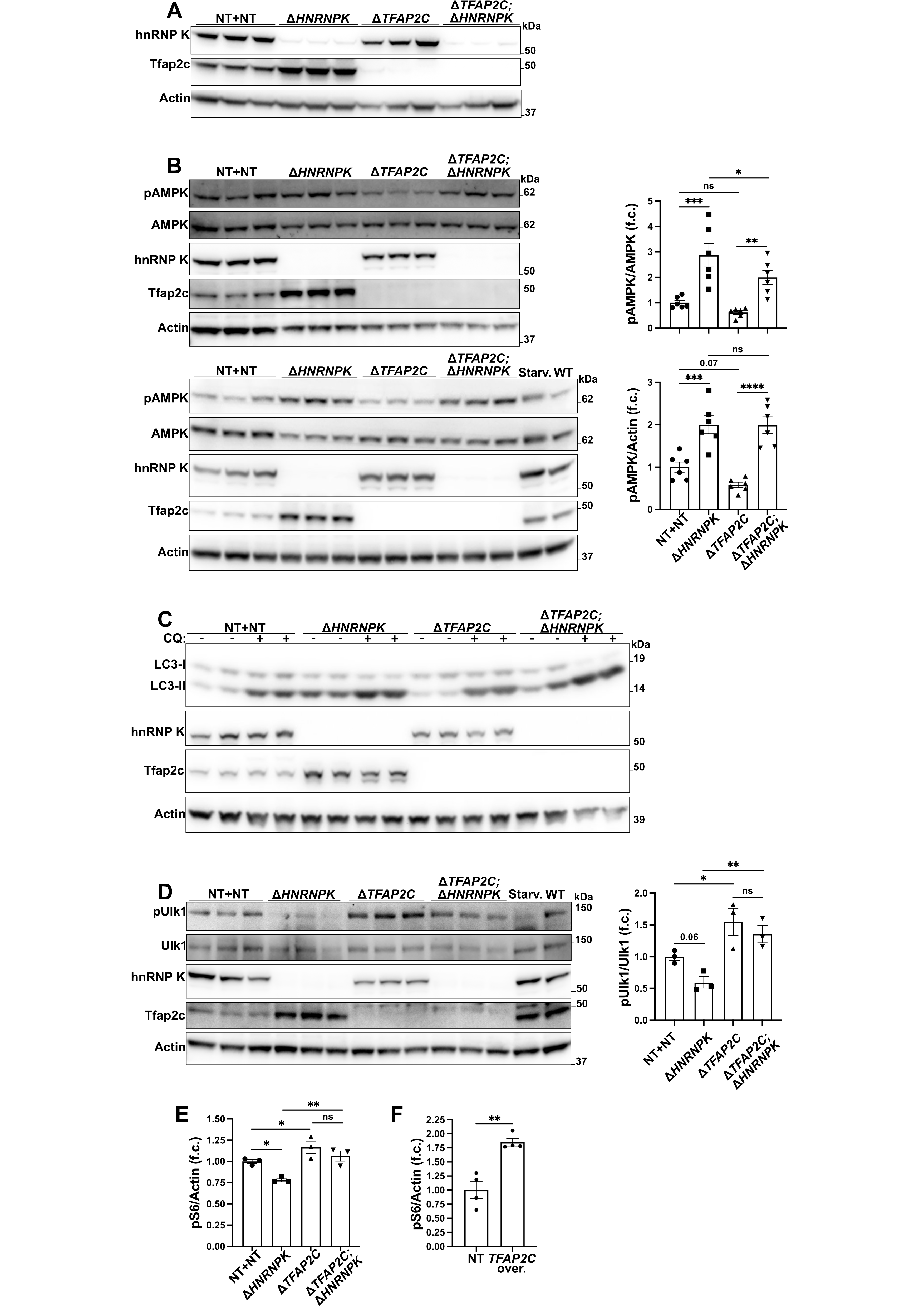

Supplement: S6 Fig — A. Ablations of HNRNPK and TFAP2C for RNA-seq samples. B, D. AMPK (B) and Ulk1 (D) phosphorylation upon deletion of HNRNPK and TFAP2C in LN-229 C3 cells. 6h HBSS-starvation (Starv.) was used as a positive control. n = 6 and 3. C. LC3-II protein in LN-229 C3 cells after HNRNPK and TFAP2C ablation. 4 hours 100 μM chloroquine (CQ). E-F. Absolute S6 phosphorylation quantification from Fig 5E (E) and Fig 5F (F). Data information: n represents independent experiments. f.c.: fold change. Mean ± SEM. ns: p > 0.05, *: p < 0.05, **: p < 0.01, ***: p < 0.001, ****: p < 0.0001 (Two-way ANOVA Uncorrected Fisher’s LSD. Unpaired t-test in F). (TIFF) [file ppat.1014056.s006.tiff]

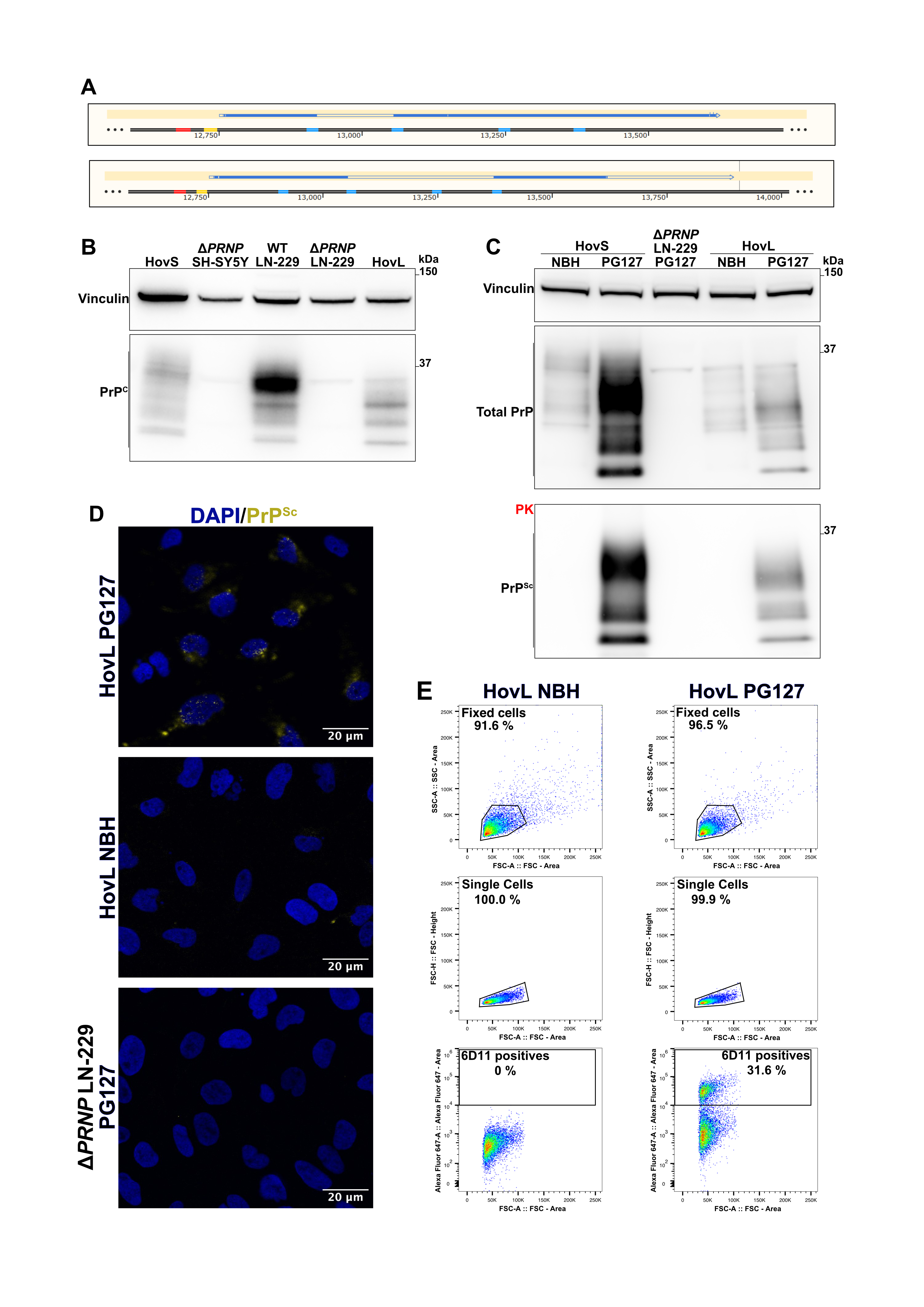

Supplement: S7 Fig — A. qgRNAs (blue segments in reference sequence) and Cas9 transiently transfected in LN-229 cells promoted two major PRNP deletions from position 12922–13055 and from 13055 to 13372. B. Western blot showing the lack of the human PrPC protein in the LN-229ΔPRNP from A and the expression of the ovine PrPC in the resulting HovL cells. HovS and SH-SY5YΔPRNP cells were used as controls for the “ovinization” and PRNP ablation, respectively. C. Proteinase K (PK) digested (bottom) and undigested (top) western blots showing, respectively, PrPSc and the total PrP in LN-229ΔPRNP and HovL cells inoculated either with PG127 prion-infected Brain Homogenate (PG127) or with Not-infectious Brain Homogenate (NBH). PG127-infected and NBH mock-infected HovS cells were used as positive and negative controls, respectively. D-E. Imaging (D) and flow cytometry analysis (E) of anti-PrPSc 6D11 antibody signal in PG127-infected HovL or LN-229ΔPRNP and in NBH HovL cells. (TIFF) [file ppat.1014056.s007.tiff]

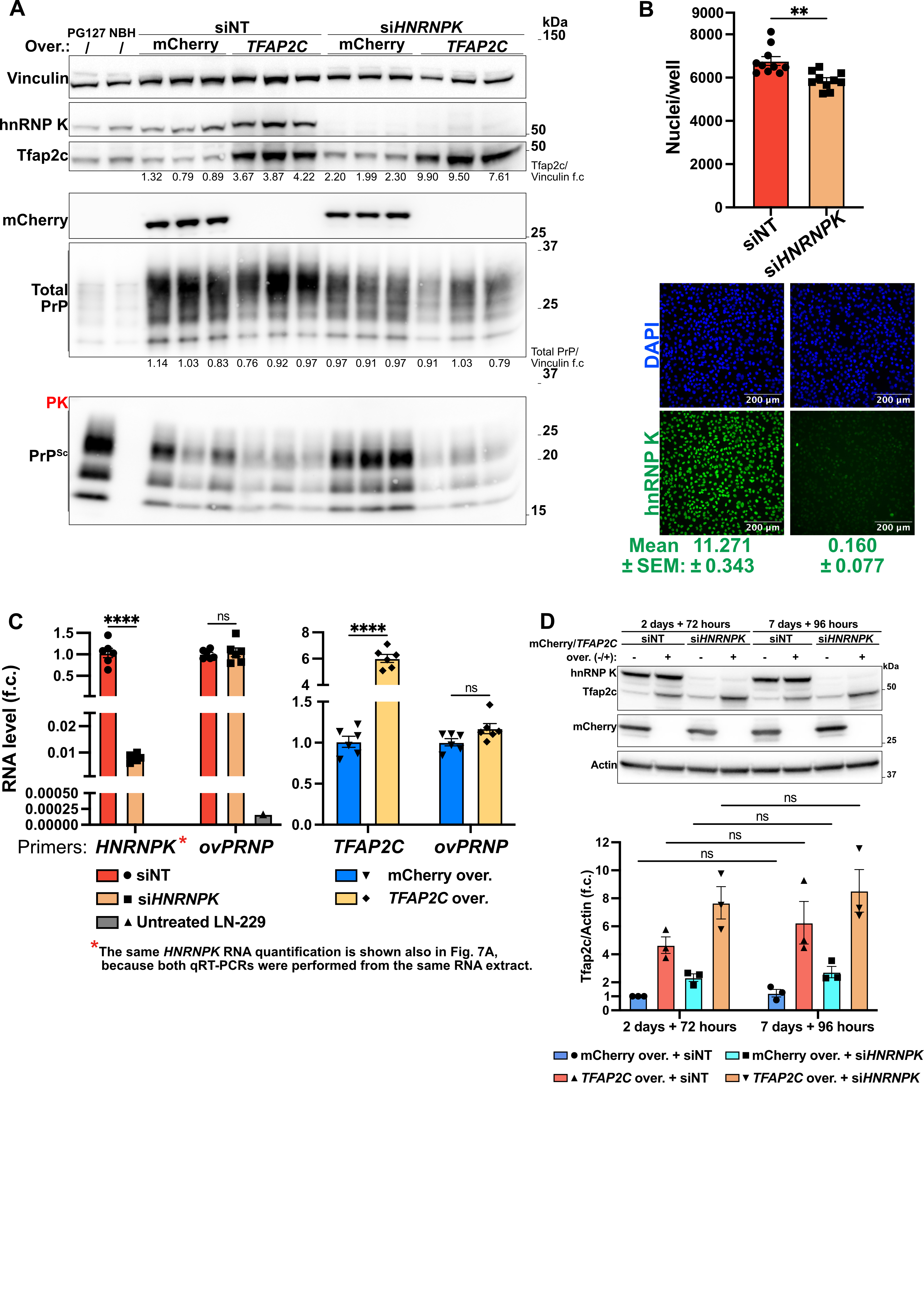

Supplement: S8 Fig — A. Proteinase K (PK) digested (bottom) and undigested (top) western blots showing, respectively, PrPSc and total PrP after HNRNPK silencing (96 hours) and TFAP2C overexpression (192 hours) in PG127-infected HovL cells. n = 3. B. Cell density (nuclei per well) (top) after HNRNPK silencing (96 hours) in PG127-infected HovL cells. Representative image and quantification of hnRNP K intensity per cell (mean ± SEM) (bottom). 10 individually treated wells. C. qRT-PCR showing HNRNPK and ovPRNP RNA upon HNRNPK silencing (96 hours) (left), and TFAP2C and ovPRNP RNA upon TFAP2C overexpression (192 hours) (right) in PG127-infected HovL cells. LN-229 cells were used as a negative control for ovPRNP expression. n = 6. *The same RNA extract was used for the qRT-PCR shown in Fig 7A. D. Representative blot (top) showing Tfap2c protein levels after its overexpression for 2 or 7 days, followed by HNRNPK downregulation for 72 or 96 hours, respectively in PG127-infected HovL cells. Quantification (bottom) n = 3. Data information: Non-targeting siRNA (siNT) and mCherry overexpression were used as controls. qRT-PCR results are normalized against GAPDH expression. n represents independent experiments. f.c.: fold change. Mean ± SEM. ns: p > 0.05, **: p < 0.01, ****: p < 0.001 (Unpaired t-test in B. Multiple Unpaired t-test Holm- Šídák method in C-D). (TIFF) [file ppat.1014056.s008.tiff]

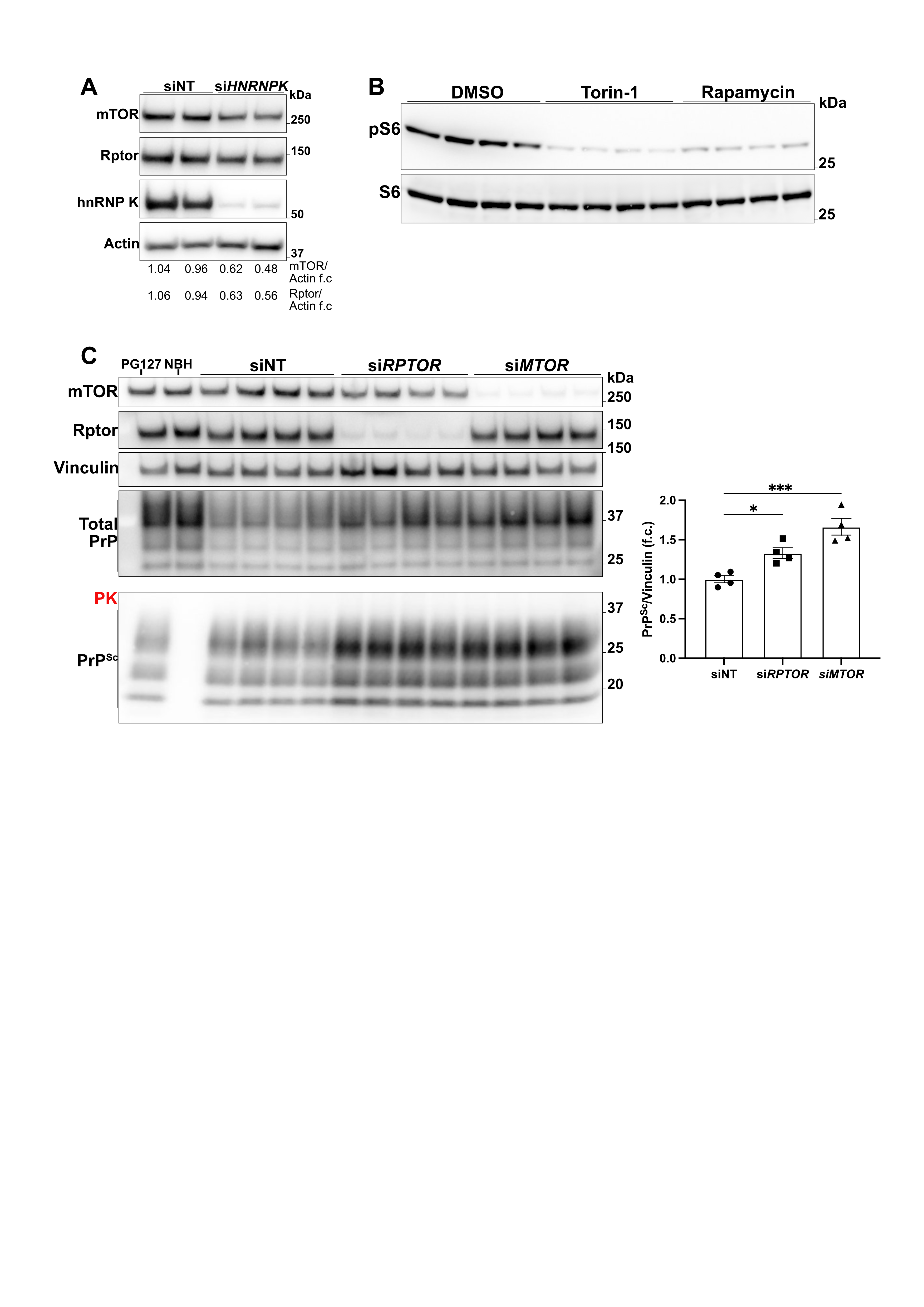

Supplement: S9 Fig — A. mTOR and Rptor protein upon HNRNPK silencing in PG127-infected HovL cells. B. S6 protein phosphorylation in PG127-infected HovL cells treated with 500 nM of Torin-1 or Rapamycin (72 hours) (Same samples used for Fig 7C). C. PK digested (bottom) and undigested (top) western blots showing, respectively, PrPSc and total PrP after RPTOR or MTOR knockdown (96 hours) in PG127-infected HovL cells. n = 4. Data information: Non-targeting siRNA (siNT) was used as control. n represents independent experiments. f.c.: fold change. Mean ± SEM. *: p < 0.05, ***: p < 0.001 (One-way ANOVA Dunnett’s test in C). (TIFF) [file ppat.1014056.s009.tiff]
