# Supplementary material for: Prion propagation is controlled by a hierarchical network involving the nuclear Tfap2c and hnRNP K factors and the cytosolic mTORC1 complex
Source: PLoS Pathog. 2026 Apr 20;22(4):e1014056. doi: 10.1371/journal.ppat.1014056 (PMC13108870; doi:10.1371/journal.ppat.1014056)

Fig 1B:

The membrane was cut at 75kDa (1 and 2). Due to a techincal issue, the ladder was cut out from membrane 1. Membrane 1 was incubated with anti-Cas9. Membrane 2 was incubated in succession with anti-hnRNP K (2), anti-actin (3) without stripping.

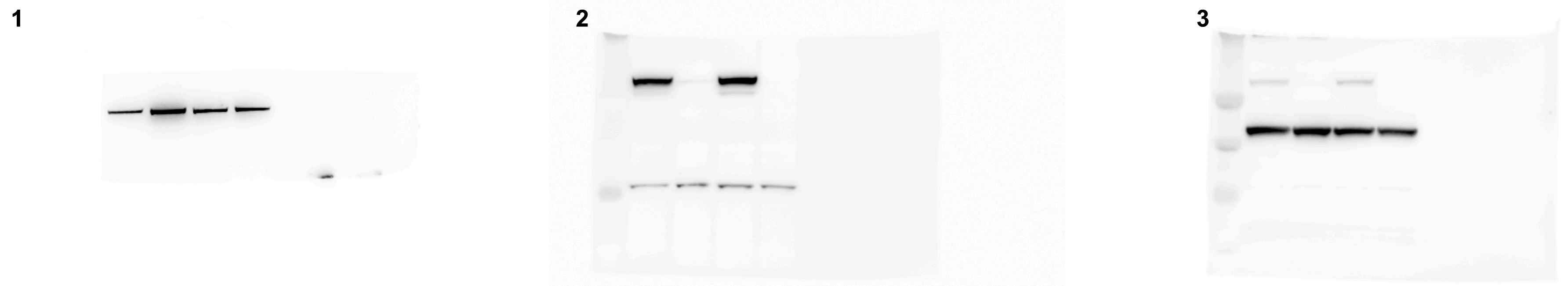

Fig 1C:

The membrane was cut at 75kDa (1 and 2). For techincal problem membrane ladder was cut from membrane 1. Membrane 1 was incubated with anti-Cas9. Membrane 2 was incubated in succession with anti-hnRNP K (2), anti-actin (3) without stripping.

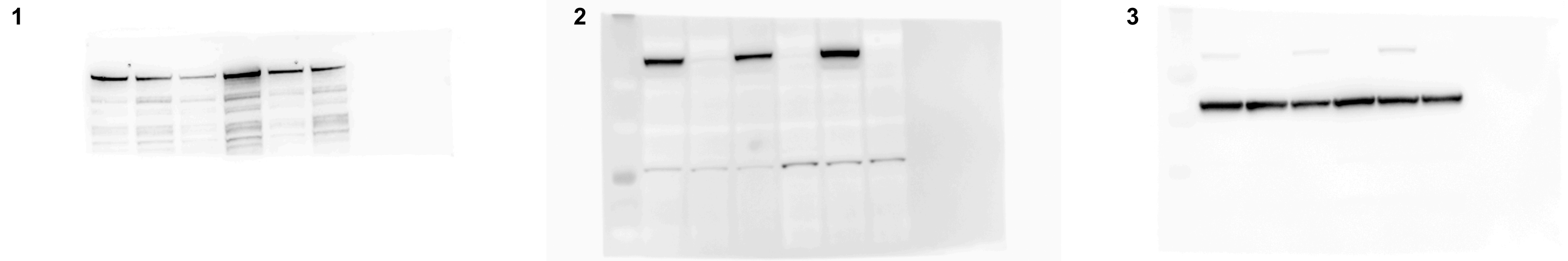

Fig 1D:

The membrane was cut at 75kDa (1 and 2). For techincal problem membrane ladder was cut from membrane 1. Membrane 1 was incubated with anti-Cas9. Membrane 2 was incubated in succession with anti-hnRNP K (2), anti-actin (3) without stripping.

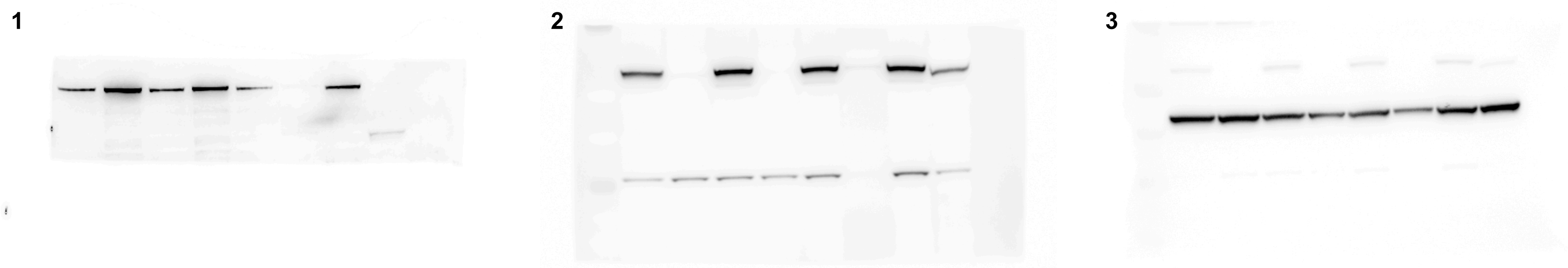

Fig 2A:

The membrane was incubated in succession with anti-hnRNP K (1), anti-Tfap2c (2), anti-actin (3) without stripping.

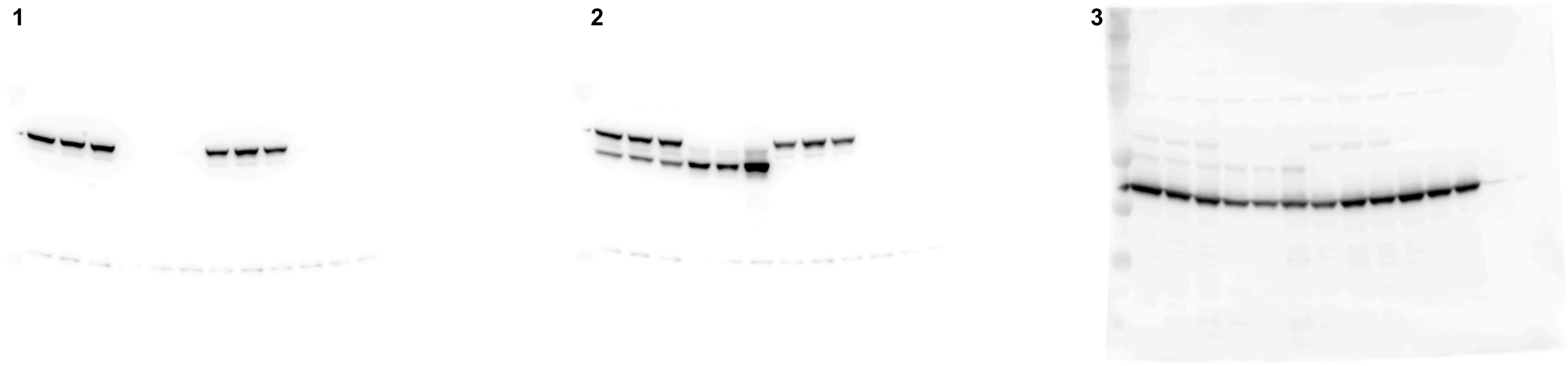

Fig 2B:

The membrane was incubated in succession with anti-hnRNP K (1), anti-Tfap2c (2), anti-Actin (3) without stripping

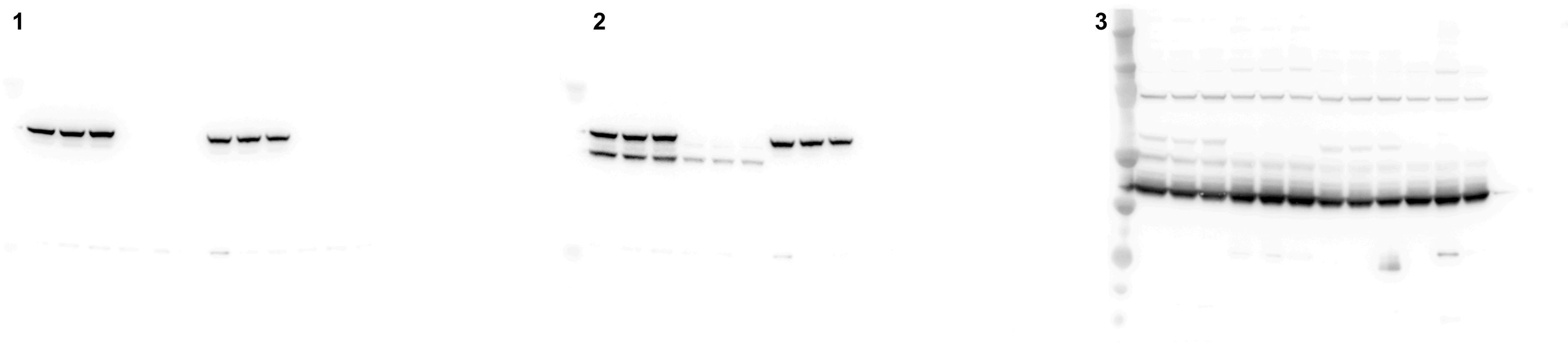

Fig 2C:

The membrane was incubated in succession with anti-Tfap2c (1), anti-hnRNP K (2). After stripping, anti-Actin (3) was used.

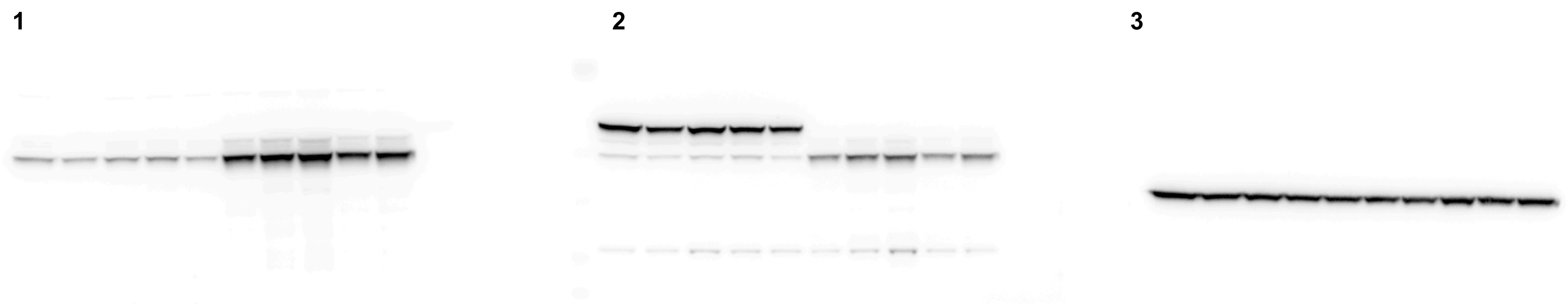

Fig 2E:

The membrane was incubated in succession with anti-Tfap2c (1). After stripping we used anti-Actin (2), followed by anti-hnRNP K (3).

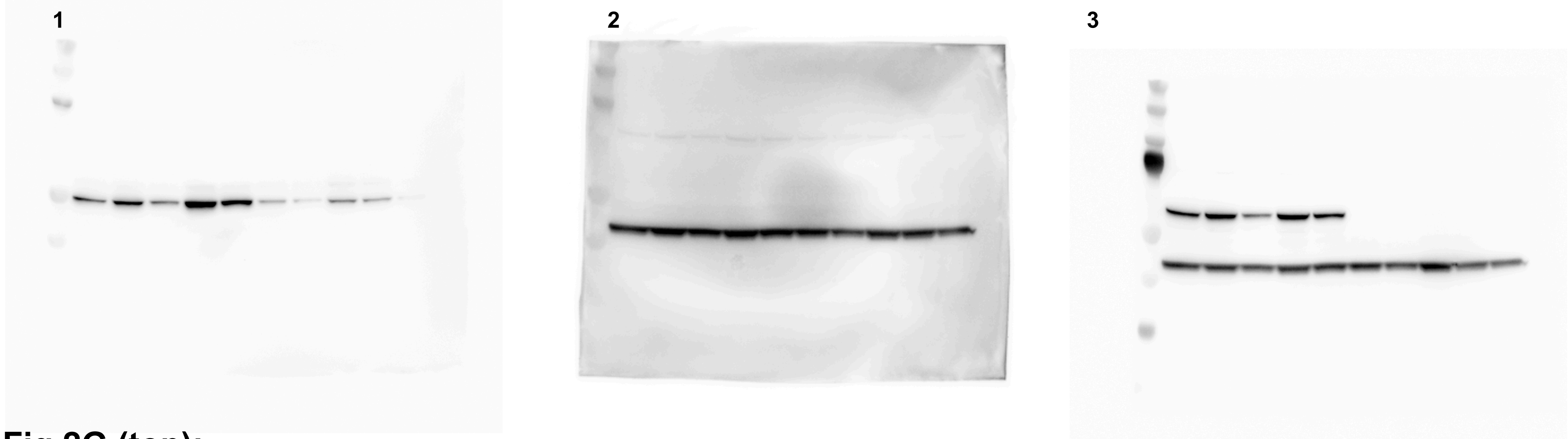

Fig 2G (top):

The membrane was cut below 37 kDa (1 and 2). Membrane 1 was incubated with anti-Tfap2c. Membrane 2 was incubated with anti-mCheery. Membrane 1 was stripped and cut below 50 kDa (3 and 4). Mebrane 3 (45-250 kDa) was incubated with anti-hnRNP K (3). Membrane 4 (35-45 kDa) was incubated with anti-Actin.

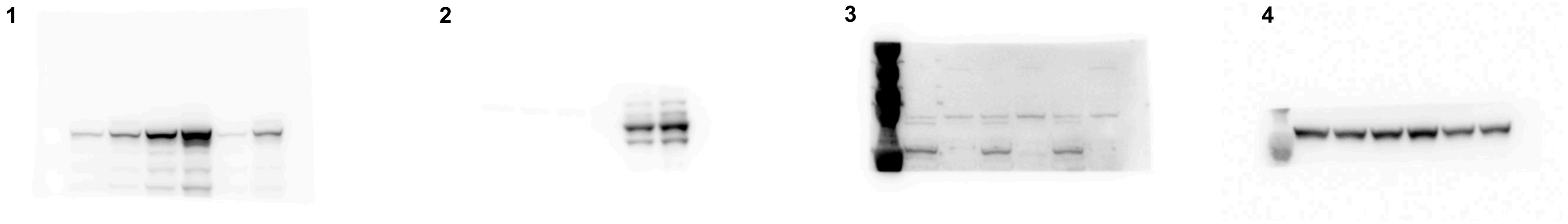

Fig 2G (bottom):

The membrane was incubated in succession with anti-Tfap2c (1), anti-hnRNP K (2), anti-mCheery (3), anti-Actin (4) without stripping.

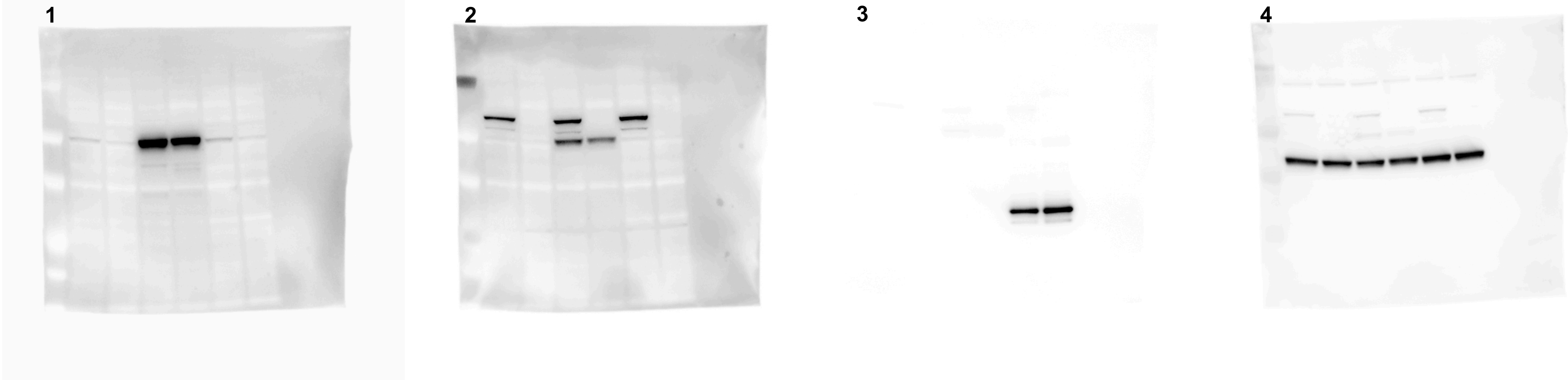

Fig 2H (left):

The Co-IP membrane (left) was incubated in succession with anti-hnRNP K (1), and after stripping, with anti-Tfap2c (2). The FT membrane (right) was incubated with anti-Tfap2c (3), stripped, and incubated in succession with anti-hnRNPK K (4) and anti-Actin (5) without stripping.

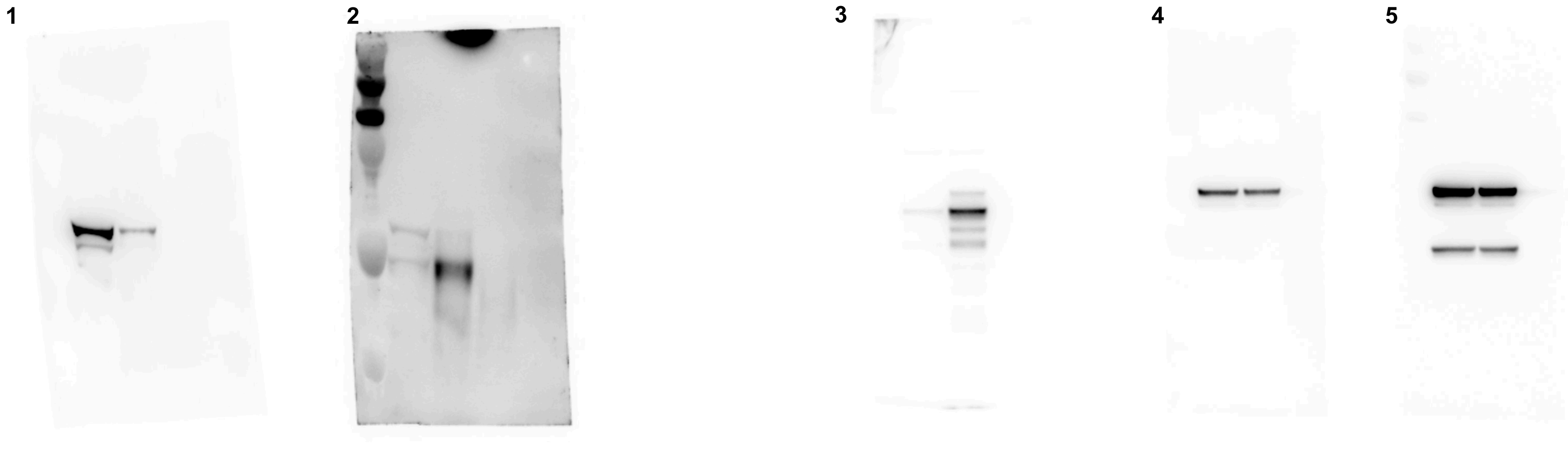

Fig 2H (right):

The Co-IP membrane (left) was incubated in succession with anti-Tfap2c (1), and after stripping, with anti-hnRNP K (2). The FT membrane (right) was incubated with anti-Tfap2c (3), stripped, and incubated in succession with anti-hnRNPK K (4) and anti-Actin (5) without stripping.

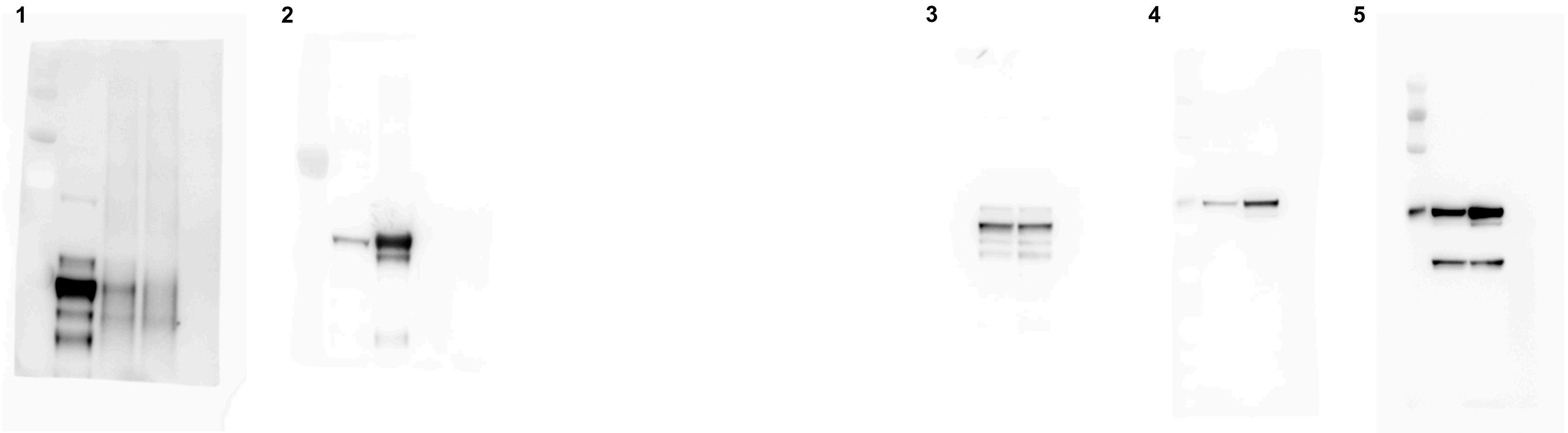

Fig 2I (left):

The Co-IP membrane (left) was incubated in succession with anti-hnRNP K (1), and after stripping, with anti-Tfap2c (2). The FT membrane (right) was incubated with anti-Tfap2c (3), stripped, and incubated in succession with anti-hnRNPK K (4) and anti-Actin (5) without stripping.

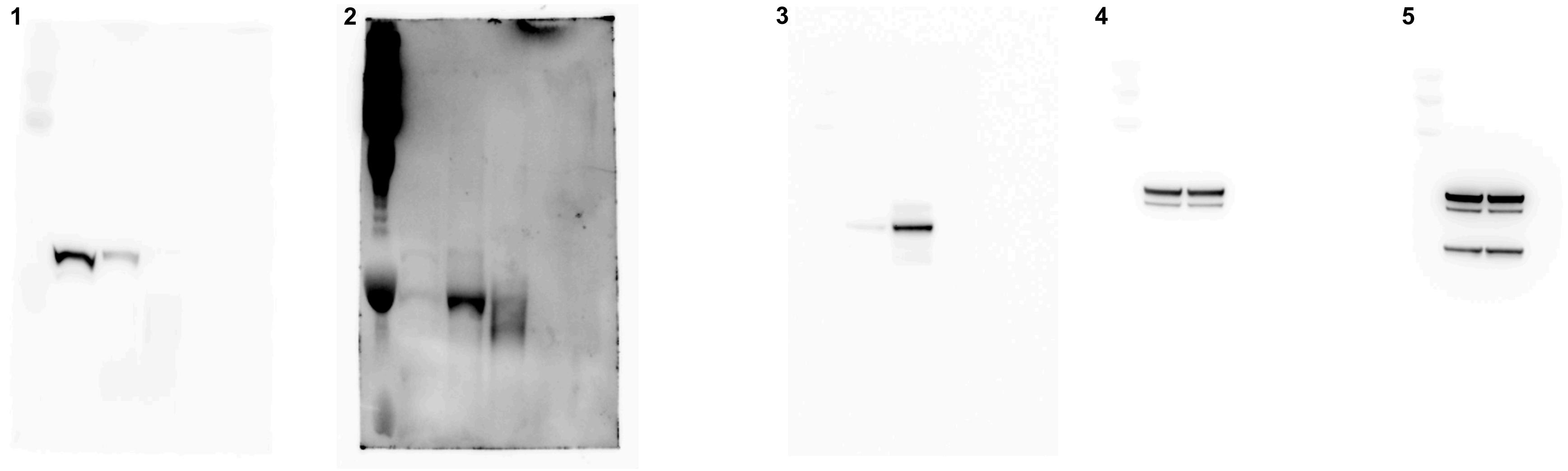

Fig 2I (right):

The Co-IP membrane (left) was incubated in succession with anti-Tfap2c (1), and after stripping, with anti-hnRNP K (2). The FT membrane (right) was incubated with anti-Tfap2c (3), stripped, and incubated in succession with anti-hnRNPK K (4) and anti-Actin (5) without stripping. The last lane is the same sample input as the first lane, but at a different concentration.

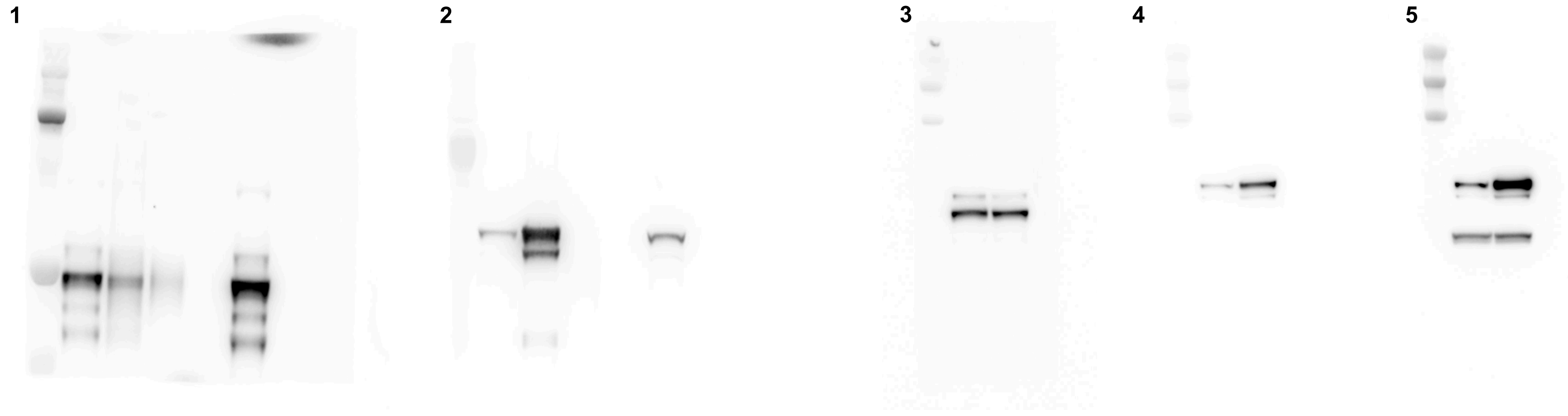

Fig 3A:

The membrane was cut at 75kDa (1 and 6). The membrane 1 (10-75 kDa) was incubated in succession with anti-cleaved Caspase3 (1), anti-Tfap2c (2), anti-hnRNP K (3), anti-Actin (4), anti-full Caspase3 (5) without stripping. Membrane 6 (75-250 kDa) was incubated with anti-PARP.

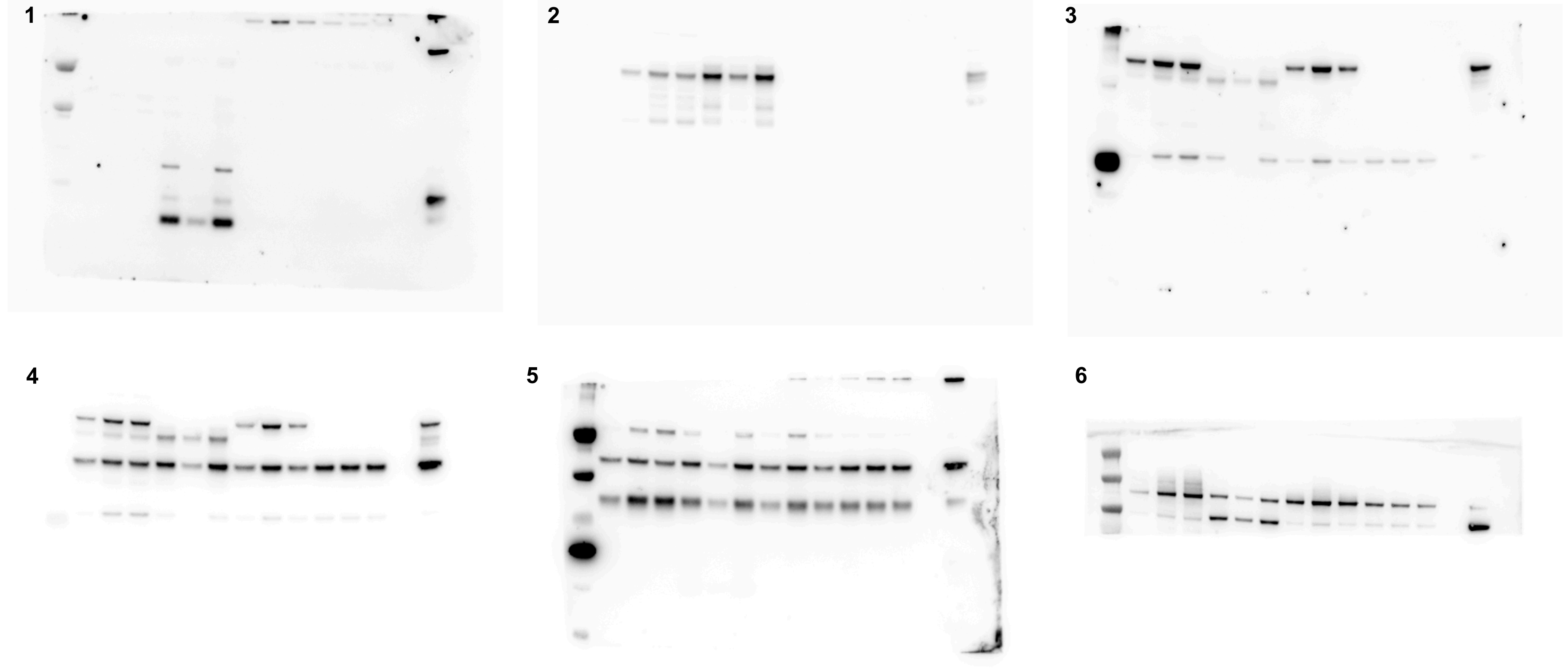

Fig 3B:

The membrane was cut at 22kDa (1), 37kDa (2) and 70kDa (3 and 6). Membrane 1 (10-22 kDa) was incubated with anti-cleaved Caspase3. Membrane 2 (22-37 kDa) was incubated with anti-full Caspase3. Membrane 3 (37-70 kDa) was incubated in succession with anti-hnRNP K (3), anti-Tap2c (4), anti-Actin (5) witout stripping. Membrane 6 (70-250 kDa) was incubated with anti-PARP.

1

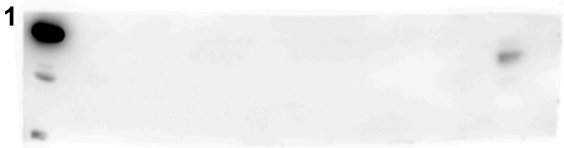

2

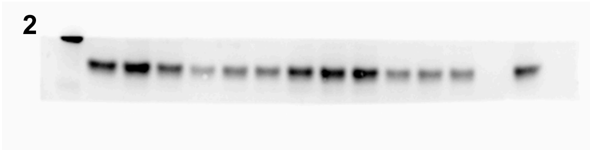

3

4

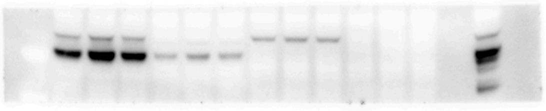

5

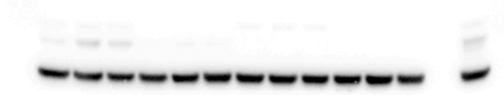

6

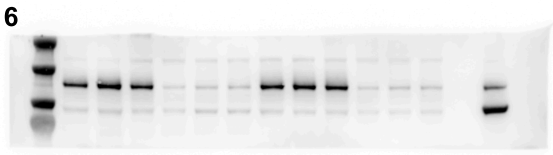

Fig 5B

The membrane was cut between 22-28 kDa (SeeBlue Ladder in MOPS) (1 and 4). Membrane 1 was incubated in succession with anti-hnRNP K (1), anti-Tfap2c (2) and after stripping with anti-Actin (3). Membrane 4 was incubated with anti-LC3B (4).

1

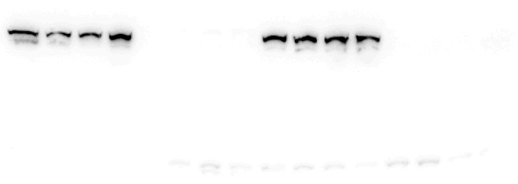

2

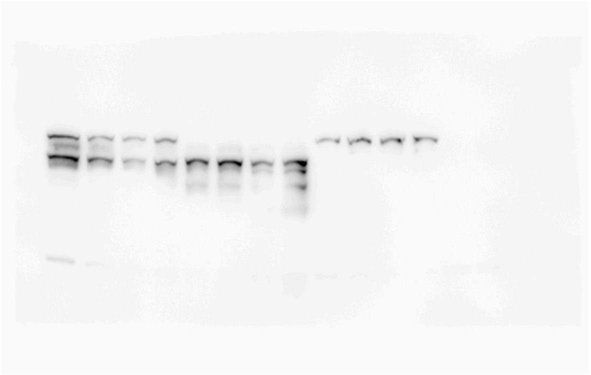

3

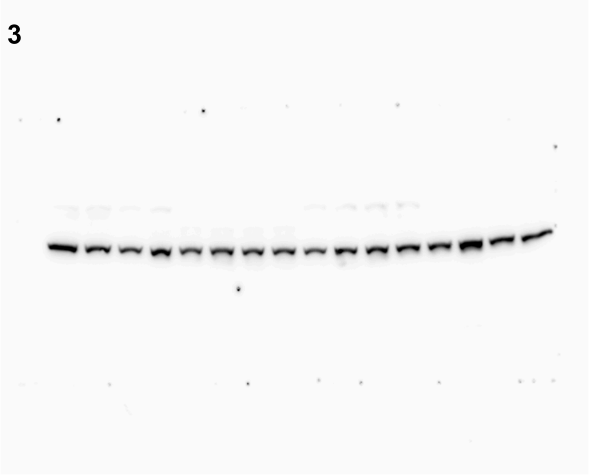

4

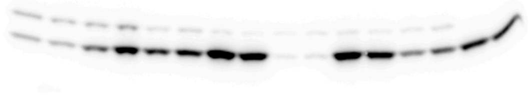

Fig 5D:

The membrane was cut just below 37 kDa (2), at 75 kDa (4) and just below 150 kDa (1). The membrane 75-150 kDa was used for a different unrelated experiment and for this reason is not shown. Membrane 1 (140-300 kDa) was incubated with anti-mTOR. Membrane 2 (10-35 kDa) was initially incubated with anti-4EBP1 (not shown). Membrane 4 (75-140 kDa) was incubated in succession with anti-Tfap2c (4), anti-hnRNP K (5), and after stripping, with anti-Actin (6).

1

4

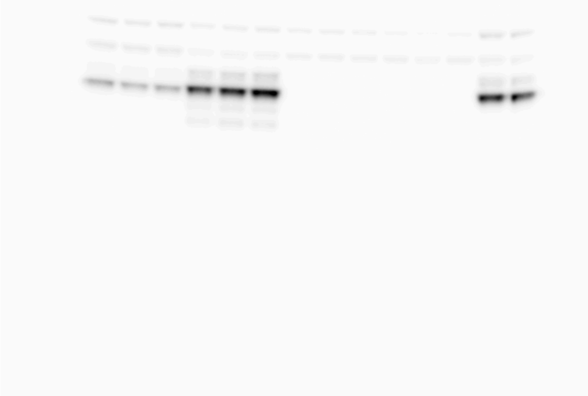

5

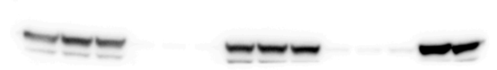

6

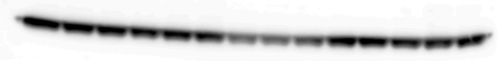

Fig 5E:

The membrane was cut just below 25 kDa, at 37 kDa and just below 75 kDa. Membrane 1 (10-23 kDa) was incubated with anti-4EBP1 (1). Membrane 2 (23-37 kDa) was incubated with anti-pS6 (2). After stripping, membrane 2 was incubated with anti-S6 (3). Membrane 4 (37-73 kDa) was incubated in succession with anti-Tfap2c (4), anti-hnRNP K (5). After stripping, membrane 4/5 was incubated with anti-Actin (6).

1

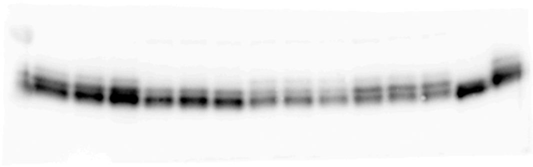

2

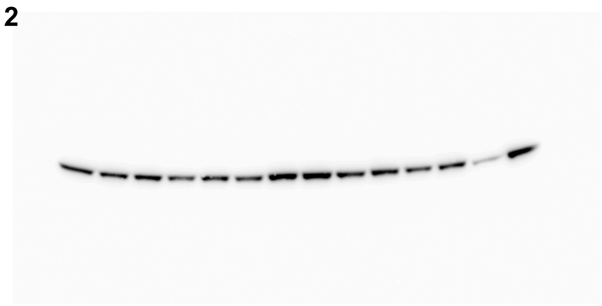

3

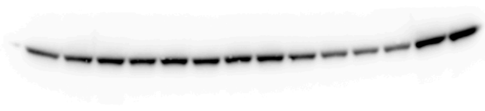

4

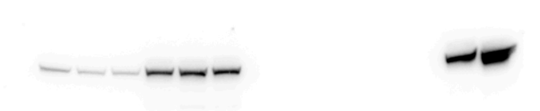

5

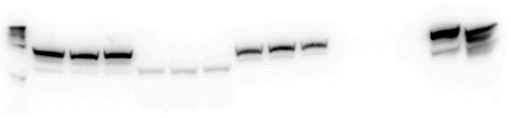

6

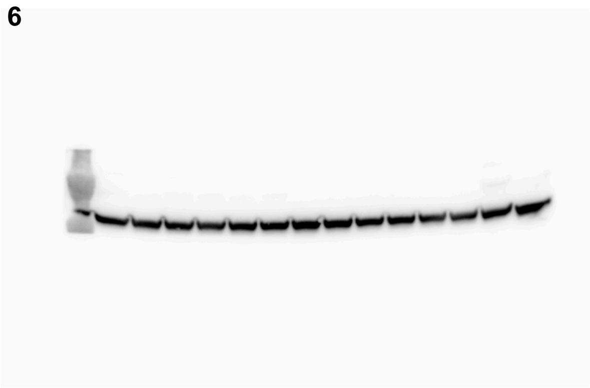

Fig 5F:

The membrane was cut at 100 kDa (1 and 3). Membrane 1 (10-100 kDa) was incubated in succession with anti-4EBP1 (1), anti-pS6 (2) without stripping. Membrane 3 (100-300 kDa) was incubated with anti-mTOR (3). Membrane 1 (10-100 kDa) was then cut at 25 kDa. Membrane 4 (25-100kDa) was incubated with anti-Tfap2c (4). Membrane 4 was cut at 37 kDa and 50 kDa (5 and 6). Membrane 5 (25-37 kDa) was stripped and incubated with anti-S6 (5). Membrane 6 (37-50 kDa) was stripped and incubated with anti-Actin (6).

1

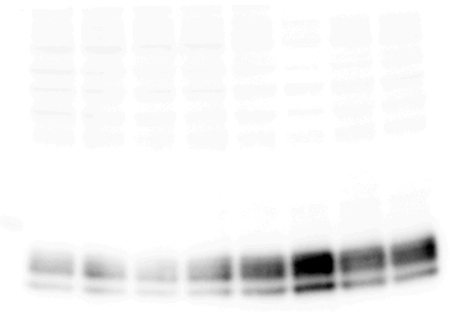

2

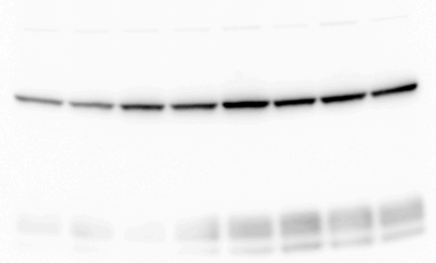

3

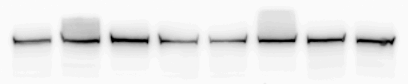

4

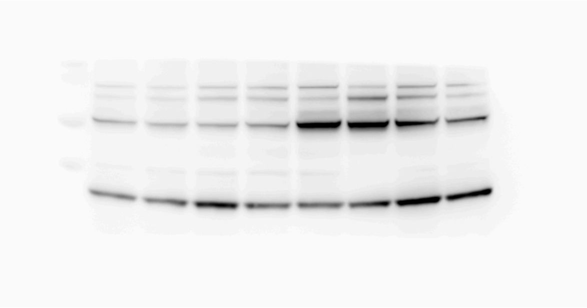

5

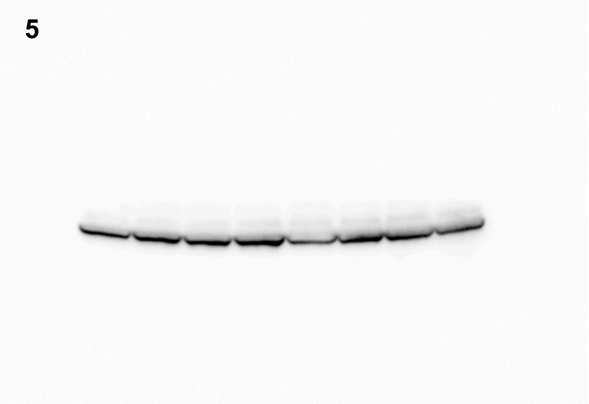

6

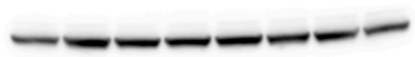

Fig 6A:

The membrane was cut at 75 kDa (1 and 2). Membrane 1 (75-250 kDa) was incubated with anti-Vinculin (1). Membrane 2 (10-75 kDa) was incubated in succession with anti-PrP (POM2) (2), anti-hnRNP K (3), and anti-tfap2c (4). Membrane 2 was then stripped and incubated with anti-mCherry (5). Membrane 6 was transferred with the same samples of membrane 1-5 pre-treated with PK digestion. Membarne 6 was incubated with anti-PrP (POM1) (6).

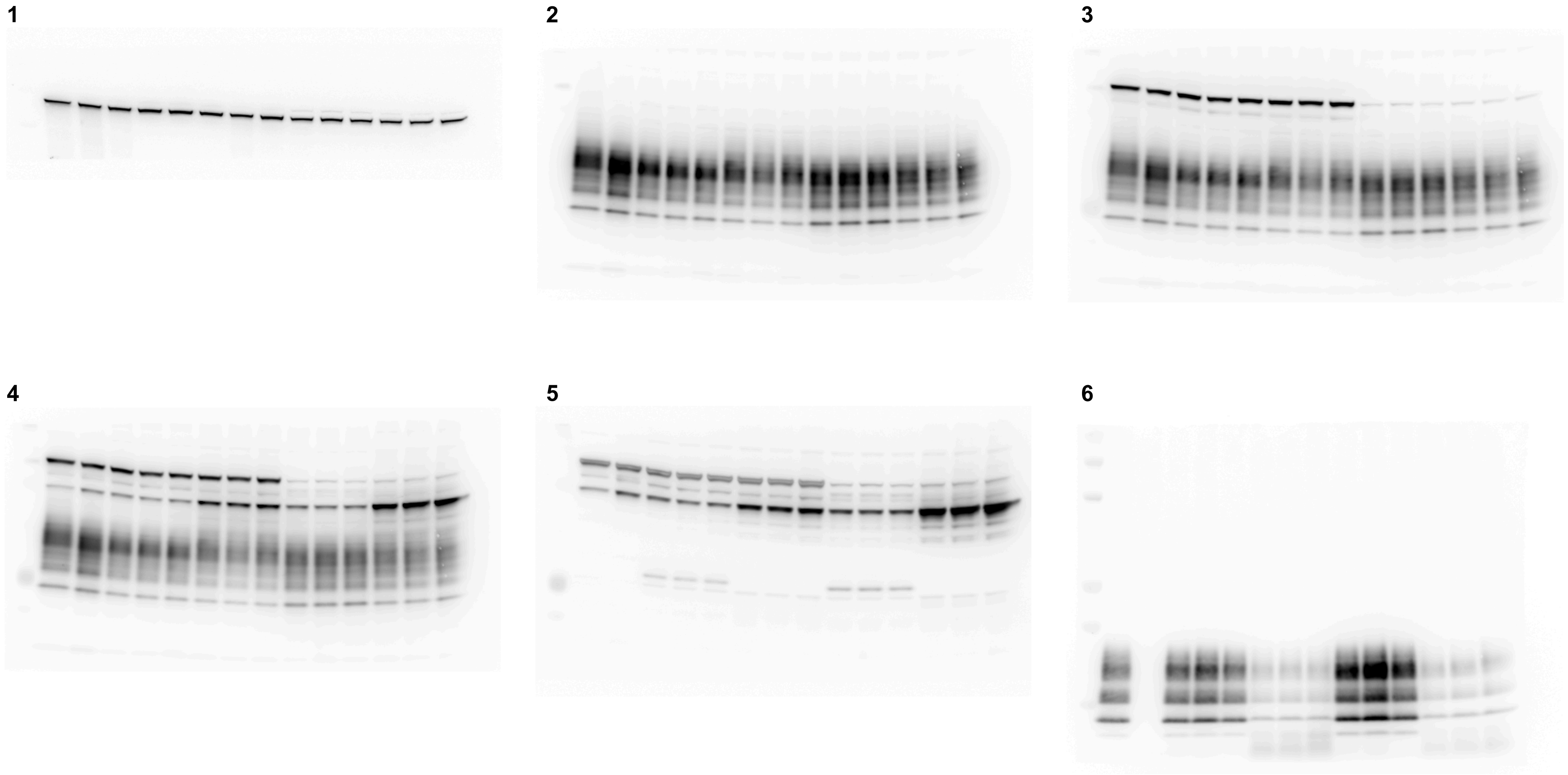

Fig 6C:

The membrane was cut at 75 kDa (1 and 2). Membrane 1 (75-250 kDa) was incubated with anti-Vinculin (1). Membrane 2 (10-75 kDa) was incubated anti-PrP (POM2) (2). Membrane 2 was cut at 35 kDa (3 and 5). Membrane 3 (35-75 kDa) was incubated in succession with anti-Tfap2c (3) and anti-hnRNP K (4) without stripping. Membrane 5 was stripped and then incubated with anti-mCherry (5).

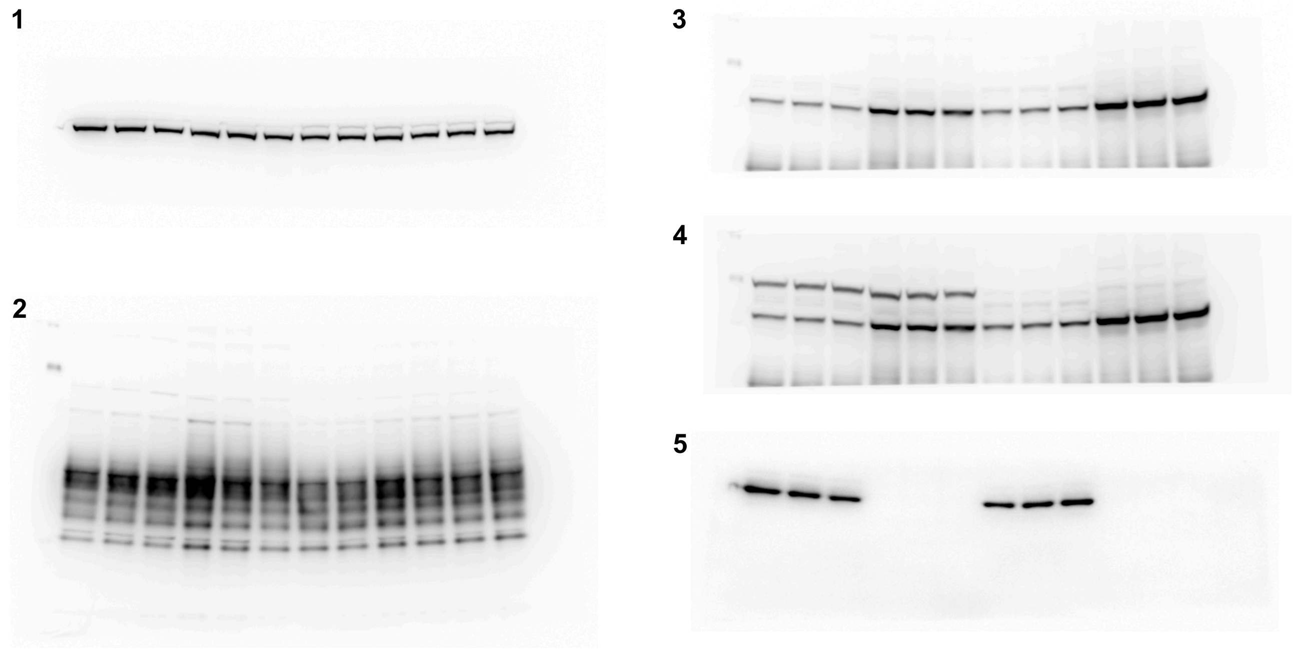

Fig 7B:

The membrane was cut at 75 kDa, just above 20 kDa (1 and 2) and just below 37 kDa (4). Membrane 1 (10-20 kDa) was incubated anti-4EBP1 (1). Membrane 2 (20-37 kDa) was incubated with anti-pS6 (2). Membrane 2 was stripped and then incubated with anti-S6 (3). Membrane 4 (37-75 kDa) was incubated with anti-hnRNP K (4) and then reincubated without stripping with anti-Actin (5).

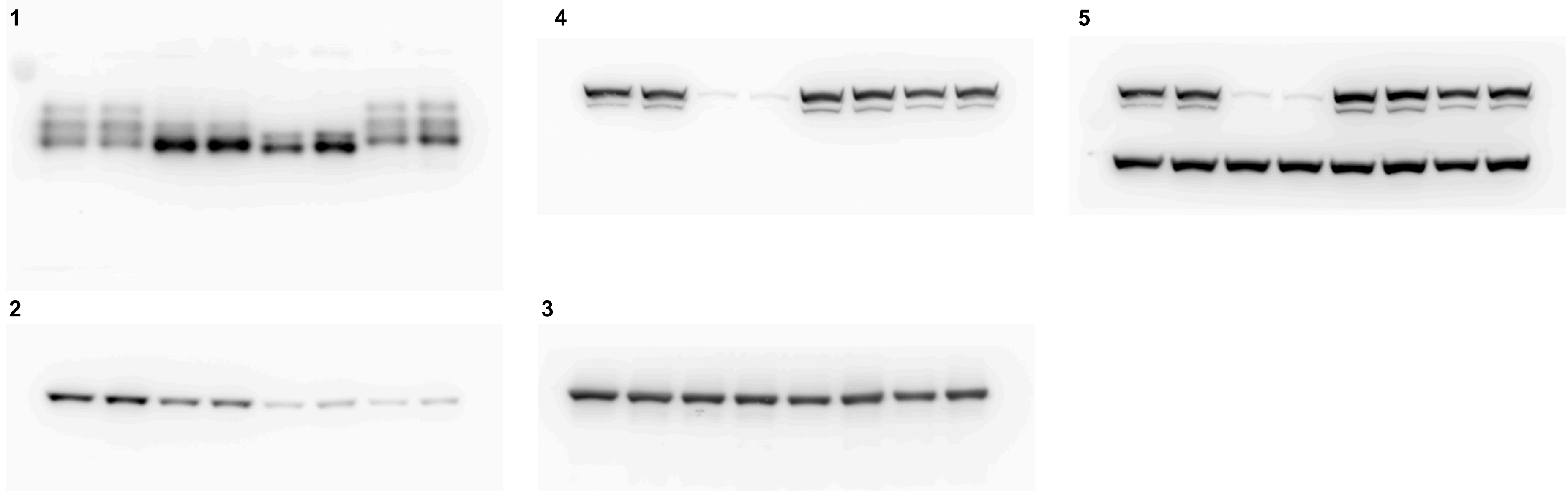

Fig 7C:

The membrane was cut at 75 kDa (1 and 2). Membrane 1 (75-250 kDa) was incubated with anti-Vinculin (1). Membrane 2 (10-75 kDa) was incubated anti-PrP (POM2) (2). Membrane 3 was transferred with the same samples of membrane 1-2 pre-treated with PK digestion. Membarne 3 was incubated with anti-PrP (POM1) (3).

1

2

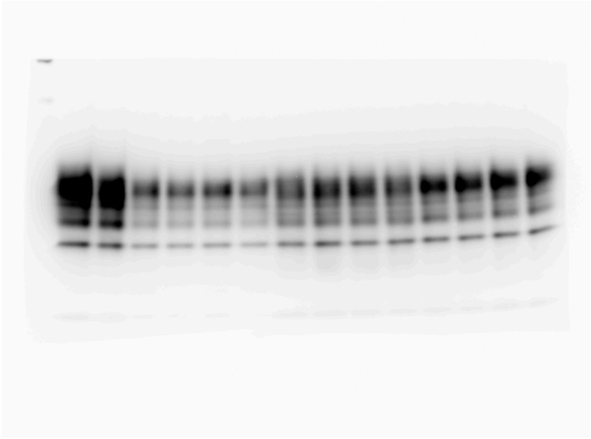

3

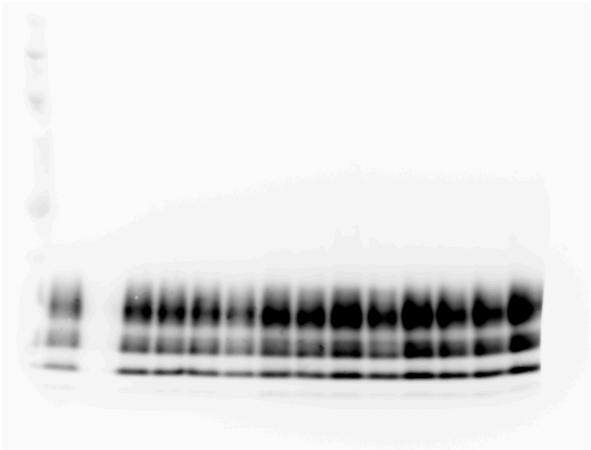

S1A Fig:

The membrane was incubated with anti-Cas9 (1). After stripping, the membrane was incubated with anti-Actin (2).

1

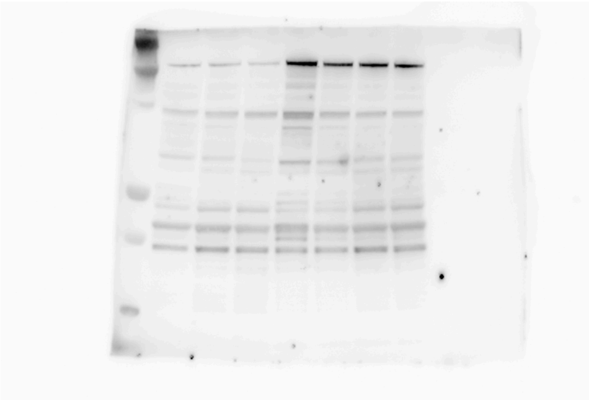

2

S1D Fig:

The membrane was incubated in succession with anti-hnRNP K (1), anti-Actin (2) without stripping. The last 4 lanes were loaded with lysates coming from C3 cells transduced with differented truncated variants of hnRNP K. They do not appear in the cropped figure because they are irrelevat to the aim of the described experiment. They were originally loaded on the same gel as controls for another unrelated experiment.

1

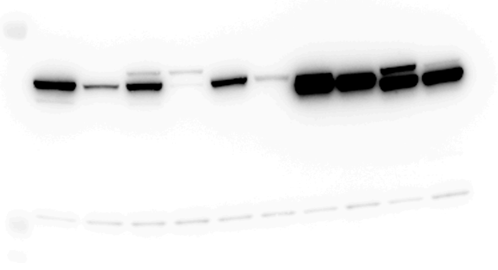

2

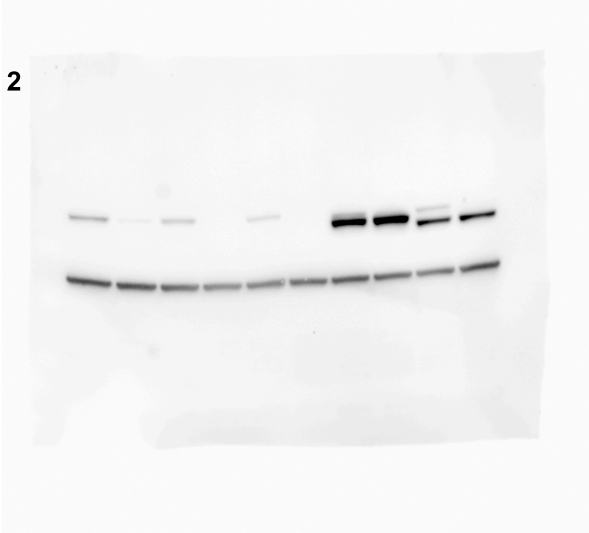

S2H Fig:

The membrane was incubated with anti-Puromycin (1). After stripping it was incubated with anti-hnRNP K (2). After following stripping it was incubated with anti-Actin (3).

1

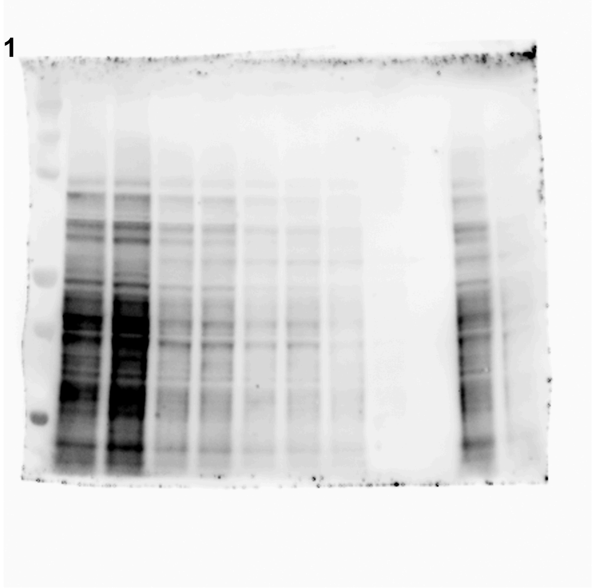

2

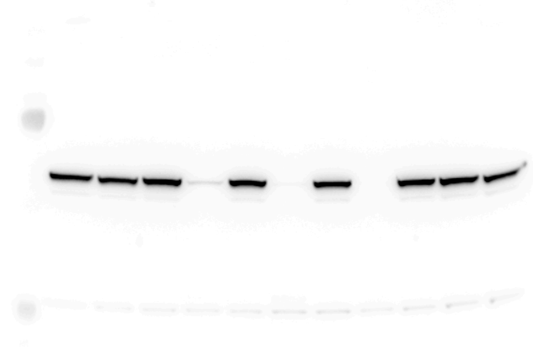

3

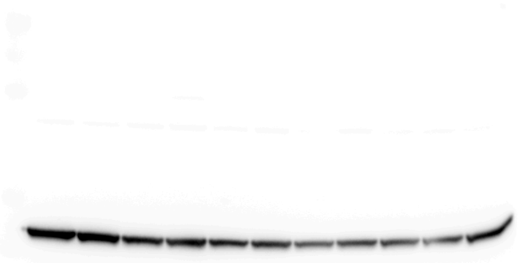

S3D Fig:

The membrane was incubated in succession with anti-hnRNP K (1), anti-Tfap2c (2), anti-Actin (3) without stripping.

1

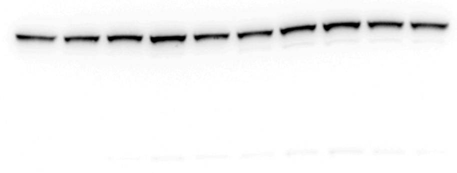

2

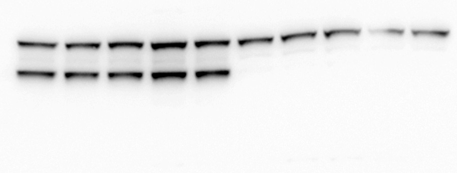

3

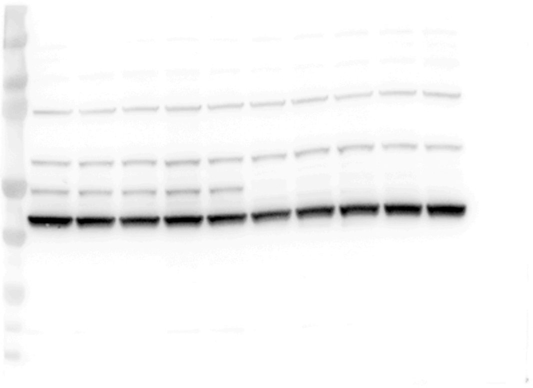

S3E Fig:

The membrane was incubated in succession with anti-hnRNP K (1), anti-Tfap2c (2), anti-Actin (3) without stripping.

1

2

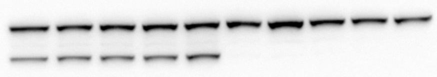

3

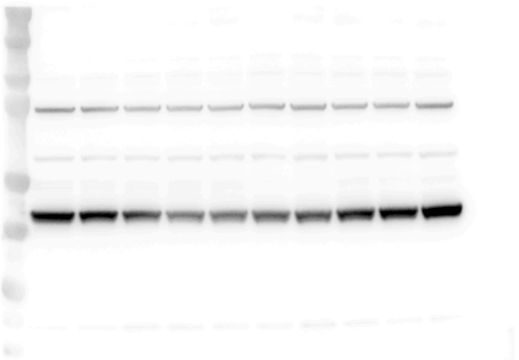

S3F Fig:

The membrane was incubated in succession with anti-hnRNP K (1), anti-Tfap2c (2), anti-Actin (3) without stripping.

1

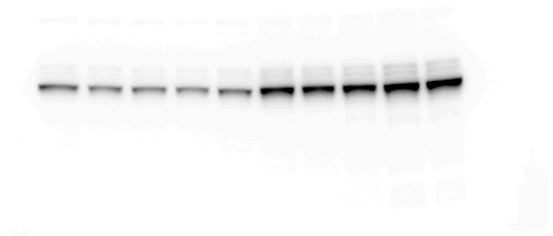

2

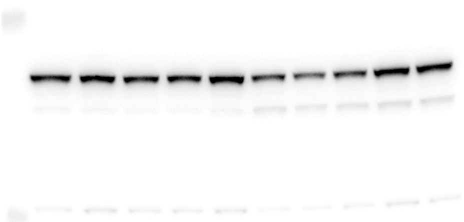

3

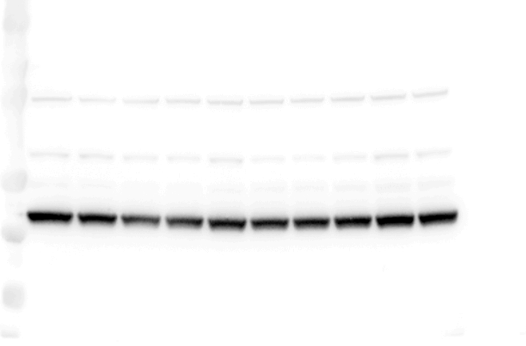

S3H Fig:

The membrane was incubated in succession with anti-hnRNP K (1), anti-Tfap2c (2), anti-Actin (3) without stripping.

1

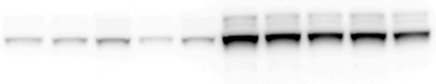

2

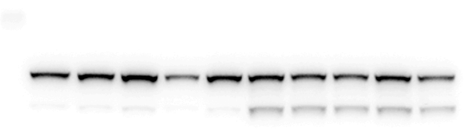

3

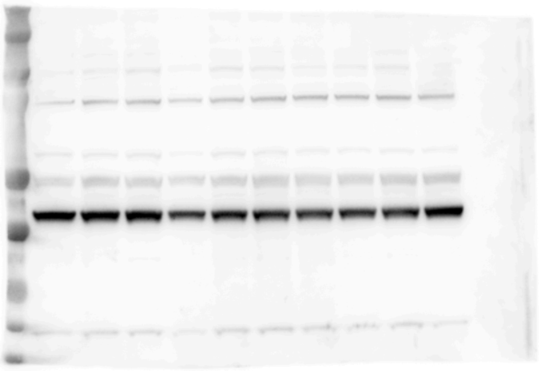

S4C Fig:

The Co-IP membrane was incubated in succession with anti-hnRNP K (1), anti-tfap2c (2) without stripping. The FT membrane was incubated in succession with anti-hnRNP K (3), anti-Actin (4), anti-Tfap2c (5) without stripping.

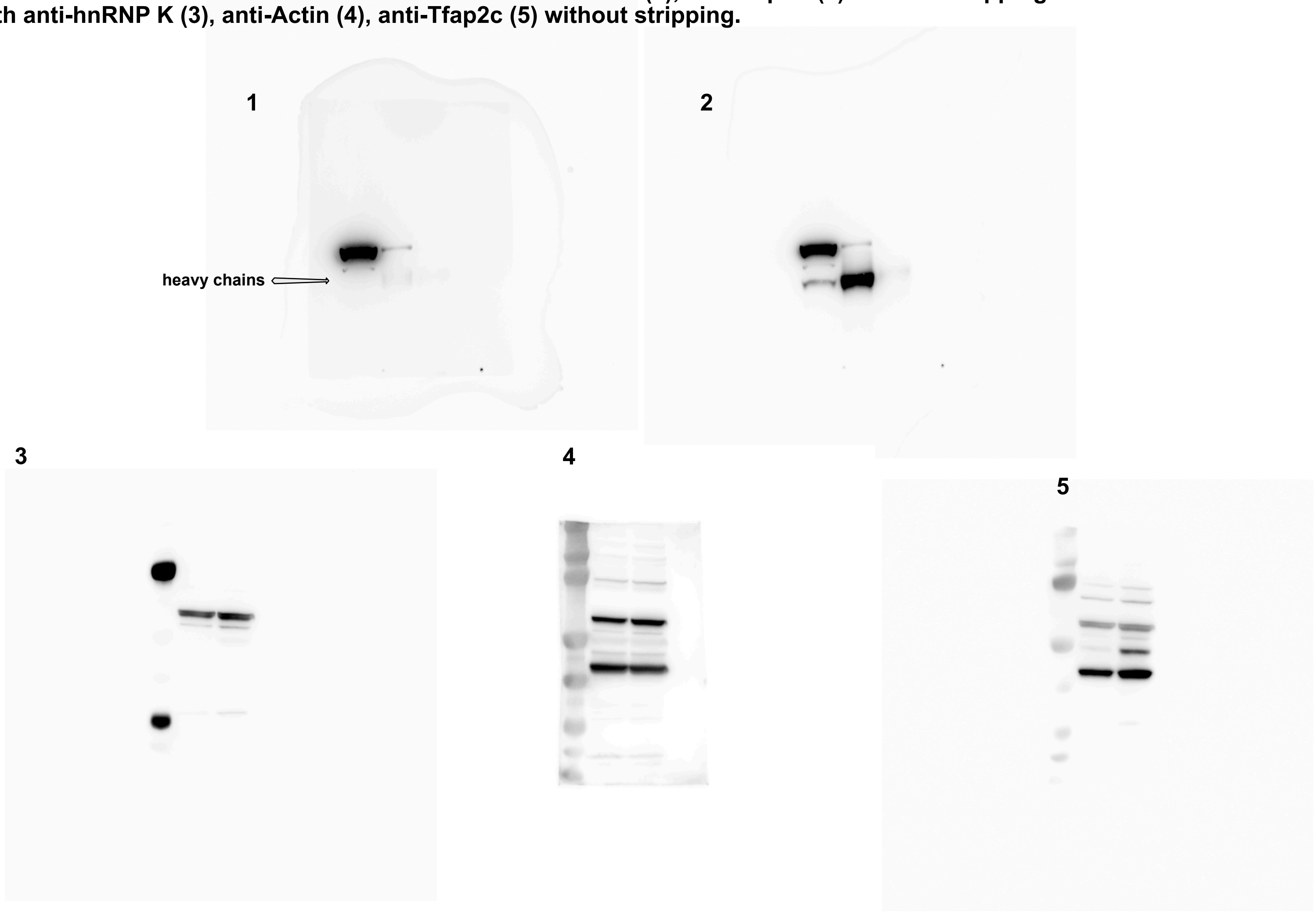

S4D Fig:

The Co-IP membrane was incubated in succession with anti-hnRNP K (1), anti-Tfap2c (2) without stripping. The FT membarne was incubated in succession with anti-hnRNP K (3), anti-Tfap2c (4), anti-Actin (4) without stripping.

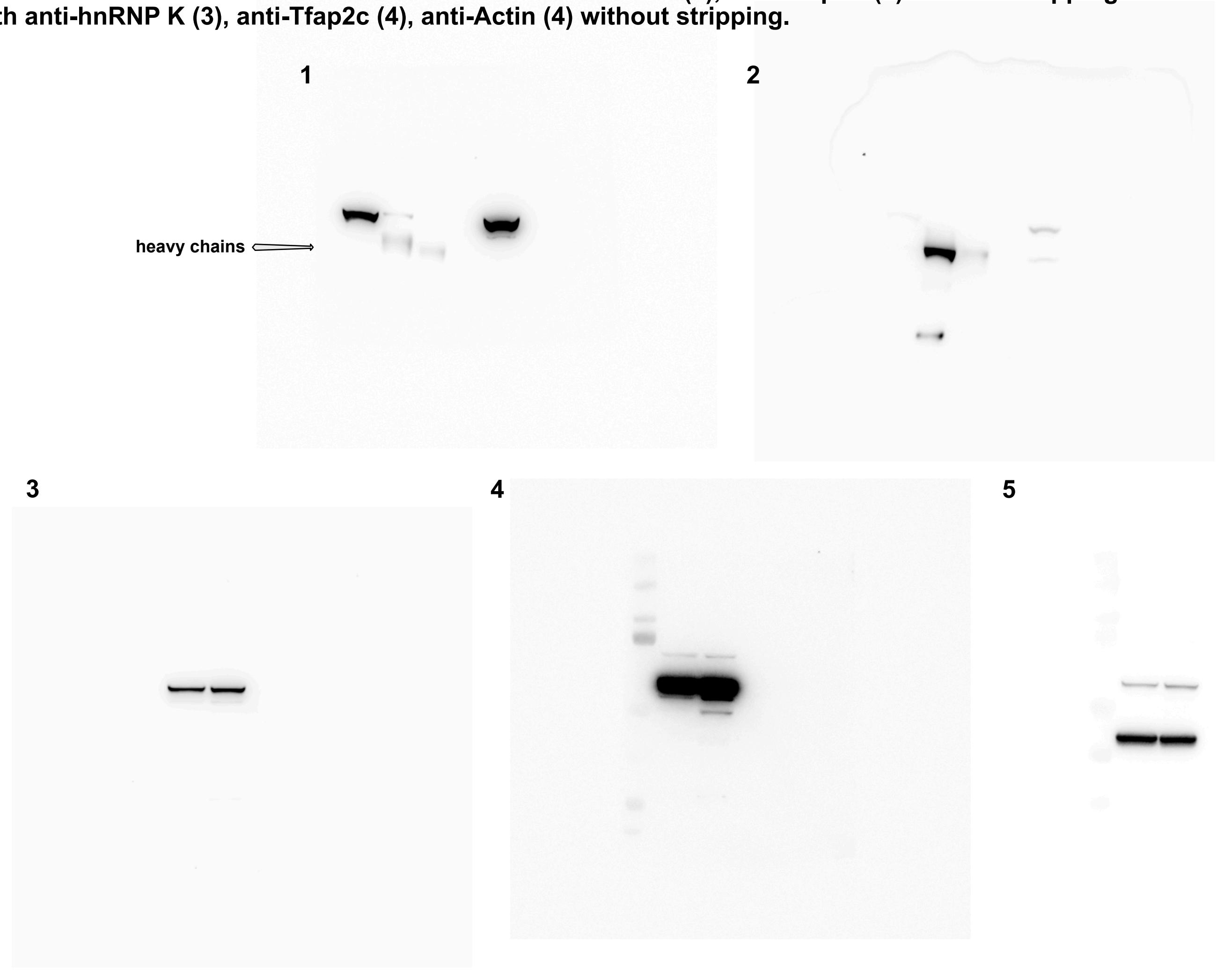

S4E Fig:

The Co-IP membrane was incubated with anti-hnRNP K (1). After stripping the membrane was incubated with anti-Tfap2c (2). The FT membrane was incubated in succession with anti-hnRNP K (3), anti-Actin (4) and anti-Tfap2c (5) without stripping.

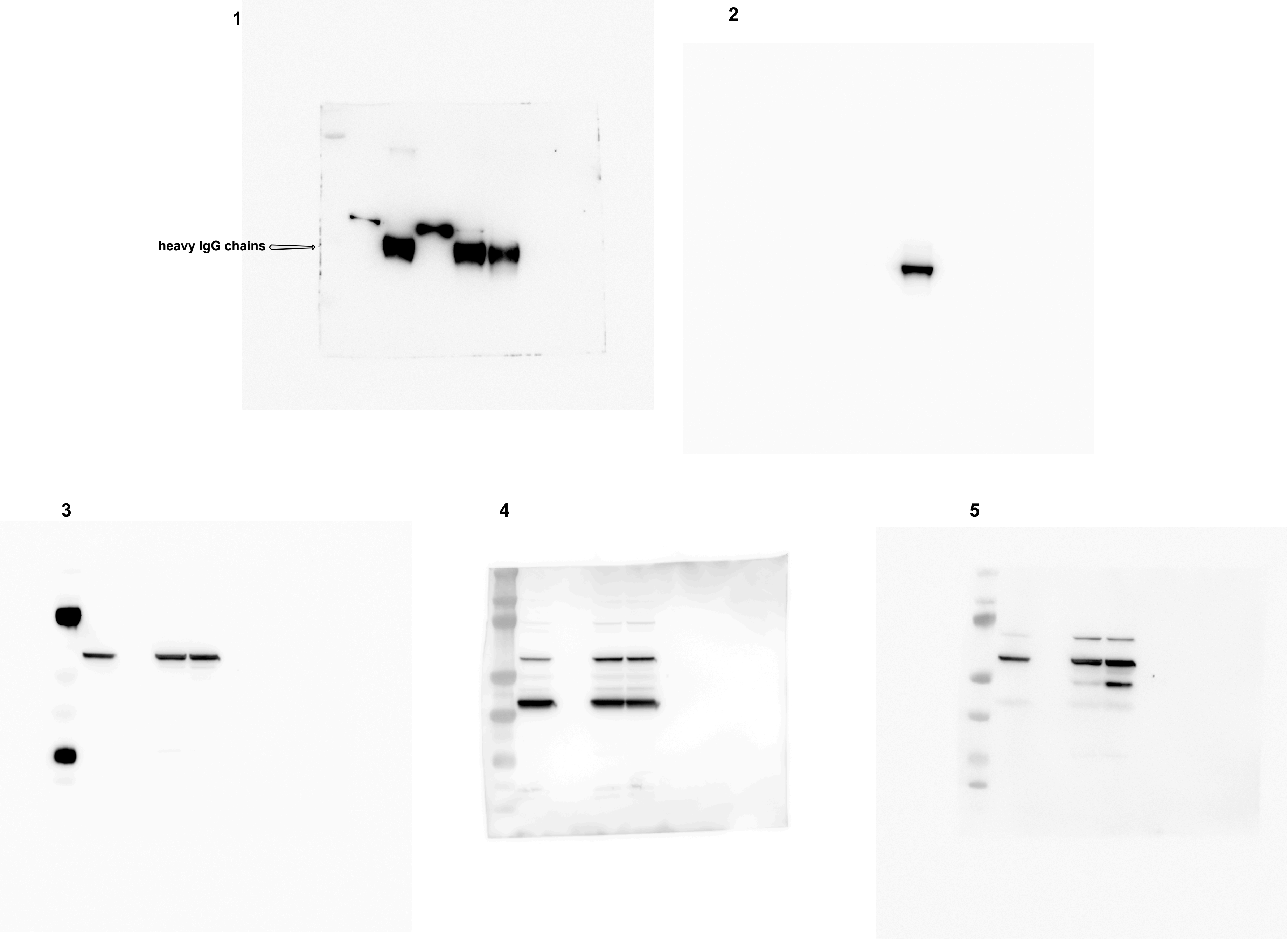

S4F (left) and S4G (right) Fig:

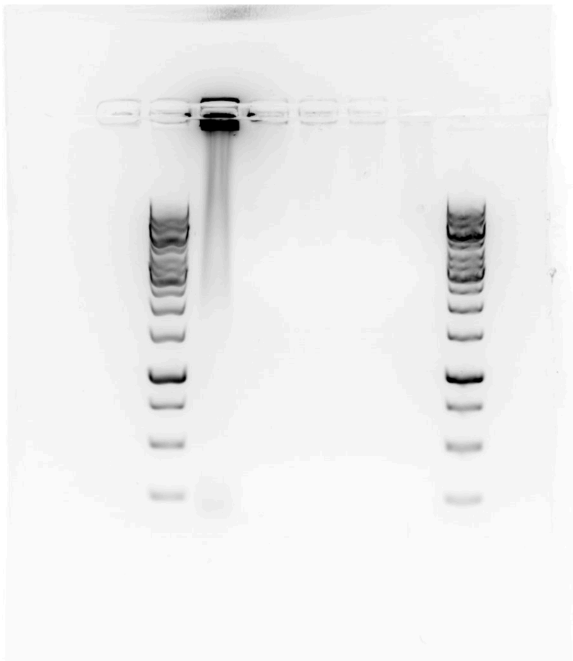

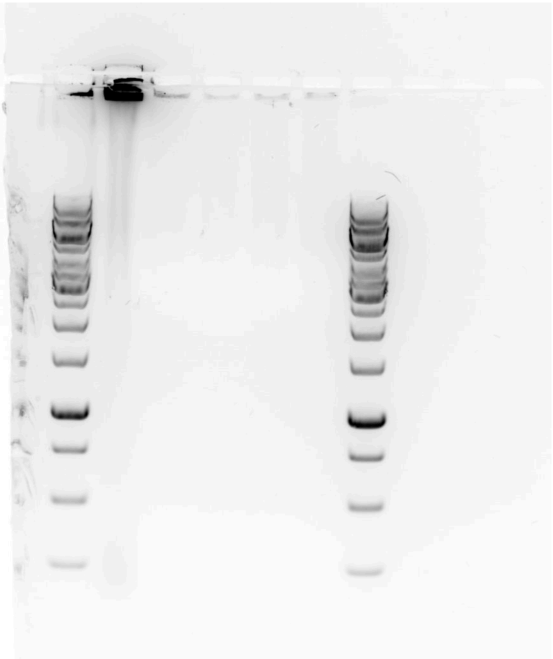

S5A Fig:

The membrane was incubated in succession with anti-GPX4 (1), anti-Actin (2), anti-Tfap2c (3), anti-hnRNP K (4) without stripping.

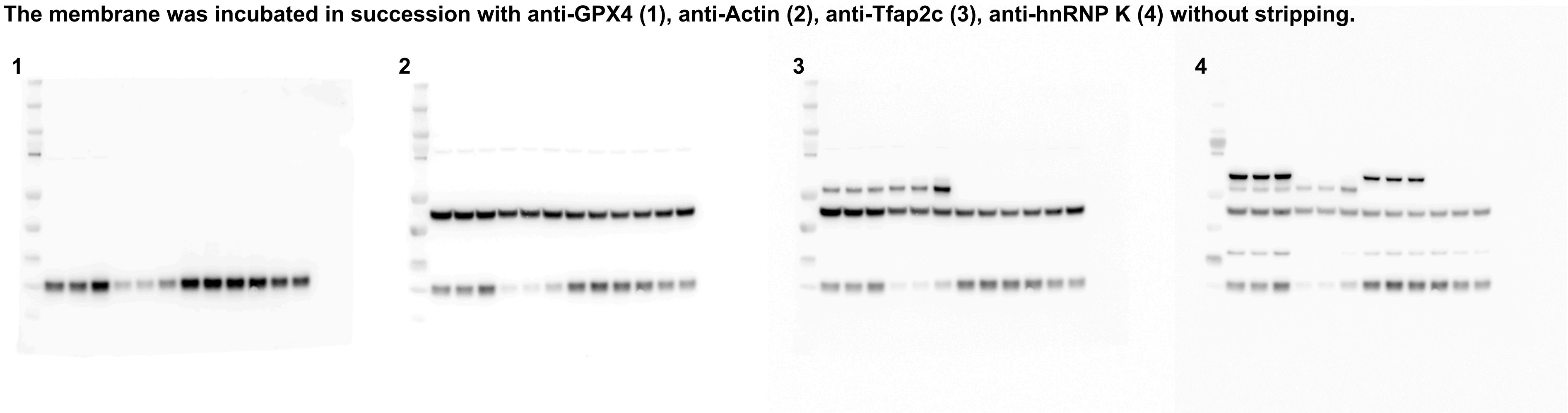

S5A Fig (extra membranes for quantification):

The membrane was incubated with anti-GPX4 (1). After stripping the membrane was incubated in succession with anti-Tfap2c (2), anti-hnRNP K (3), anti-Actin (4).

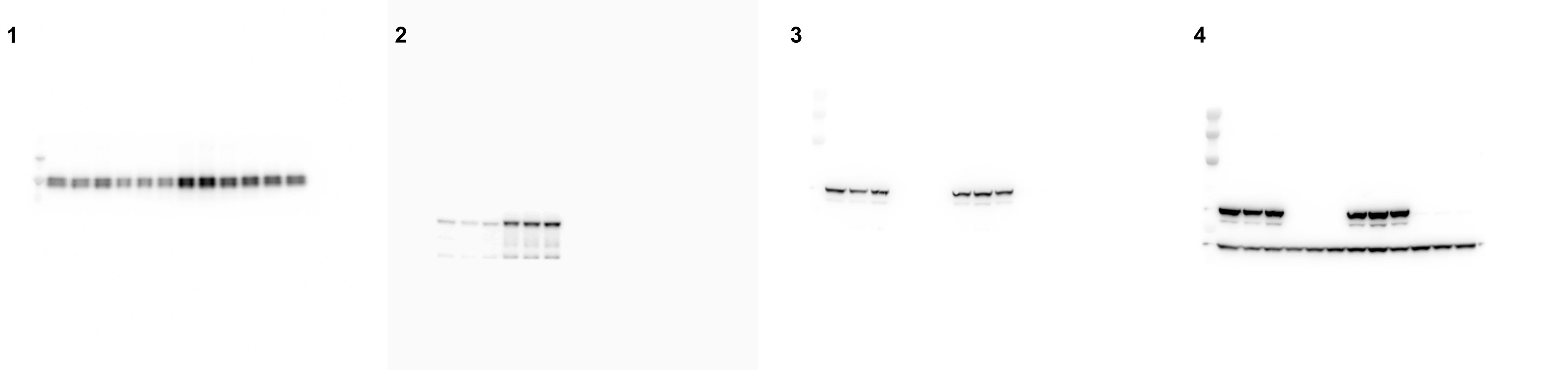

S5A Fig (extra membranes for quantification):

The membrane was incubated with anti-GPX4 (1). After stripping the membrane was incubated in succession with anti-Tfap2c (2), anti-hnRNP K (3). After another stripping, the membrane was incubated with anti-Actin (4)

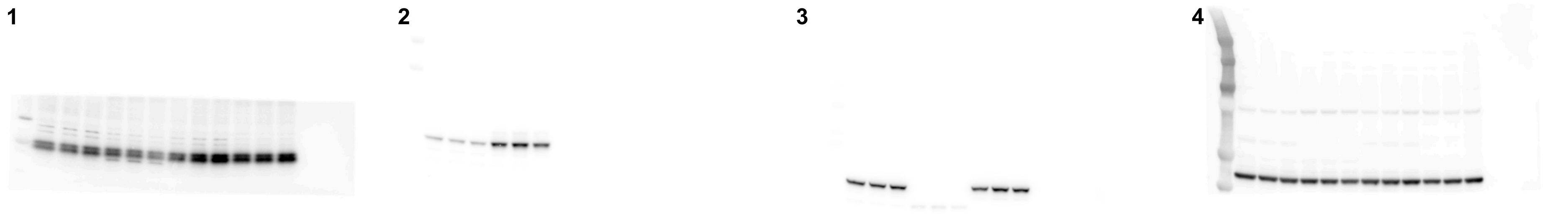

S5B Fig:

The membrane was incubated in succession with anti-GPX4 (1), anti-Tfap2c (2), anti-hnRNP K (3), anti-Actin (4) without stripping.

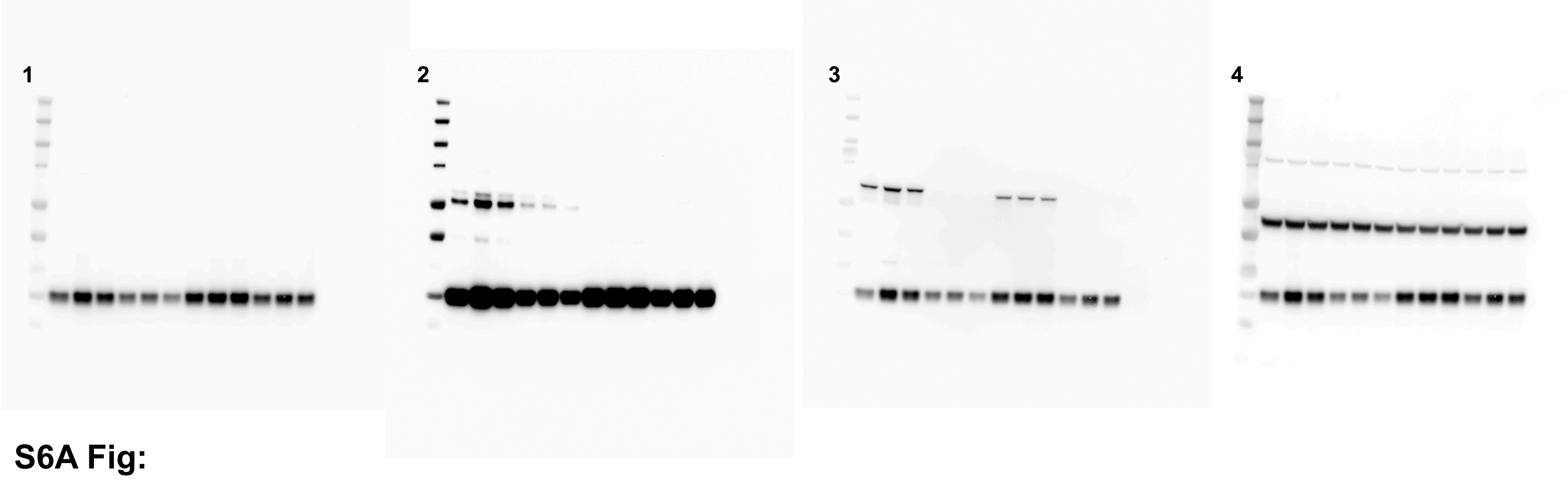

S6A Fig:

The membrane was incubated in succession with anti-hnRNP K (1), anti-Tfap2c (2) without stripping. After stripping the membrane was incubated with anti-Actin (3).

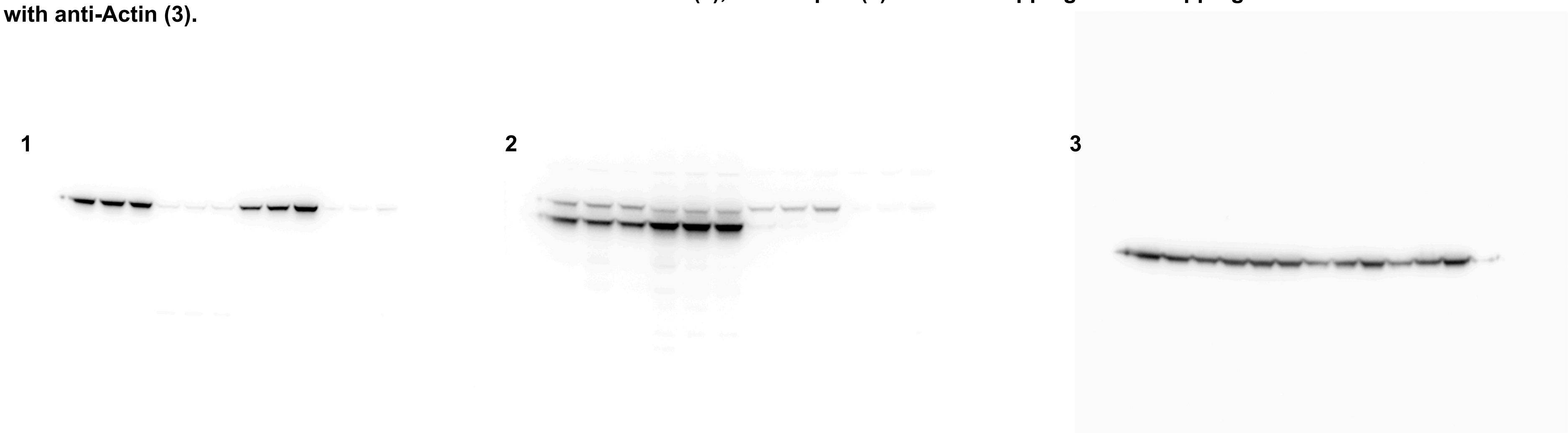

S6B Fig (top):

The membrane was incubated in succession with anti-pAMPK (1), anti-Tfap2c (2), anti-hnRNP K (3), anti-Actin (4) without stripping. After stripping the membrane was incubated with anti-AMPK (5). The last two lane of the gel were loaded with unrelated samples.

1

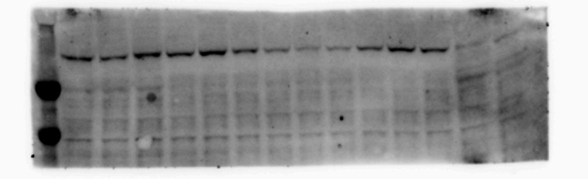

2

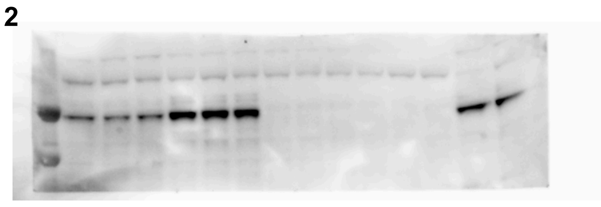

3

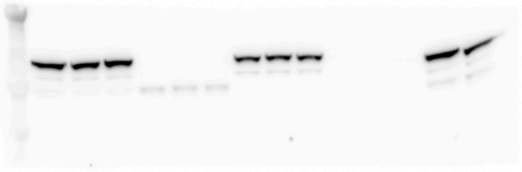

4

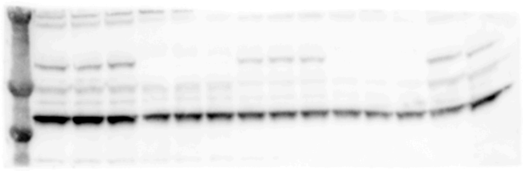

5

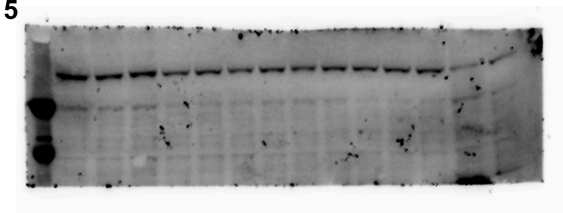

S6B Fig (bottom):

The membrane was incubated with anti-pAMPK (1). After stripping the membrane was incubated with anti-AMPK (2). After stripping the membrane was incubated in succession with anti-Tfap2c (3) and anti-hnRNP K (4) without stripping. After stripping the membrane was incubated with anti-Actin (5).

1

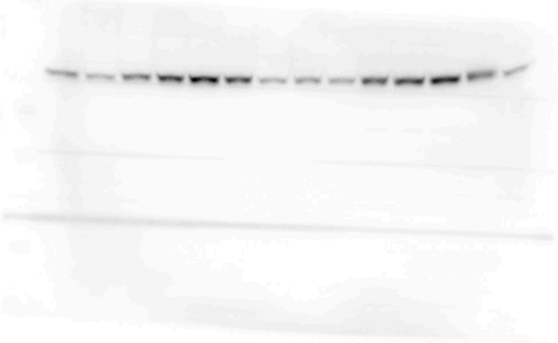

2

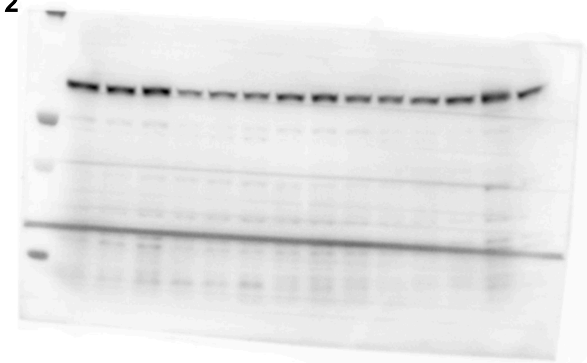

3

4

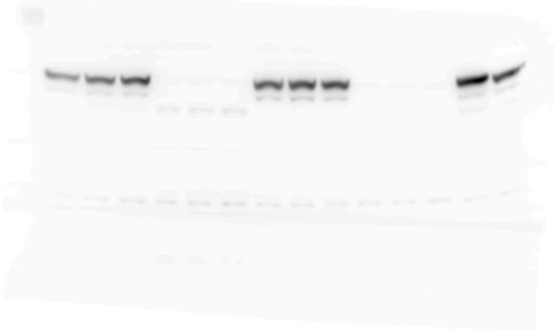

5

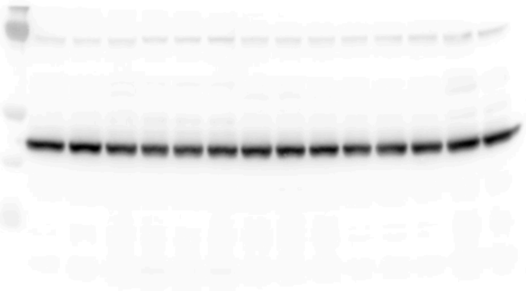

S6C Fig:

The membrane was cut between 22-28 kDa (SeeBlue Ladder in MOPS) (1 and 4). Membrane 1 was incubated in succession with anti-TFAP2C K (1), anti-hnRNP K (2) and after stripping with anti-Actin (3). Membrane 4 was incubated with anti-LC3B (4). Shown are two membranes loaded with the same samples, but subjected to different protein transfer strategies. The final figure was generated using the top membrane as the bottom membrane showed issues with protein loading or actin detection.

1

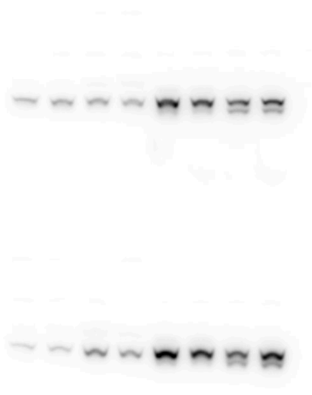

2

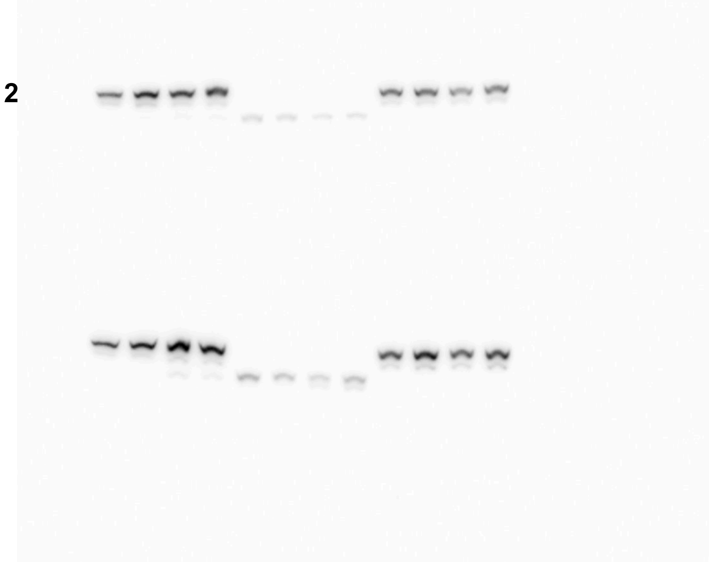

3

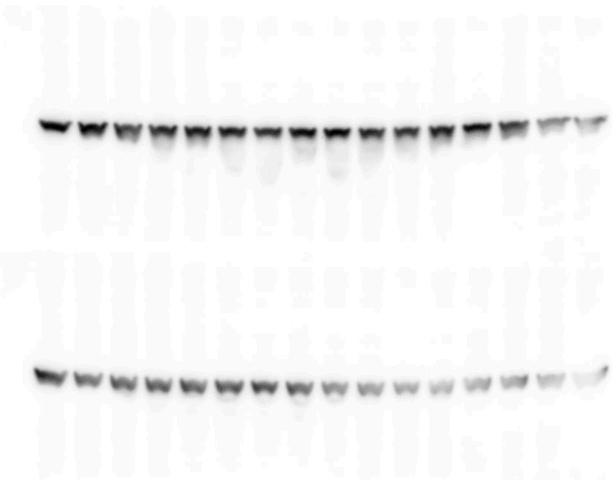

4

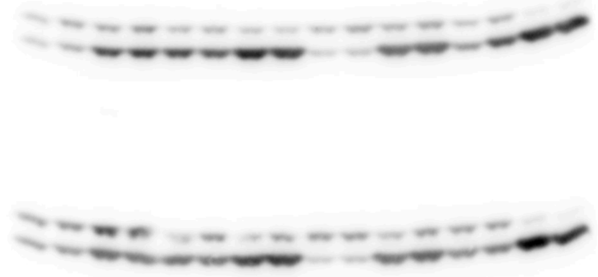

S6D Fig:

The membrane was cut just above 75 kDa and below 37 kDa (1, 2 and 5). Membrane 1 (10-37 kDa) was incubated with anti-4EBP1 (not shown). Membrane 2 (37-75 kDa) was incubated in succession with anti-hnRNP K (2), anti-Tfap2c (3) without stripping (for Tfap2c acquisition the membrane was covered with alluminium foil to mask the too intense signal coming from hnRNP K). After stripping membrane 2 was incubated with anti-Actin (4). Membrane 5 was incubated with anti-pUlk1 (5) and, after stripping, with anti-Ulk1 (6).

2

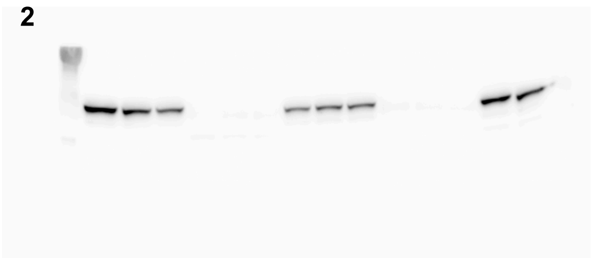

3

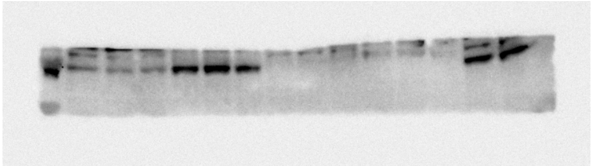

4

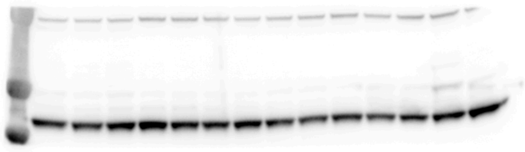

5

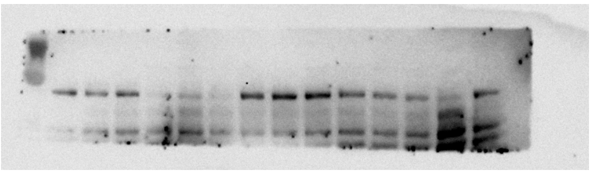

6

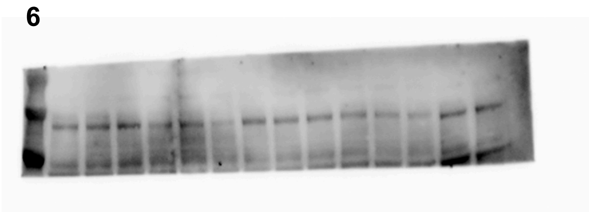

S7B Fig:

The membrane was cut at 75 kDa and membrane 1 (75-250 kDa) was incubated with anti-Vinculin (1), while membrane 2 (10-75 kDa) was incubated with anti-PrP (POM2) (2).

1

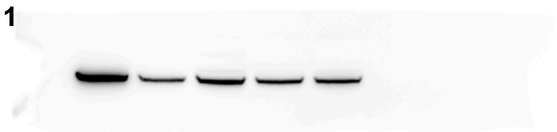

2

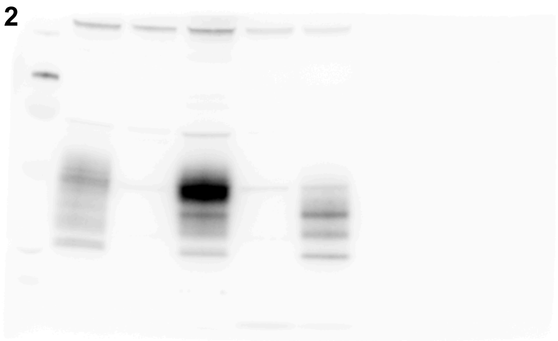

S7C Fig:

The membrane 1-2 was cut at 75 kDa. Membrane 1 (75-250 kDa) was incubated with anti-Vinculin (1), while membrane 2 (10-75 kDa) was incubated with anti-PrP (POM2) (2). The membrane 3 was transferred with the same samples pre-treated with PK digestion. Membrane 3 was incubated with anti-PrP (POM1) (3).

1

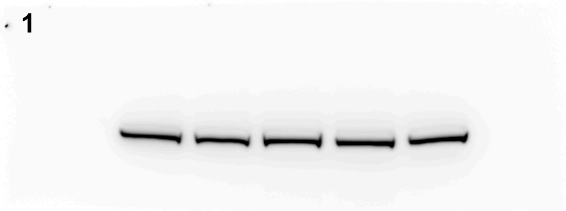

2

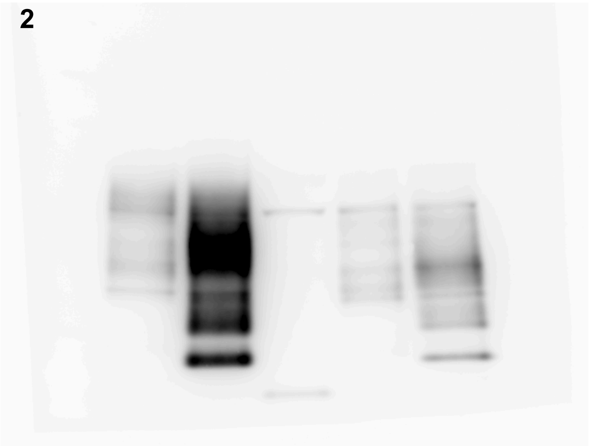

3

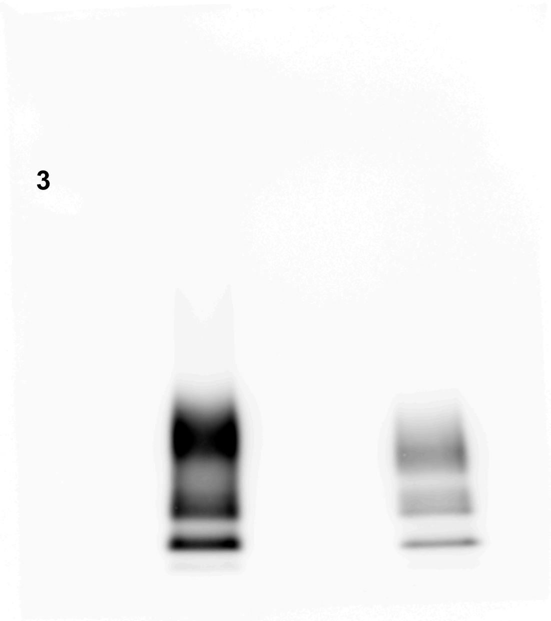

S8A Fig:

The membrane was cut at 75 kDa (1 and 2). Membrane 1 (75-250 kDa) was incubated with anti-Vinculin (1). Membrane 2 (10-75 kDa) was incubated anti-PrP (POM2) (2). Membrane 2 was following cut at 45 kDa (3 and 4). Membrane 3 (45-75 kDa) was incubated with both anti-hnRNP K and anti-Tfap2c (3). Membrane 4 was stripped and then incubated with anti-Actin first (not shown in the main figures) and then with anti-mCherry (4). Membrane 5 was transferred with the same samples of membrane 1-4 pre-treated with PK digestion. Membarne 5 was incubated with anti-PrP (POM1) (5).

1

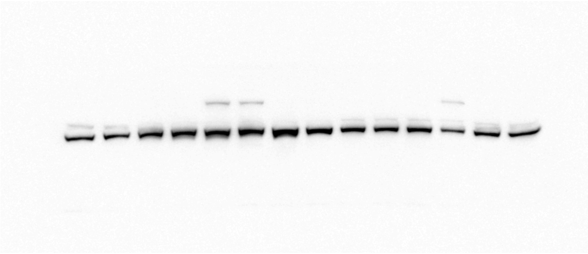

2

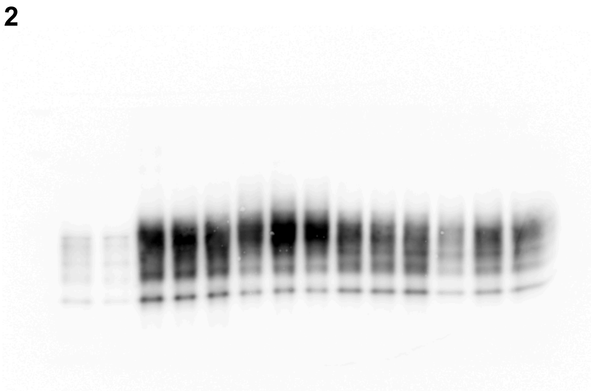

3

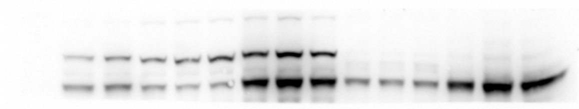

4

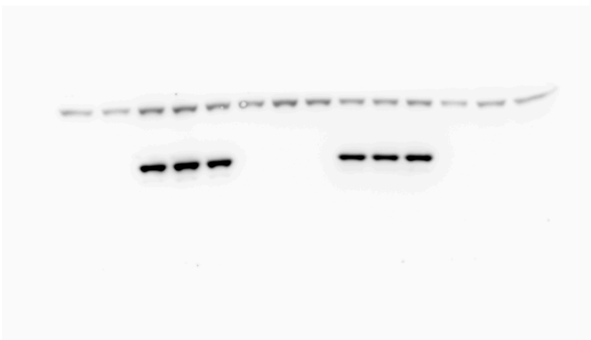

5

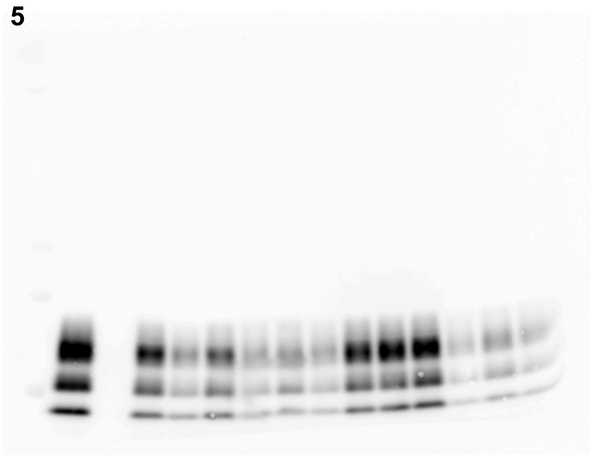

S8D Fig:

The membrane was cut below 37 kDa (1 and 2). Membrane 1 (35-250 kDa) was incubated with anti-Tfap2c and anti-hnRNP K (1). Membrane 2 (10-35 kDa) was incubated anti-mCherry (2). Membrane 1 was following stripped and incubated with anti-Actin (3). Shown are three experimental repeats. Only the first row of images was used as the representative figure in the main text.

1

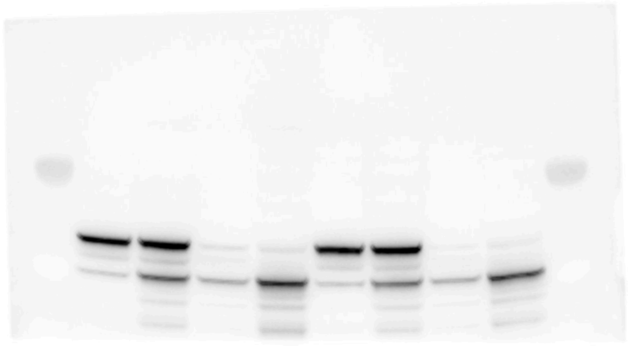

2

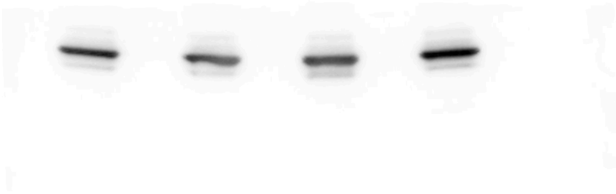

3

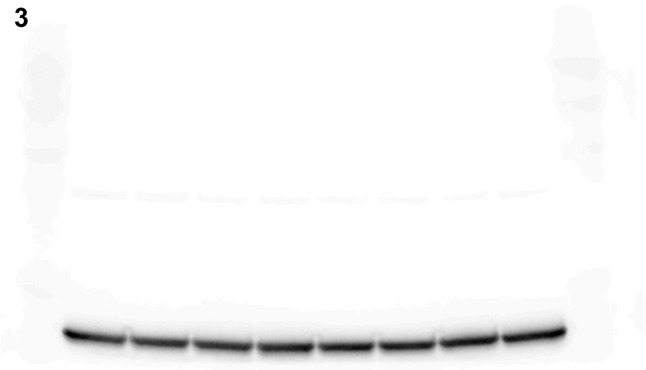

1

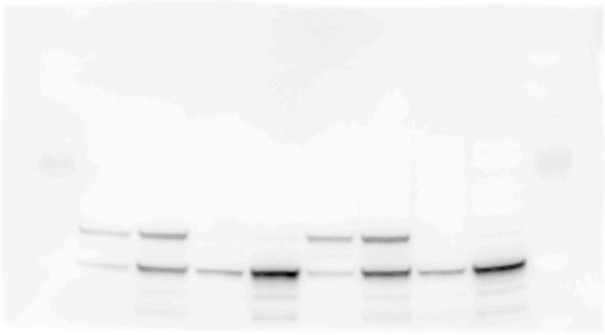

2

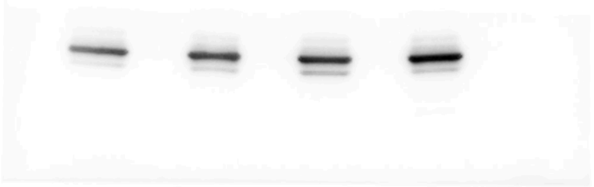

3

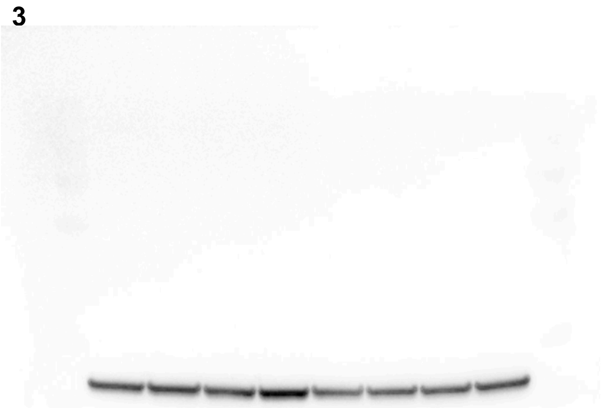

1

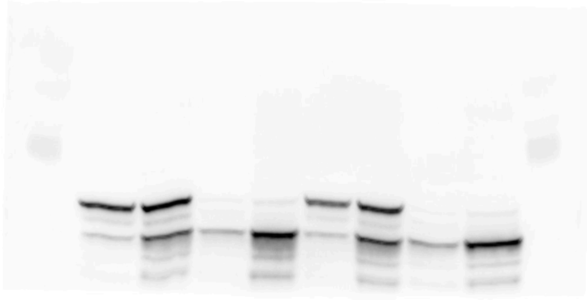

2

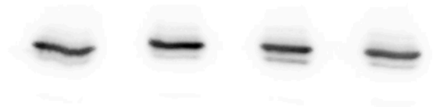

3

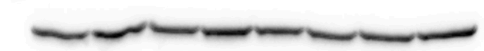

S9A Fig:

The membrane was cut between 250 and 150 kDa, at 75 kDa and below 50 kDa (1, 2, 3 and 4). Membrane 1 (190-350 kDa) was incubated with anti-mTOR. Membrane 2 (75-190 kDa) was incubated anti-Rptor. Membrane 3 (45-75 kDa) was incubated with anti-hnRNP K. Membrane 4 was incubated with anti-Actin.

1

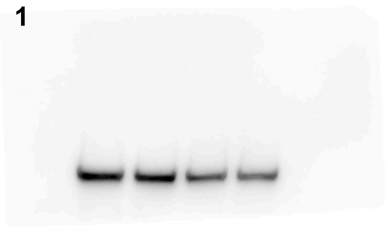

2

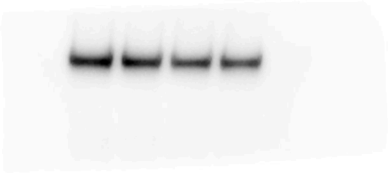

3

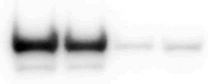

4

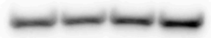

S9B Fig:

The membrane was incubated with anti-pS6 (1). After stripping the membrane was incubated with anti-S6 (2).

1

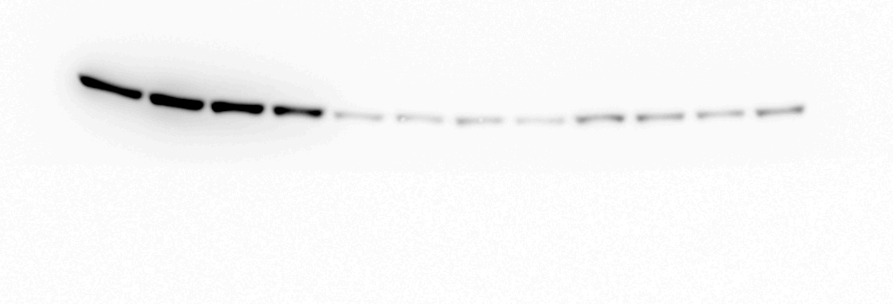

2

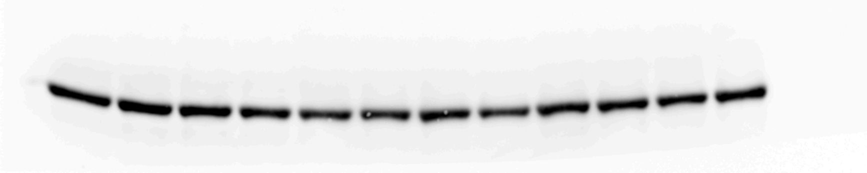

S9C Fig:

The membrane was cut between 250 and 150 kDa and at 75 kDa kDa (1, 2 and 3). Membrane 1 (180-300 kDa) was incubated with anti-mTor. Membrane 2 (75-190 kDa) was incubated with anti-Rptor. Membrane 3 was incubated with anti-PrP (POM2). After stripping, membrane 2 was incubated with anti-Vinculin (4). Membrane 5 was transferred with the same samples of membrane 1-4 pre-treated with PK digestion. Membarne 5 was incubated with anti-PrP (POM1).

1

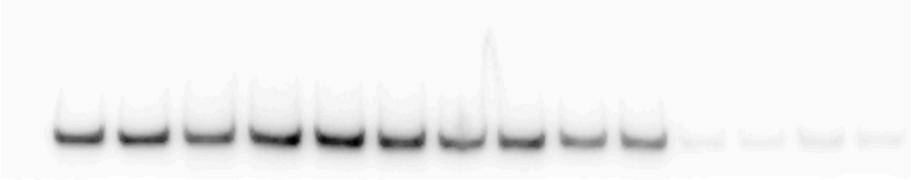

2

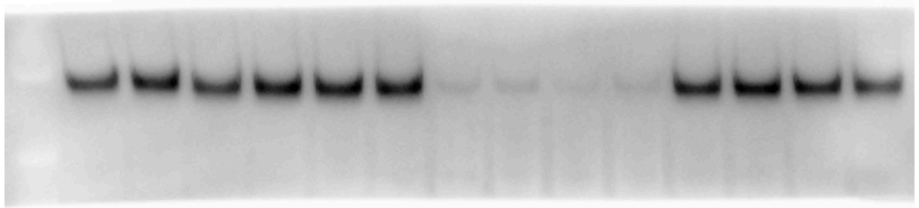

3

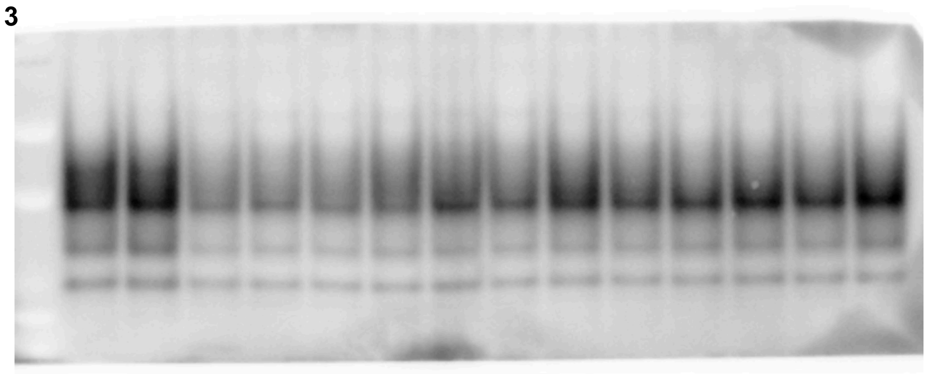

4

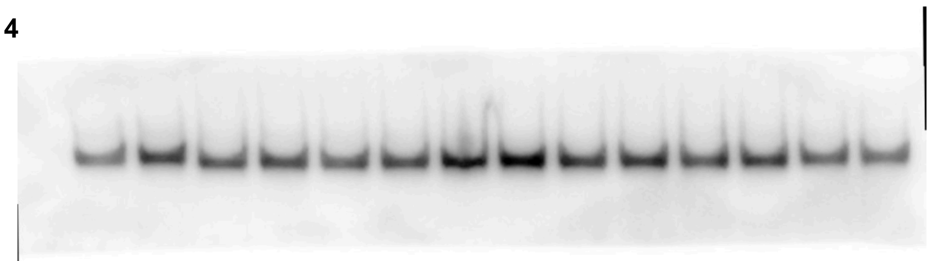

5

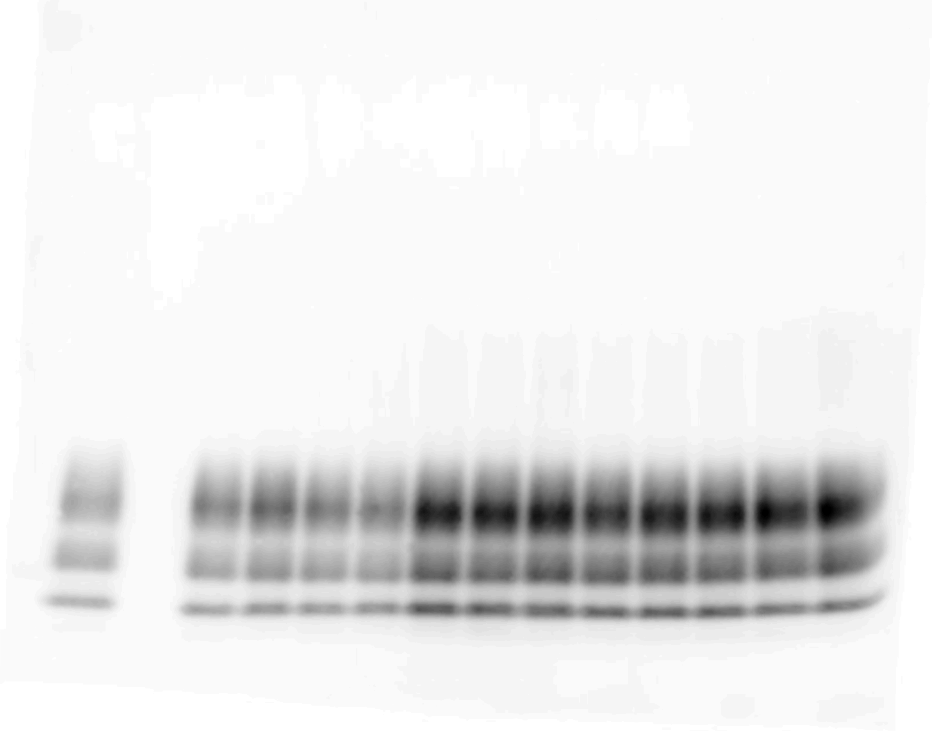

Supplement: S1 Appendix — (PDF) [file ppat.1014056.s015.pdf]
