# Supplementary material for: Prion propagation is controlled by a hierarchical network involving the nuclear Tfap2c and hnRNP K factors and the cytosolic mTORC1 complex
Source: PLoS Pathog. 2026 Apr 20;22(4):e1014056. doi: 10.1371/journal.ppat.1014056 (PMC13108870; doi:10.1371/journal.ppat.1014056)
Supplement: S2 Appendix — (PDF) [file ppat.1014056.s016.pdf]

Fig 6D (left)

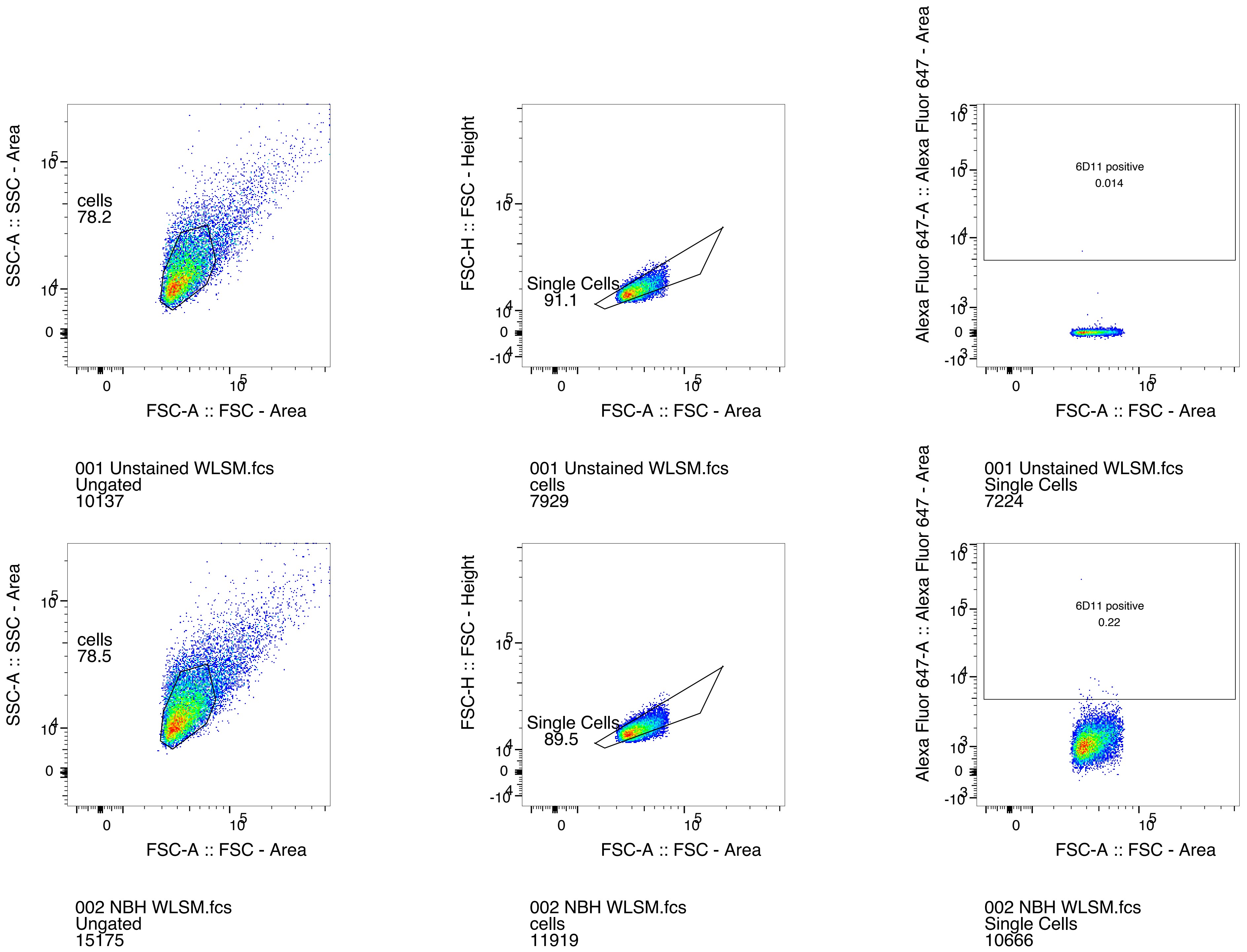

SSC-A :: SSC - Area
FSC-A :: FSC - Area
cells
78.2
001 Unstained WLSM.fcs
Ungated
10137
FSC-H :: FSC - Height
Single Cells
91.1
001 Unstained WLSM.fcs
cells
7929
Alexa Fluor 647-A :: Alexa Fluor 647 - Area
6D11 positive
0.014
001 Unstained WLSM.fcs
Single Cells
7224
cells
78.5
002 NBH WLSM.fcs
Ungated
15175
Single Cells
89.5
002 NBH WLSM.fcs
cells
11919
6D11 positive
0.22
002 NBH WLSM.fcs
Single Cells
10666

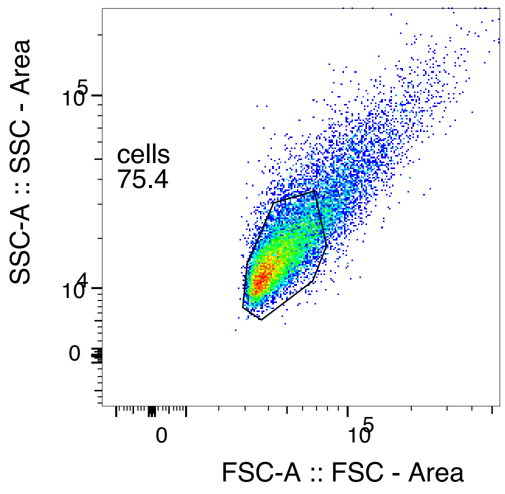

003 NTxTFAP_1 WLSM.fcs
Ungated
15207

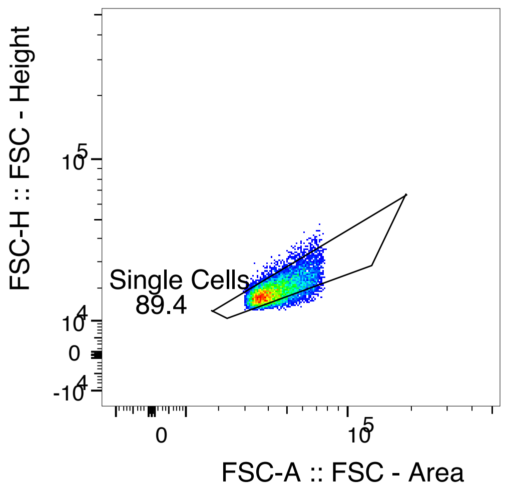

003 NTxTFAP_1 WLSM.fcs
cells
11466

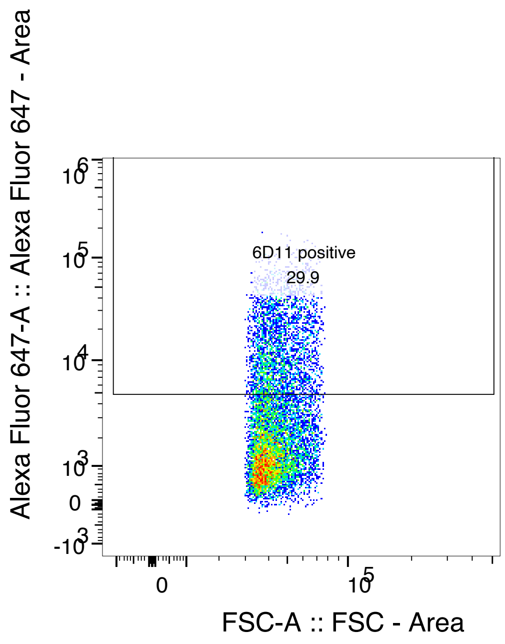

003 NTxTFAP_1 WLSM.fcs
Single Cells
10248

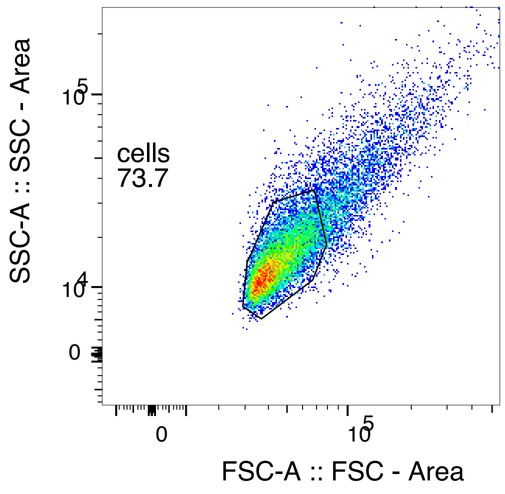

004 NTxTFAP_2 WLSM.fcs
Ungated
15282

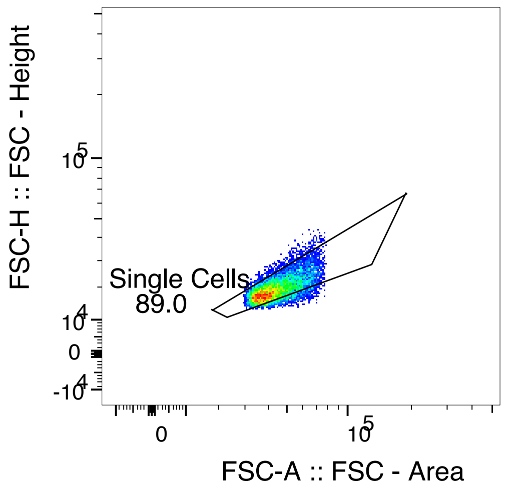

004 NTxTFAP_2 WLSM.fcs
cells
11264

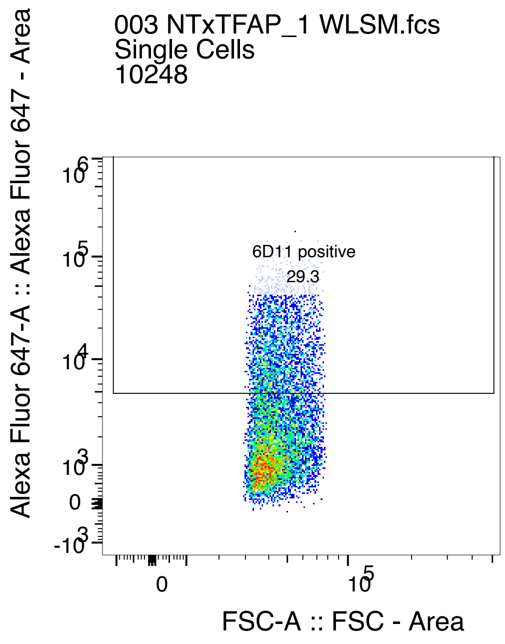

004 NTxTFAP_2 WLSM.fcs
Single Cells
10022

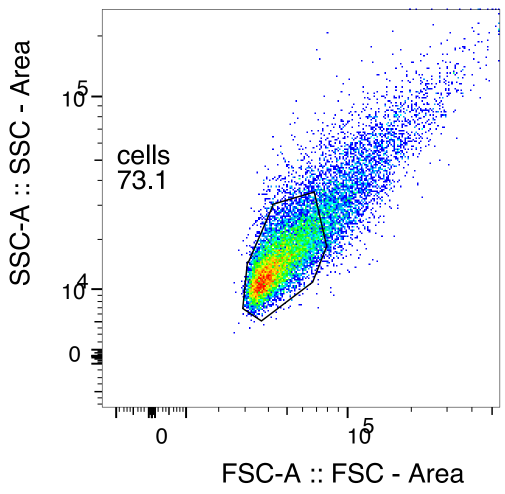

005 NTxTFAP_3 WLSM.fcs
Ungated
15255

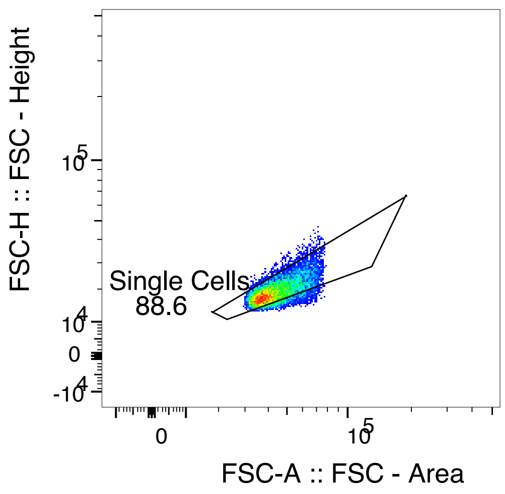

005 NTxTFAP_3 WLSM.fcs
cells
11157

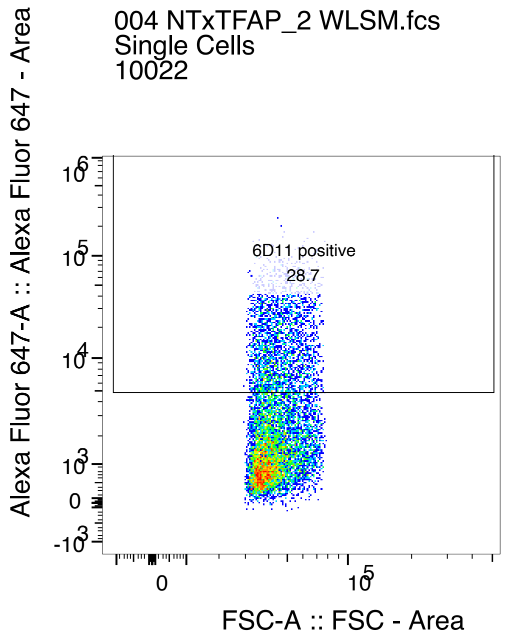

005 NTxTFAP_3 WLSM.fcs
Single Cells
9885

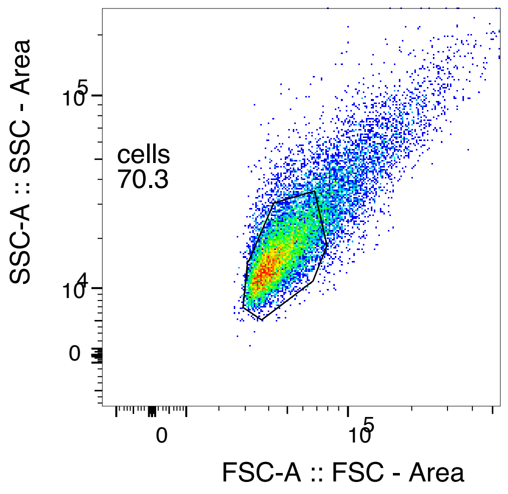

006 HxTFAP_1 WLSM.fcs
Ungated
15286

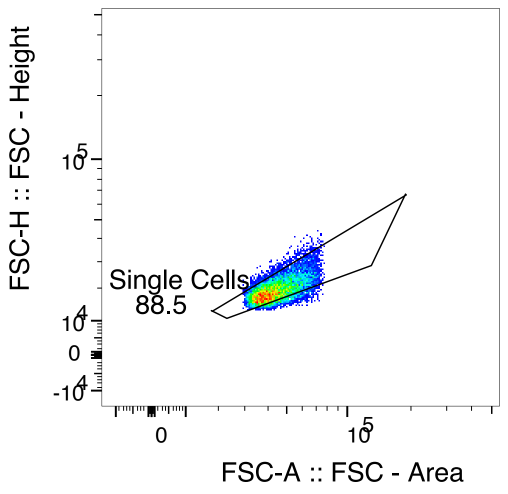

006 HxTFAP_1 WLSM.fcs
cells
10749

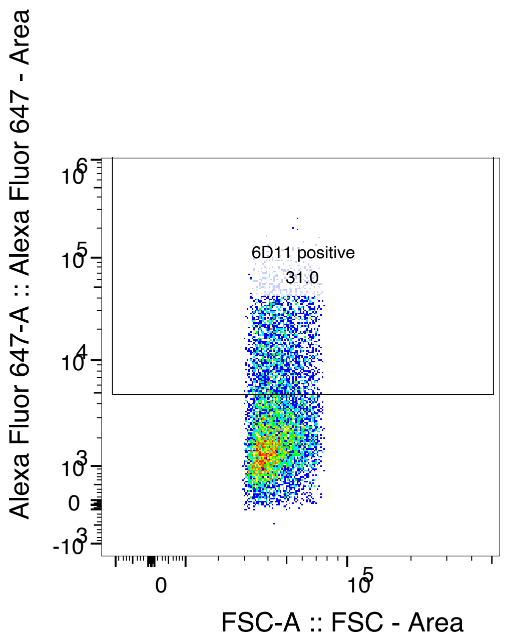

006 HxTFAP_1 WLSM.fcs
Single Cells
9511

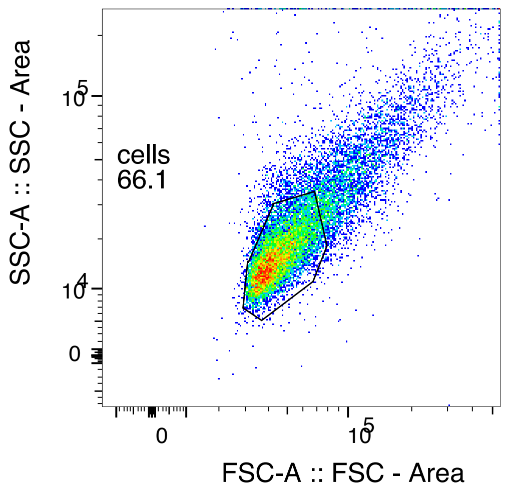

007 HxTFAP_2 WLSM.fcs
Ungated
15633

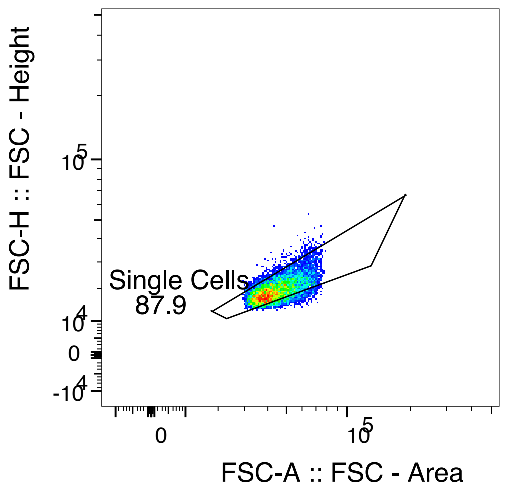

007 HxTFAP_2 WLSM.fcs
cells
10333

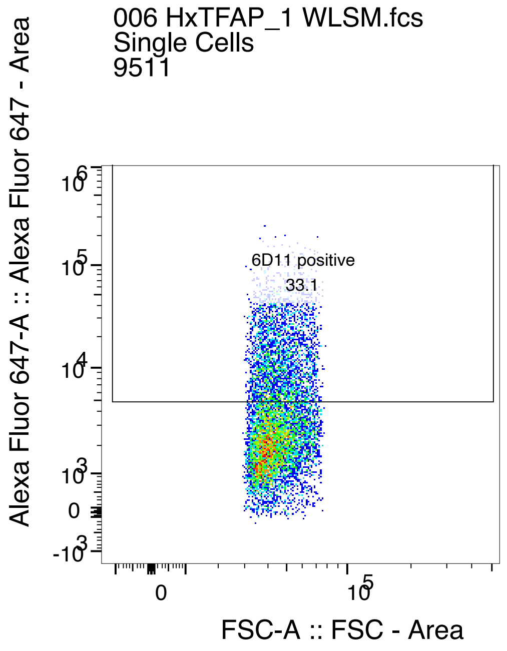

007 HxTFAP_2 WLSM.fcs
Single Cells
9083

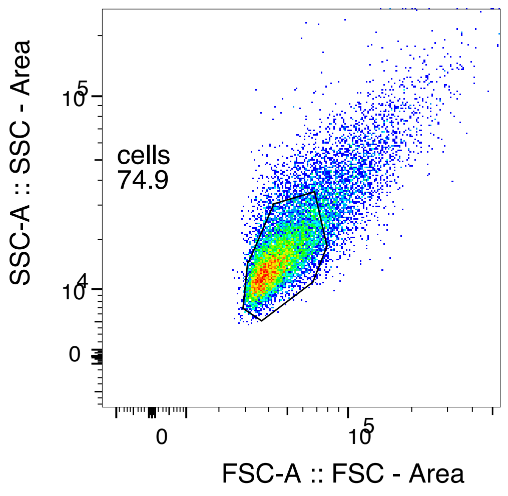

008 HxTFAP_3 WLSM.fcs
Ungated
15192

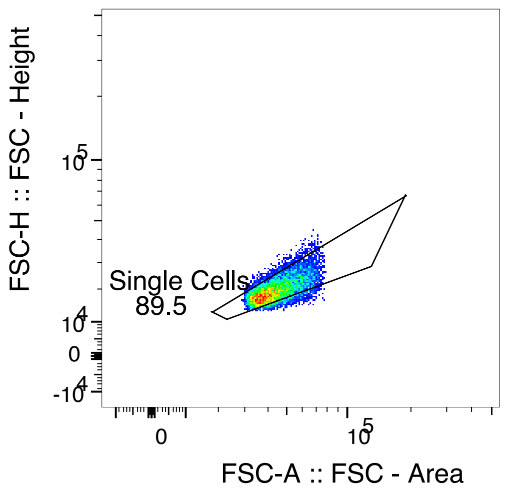

008 HxTFAP_3 WLSM.fcs
cells
11381

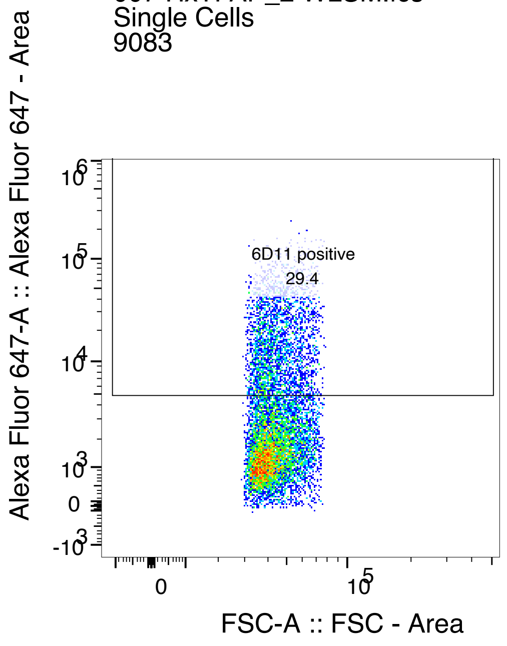

008 HxTFAP_3 WLSM.fcs
Single Cells
10187

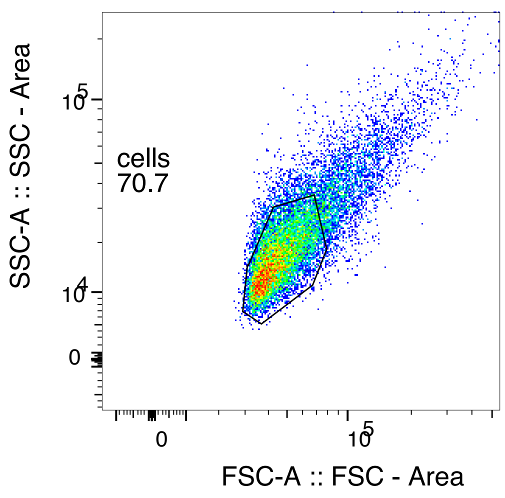

009 NTxmCherry_1 WLSM.fcs
Ungated
15198

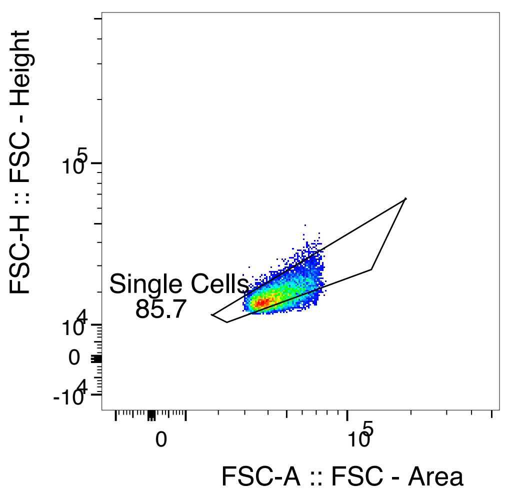

009 NTxmCherry_1 WLSM.fcs
cells
10751

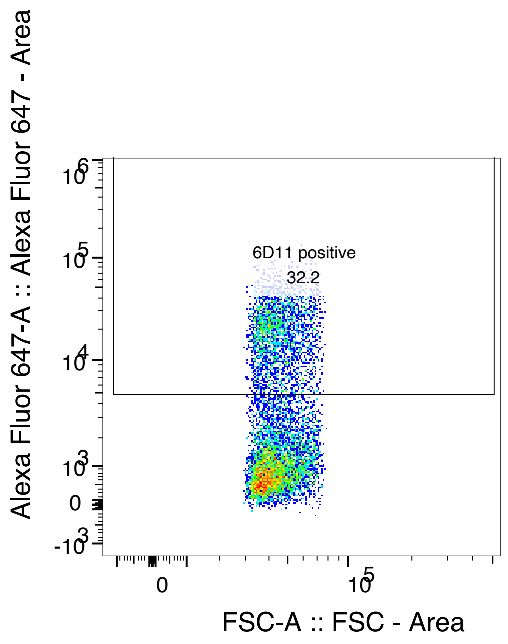

009 NTxmCherry_1 WLSM.fcs
Single Cells
9216

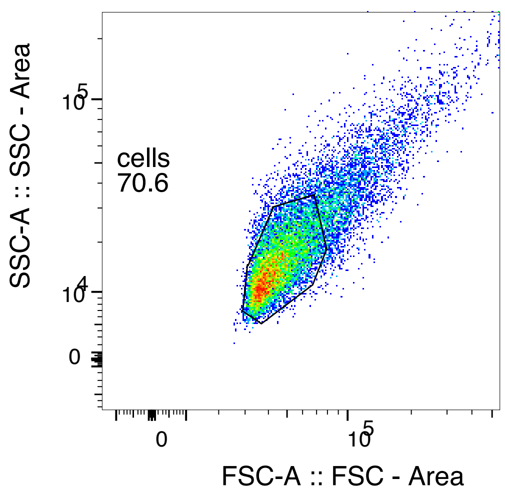

010 NTxmCherry_2 WLSM.fcs
Ungated
15309

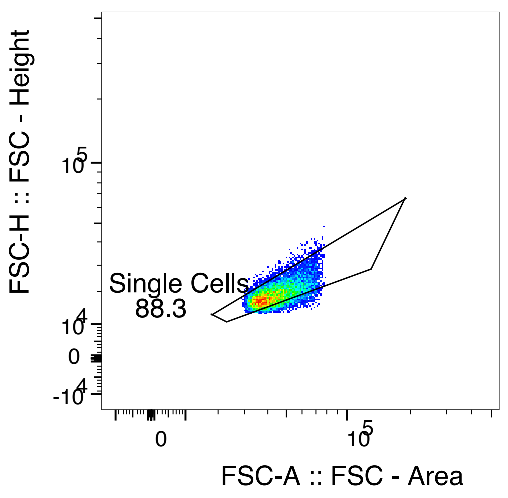

010 NTxmCherry_2 WLSM.fcs
cells
10801

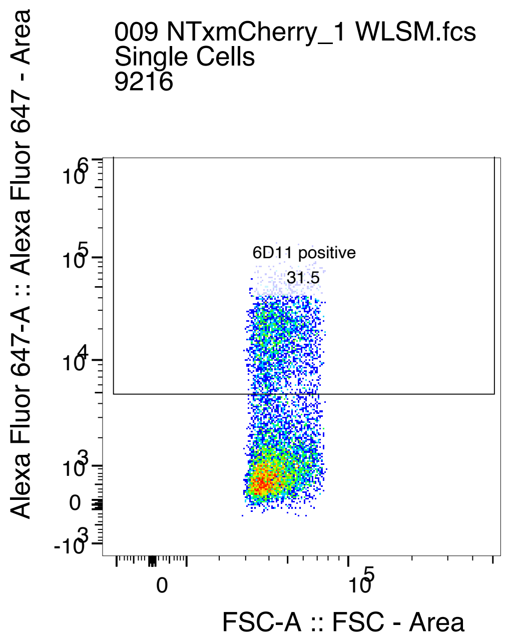

010 NTxmCherry_2 WLSM.fcs
Single Cells
9537

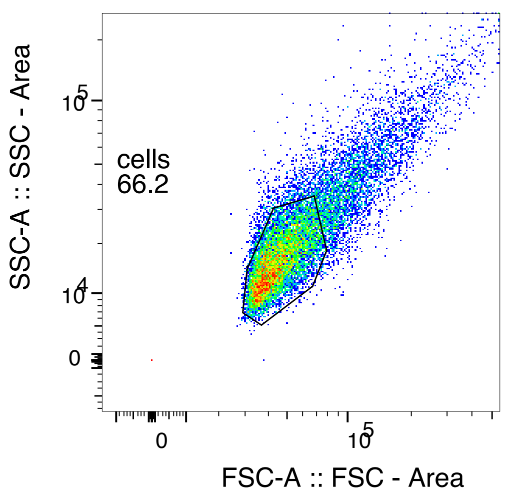

014 NTxmCherry_2 WLSM.fcs
Ungated
15379

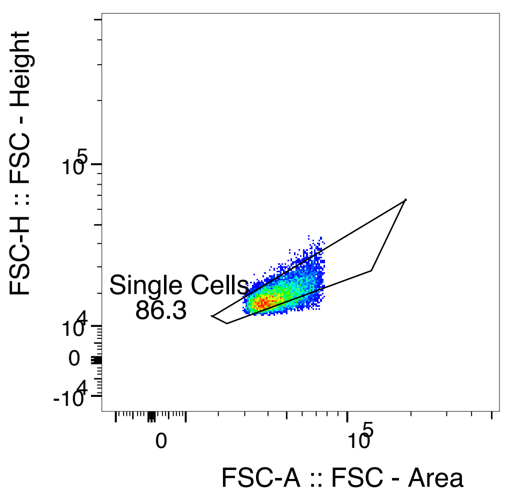

014 NTxmCherry_2 WLSM.fcs
cells
10176

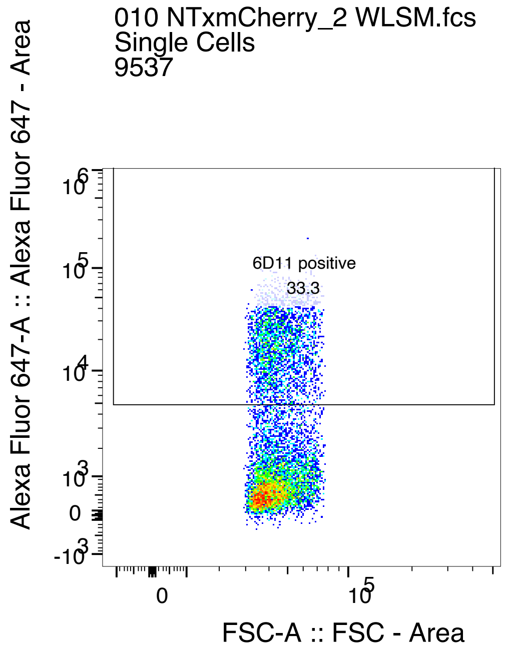

014 NTxmCherry_2 WLSM.fcs
Single Cells
8783

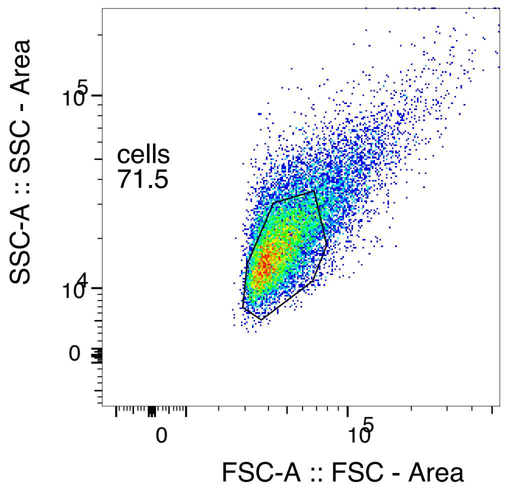

011 HxmCherry_1 WLSM.fcs
Ungated
15178

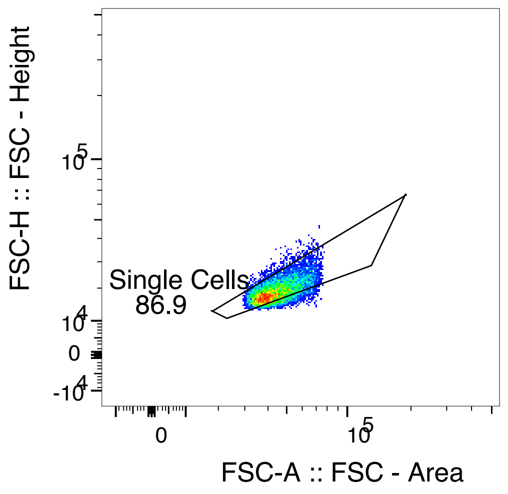

011 HxmCherry_1 WLSM.fcs
cells
10858

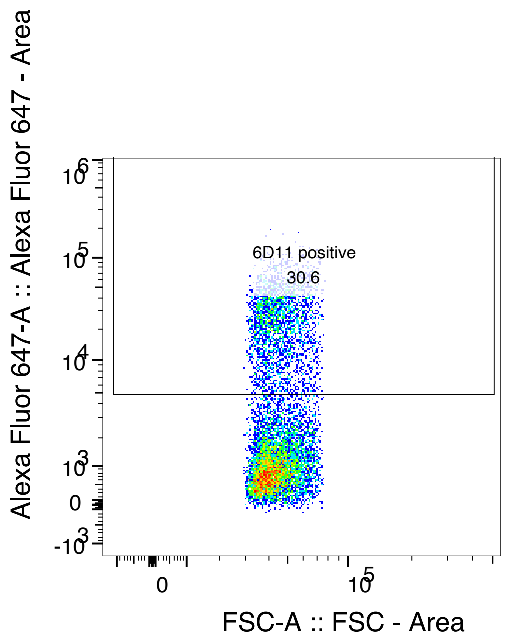

011 HxmCherry_1 WLSM.fcs
Single Cells
9435

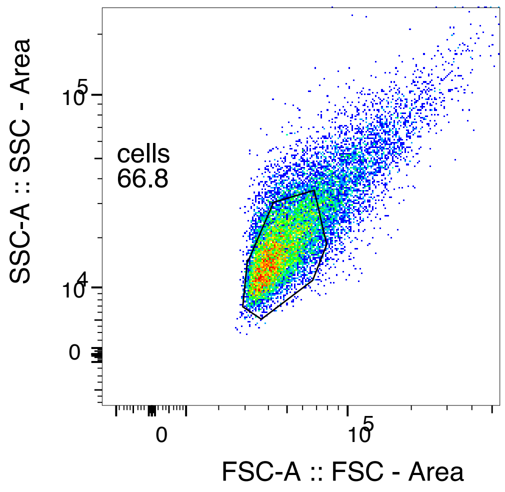

012 HxmCherry_2 WLSM.fcs
Ungated
15226

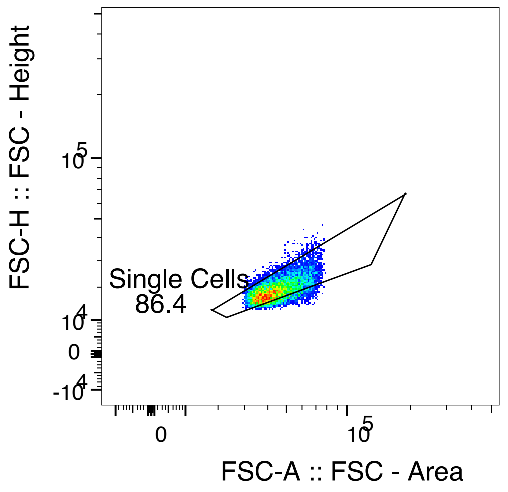

012 HxmCherry_2 WLSM.fcs
cells
10177

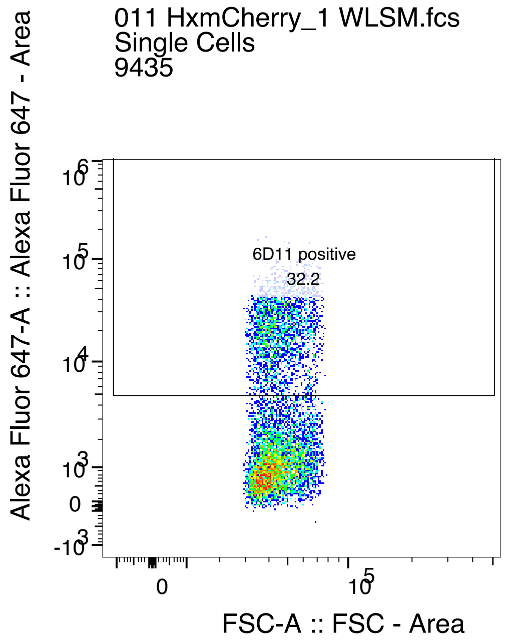

012 HxmCherry_2 WLSM.fcs
Single Cells
8788

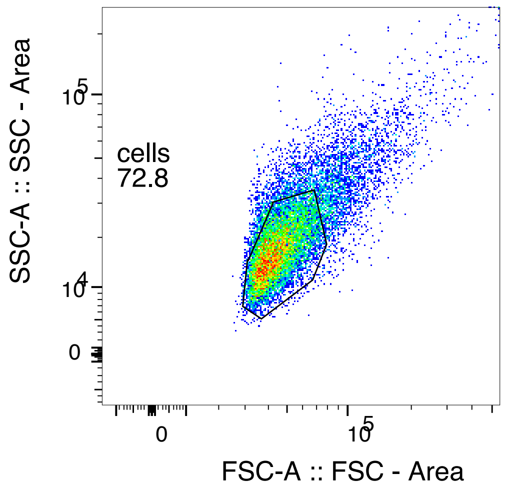

013 HxmCherry_3 WLSM.fcs
Ungated
15150

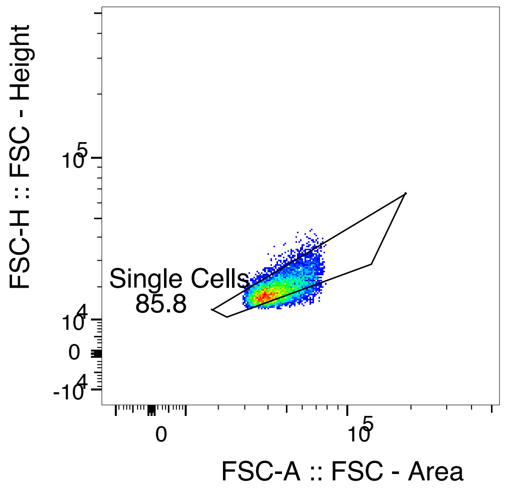

013 HxmCherry_3 WLSM.fcs
cells
11033

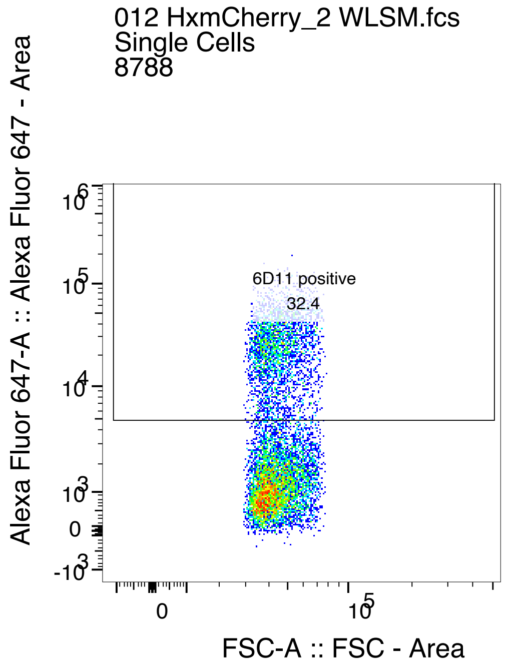

013 HxmCherry_3 WLSM.fcs
Single Cells
9465

Fig 6D (Right)

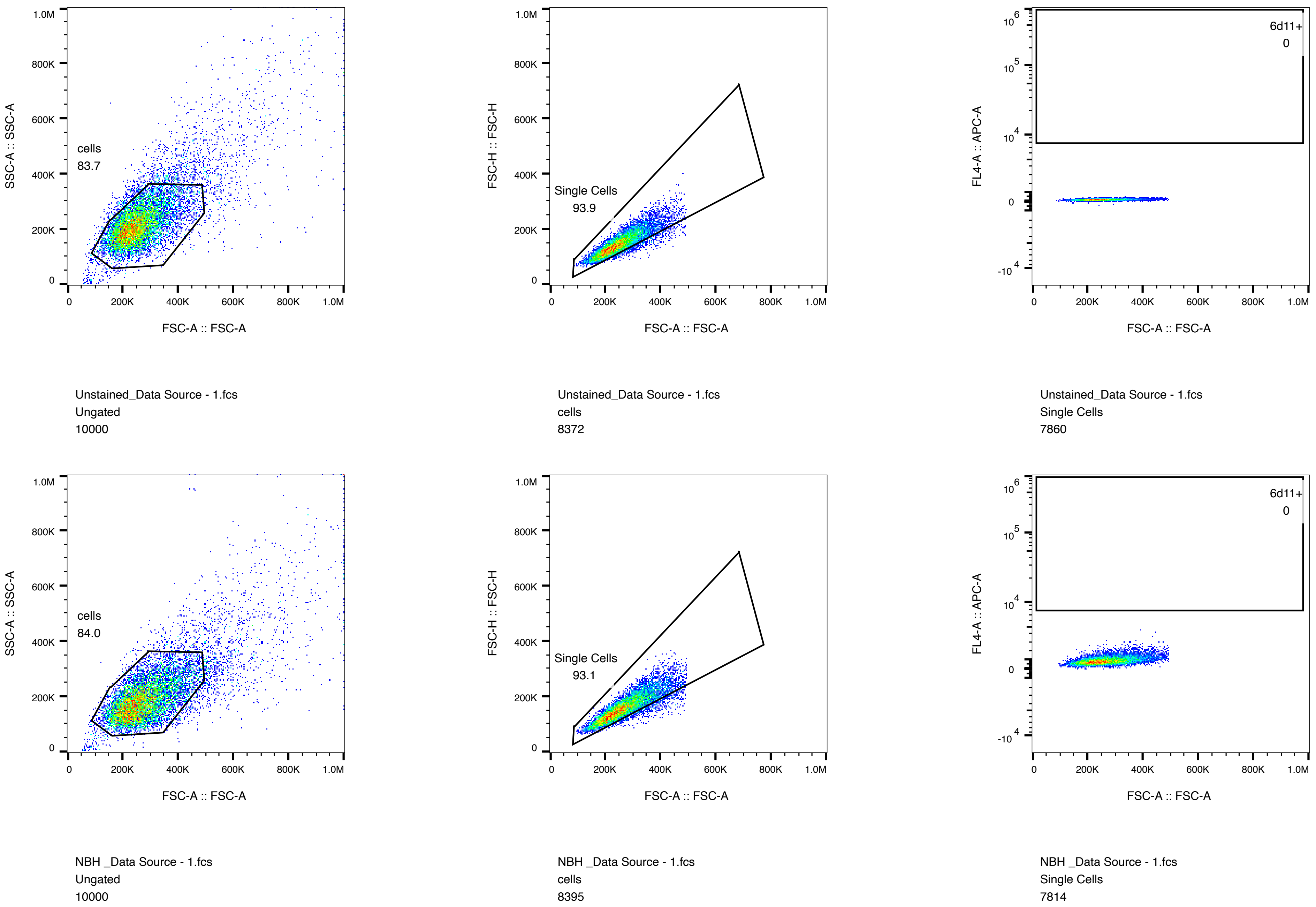

SSC-A :: SSC-A
FSC-A :: FSC-A
cells
83.7
Unstained_Data Source - 1.fcs
Ungated
10000
FSC-H :: FSC-H
FSC-A :: FSC-A
Single Cells
93.9
Unstained_Data Source - 1.fcs
cells
8372
FL4-A :: APC-A
FSC-A :: FSC-A
6d11+
0
Unstained_Data Source - 1.fcs
Single Cells
7860
SSC-A :: SSC-A
FSC-A :: FSC-A
cells
84.0
NBH _Data Source - 1.fcs
Ungated
10000
FSC-H :: FSC-H
FSC-A :: FSC-A
Single Cells
93.1
NBH _Data Source - 1.fcs
cells
8395
FL4-A :: APC-A
FSC-A :: FSC-A
6d11+
0
NBH _Data Source - 1.fcs
Single Cells
7814

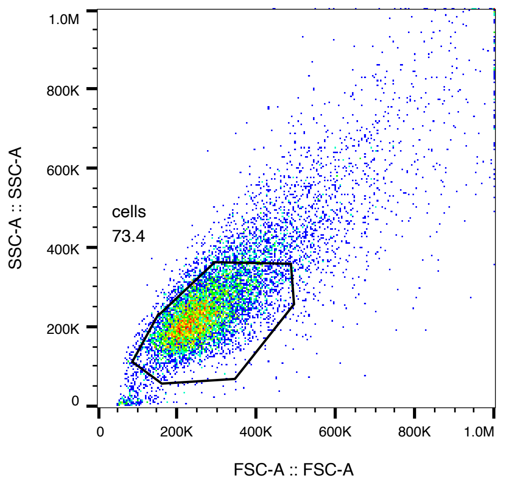

NTxmCherry_1_Data Source - 1.fcs
Ungated
10000

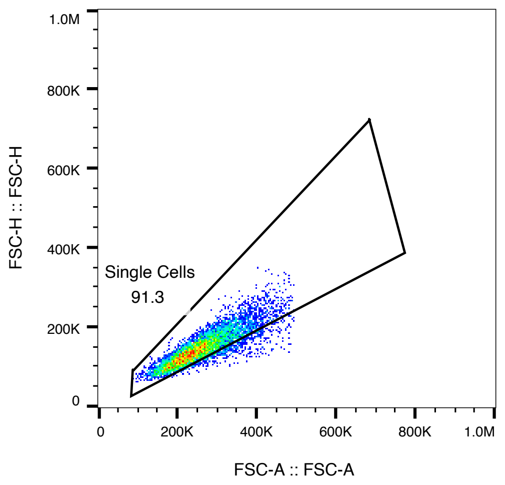

NTxmCherry_1_Data Source - 1.fcs
cells
7339

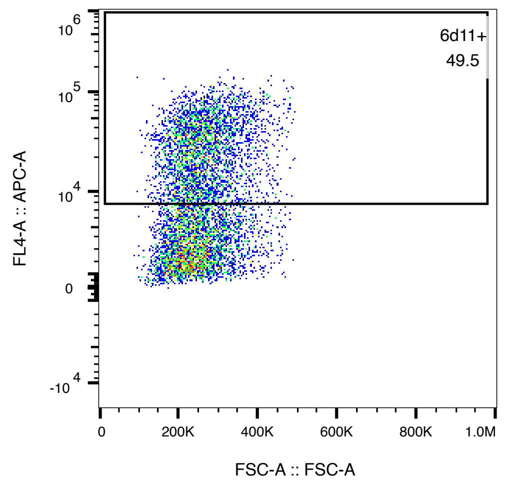

NTxmCherry_1_Data Source - 1.fcs
Single Cells
6704

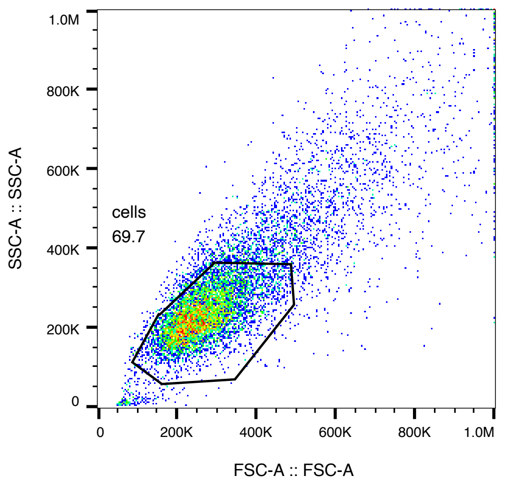

NTxmCherry_2_Data Source - 1.fcs
Ungated
10000

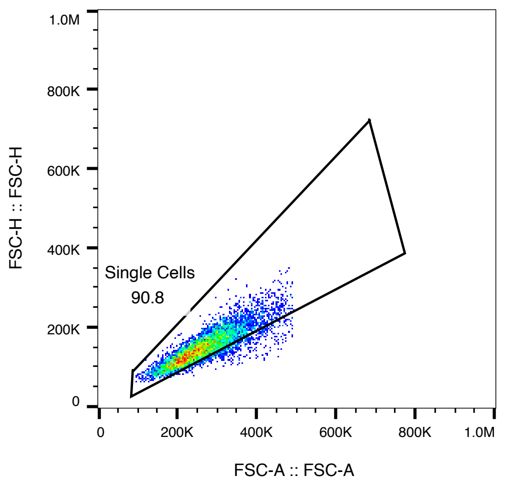

NTxmCherry_2_Data Source - 1.fcs
cells
6966

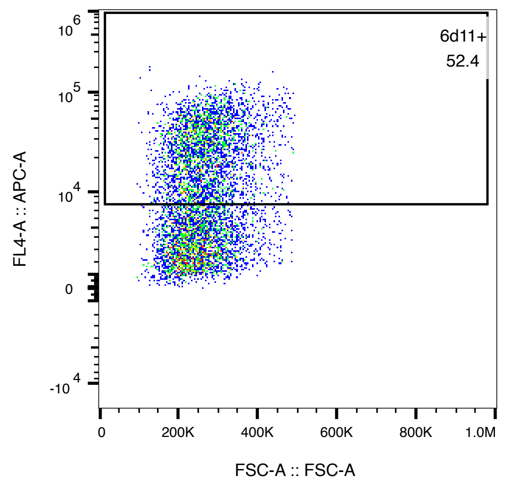

NTxmCherry_2_Data Source - 1.fcs
Single Cells
6322

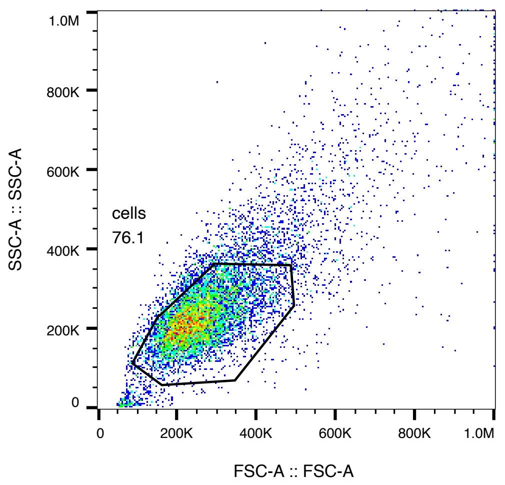

NTxmCherry_3_Data Source - 1.fcs
Ungated
10000

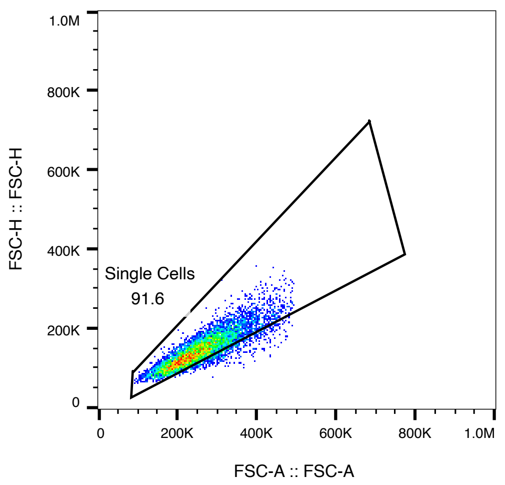

NTxmCherry_3_Data Source - 1.fcs
cells
7609

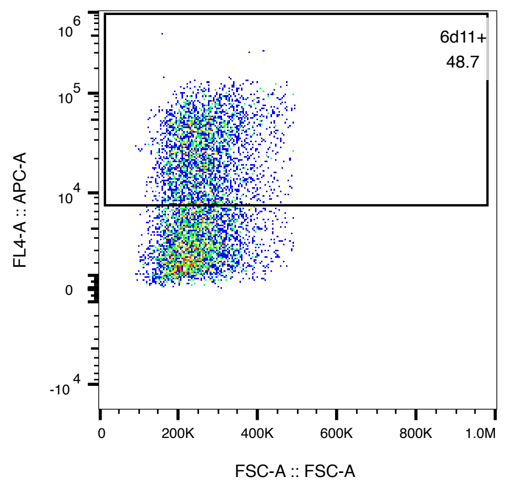

NTxmCherry_3_Data Source - 1.fcs
Single Cells
6970

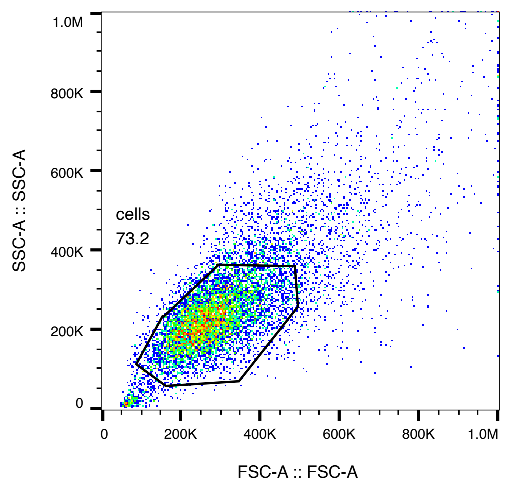

HxmCherry_1_Data Source - 1.fcs
Ungated
10000

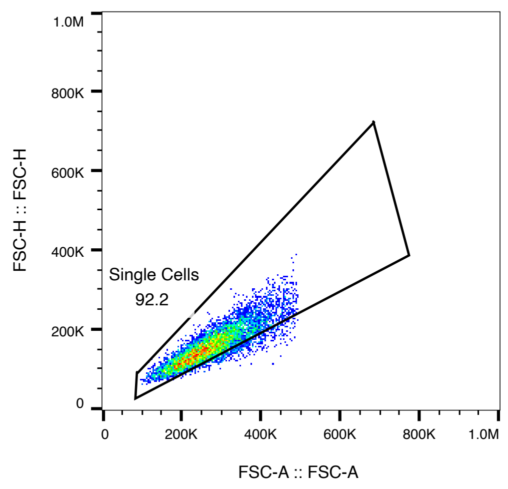

HxmCherry_1_Data Source - 1.fcs
cells
7318

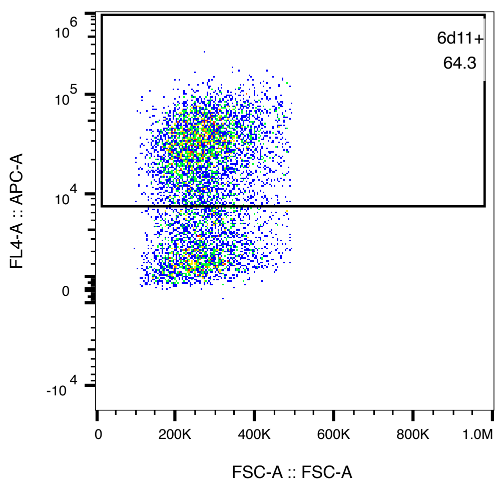

HxmCherry_1_Data Source - 1.fcs
Single Cells
6745

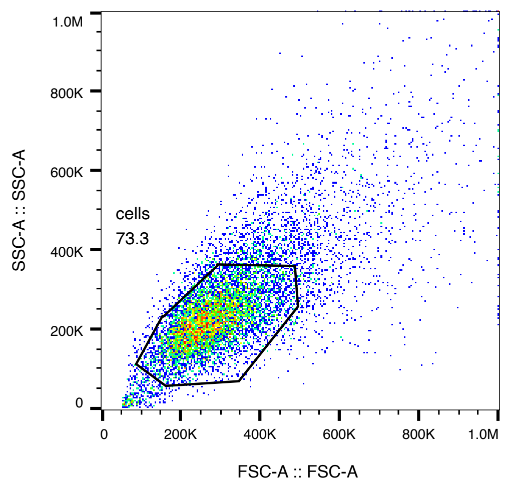

HxmCherry_2_Data Source - 1.fcs
Ungated
10000

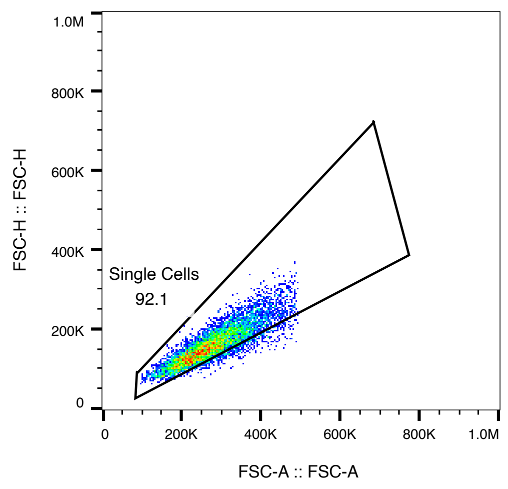

HxmCherry_2_Data Source - 1.fcs
cells
7329

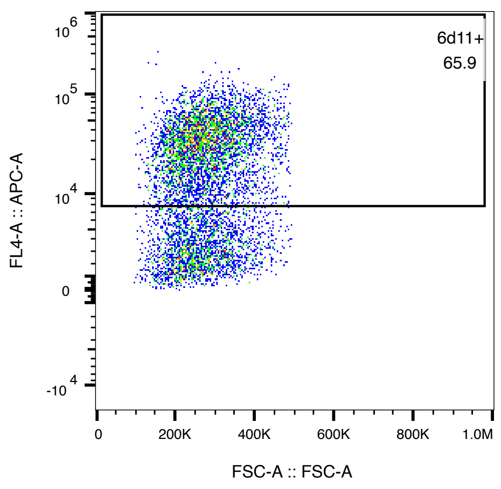

HxmCherry_2_Data Source - 1.fcs
Single Cells
6750

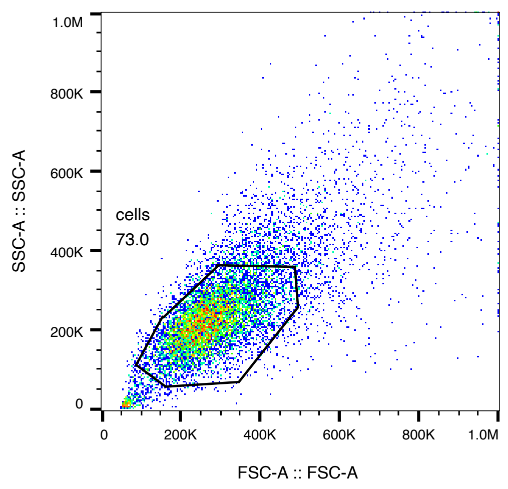

HxmCherry_3_Data Source - 1.fcs
Ungated
10000

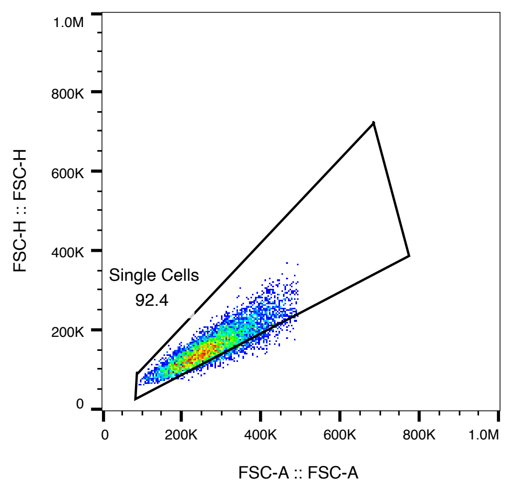

HxmCherry_3_Data Source - 1.fcs
cells
7304

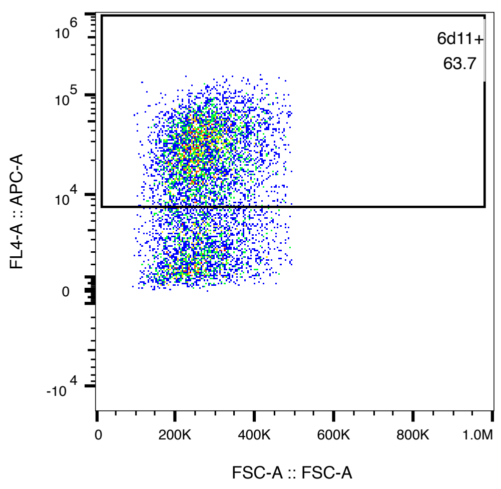

HxmCherry_3_Data Source - 1.fcs
Single Cells
6748

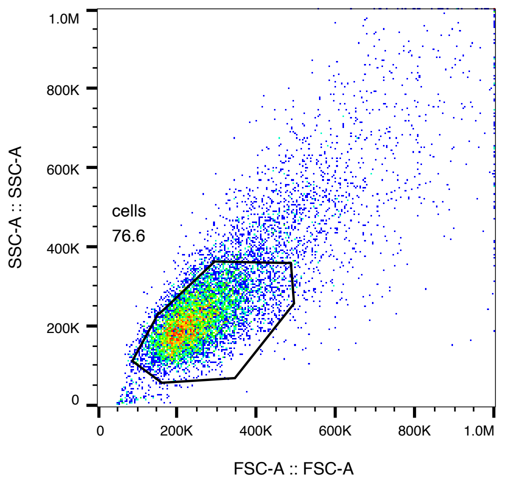

NTxTFAP_1_Data Source - 1.fcs
Ungated
10000

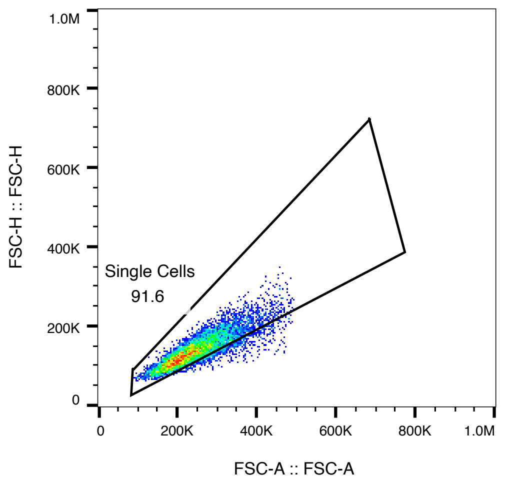

NTxTFAP_1_Data Source - 1.fcs
cells
7665

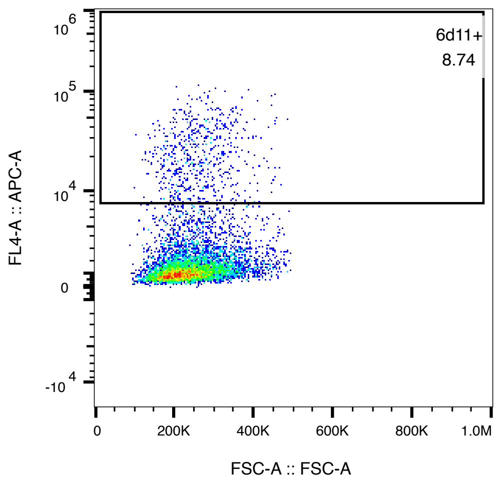

NTxTFAP_1_Data Source - 1.fcs
Single Cells
7024

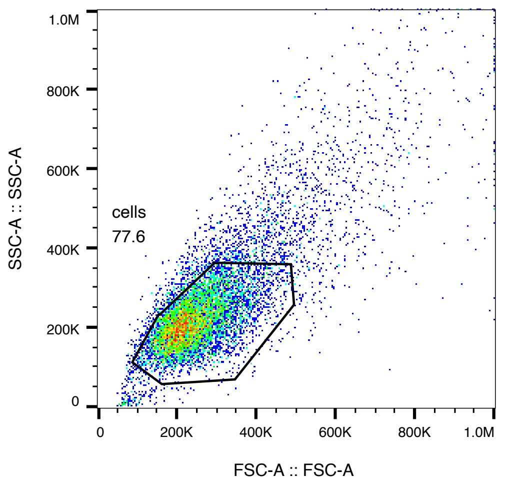

NTxTFAP_2_Data Source - 1.fcs
Ungated
10000

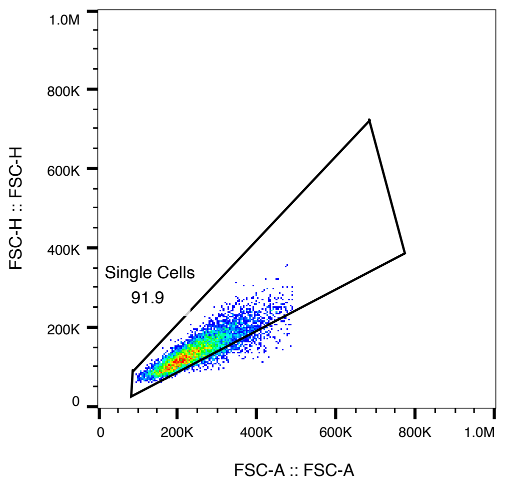

NTxTFAP_2_Data Source - 1.fcs
cells
7760

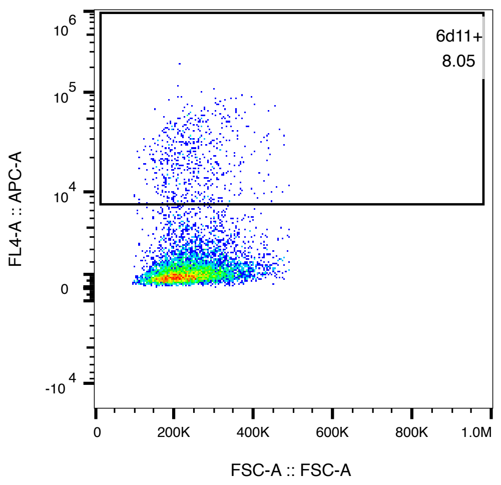

NTxTFAP_2_Data Source - 1.fcs
Single Cells
7130

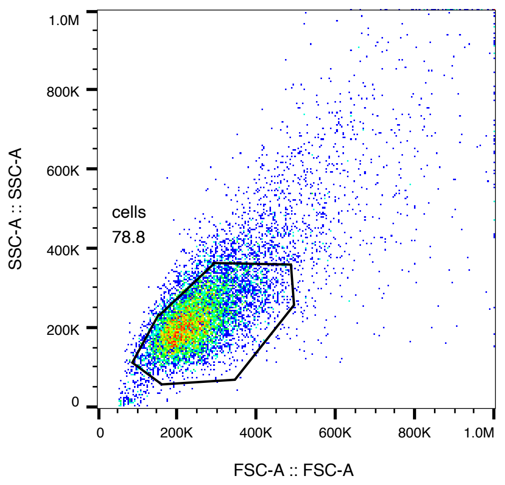

NTxTFAP_3_Data Source - 1.fcs
Ungated
10000

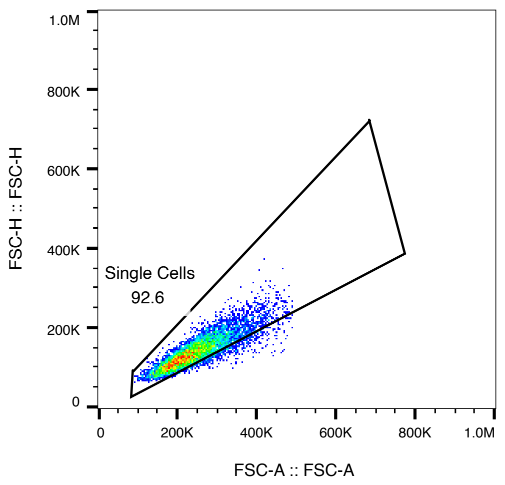

NTxTFAP_3_Data Source - 1.fcs
cells
7876

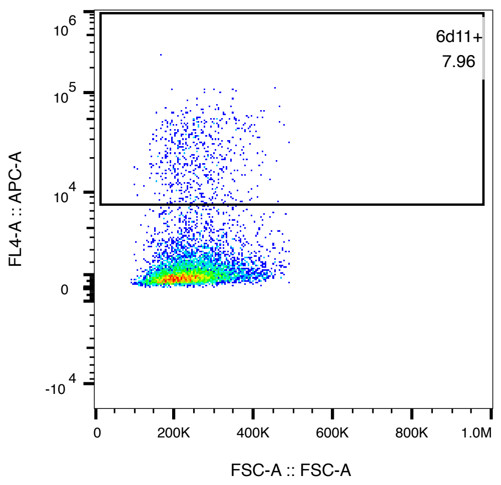

NTxTFAP_3_Data Source - 1.fcs
Single Cells
7290

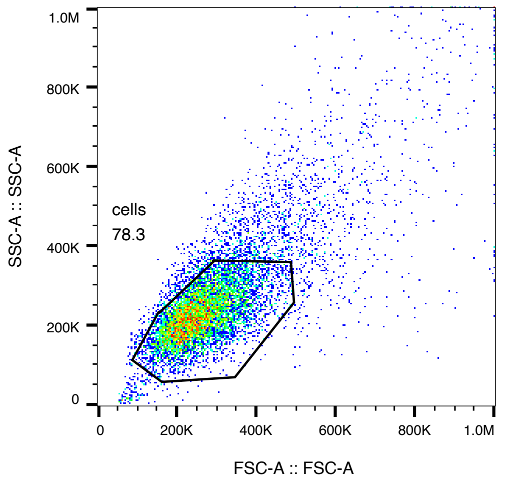

HxTFAP_1_Data Source - 1.fcs
Ungated
10000

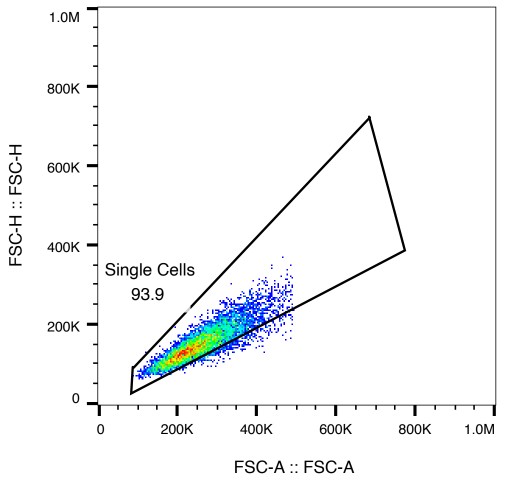

HxTFAP_1_Data Source - 1.fcs
cells
7834

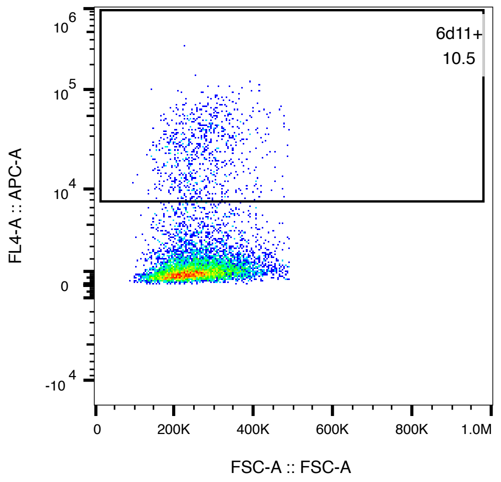

HxTFAP_1_Data Source - 1.fcs
Single Cells
7354

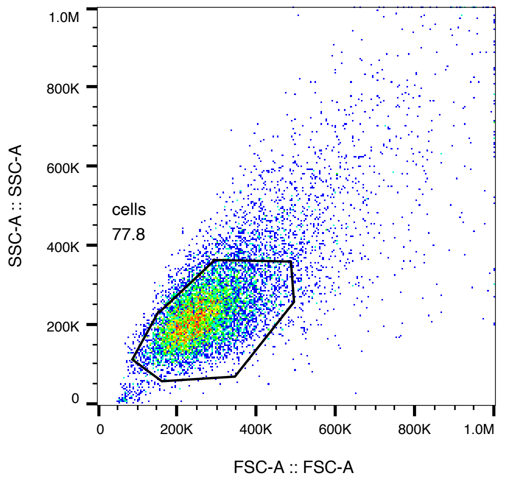

HxTFAP_2_Data Source - 1.fcs
Ungated
10000

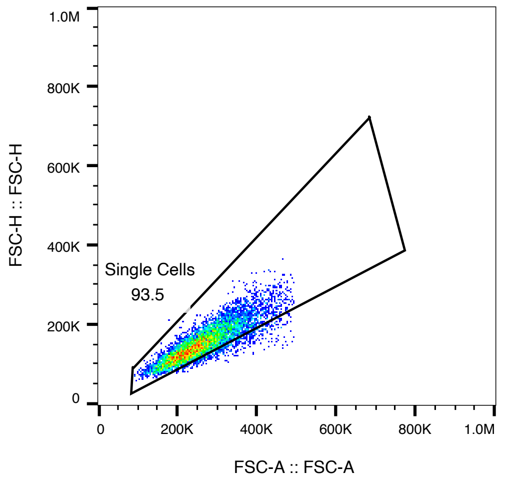

HxTFAP_2_Data Source - 1.fcs
cells
7777

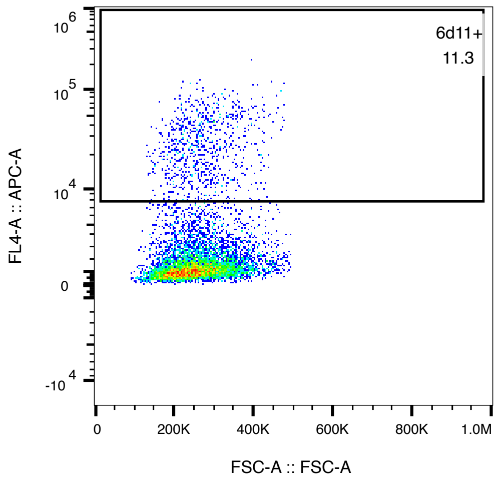

HxTFAP_2_Data Source - 1.fcs
Single Cells
7269

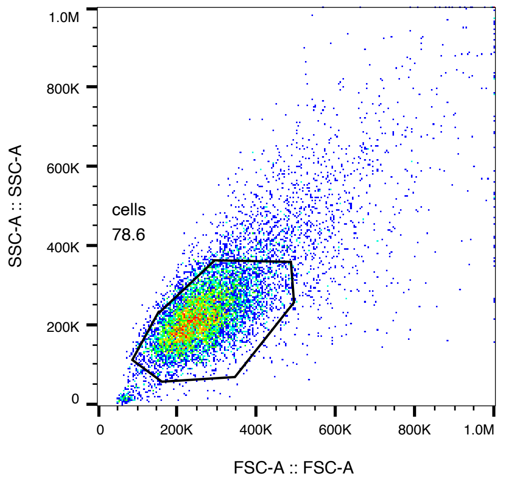

HxTFAP_3_Data Source - 1.fcs
Ungated
10000

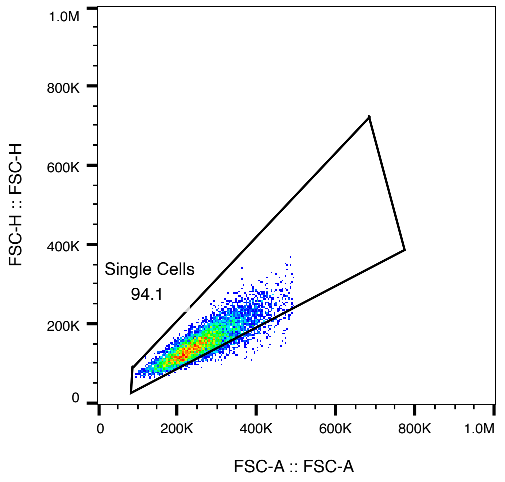

HxTFAP_3_Data Source - 1.fcs
cells
7857

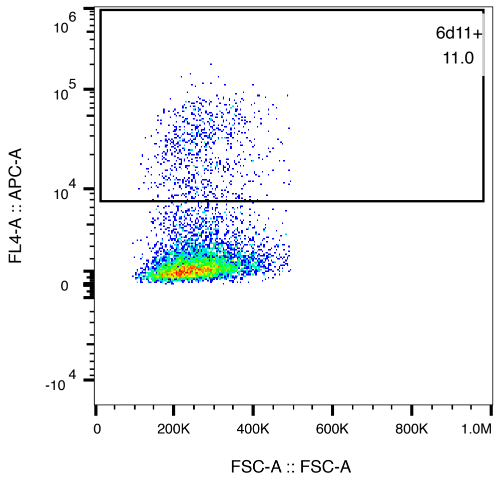

HxTFAP_3_Data Source - 1.fcs
Single Cells
7394

Fig 7D

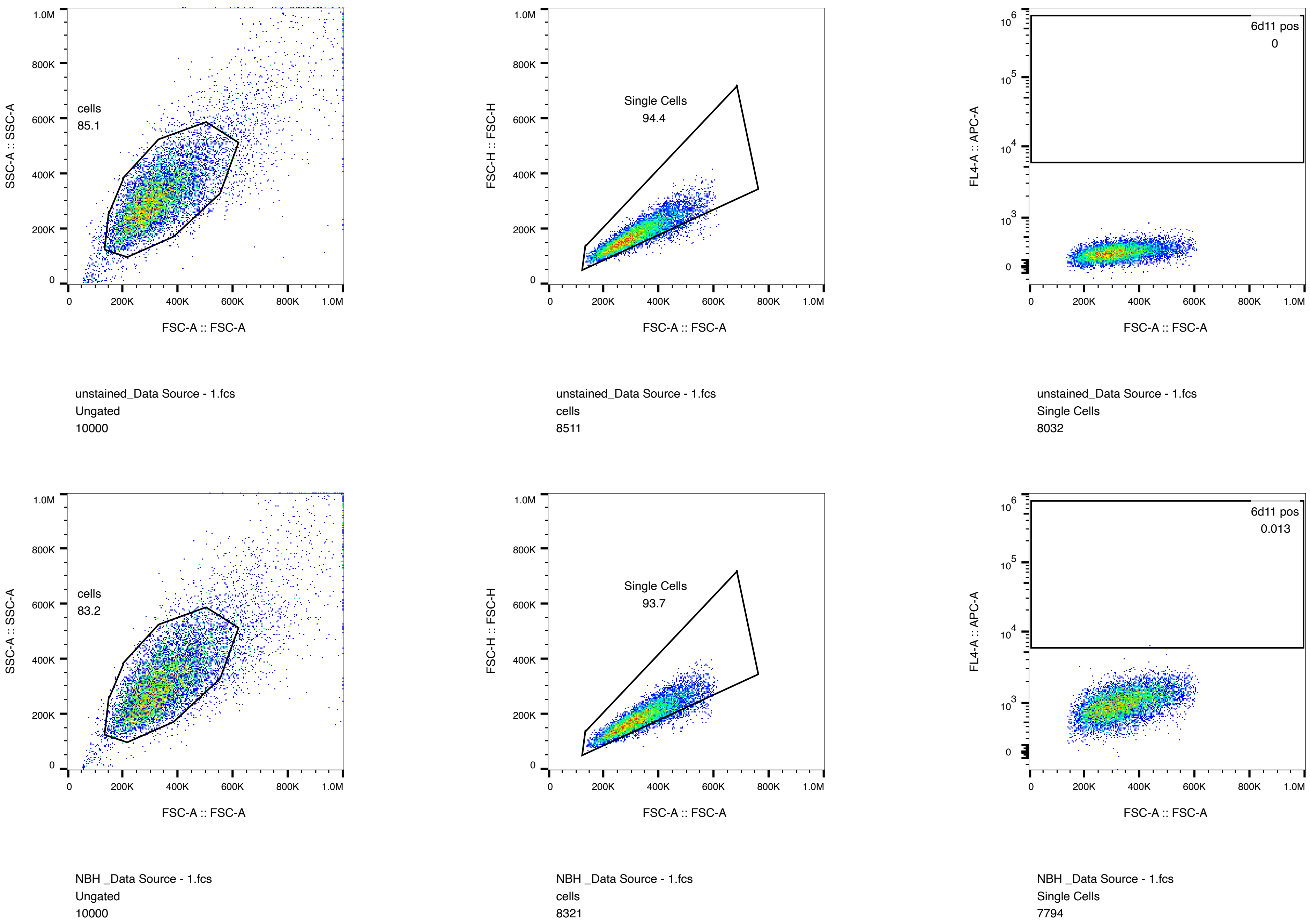

cells
85.1
SSC-A :: SSC-A
FSC-A :: FSC-A
Single Cells
94.4
FSC-H :: FSC-H
FSC-A :: FSC-A
6d11 pos
0
FL4-A :: APC-A
FSC-A :: FSC-A
unstained_Data Source - 1.fcs
Ungated
10000
unstained_Data Source - 1.fcs
cells
8511
unstained_Data Source - 1.fcs
Single Cells
8032
cells
83.2
SSC-A :: SSC-A
FSC-A :: FSC-A
Single Cells
93.7
FSC-H :: FSC-H
FSC-A :: FSC-A
6d11 pos
0.013
FL4-A :: APC-A
FSC-A :: FSC-A
NBH _Data Source - 1.fcs
Ungated
10000
NBH _Data Source - 1.fcs
cells
8321
NBH _Data Source - 1.fcs
Single Cells
7794

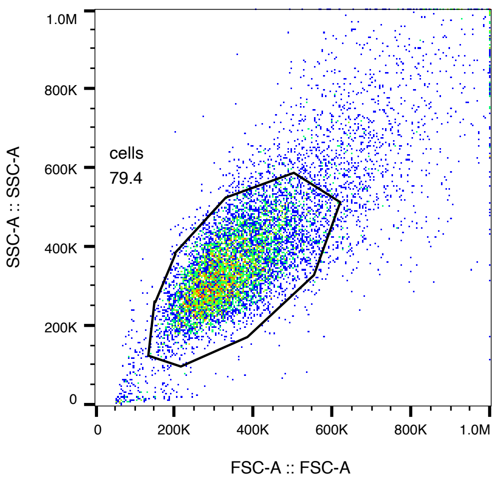

DMSO_NT_1_Data Source - 1.fcs
Ungated
10000

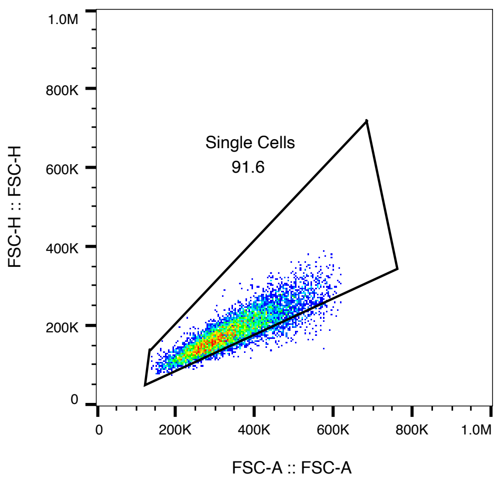

DMSO_NT_1_Data Source - 1.fcs
cells
7941

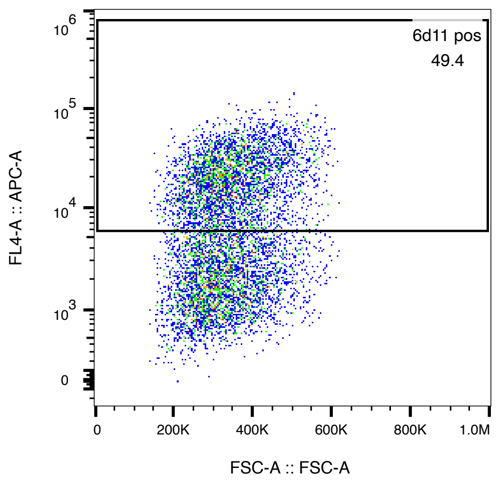

DMSO_NT_1_Data Source - 1.fcs
Single Cells
7275

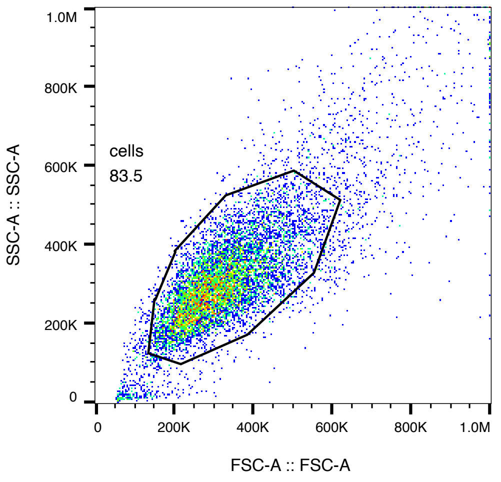

DMSO_NT_2_Data Source - 1.fcs
Ungated
10000

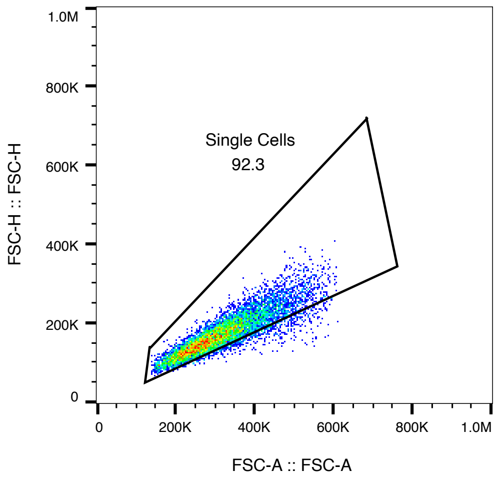

DMSO_NT_2_Data Source - 1.fcs
cells
8351

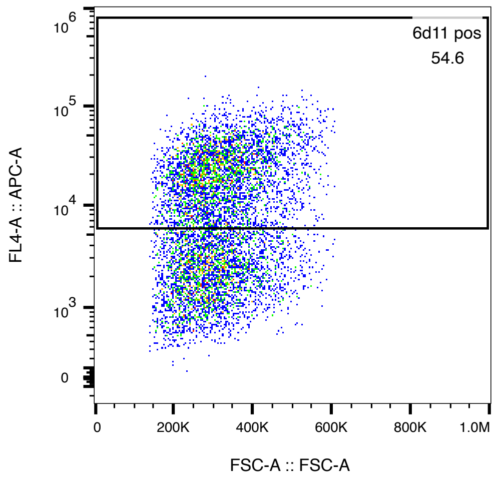

DMSO_NT_2_Data Source - 1.fcs
Single Cells
7705

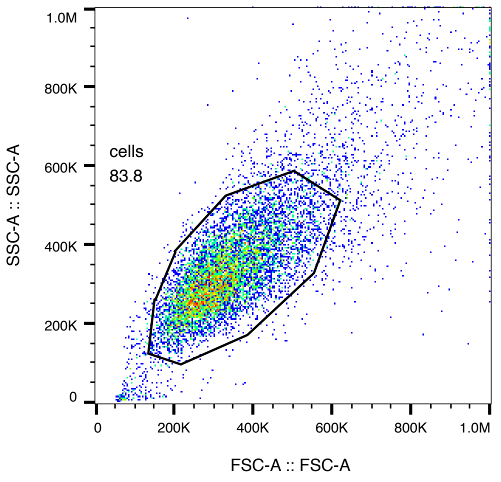

DMSO_NT_3_Data Source - 1.fcs
Ungated
10000

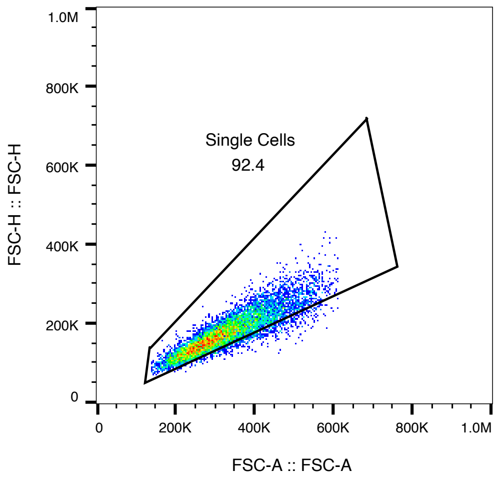

DMSO_NT_3_Data Source - 1.fcs
cells
8378

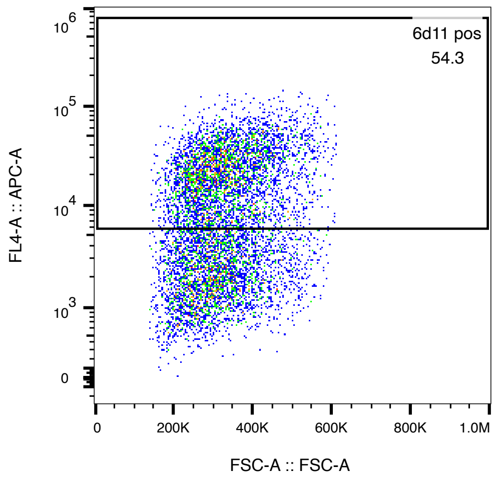

DMSO_NT_3_Data Source - 1.fcs
Single Cells
7741

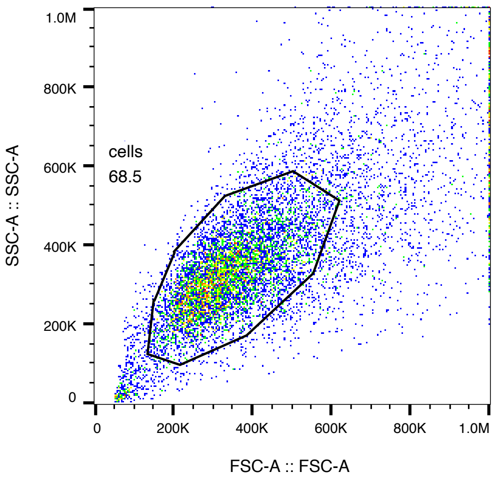

DMSO_H_1_Data Source - 1.fcs
Ungated
10000

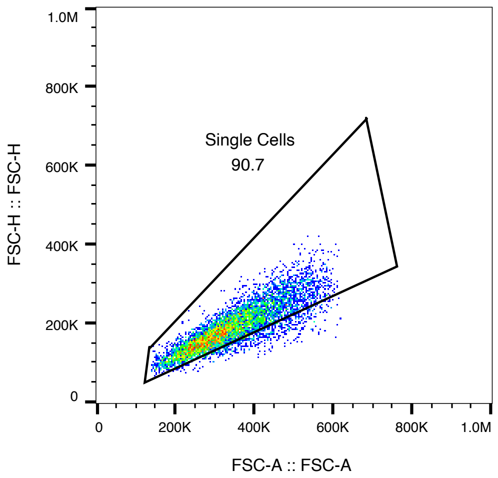

DMSO_H_1_Data Source - 1.fcs
cells
6846

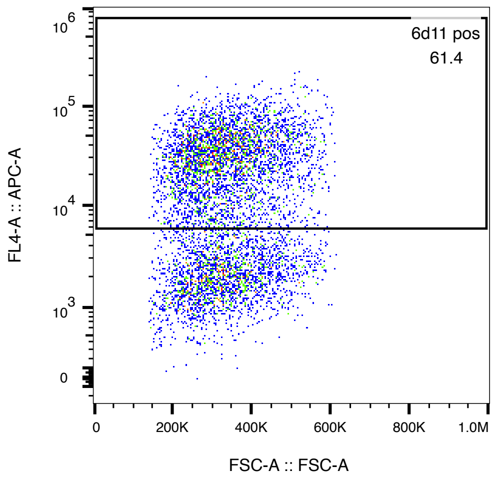

DMSO_H_1_Data Source - 1.fcs
Single Cells
6212

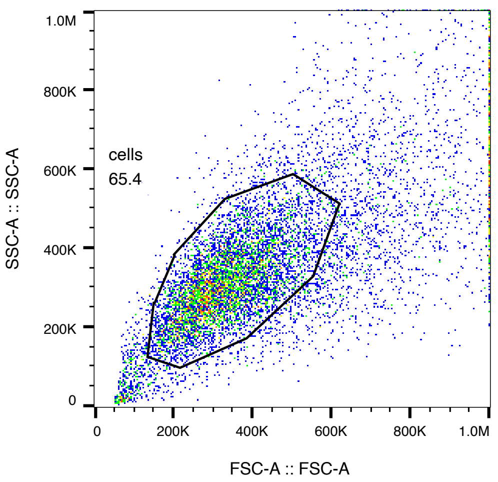

DMSO_H_2_Data Source - 1.fcs
Ungated
10000

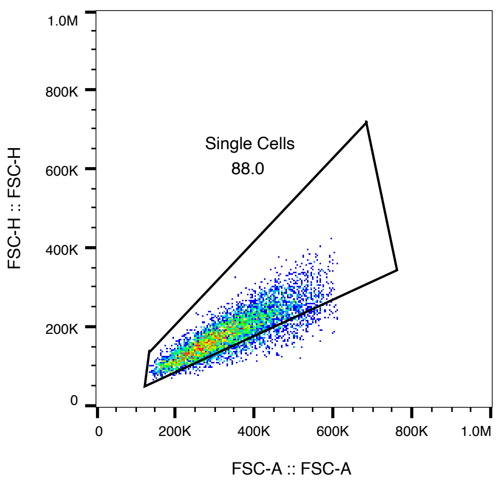

DMSO_H_2_Data Source - 1.fcs
cells
6537

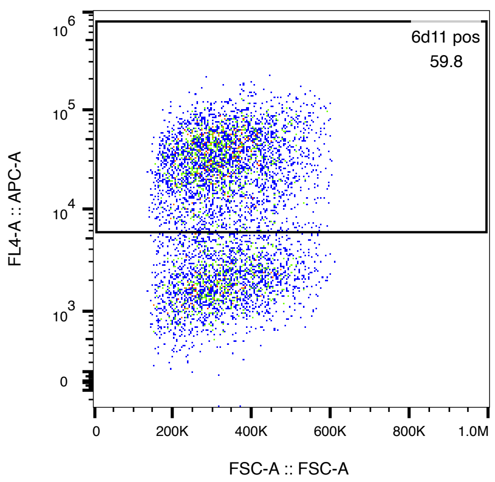

DMSO_H_2_Data Source - 1.fcs
Single Cells
5755

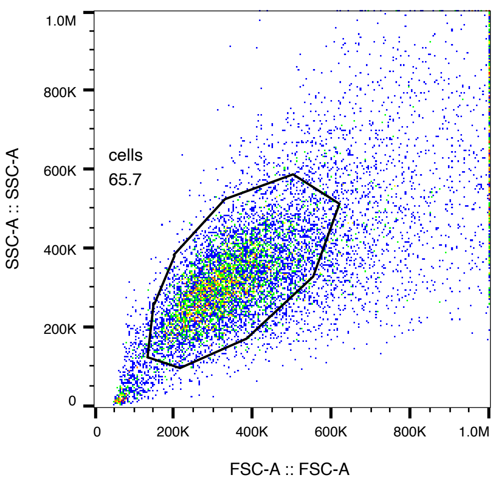

DMSO_H_3_Data Source - 1.fcs
Ungated
10000

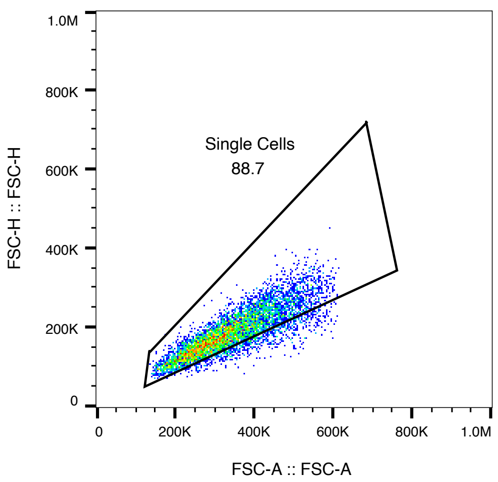

DMSO_H_3_Data Source - 1.fcs
cells
6569

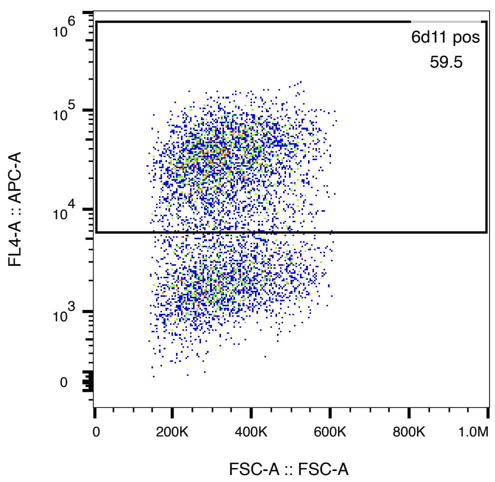

DMSO_H_3_Data Source - 1.fcs
Single Cells
5827

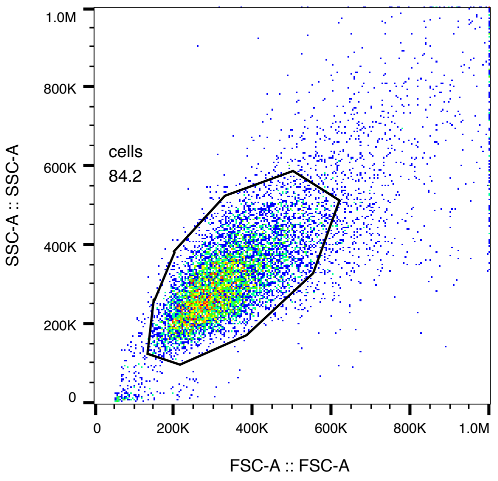

Torin_NT_1_Data Source - 1.fcs
Ungated
10000

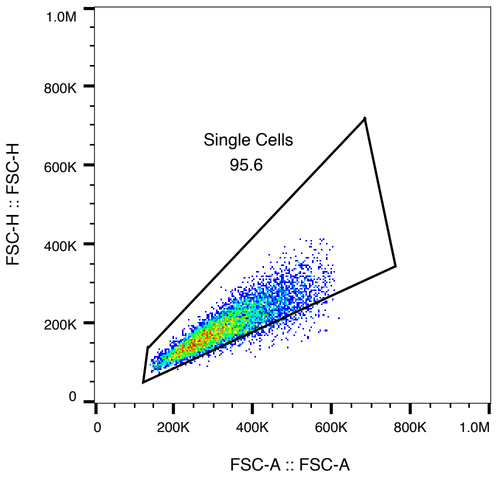

Torin_NT_1_Data Source - 1.fcs
cells
8424

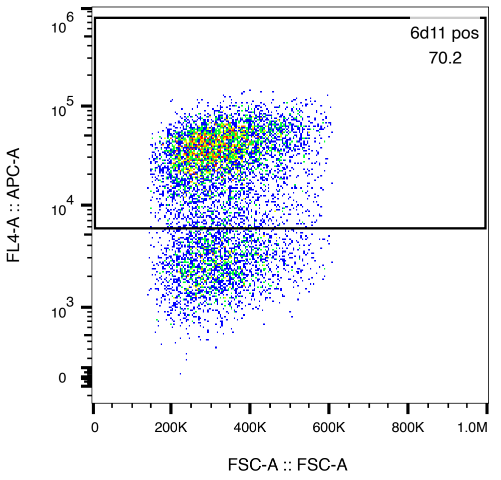

Torin_NT_1_Data Source - 1.fcs
Single Cells
8055

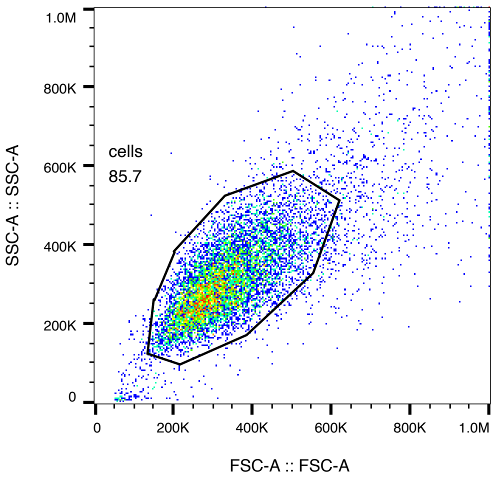

Torin_NT_2_Data Source - 1.fcs
Ungated
10000

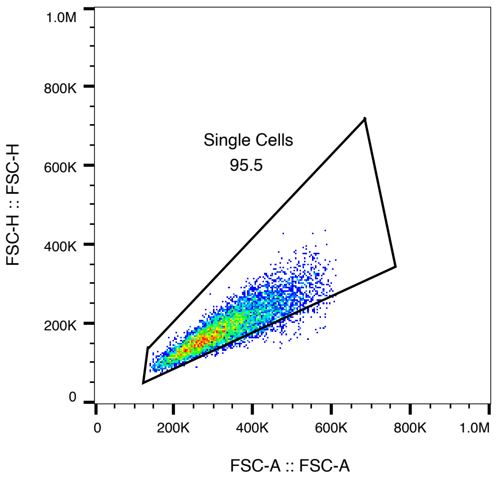

Torin_NT_2_Data Source - 1.fcs
cells
8570

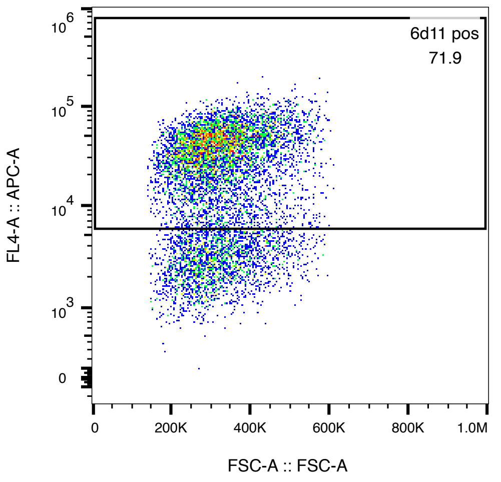

Torin_NT_2_Data Source - 1.fcs
Single Cells
8185

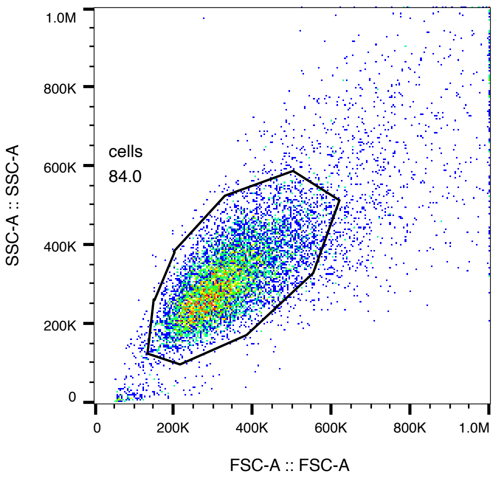

Torin_NT_3_Data Source - 1.fcs
Ungated
10000

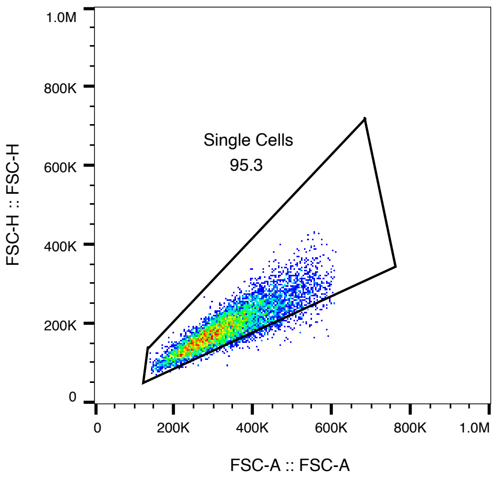

Torin_NT_3_Data Source - 1.fcs
cells
8403

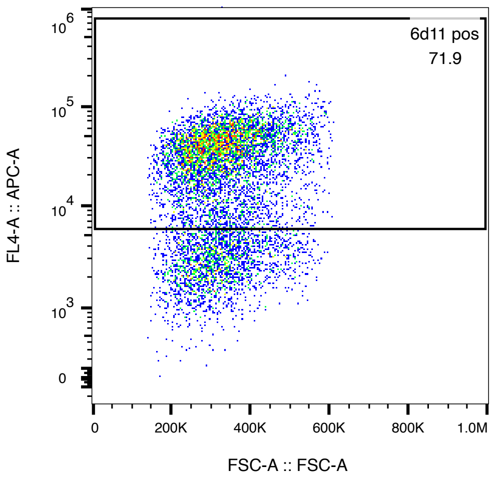

Torin_NT_3_Data Source - 1.fcs
Single Cells
8010

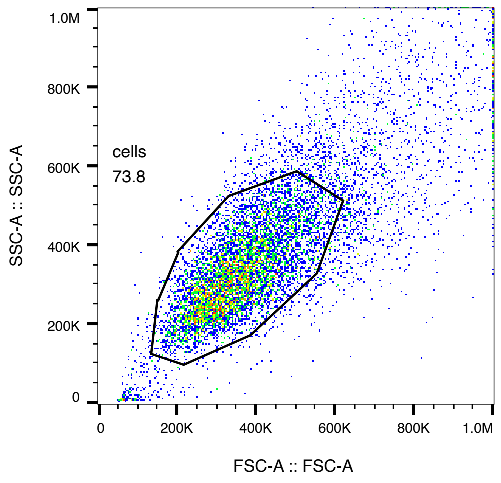

Torin_H_1_Data Source - 1.fcs
Ungated
10000

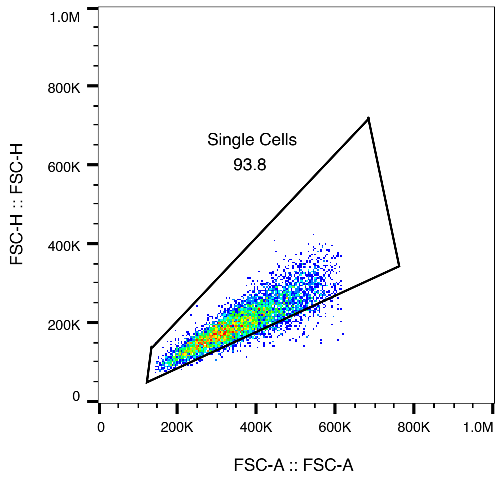

Torin_H_1_Data Source - 1.fcs
cells
7376

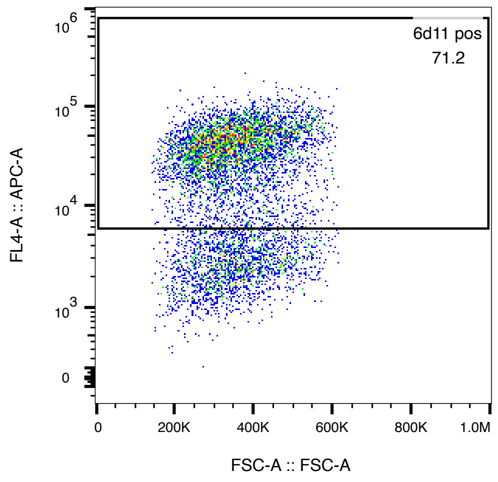

Torin_H_1_Data Source - 1.fcs
Single Cells
6919

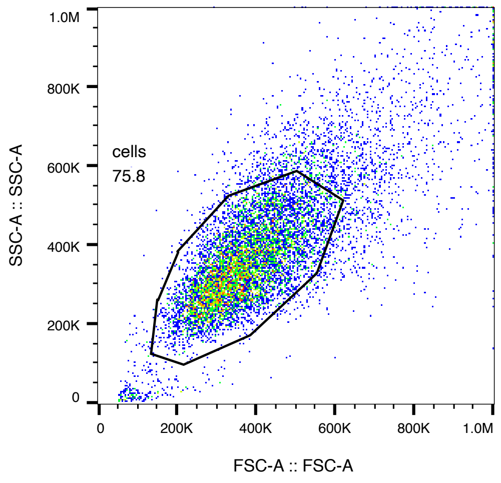

Torin_H_2_Data Source - 1.fcs
Ungated
10000

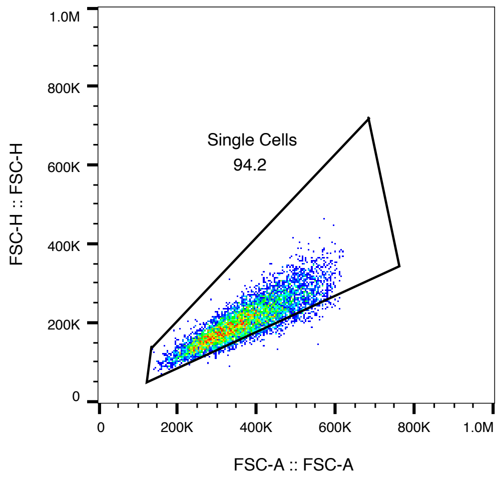

Torin_H_2_Data Source - 1.fcs
cells
7584

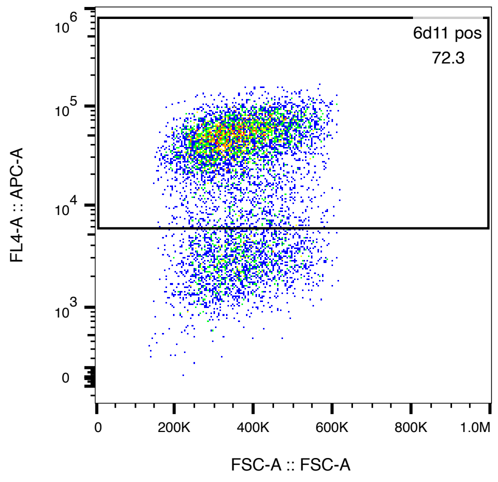

Torin_H_2_Data Source - 1.fcs
Single Cells
7146

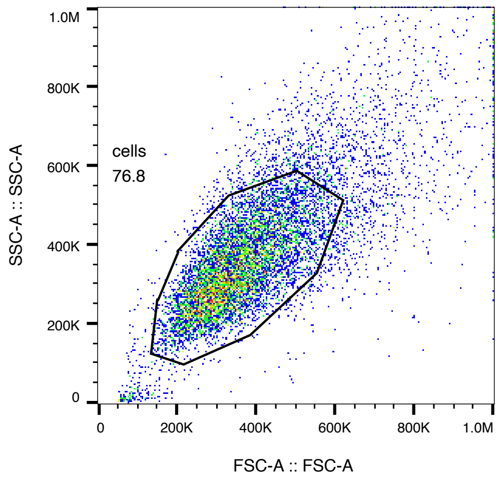

Torin_H_3_Data Source - 1.fcs
Ungated
10000

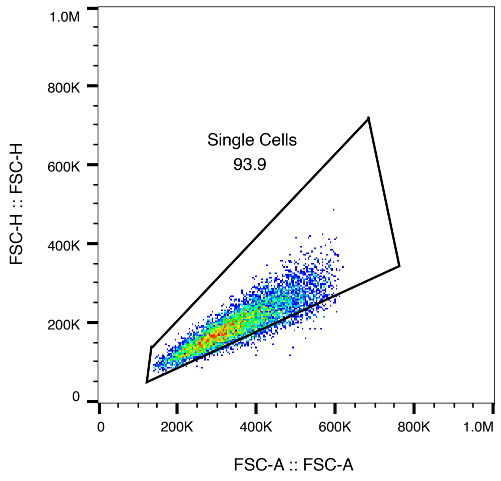

Torin_H_3_Data Source - 1.fcs
cells
7681

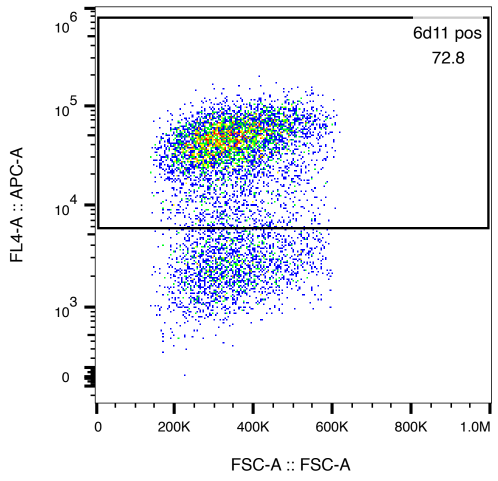

Torin_H_3_Data Source - 1.fcs
Single Cells
7214

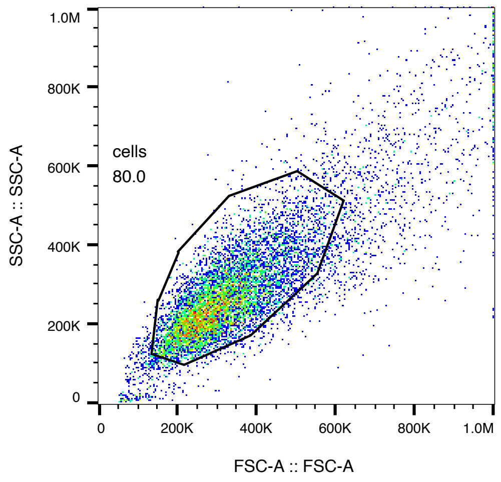

Rapa_NT_1_Data Source - 1.fcs
Ungated
10000

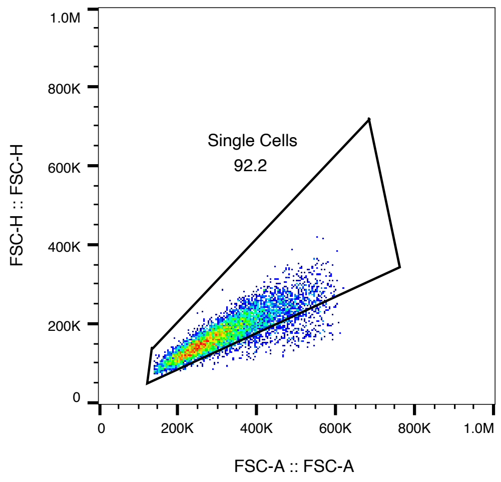

Rapa_NT_1_Data Source - 1.fcs
cells
8005

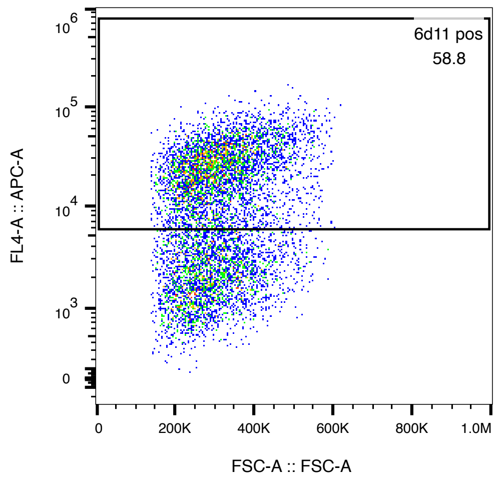

Rapa_NT_1_Data Source - 1.fcs
Single Cells
7384

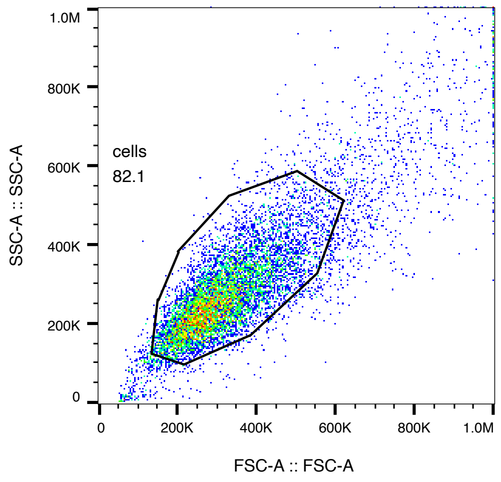

Rapa_NT_2_Data Source - 1.fcs
Ungated
10000

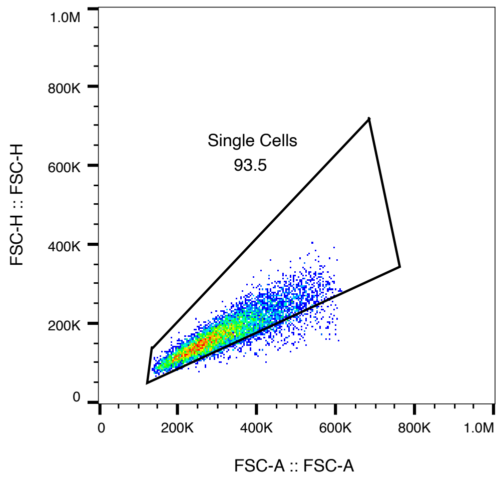

Rapa_NT_2_Data Source - 1.fcs
cells
8209

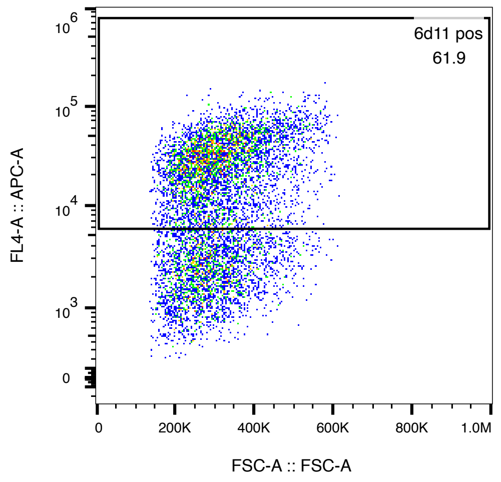

Rapa_NT_2_Data Source - 1.fcs
Single Cells
7672

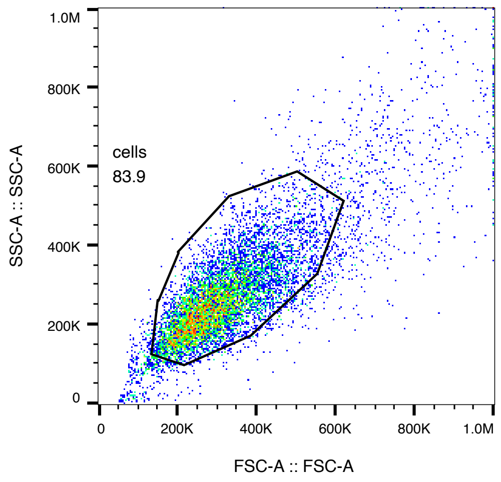

Rapa_NT_3_Data Source - 1.fcs
Ungated
10000

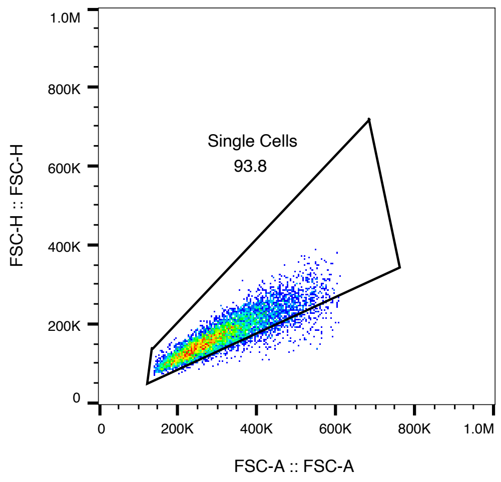

Rapa_NT_3_Data Source - 1.fcs
cells
8390

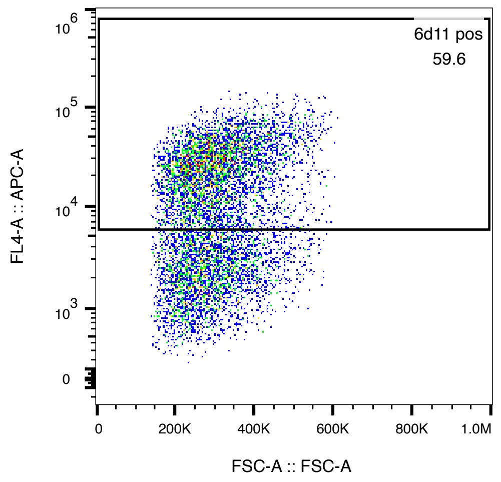

Rapa_NT_3_Data Source - 1.fcs
Single Cells
7872

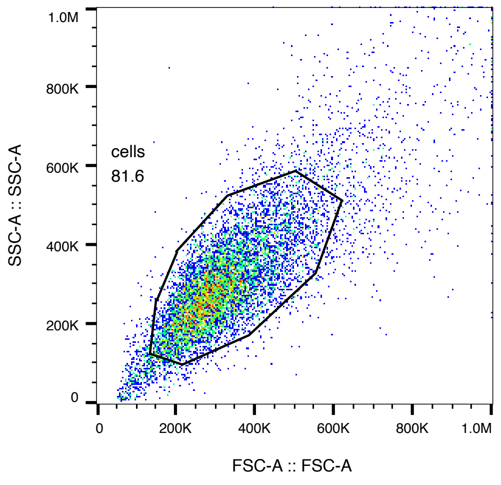

Rapa_H_1_Data Source - 1.fcs
Ungated
10000

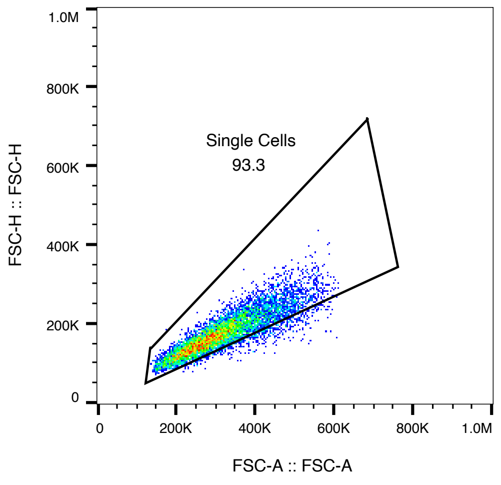

Rapa_H_1_Data Source - 1.fcs
cells
8156

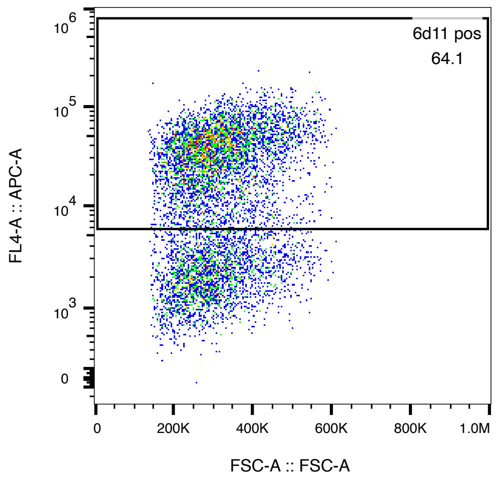

Rapa_H_1_Data Source - 1.fcs
Single Cells
7606

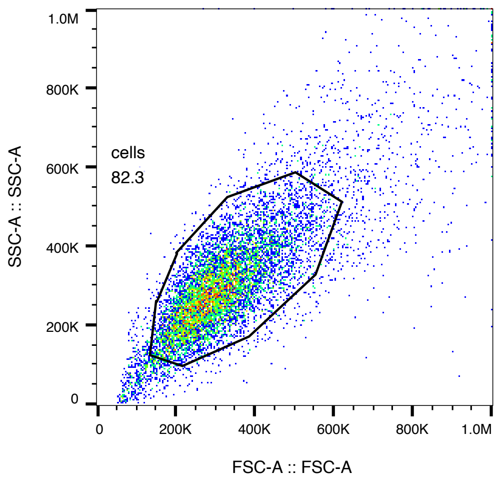

Rapa_H_2_Data Source - 1.fcs
Ungated
10000

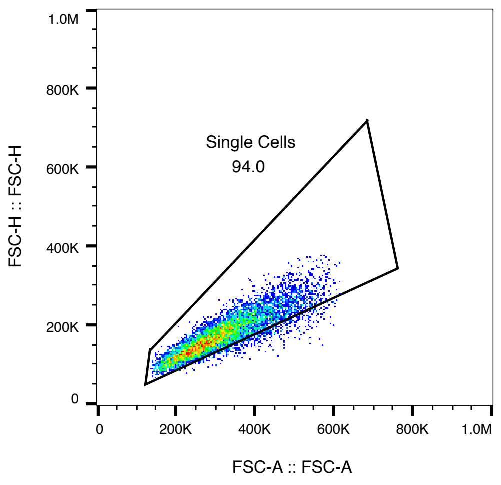

Rapa_H_2_Data Source - 1.fcs
cells
8226

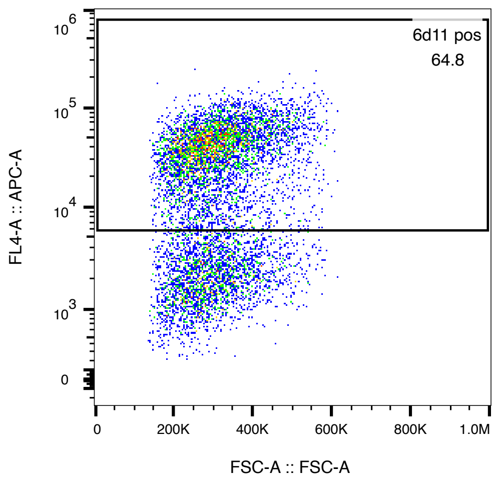

Rapa_H_2_Data Source - 1.fcs
Single Cells
7731

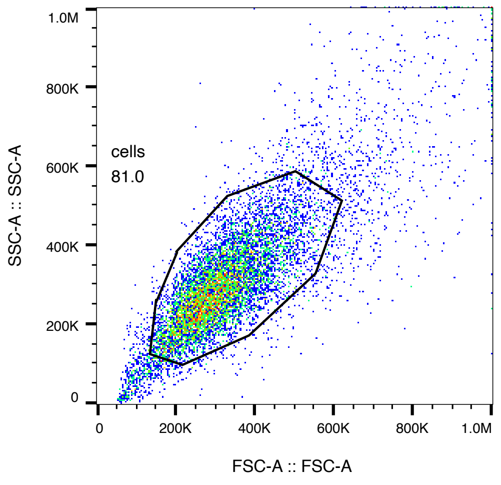

Rapa_H_3_Data Source - 1.fcs
Ungated
10000

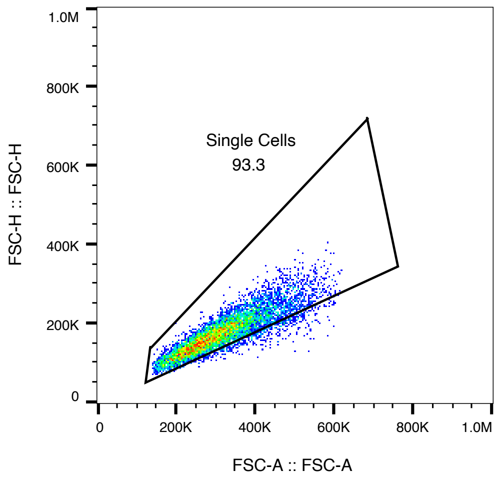

Rapa_H_3_Data Source - 1.fcs
cells
8100

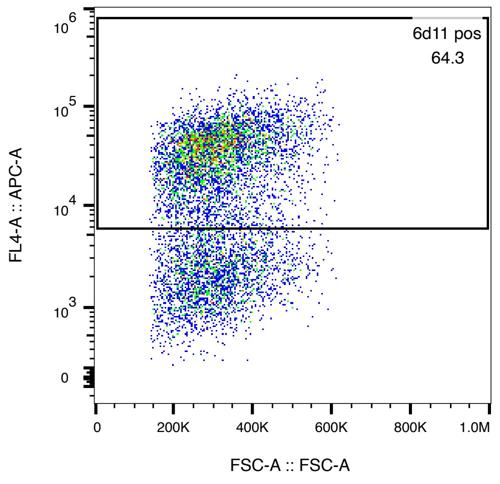

Rapa_H_3_Data Source - 1.fcs
Single Cells
7555

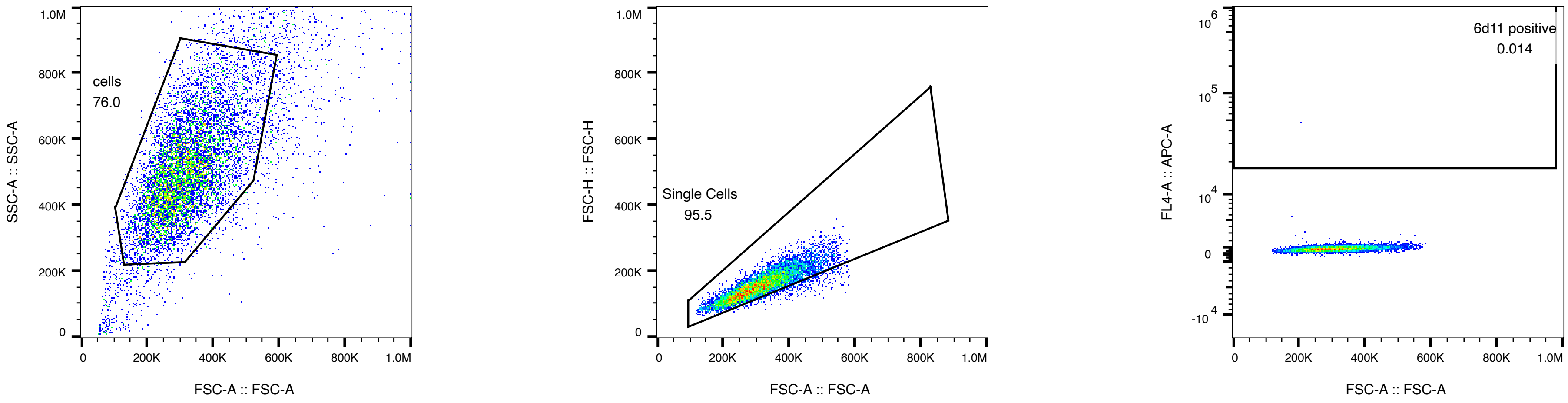

unstained_Data Source - 1.fcs
Ungated
10000

unstained_Data Source - 1.fcs
cells
7599

unstained_Data Source - 1.fcs
Single Cells
7258

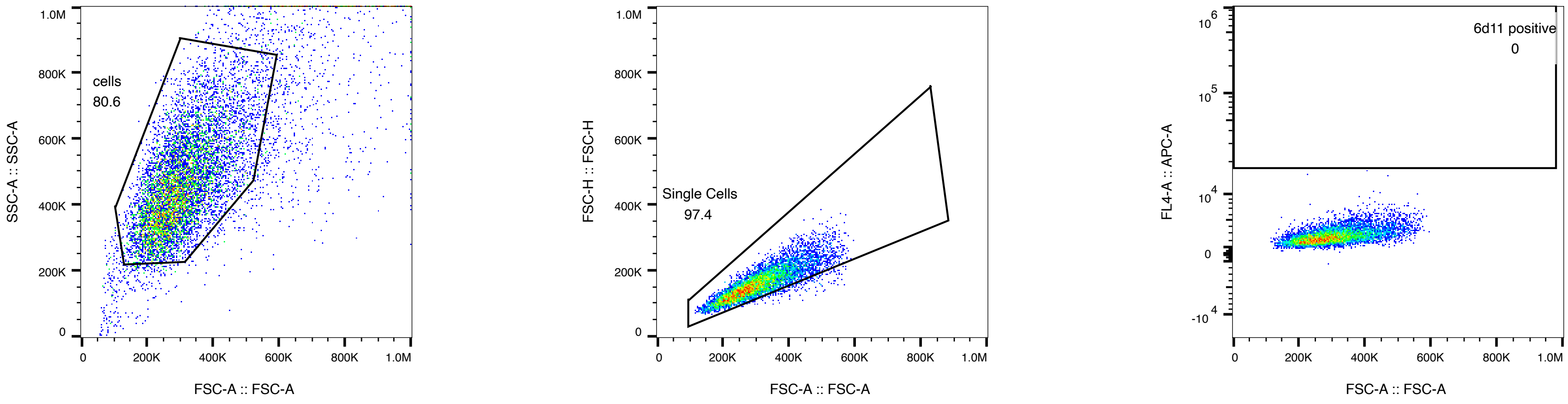

NBH _Data Source - 1.fcs
Ungated
10000

NBH _Data Source - 1.fcs
cells
8062

NBH _Data Source - 1.fcs
Single Cells
7856

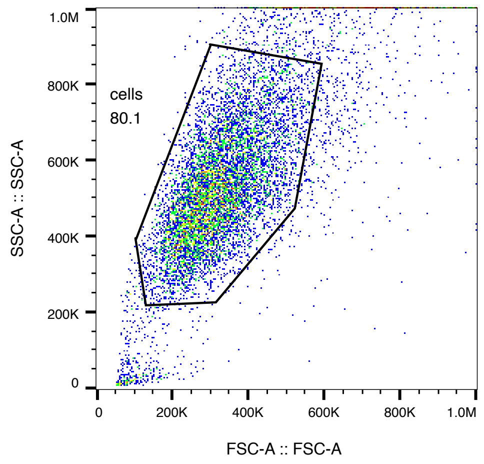

DMSO_NT_1_Data Source - 1.fcs
Ungated
10000

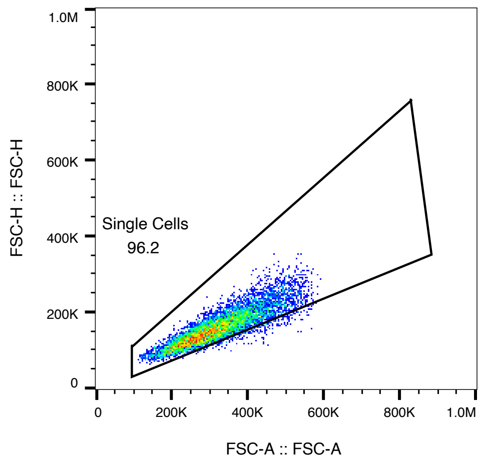

DMSO_NT_1_Data Source - 1.fcs
cells
8011

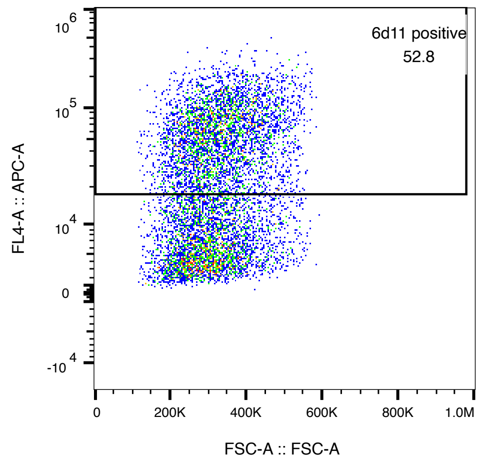

DMSO_NT_1_Data Source - 1.fcs
Single Cells
7708

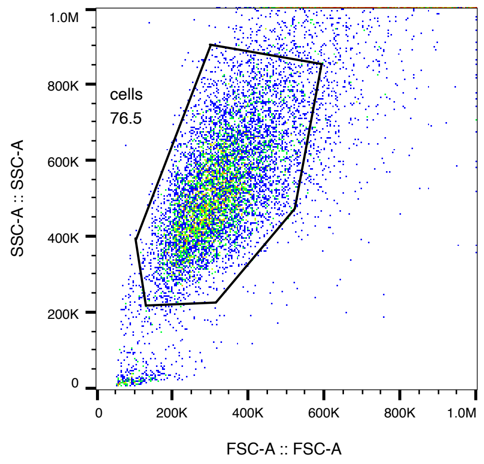

DMSO_NT_2_Data Source - 1.fcs
Ungated
10000

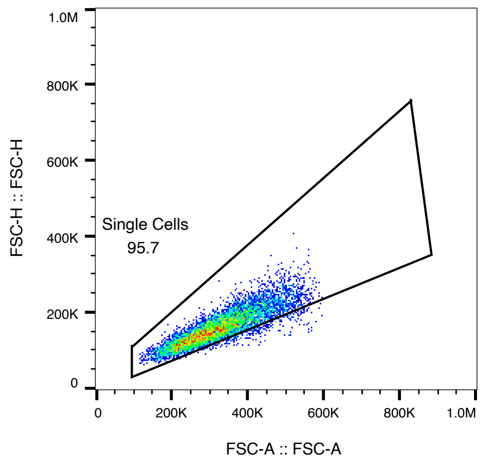

DMSO_NT_2_Data Source - 1.fcs
cells
7649

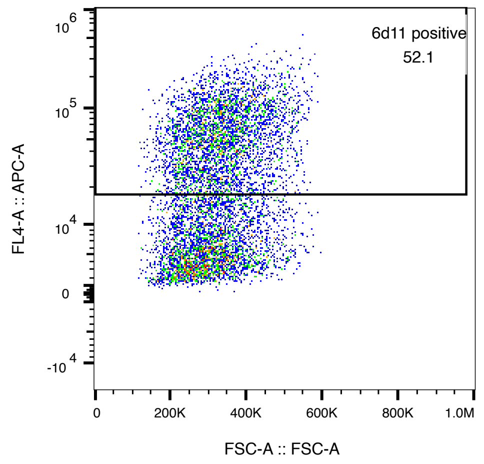

DMSO_NT_2_Data Source - 1.fcs
Single Cells
7322

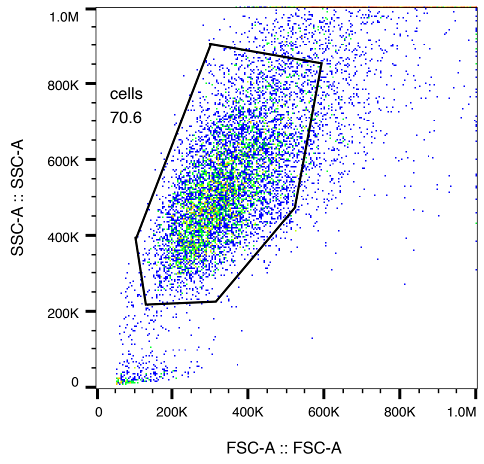

DMSO_NT_3_Data Source - 1.fcs
Ungated
10000

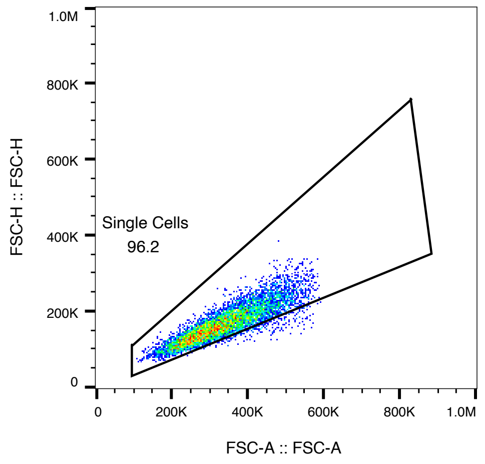

DMSO_NT_3_Data Source - 1.fcs
cells
7062

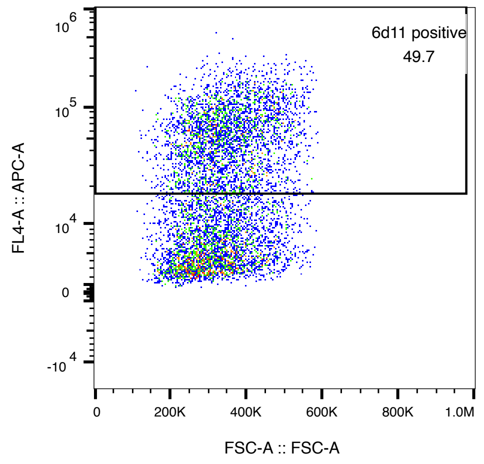

DMSO_NT_3_Data Source - 1.fcs
Single Cells
6795

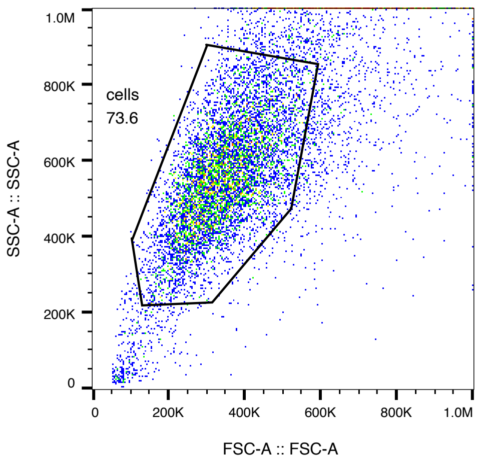

DMSO_H_1_Data Source - 1.fcs
Ungated
10000

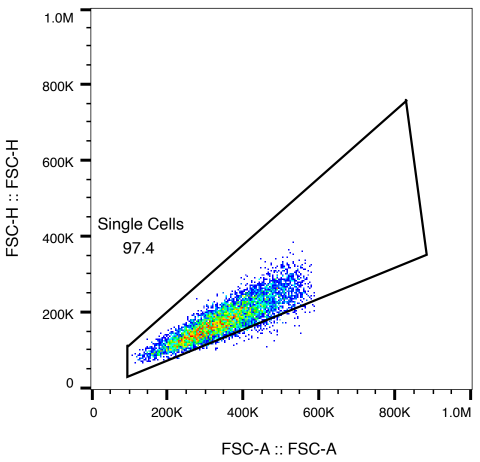

DMSO_H_1_Data Source - 1.fcs
cells
7363

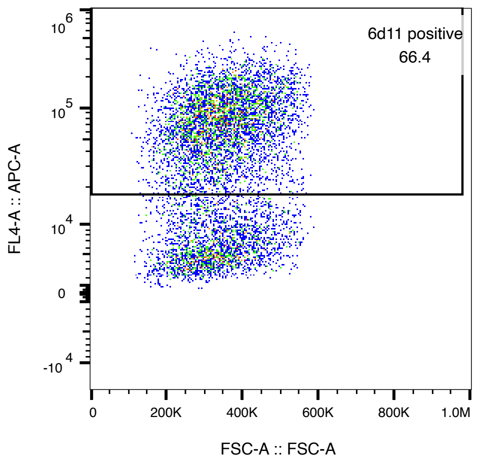

DMSO_H_1_Data Source - 1.fcs
Single Cells
7174

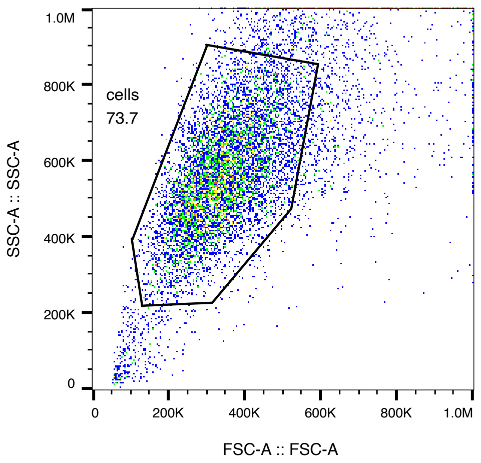

DMSO_H_2_Data Source - 1.fcs
Ungated
10000

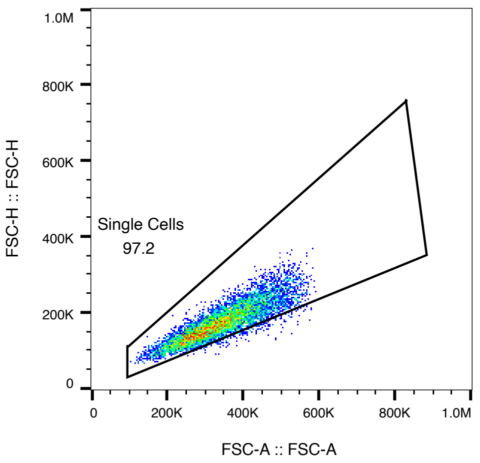

DMSO_H_2_Data Source - 1.fcs
cells
7374

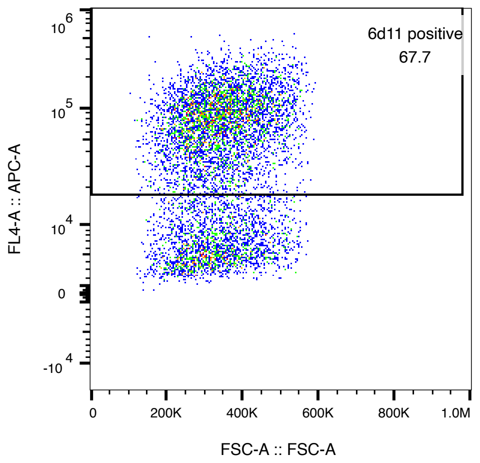

DMSO_H_2_Data Source - 1.fcs
Single Cells
7165

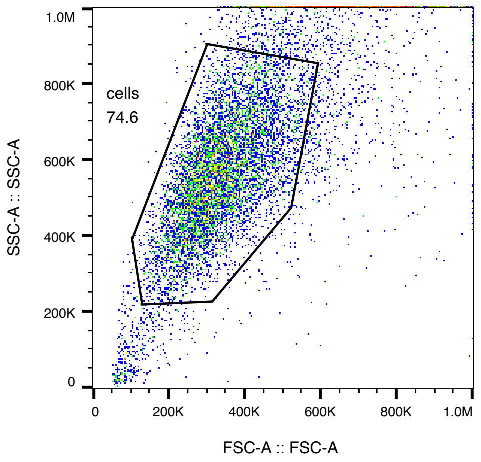

DMSO_H_3_Data Source - 1.fcs
Ungated
10000

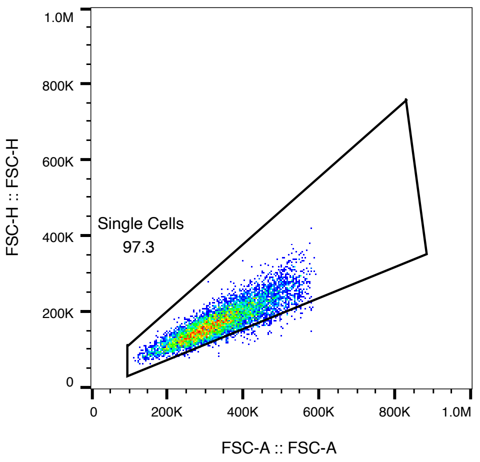

DMSO_H_3_Data Source - 1.fcs
cells
7459

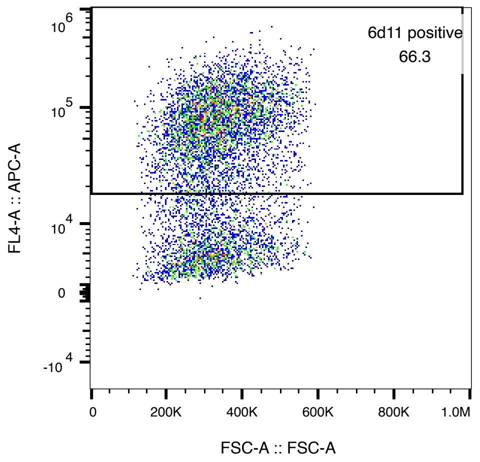

DMSO_H_3_Data Source - 1.fcs
Single Cells
7257

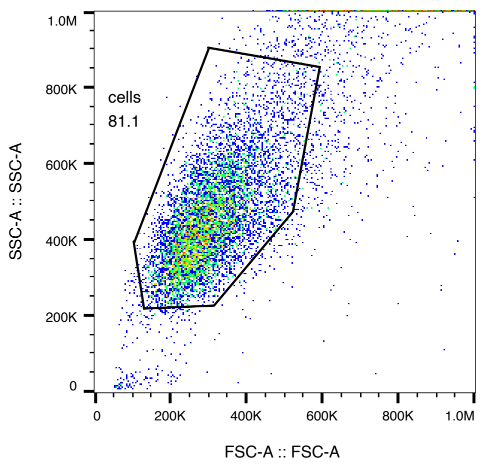

Torin_NT_1_Data Source - 1.fcs
Ungated
10000

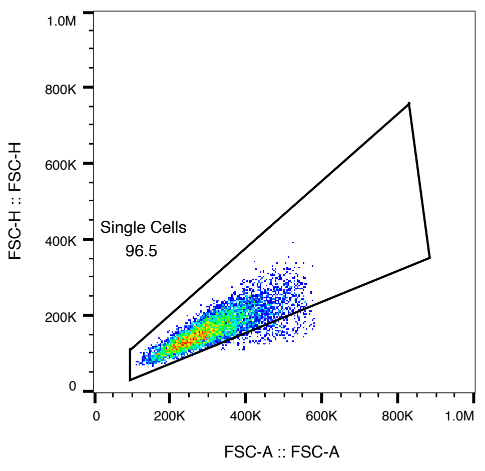

Torin_NT_1_Data Source - 1.fcs
cells
8110

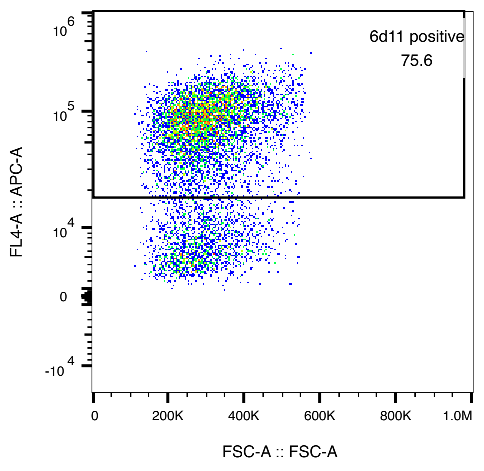

Torin_NT_1_Data Source - 1.fcs
Single Cells
7828

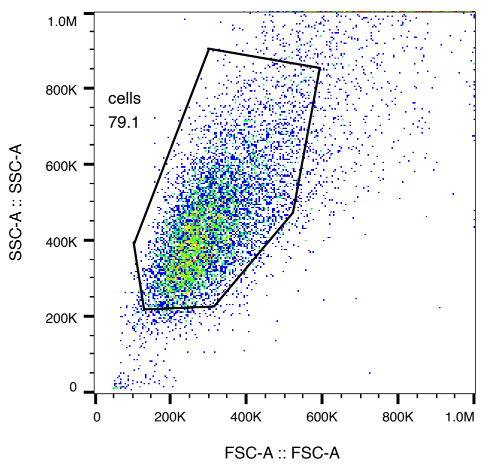

Torin_NT_2_Data Source - 1.fcs
Ungated
10000

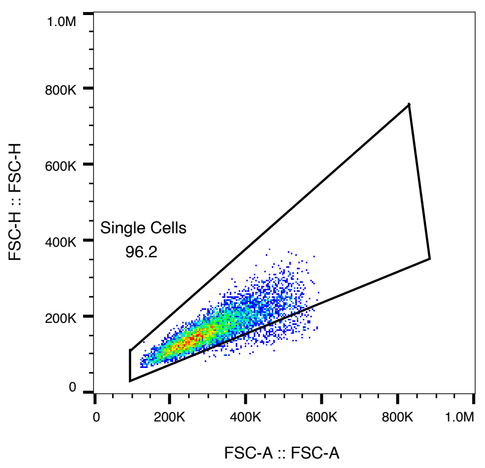

Torin_NT_2_Data Source - 1.fcs
cells
7909

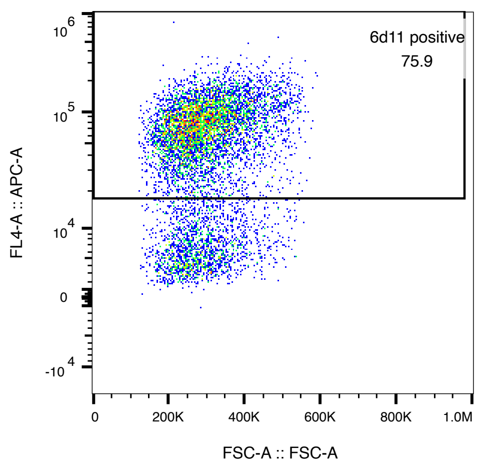

Torin_NT_2_Data Source - 1.fcs
Single Cells
7605

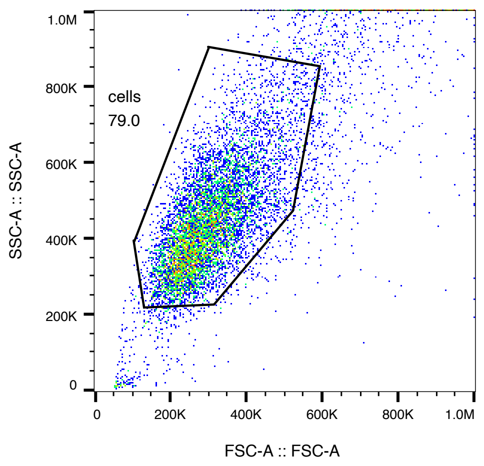

Torin_NT_3_Data Source - 1.fcs
Ungated
10000

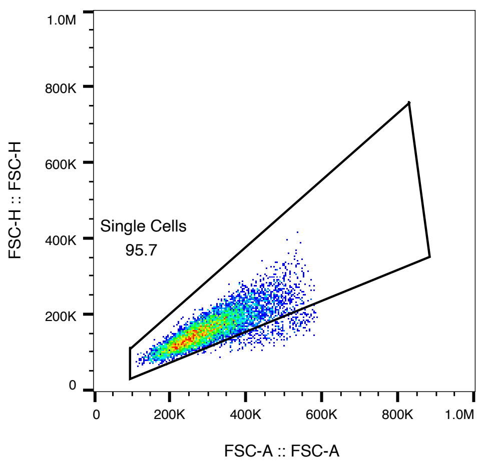

Torin_NT_3_Data Source - 1.fcs
cells
7898

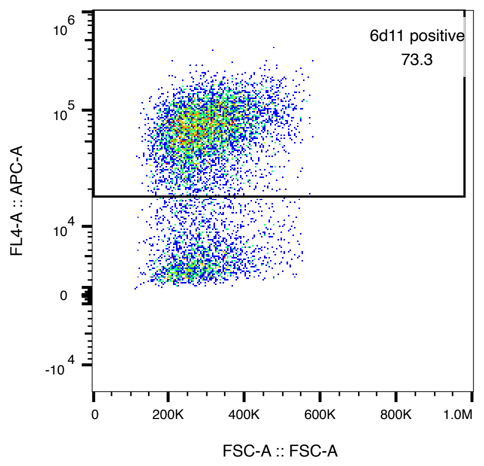

Torin_NT_3_Data Source - 1.fcs
Single Cells
7559

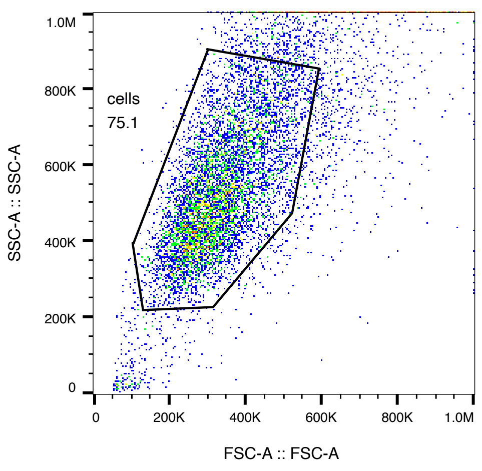

Torin_H_1_Data Source - 1.fcs
Ungated
10000

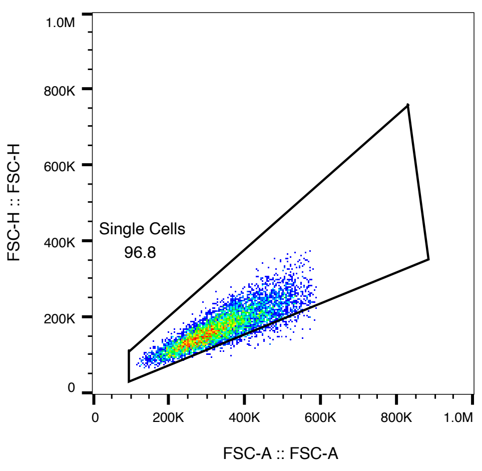

Torin_H_1_Data Source - 1.fcs
cells
7513

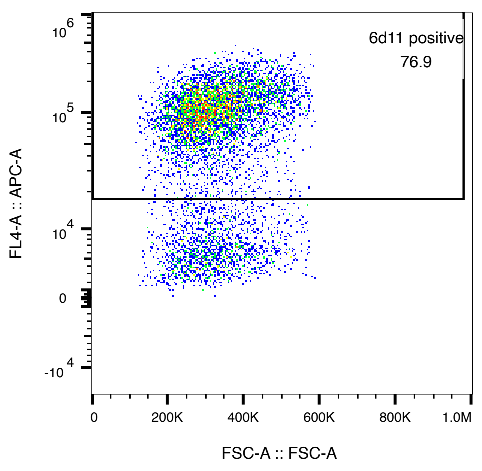

Torin_H_1_Data Source - 1.fcs
Single Cells
7271

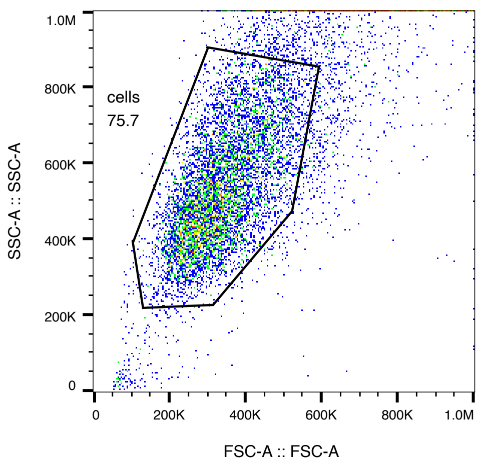

Torin_H_2_Data Source - 1.fcs
Ungated
10000

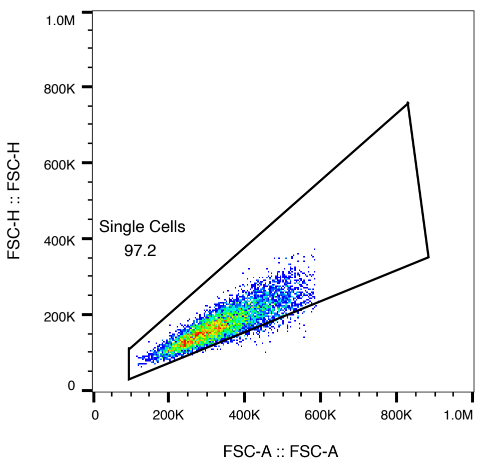

Torin_H_2_Data Source - 1.fcs
cells
7568

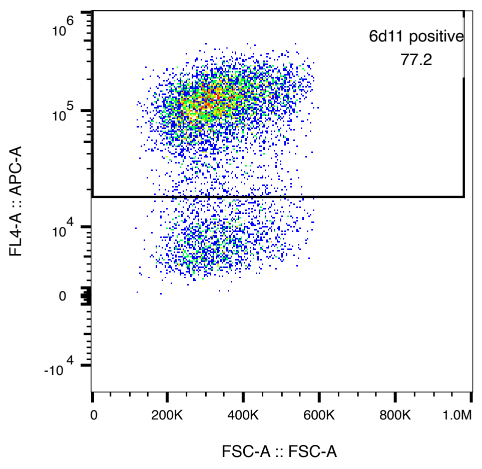

Torin_H_2_Data Source - 1.fcs
Single Cells
7353

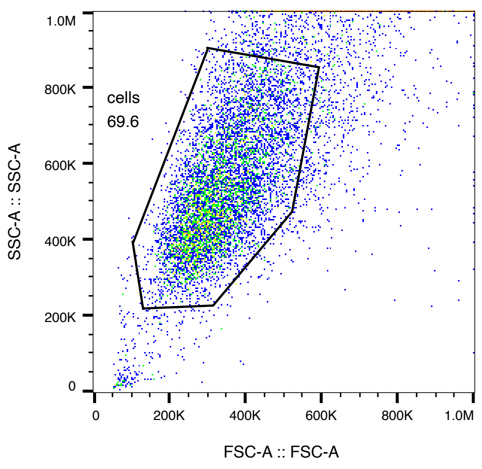

Torin_H_3_Data Source - 1.fcs
Ungated
10000

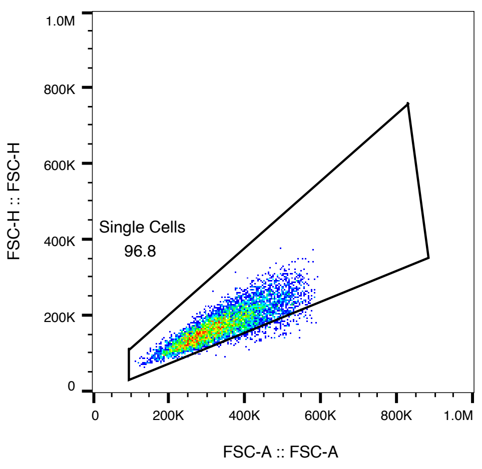

Torin_H_3_Data Source - 1.fcs
cells
6957

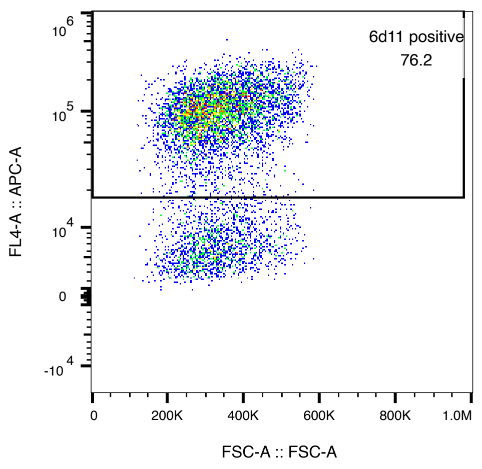

Torin_H_3_Data Source - 1.fcs
Single Cells
6735

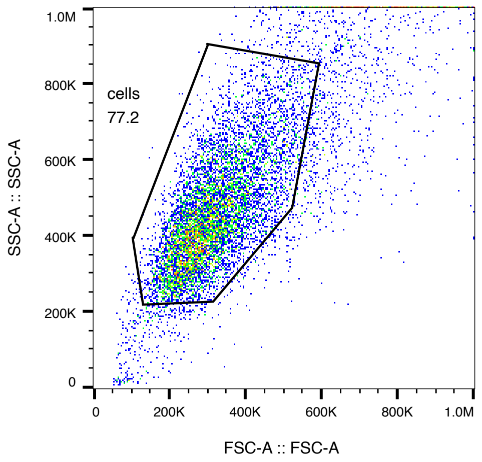

Rapa_NT_1_Data Source - 1.fcs
Ungated
10000

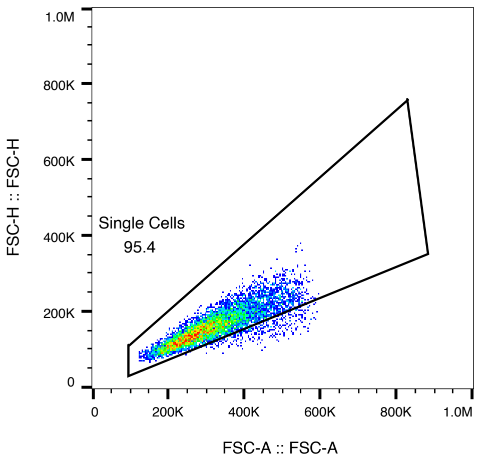

Rapa_NT_1_Data Source - 1.fcs
cells
7725

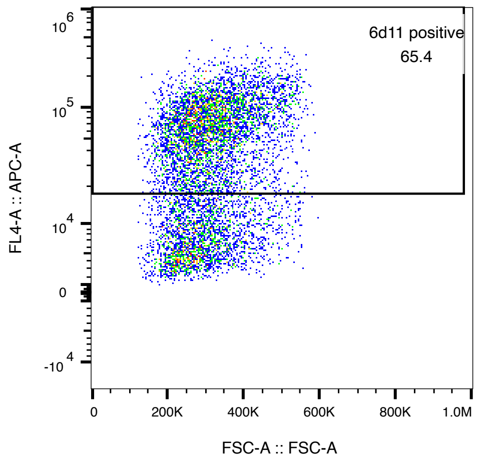

Rapa_NT_1_Data Source - 1.fcs
Single Cells
7370

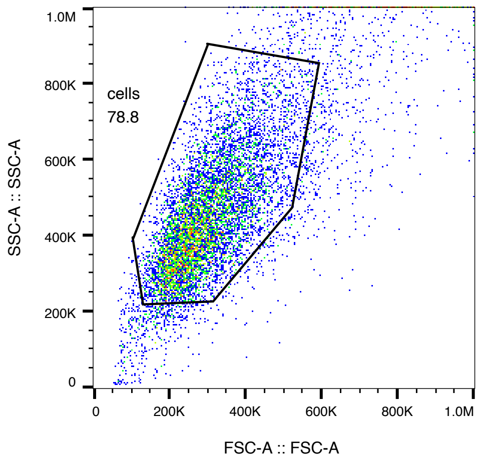

Rapa_NT_2_Data Source - 1.fcs
Ungated
10000

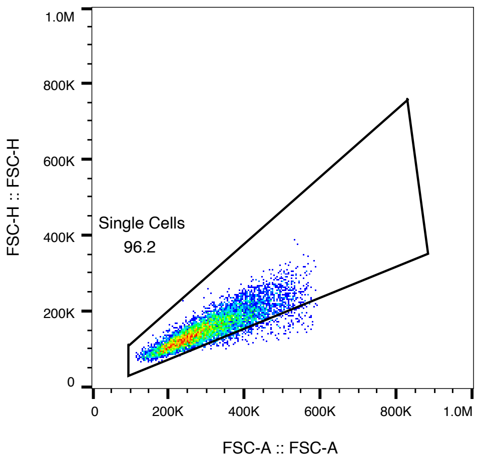

Rapa_NT_2_Data Source - 1.fcs
cells
7875

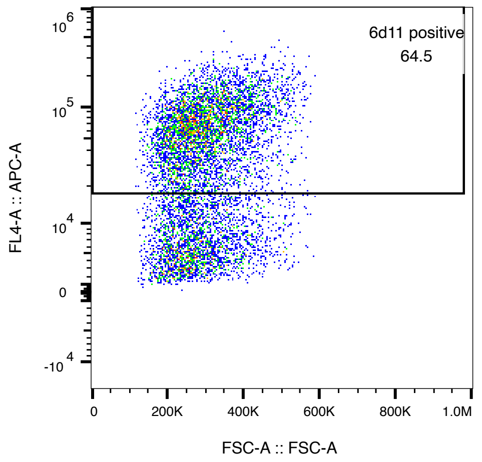

Rapa_NT_2_Data Source - 1.fcs
Single Cells
7572

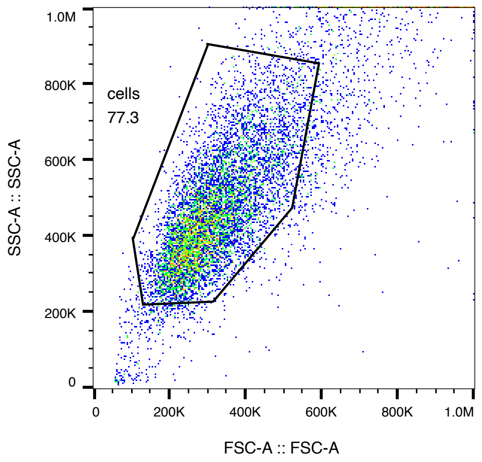

Rapa_NT_3_Data Source - 1.fcs
Ungated
10000

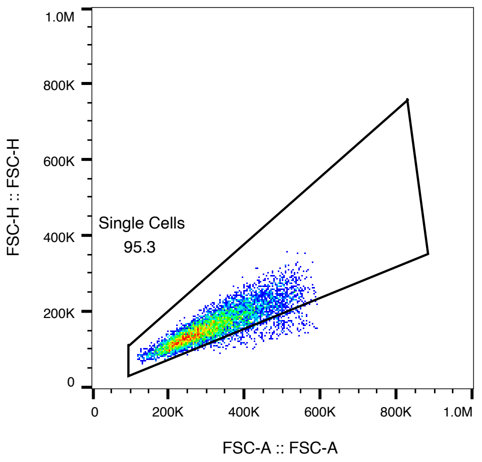

Rapa_NT_3_Data Source - 1.fcs
cells
7727

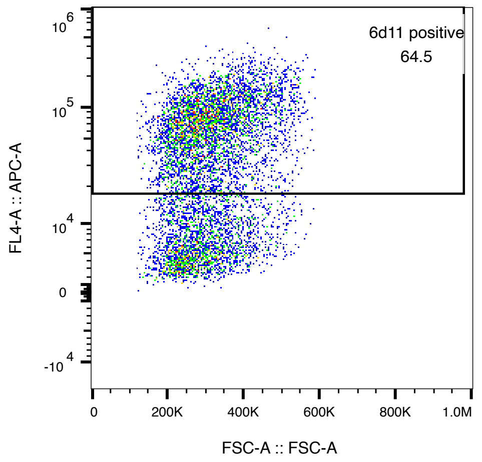

Rapa_NT_3_Data Source - 1.fcs
Single Cells
7364

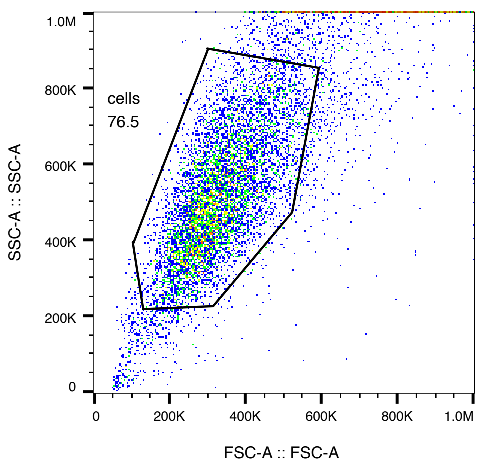

Rapa_H_1_Data Source - 1.fcs
Ungated
10000

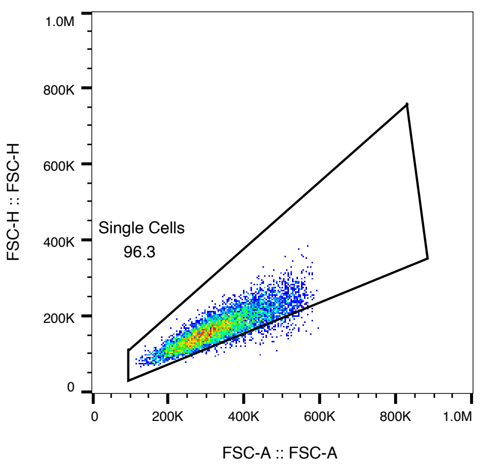

Rapa_H_1_Data Source - 1.fcs
cells
7651

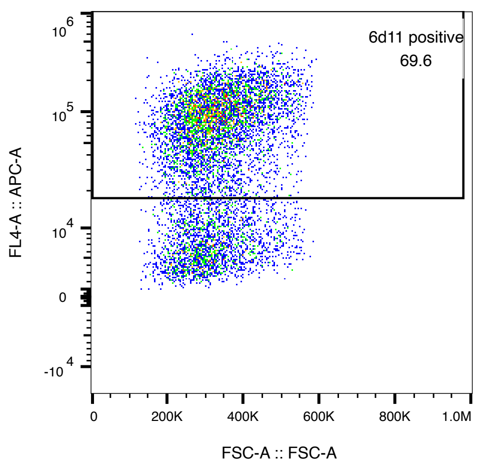

Rapa_H_1_Data Source - 1.fcs
Single Cells
7371

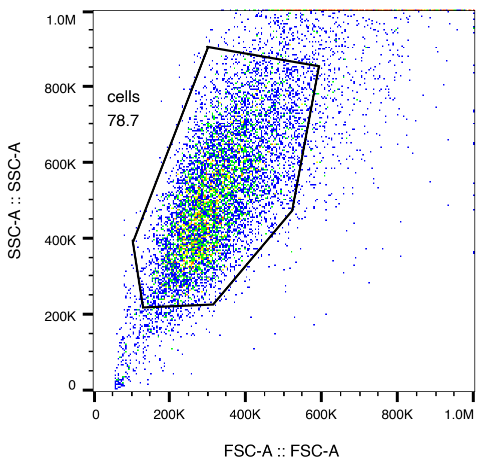

Rapa_H_2_Data Source - 1.fcs
Ungated
10000

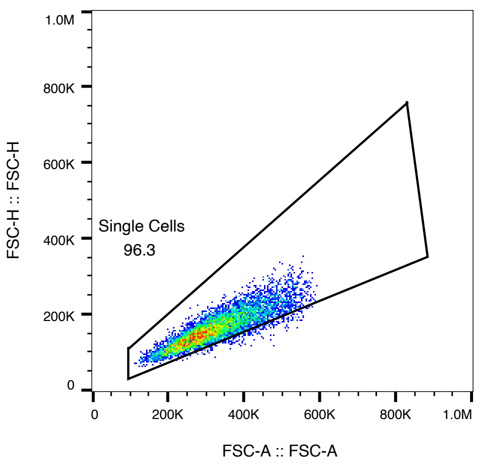

Rapa_H_2_Data Source - 1.fcs
cells
7870

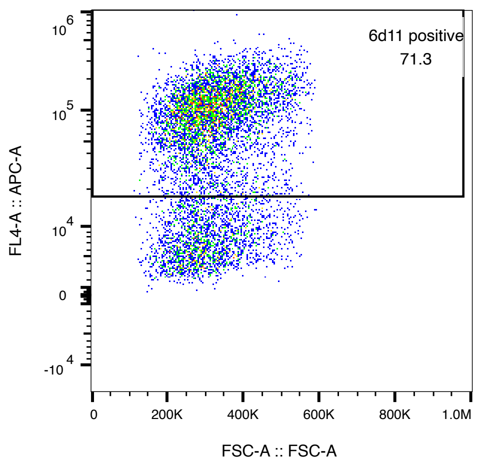

Rapa_H_2_Data Source - 1.fcs
Single Cells
7577

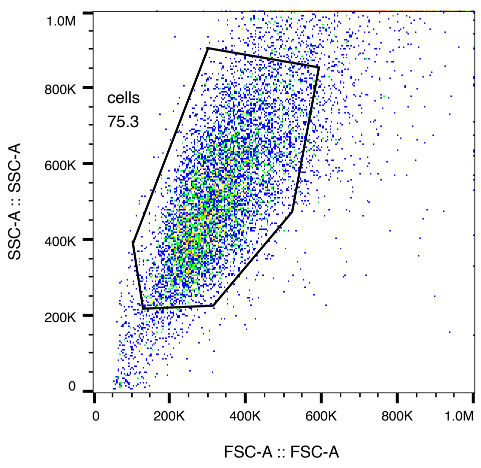

Rapa_H_3_Data Source - 1.fcs
Ungated
10000

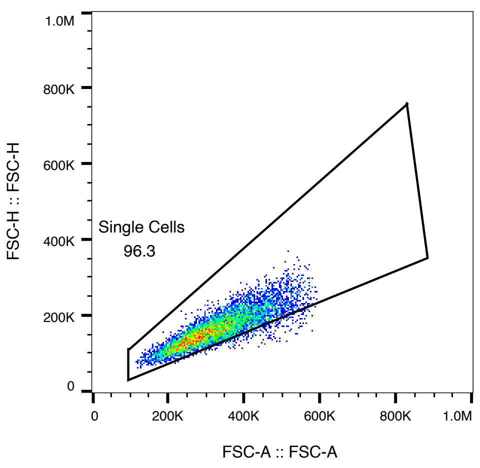

Rapa_H_3_Data Source - 1.fcs
cells
7532

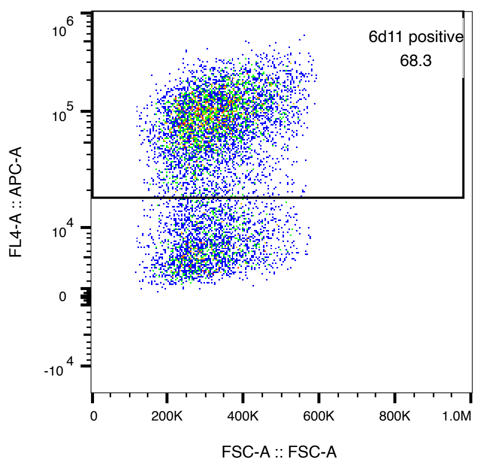

Rapa_H_3_Data Source - 1.fcs
Single Cells
7257

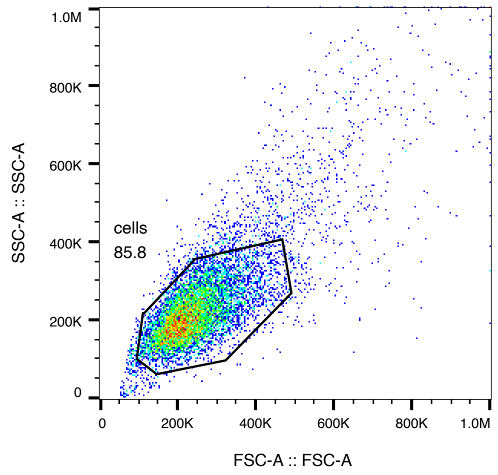

unstained_Data Source - 1.fcs
Ungated
10000

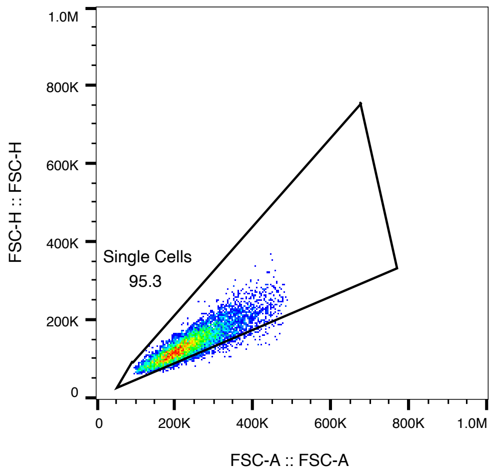

unstained_Data Source - 1.fcs
cells
8580

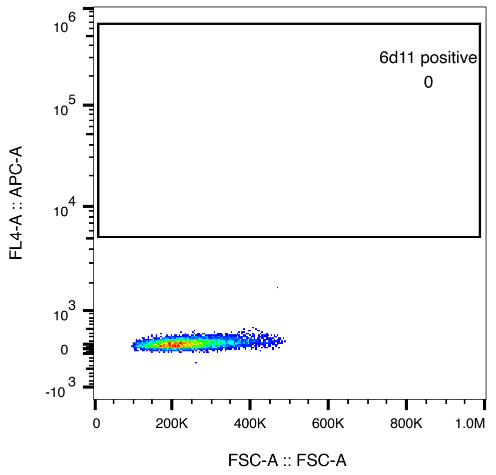

unstained_Data Source - 1.fcs
Single Cells
8181

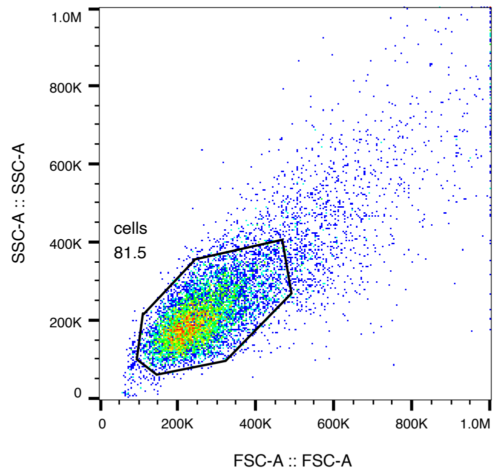

NBH _Data Source - 1.fcs
Ungated
10000

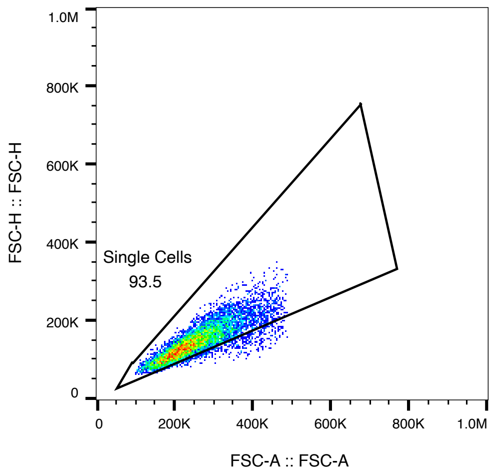

NBH _Data Source - 1.fcs
cells
8149

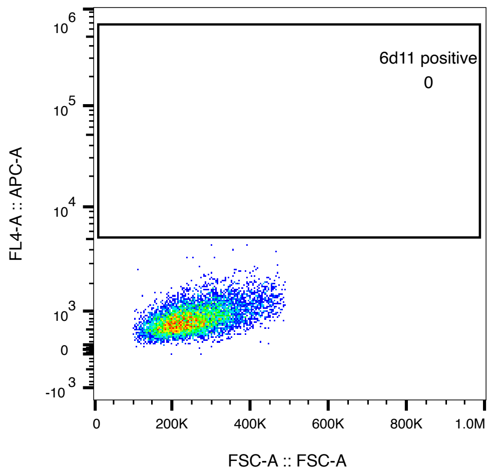

NBH _Data Source - 1.fcs
Single Cells
7623

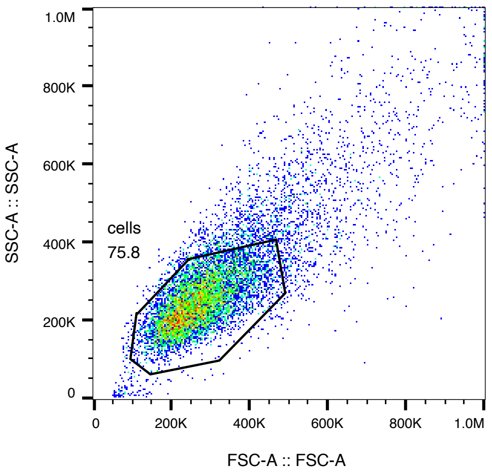

DMSO_NT_1_Data Source - 1.fcs
Ungated
10000

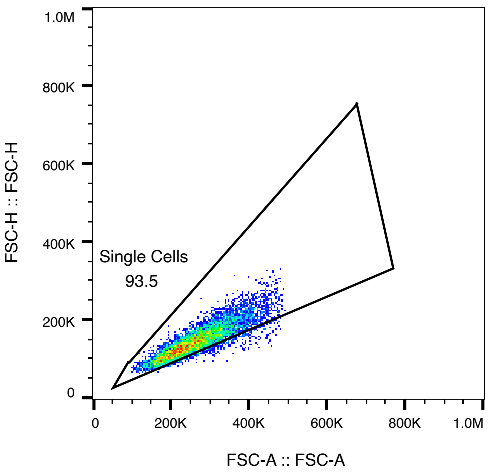

DMSO_NT_1_Data Source - 1.fcs
cells
7579

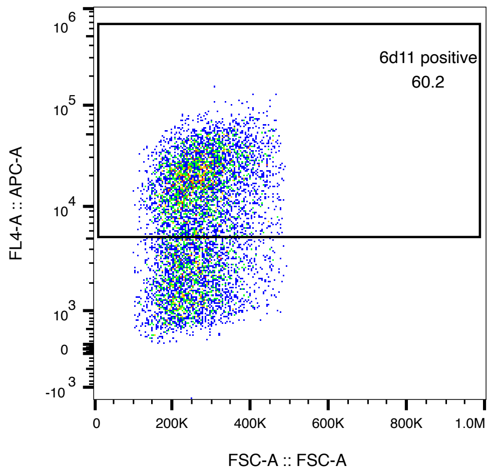

DMSO_NT_1_Data Source - 1.fcs
Single Cells
7088

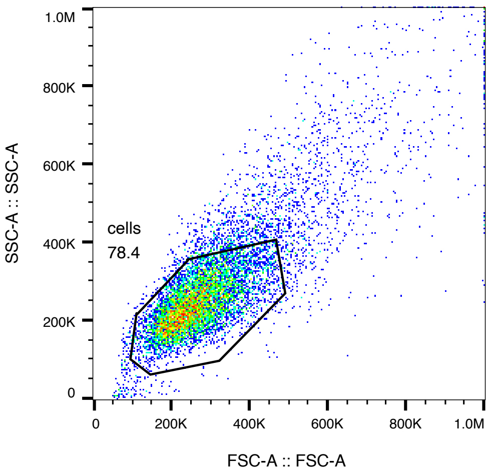

DMSO_NT_2_Data Source - 1.fcs
Ungated
10000

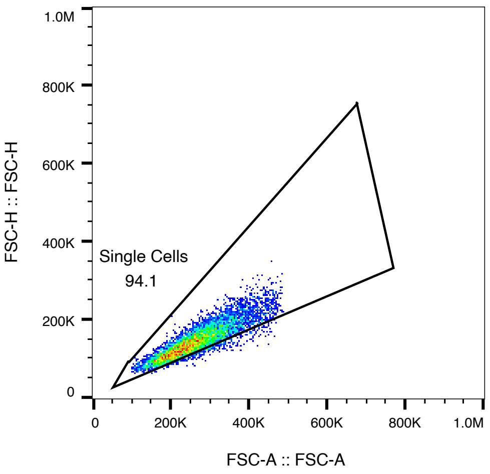

DMSO_NT_2_Data Source - 1.fcs
cells
7836

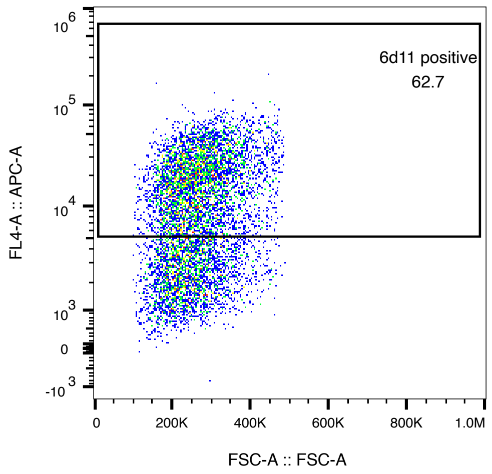

DMSO_NT_2_Data Source - 1.fcs
Single Cells
7375

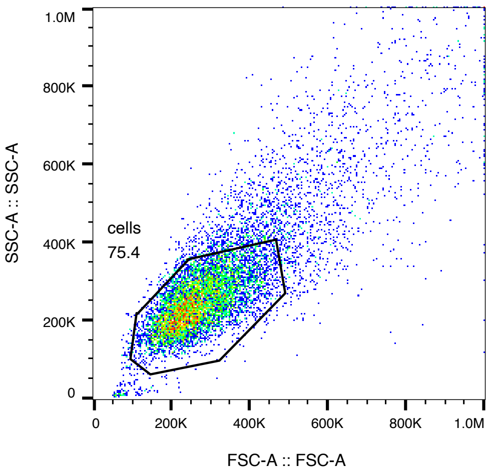

DMSO_NT_3_Data Source - 1.fcs
Ungated
10000

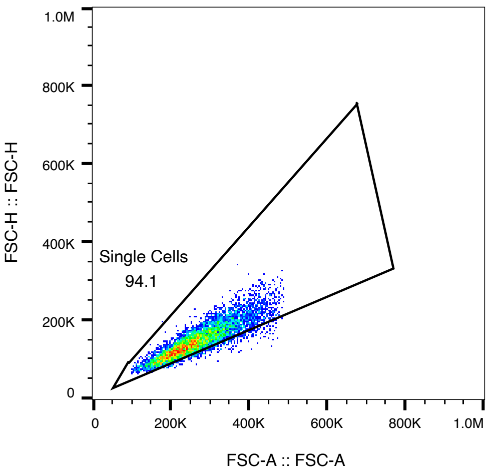

DMSO_NT_3_Data Source - 1.fcs
cells
7536

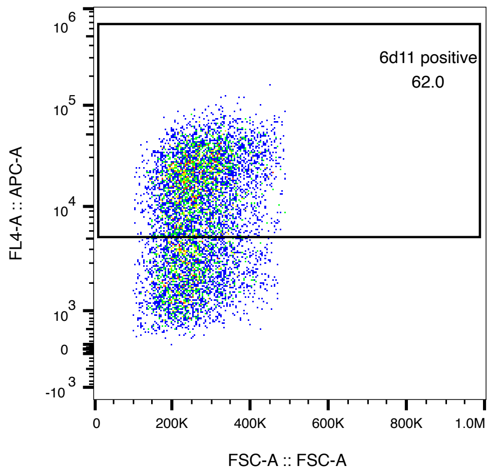

DMSO_NT_3_Data Source - 1.fcs
Single Cells
7092

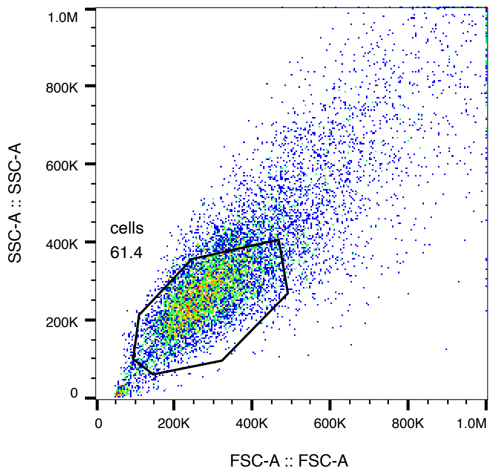

DMSO_H_1_Data Source - 1.fcs
Ungated
10000

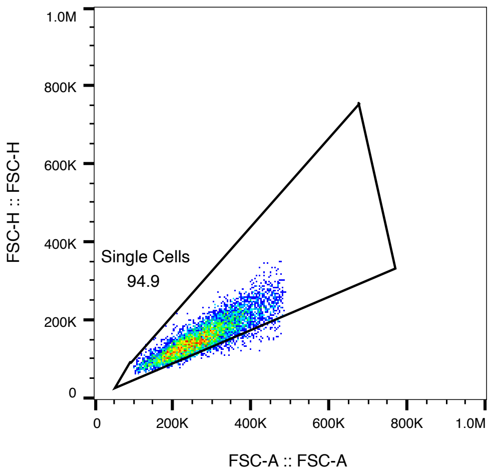

DMSO_H_1_Data Source - 1.fcs
cells
6138

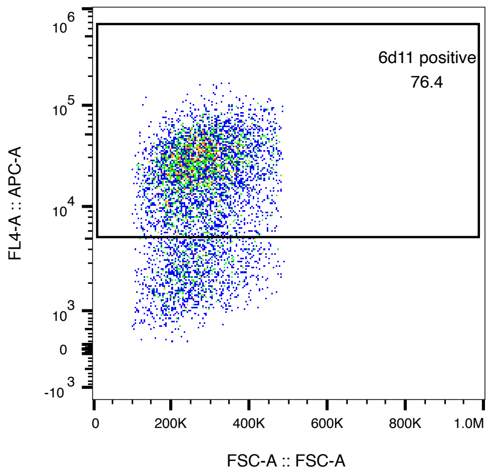

DMSO_H_1_Data Source - 1.fcs
Single Cells
5827

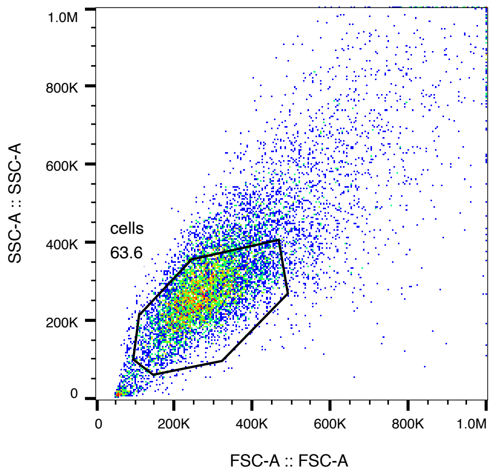

DMSO_H_2_Data Source - 1.fcs
Ungated
10000

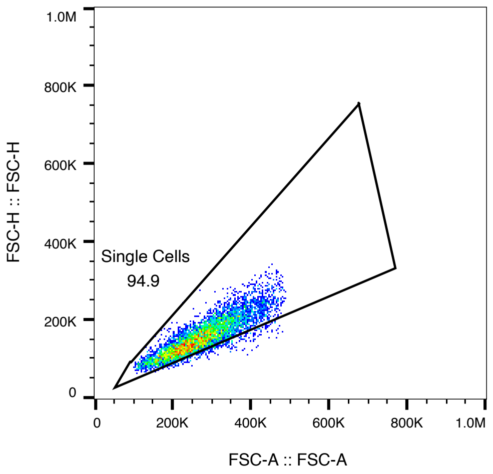

DMSO_H_2_Data Source - 1.fcs
cells
6362

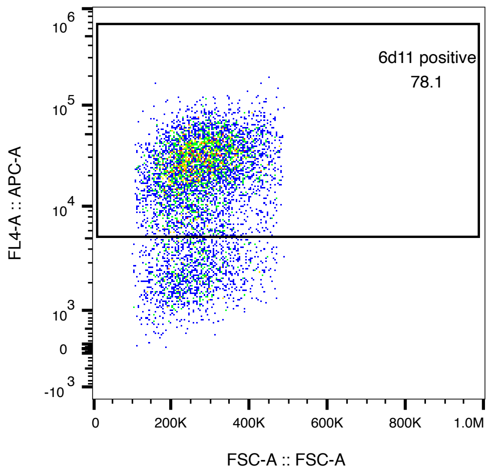

DMSO_H_2_Data Source - 1.fcs
Single Cells
6036

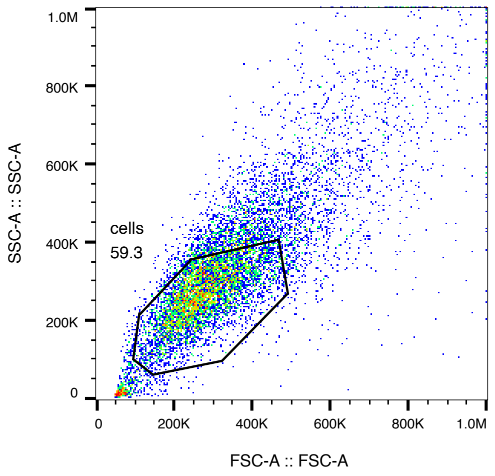

DMSO_H_3_Data Source - 1.fcs
Ungated
10000

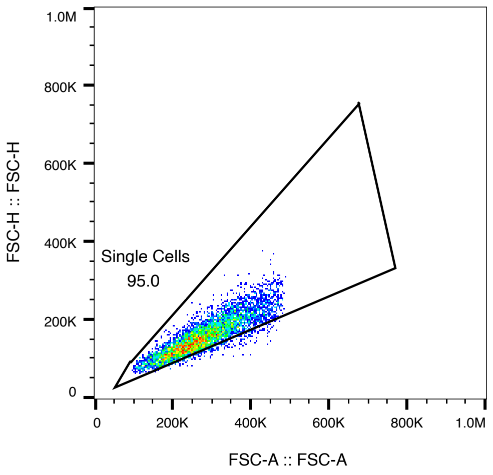

DMSO_H_3_Data Source - 1.fcs
cells
5928

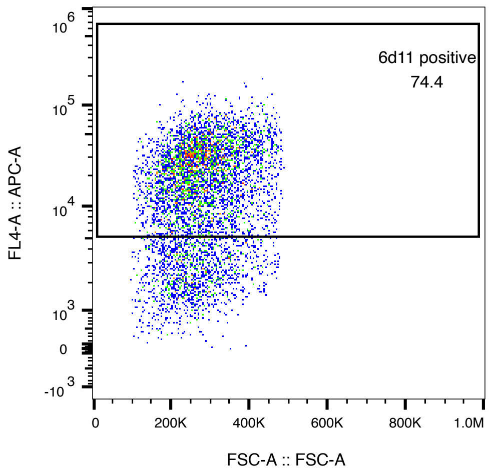

DMSO_H_3_Data Source - 1.fcs
Single Cells
5631

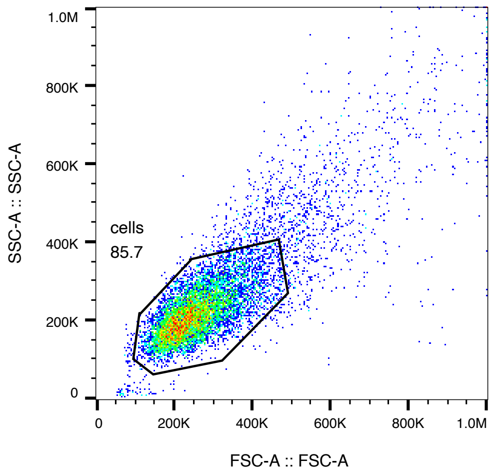

Torin_NT_1_Data Source - 1.fcs
Ungated
10000

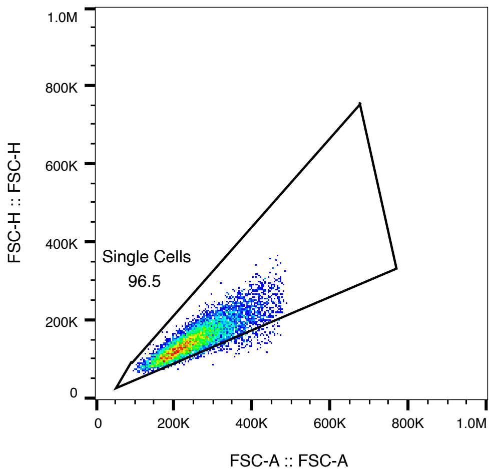

Torin_NT_1_Data Source - 1.fcs
cells
8574

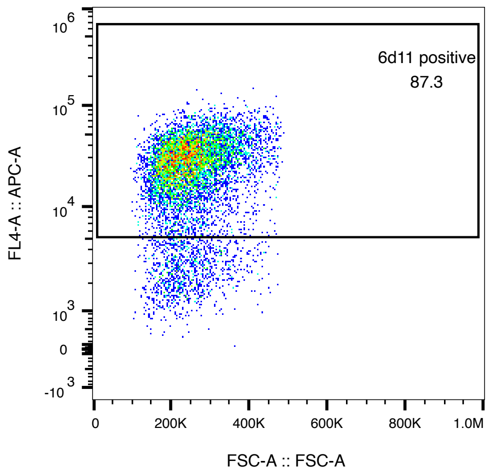

Torin_NT_1_Data Source - 1.fcs
Single Cells
8276

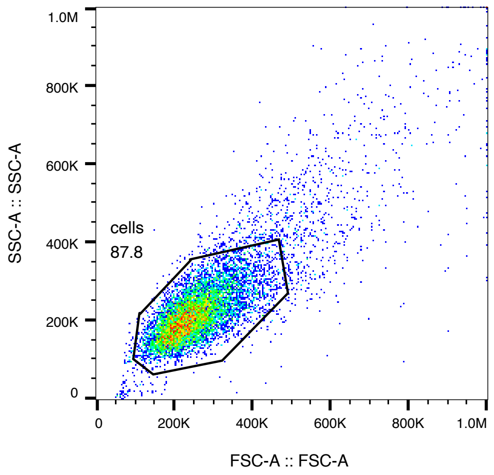

Torin_NT_2_Data Source - 1.fcs
Ungated
10000

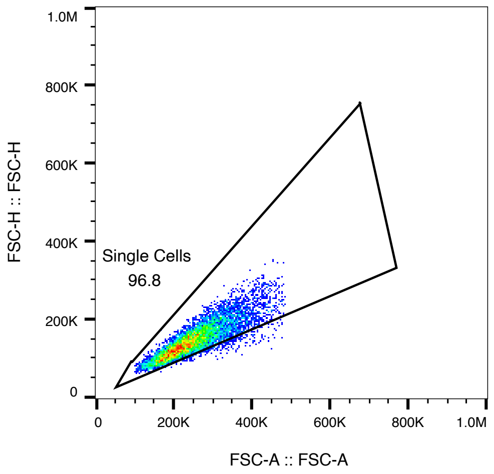

Torin_NT_2_Data Source - 1.fcs
cells
8785

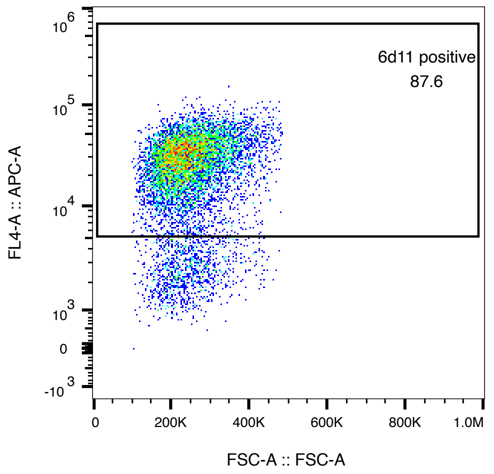

Torin_NT_2_Data Source - 1.fcs
Single Cells
8501

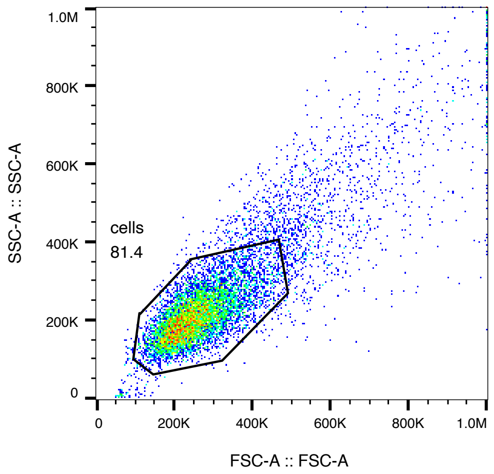

Torin_NT_3_Data Source - 1.fcs
Ungated
10000

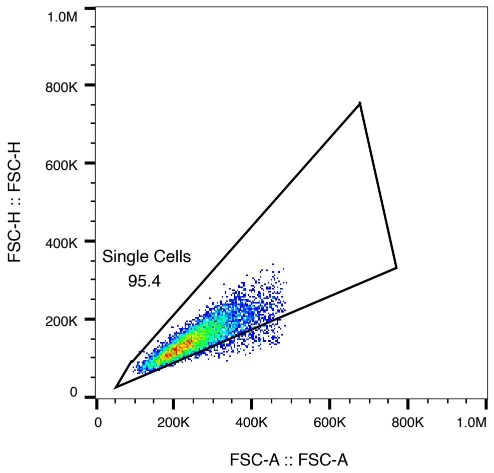

Torin_NT_3_Data Source - 1.fcs
cells
8138

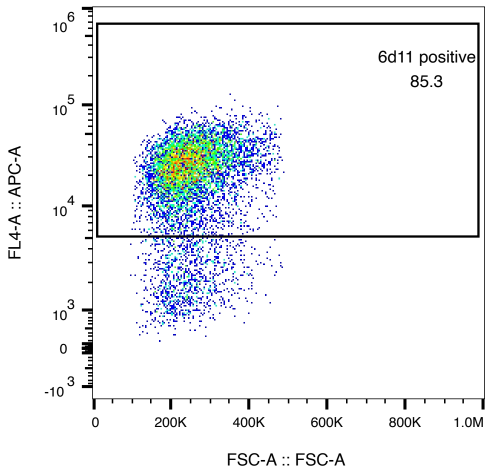

Torin_NT_3_Data Source - 1.fcs
Single Cells
7765

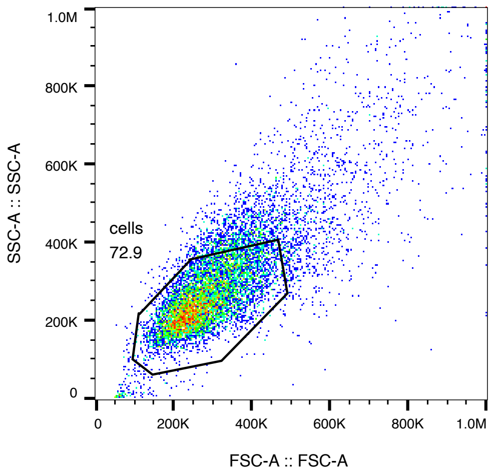

Torin_H_1_Data Source - 1.fcs
Ungated
10000

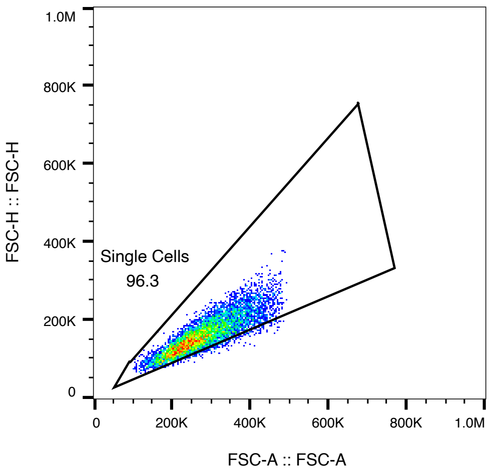

Torin_H_1_Data Source - 1.fcs
cells
7294

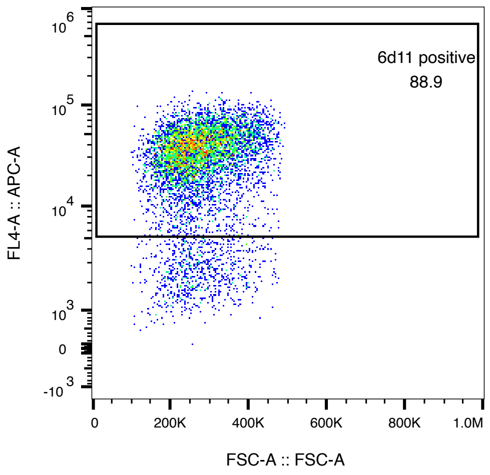

Torin_H_1_Data Source - 1.fcs
Single Cells
7023

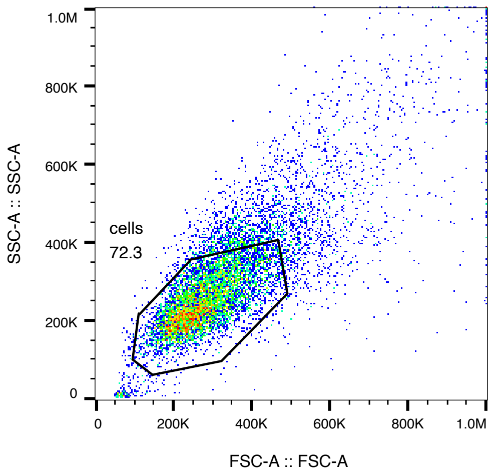

Torin_H_2_Data Source - 1.fcs
Ungated
10000

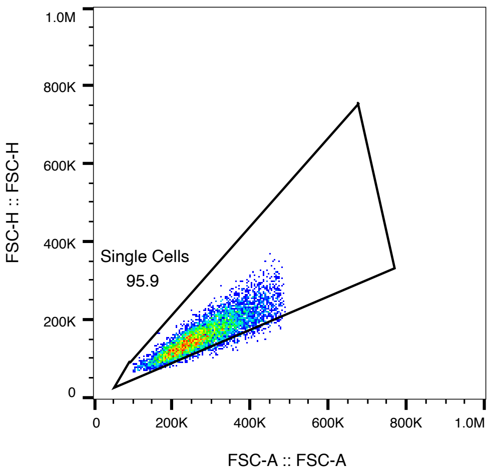

Torin_H_2_Data Source - 1.fcs
cells
7231

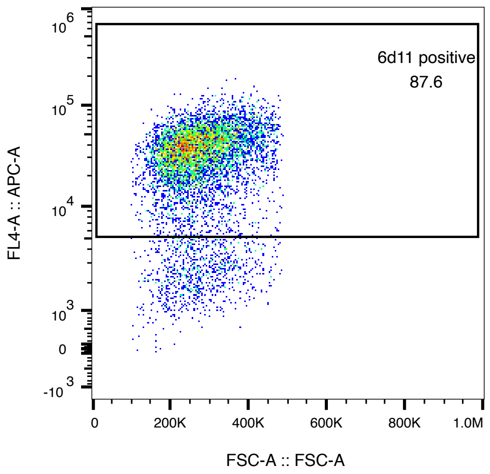

Torin_H_2_Data Source - 1.fcs
Single Cells
6935

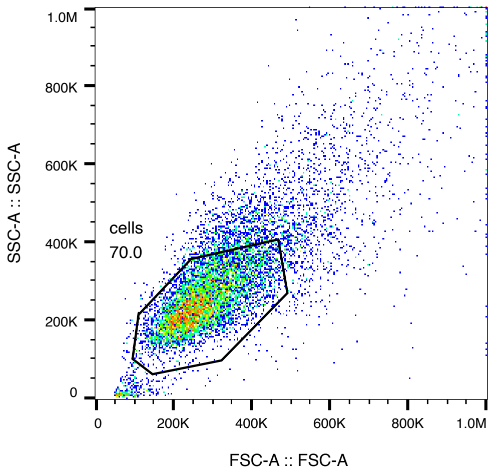

Torin_H_3_Data Source - 1.fcs
Ungated
10000

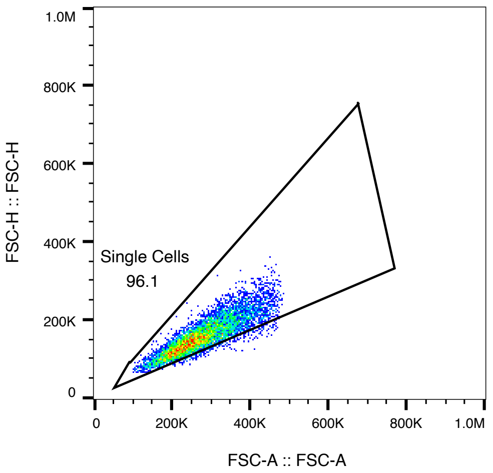

Torin_H_3_Data Source - 1.fcs
cells
7000

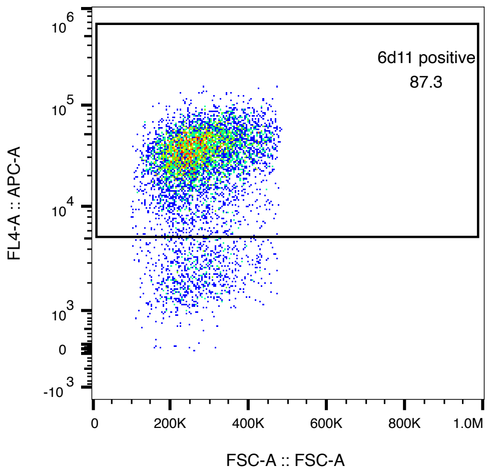

Torin_H_3_Data Source - 1.fcs
Single Cells
6729

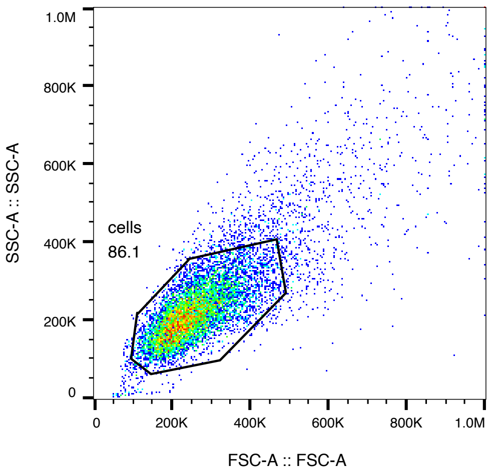

Rapa_NT_1_Data Source - 1.fcs
Ungated
10000

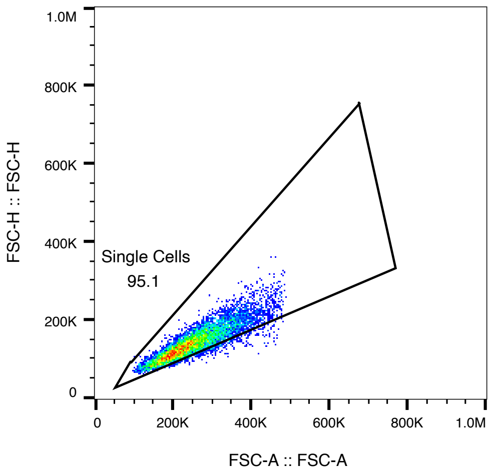

Rapa_NT_1_Data Source - 1.fcs
cells
8608

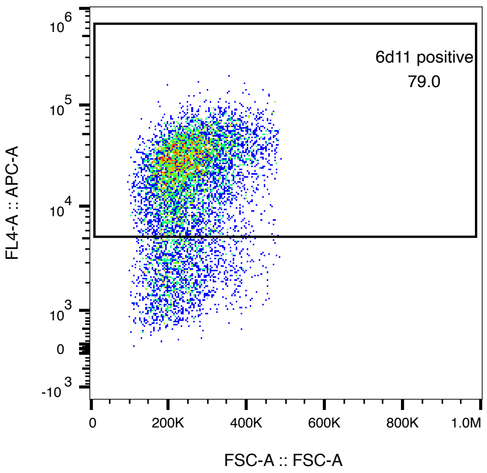

Rapa_NT_1_Data Source - 1.fcs
Single Cells
8189

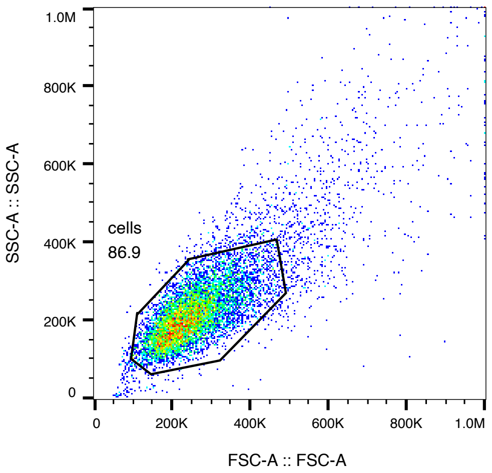

Rapa_NT_2_Data Source - 1.fcs
Ungated
10000

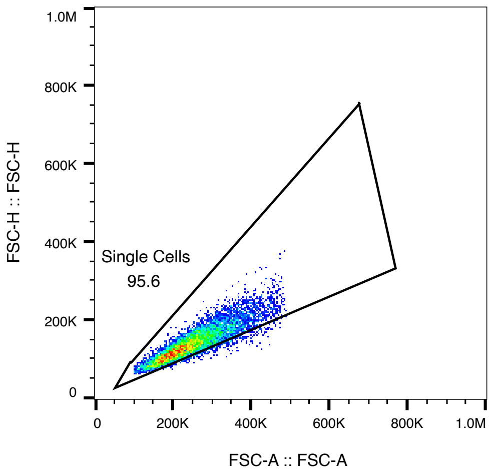

Rapa_NT_2_Data Source - 1.fcs
cells
8690

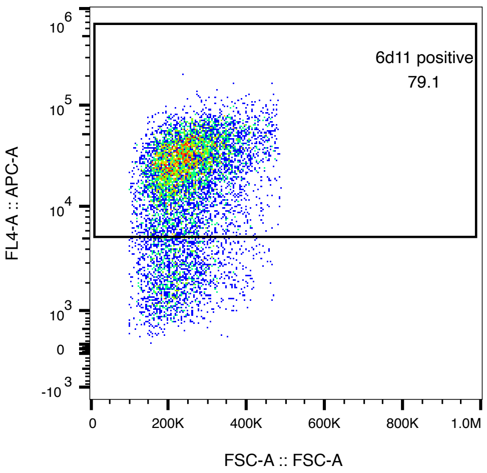

Rapa_NT_2_Data Source - 1.fcs
Single Cells
8304

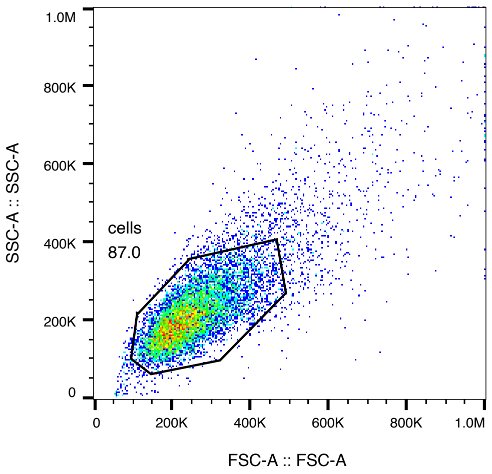

Rapa_NT_3_Data Source - 1.fcs
Ungated
10000

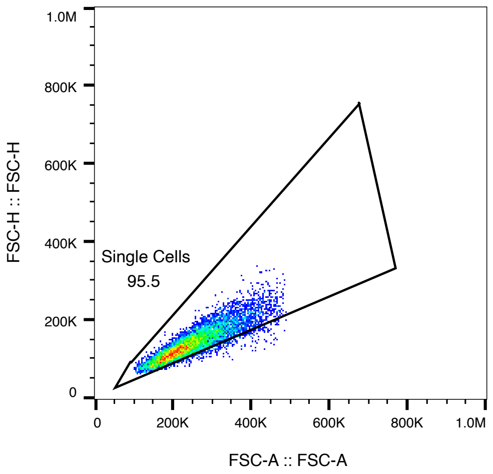

Rapa_NT_3_Data Source - 1.fcs
cells
8699

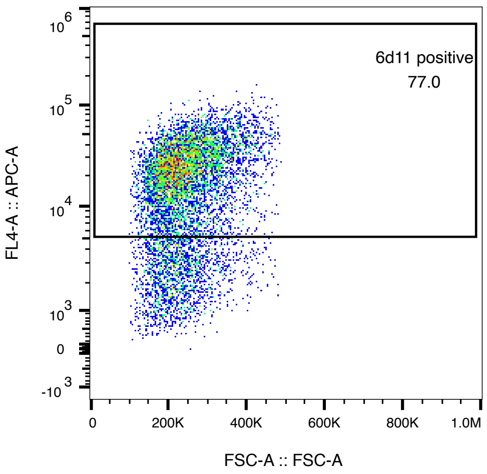

Rapa_NT_3_Data Source - 1.fcs
Single Cells
8308

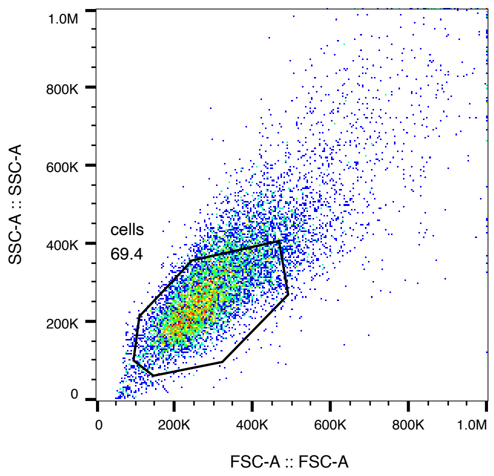

Rapa_H_1_Data Source - 1.fcs
Ungated
10000

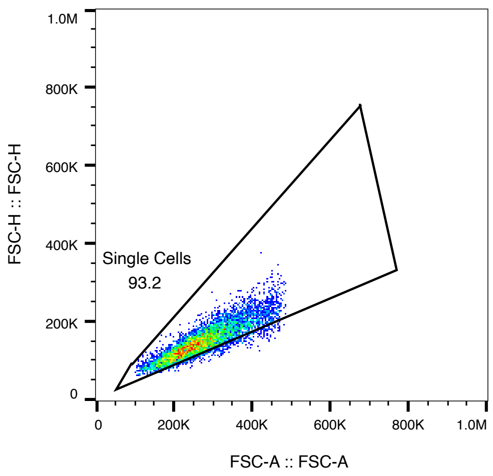

Rapa_H_1_Data Source - 1.fcs
cells
6938

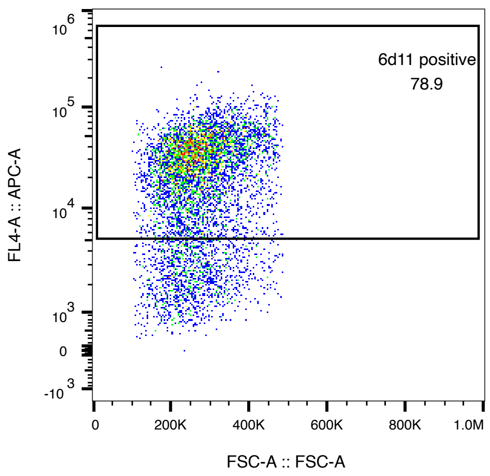

Rapa_H_1_Data Source - 1.fcs
Single Cells
6468

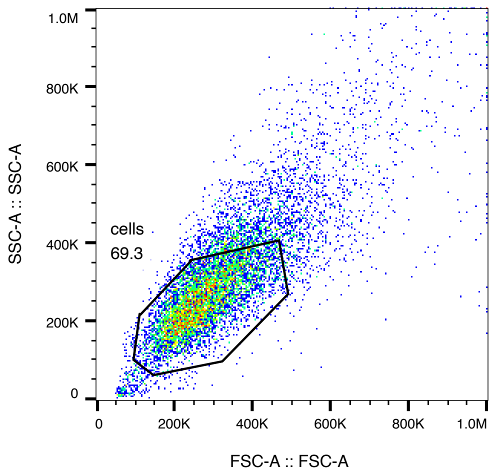

Rapa_H_2_Data Source - 1.fcs
Ungated
10000

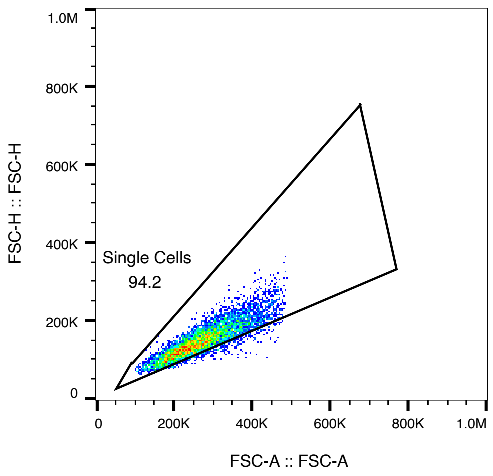

Rapa_H_2_Data Source - 1.fcs
cells
6932

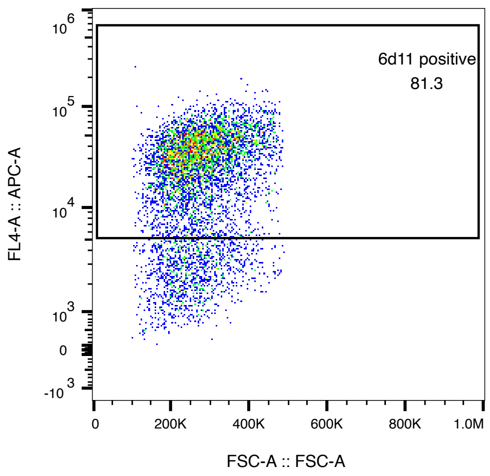

Rapa_H_2_Data Source - 1.fcs
Single Cells
6527

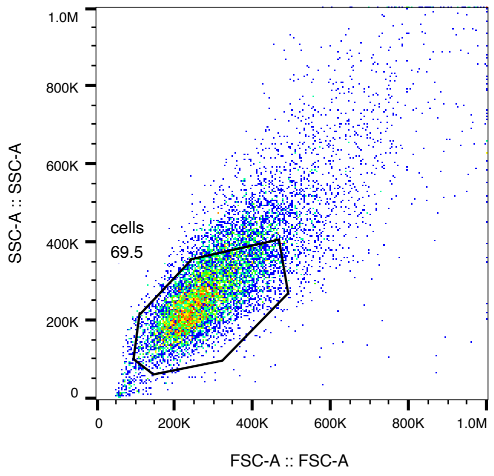

Rapa_H_3_Data Source - 1.fcs
Ungated
10000

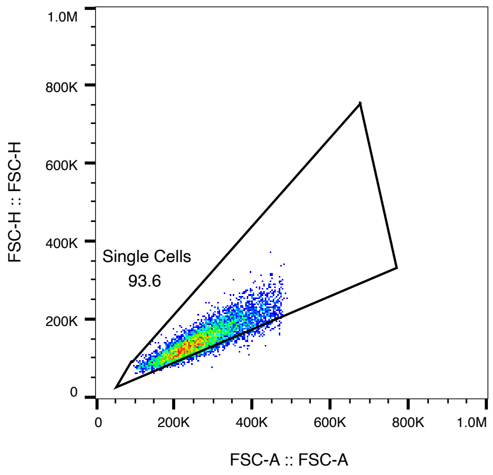

Rapa_H_3_Data Source - 1.fcs
cells
6946

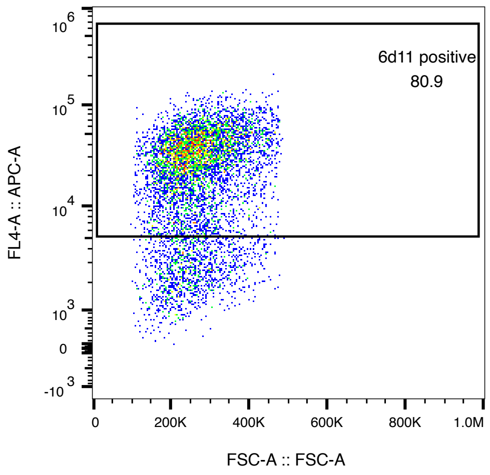

Rapa_H_3_Data Source - 1.fcs
Single Cells
6500

S1B Fig

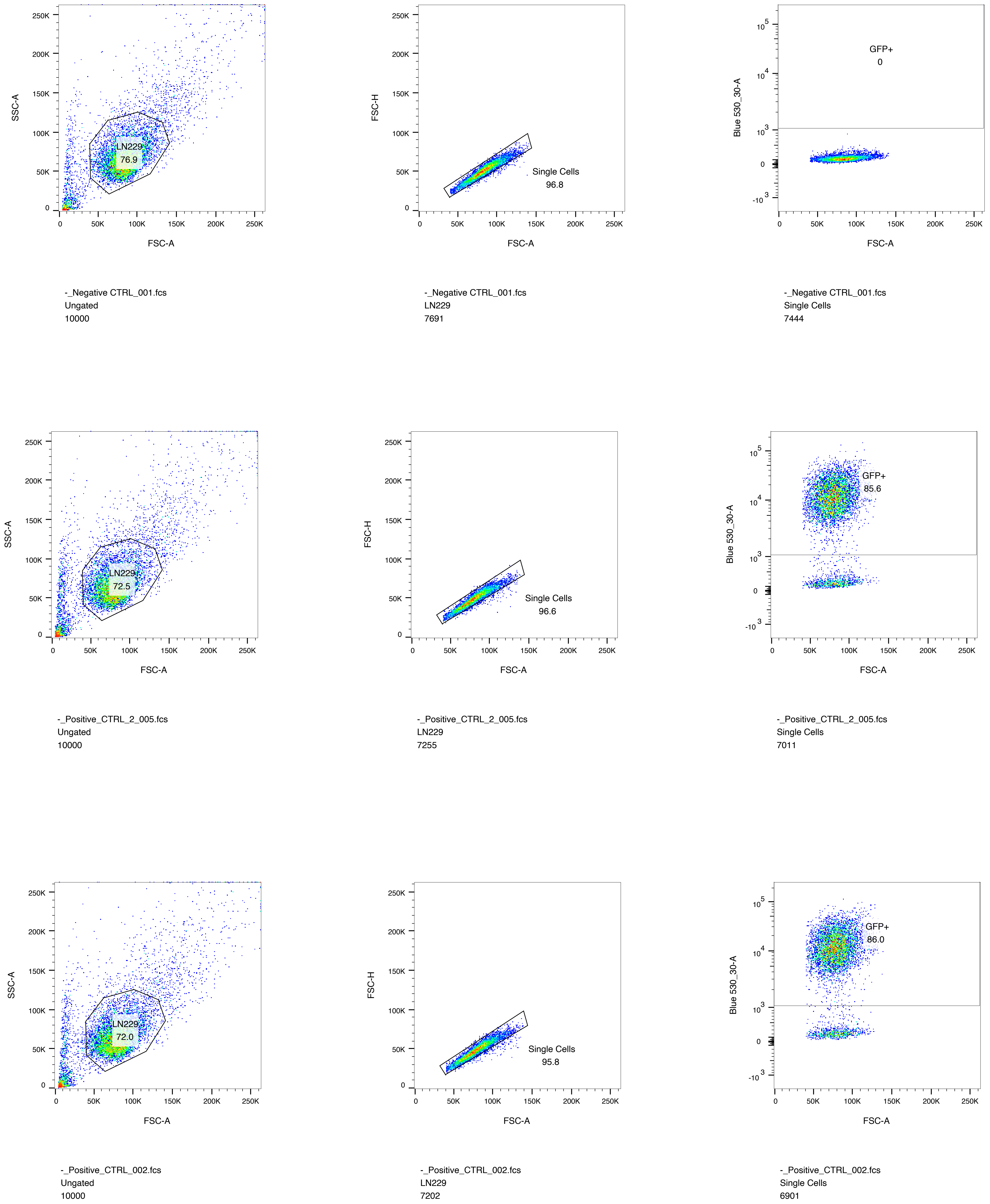
SSC-A
FSC-A
LN229
76.9
-_Negative CTRL_001.fcs
Ungated
10000
FSC-H
FSC-A
Single Cells
96.8
-_Negative CTRL_001.fcs
LN229
7691
Blue 530_30-A
FSC-A
GFP+
0
-_Negative CTRL_001.fcs
Single Cells
7444
SSC-A
FSC-A
LN229
72.5
-_Positive_CTRL_2_005.fcs
Ungated
10000
FSC-H
FSC-A
Single Cells
96.6
-_Positive_CTRL_2_005.fcs
LN229
7255
Blue 530_30-A
FSC-A
GFP+
85.6
-_Positive_CTRL_2_005.fcs
Single Cells
7011
SSC-A
FSC-A
LN229
72.0
-_Positive_CTRL_002.fcs
Ungated
10000
FSC-H
FSC-A
Single Cells
95.8
-_Positive_CTRL_002.fcs
LN229
7202
Blue 530_30-A
FSC-A
GFP+
86.0
-_Positive_CTRL_002.fcs
Single Cells
6901

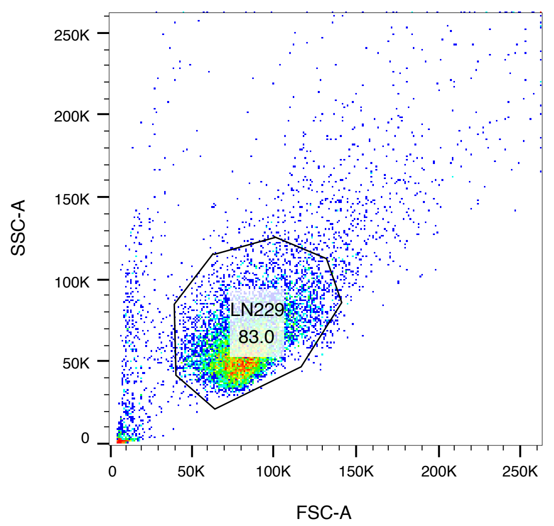

-_A9_2_017.fcs
Ungated
10000

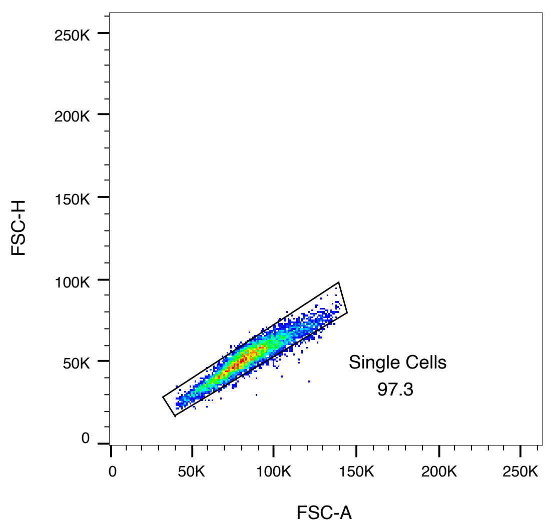

-_A9_2_017.fcs
LN229
8303

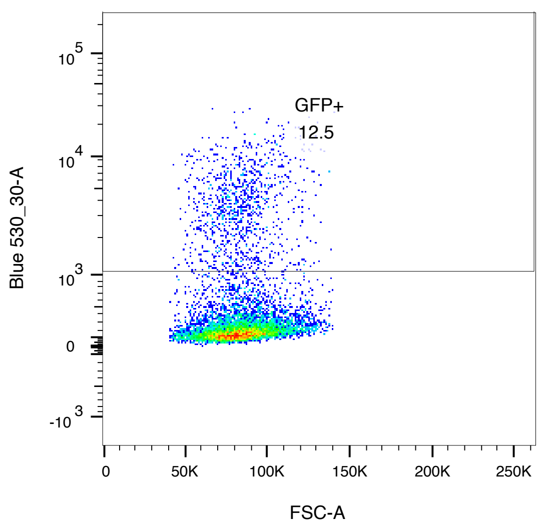

-_A9_2_017.fcs
Single Cells
8080

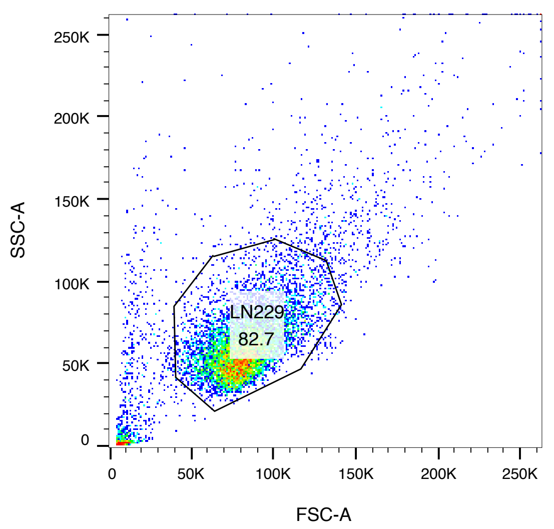

-_A9_016.fcs
Ungated
10000

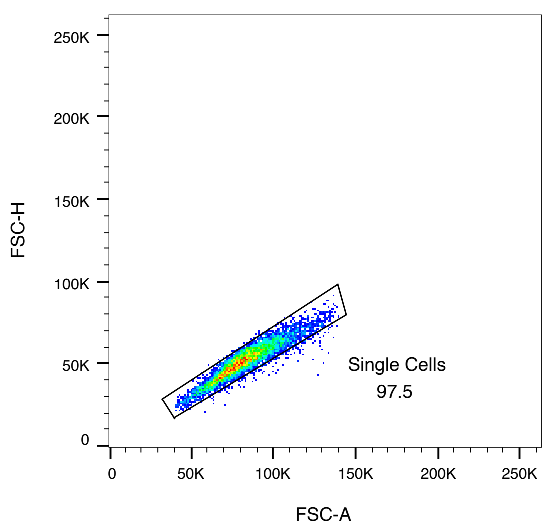

-_A9_016.fcs
LN229
8274

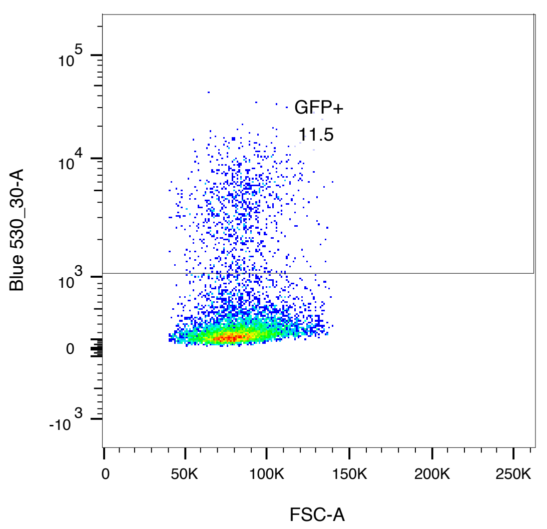

-_A9_016.fcs
Single Cells
8069

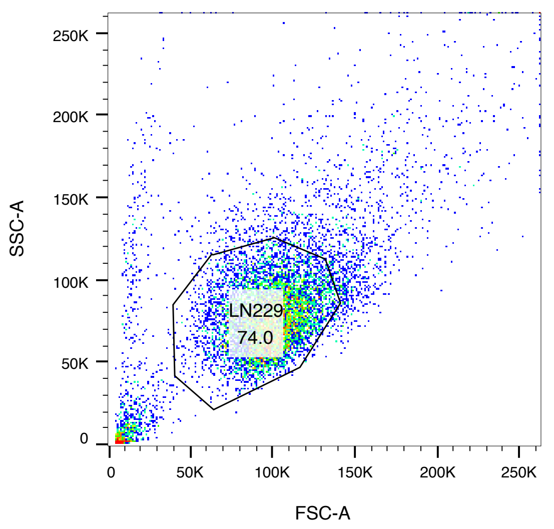

-_A10_2_007.fcs
Ungated
10000

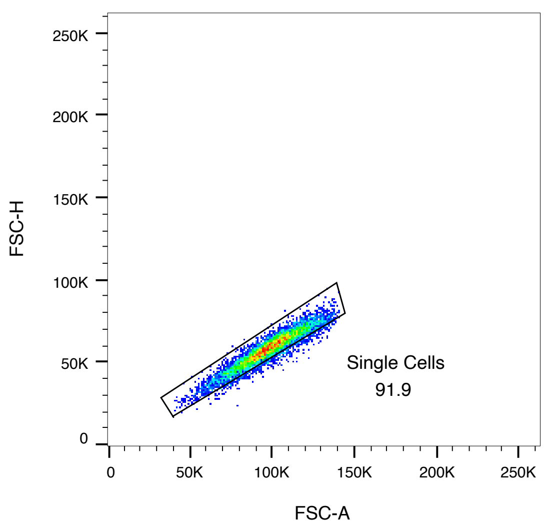

-_A10_2_007.fcs
LN229
7395

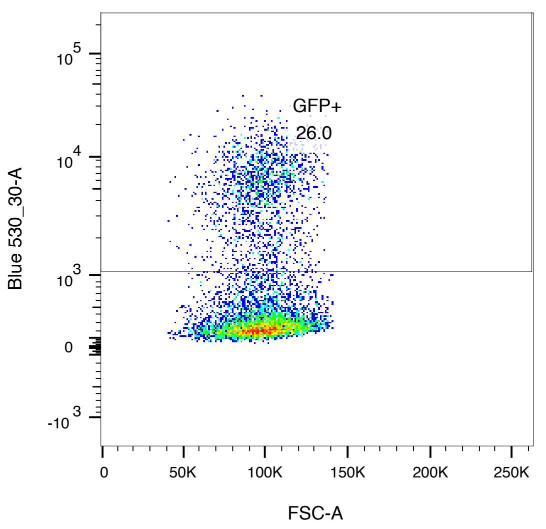

-_A10_2_007.fcs
Single Cells
6799

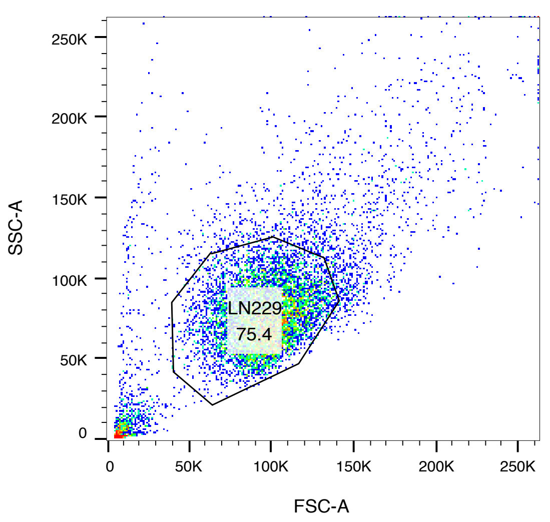

-_A10_006.fcs
Ungated
10000

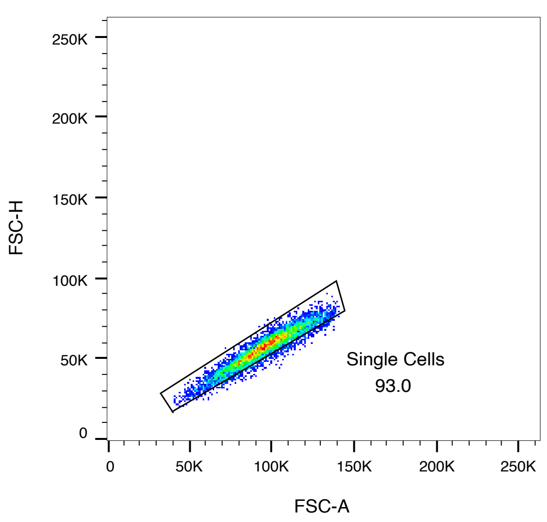

-_A10_006.fcs
LN229
7544

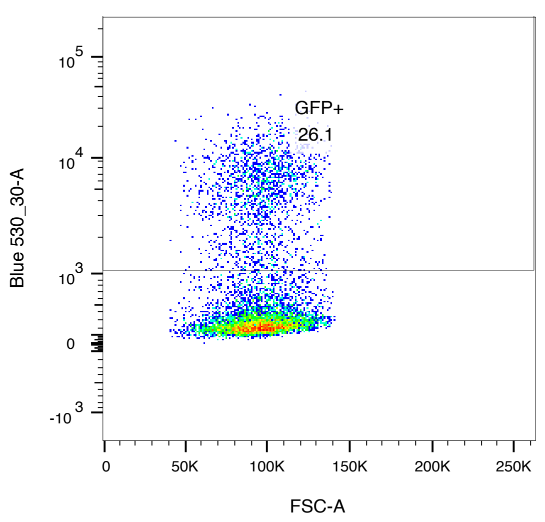

-_A10_006.fcs
Single Cells
7014

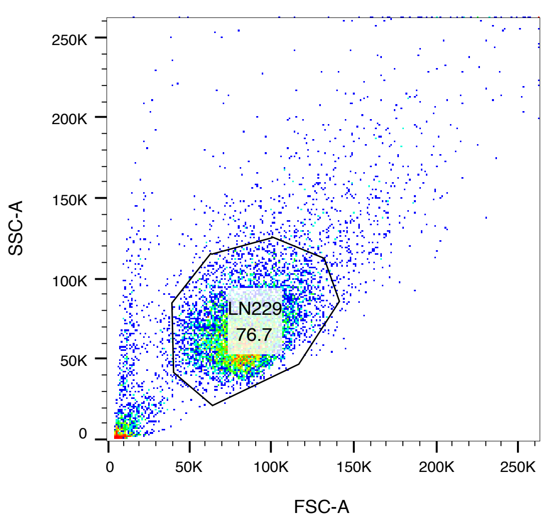

-_Polyclonal Cas9+_2_019.fcs
Ungated
10000

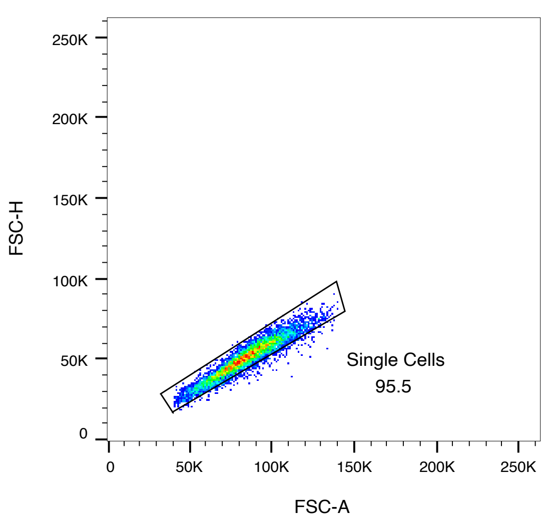

-_Polyclonal Cas9+_2_019.fcs
LN229
7674

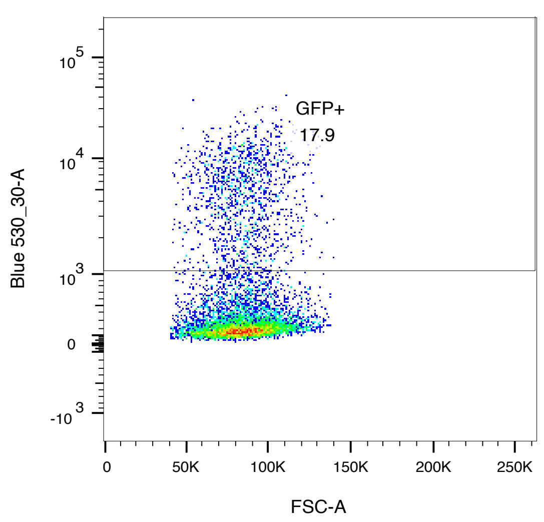

-_Polyclonal Cas9+_2_019.fcs
Single Cells
7329

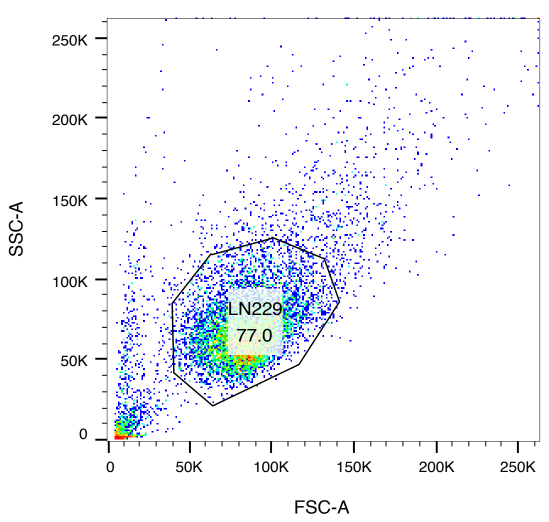

-_Polyclonal Cas9+_018.fcs
Ungated
10000

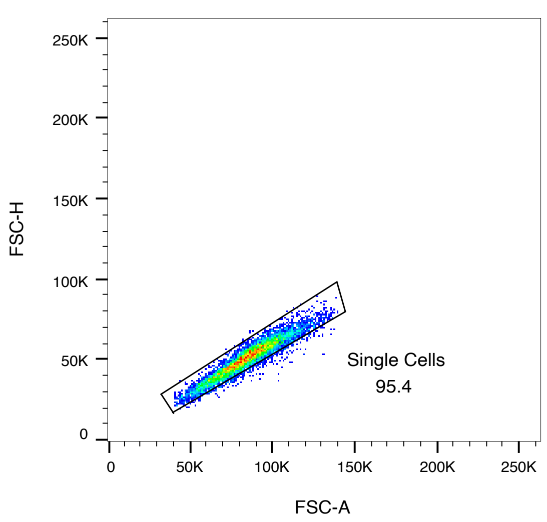

-_Polyclonal Cas9+_018.fcs
LN229
7696

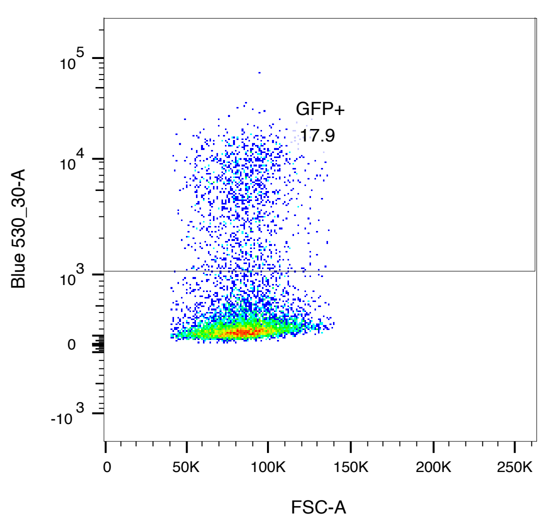

-_Polyclonal Cas9+_018.fcs
Single Cells
7342

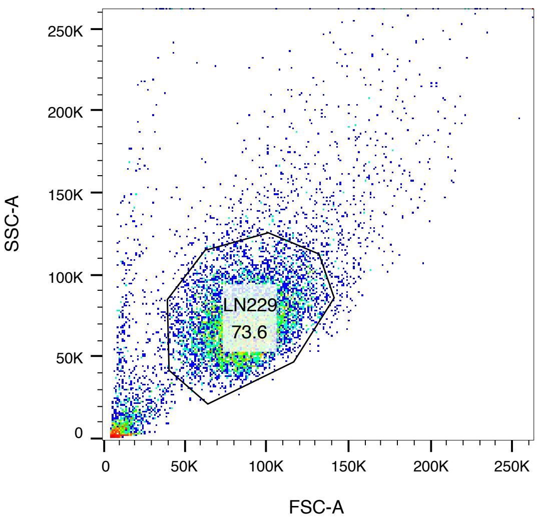

-_G11_2_013.fcs
Ungated
10000

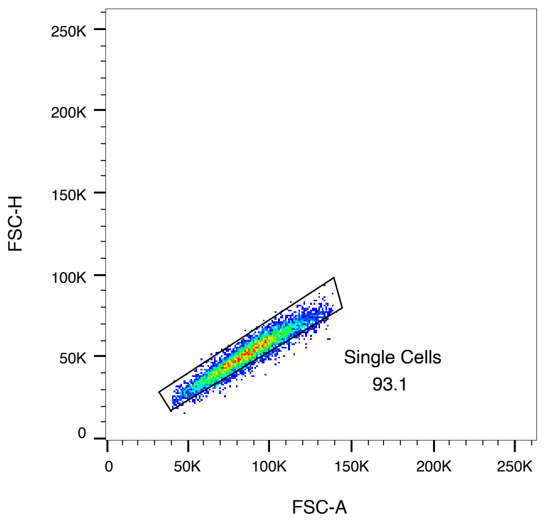

-_G11_2_013.fcs
LN229
7363

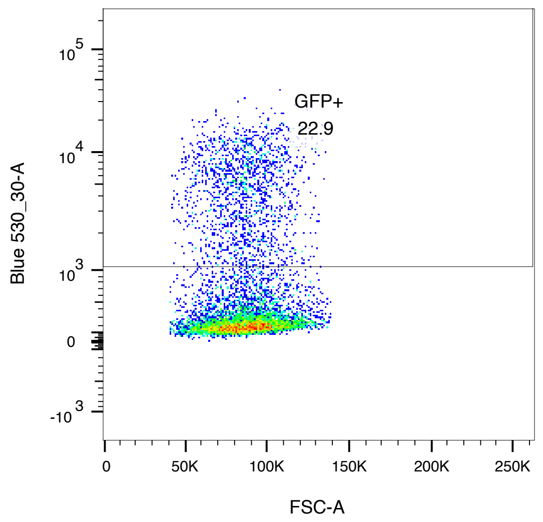

-_G11_2_013.fcs
Single Cells
6856

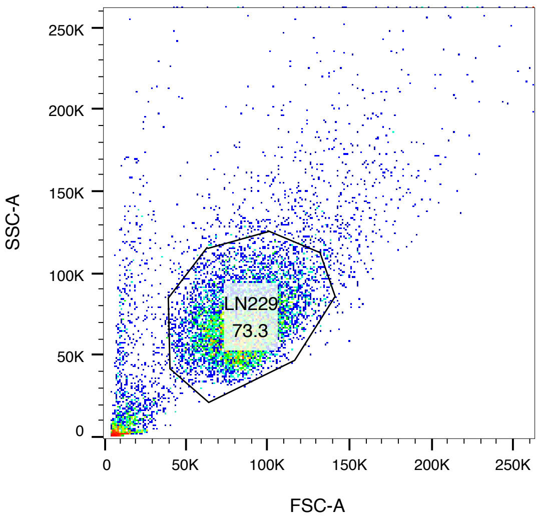

-_G11_012.fcs
Ungated
10000

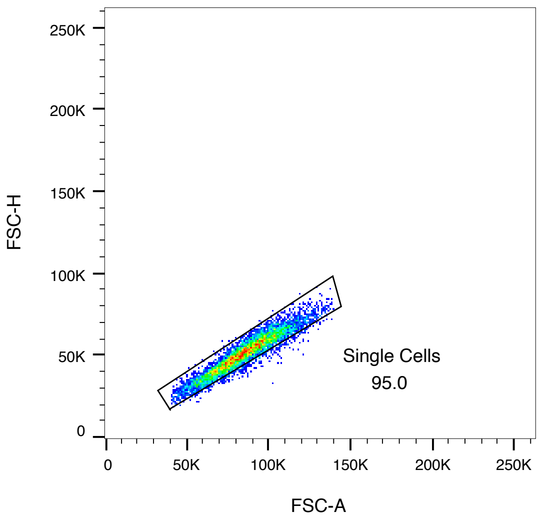

-_G11_012.fcs
LN229
7328

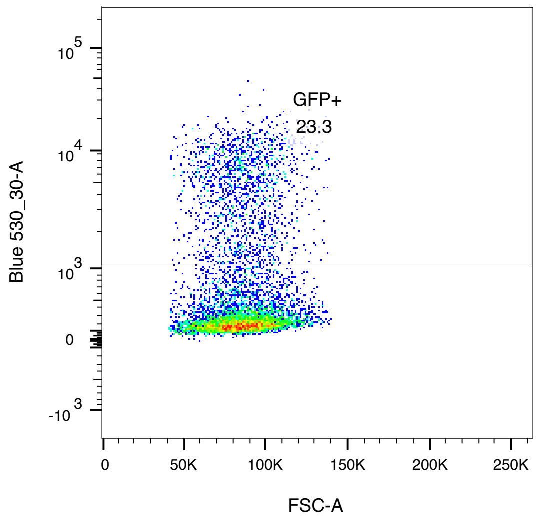

-_G11_012.fcs
Single Cells
6964

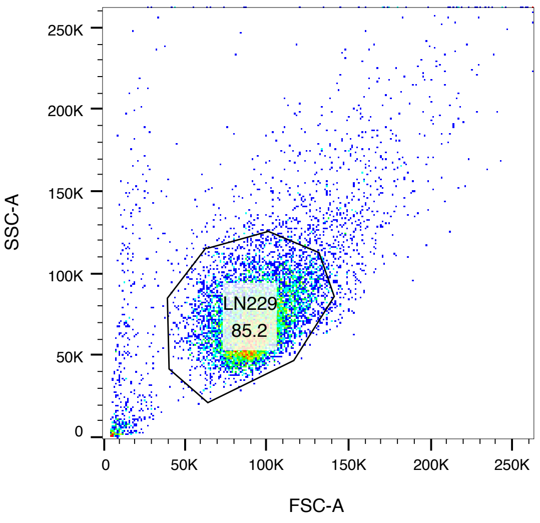

-_D7_2_004.fcs
Ungated
10000

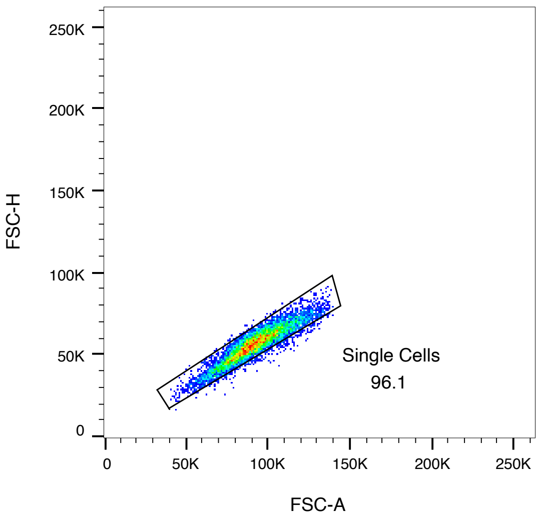

-_D7_2_004.fcs
LN229
8516

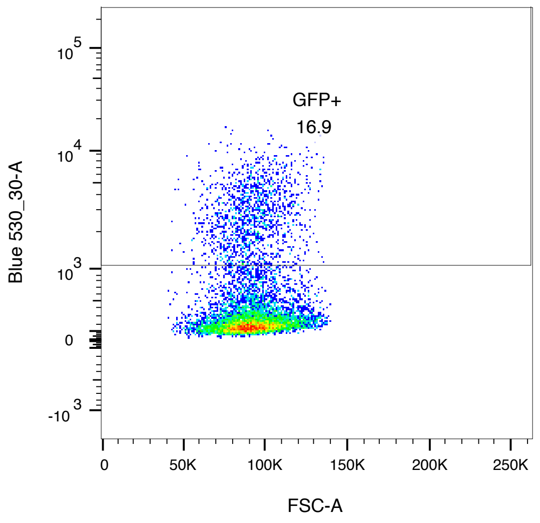

-_D7_2_004.fcs
Single Cells
8183

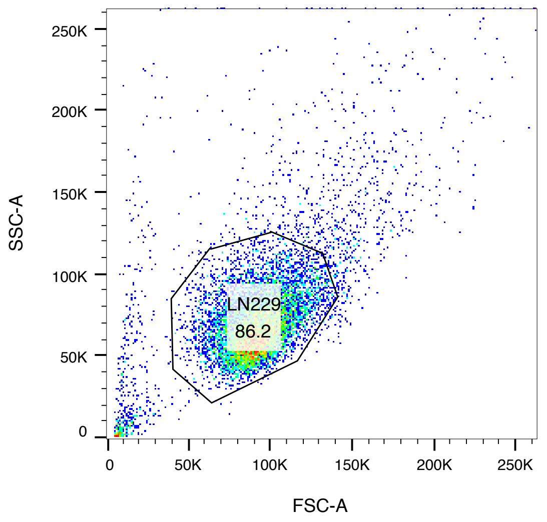

-_D7_003.fcs
Ungated
10000

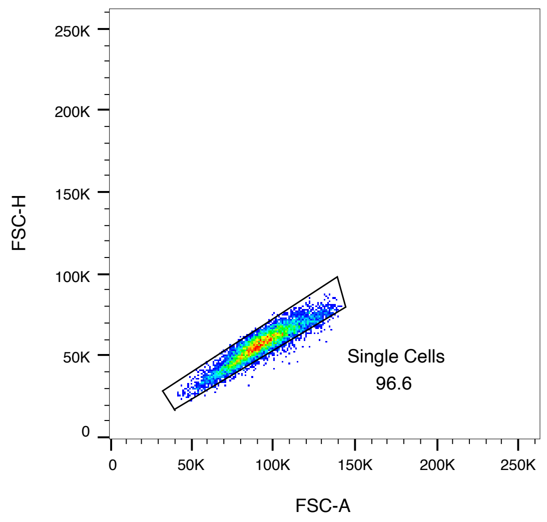

-_D7_003.fcs
LN229
8618

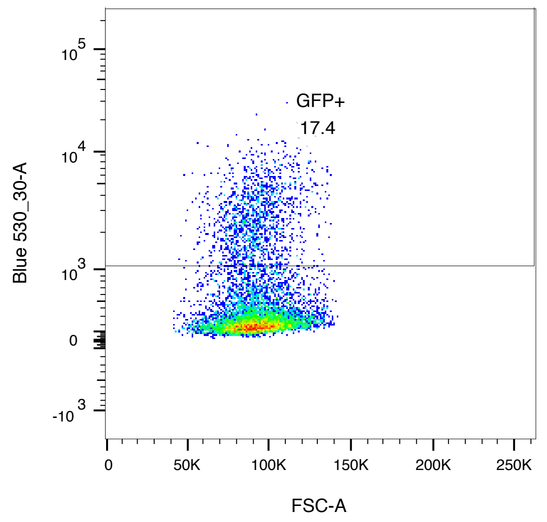

-_D7_003.fcs
Single Cells
8323

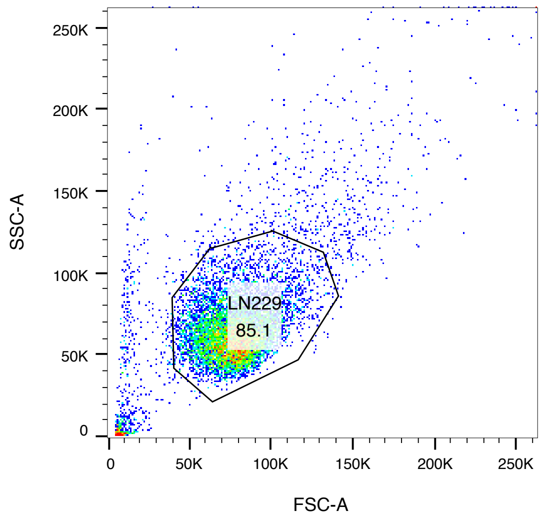

-_E2_2_009.fcs
Ungated
10000

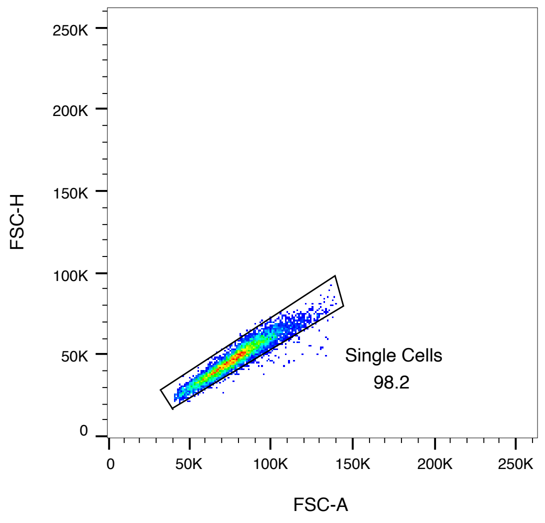

-_E2_2_009.fcs
LN229
8509

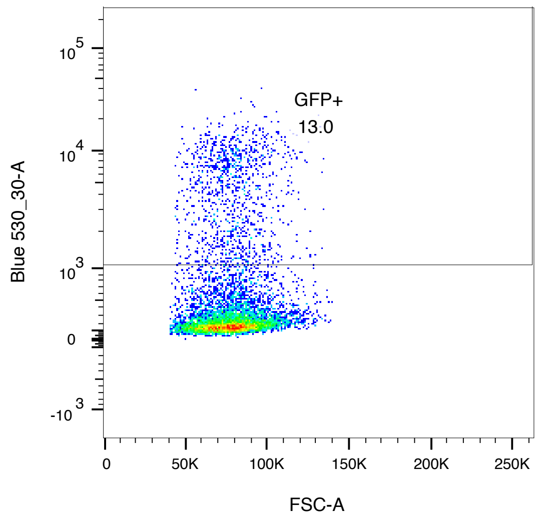

-_E2_2_009.fcs
Single Cells
8354

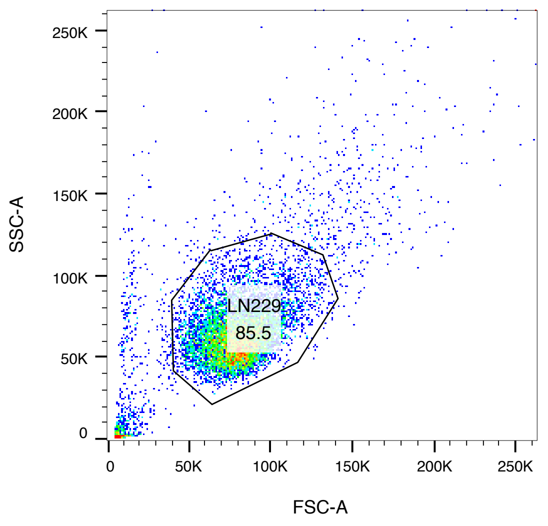

-_E2_008.fcs
Ungated
10000

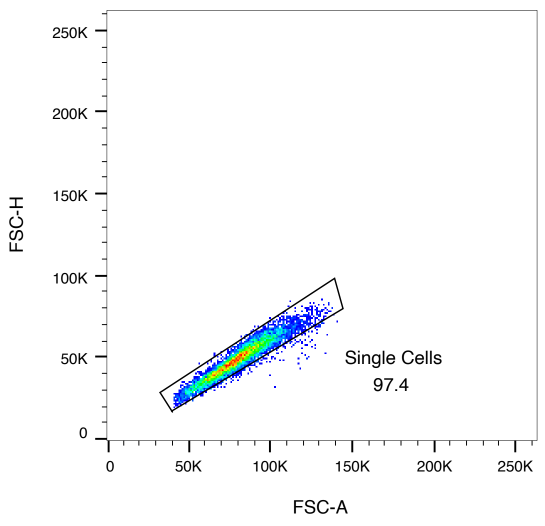

-_E2_008.fcs
LN229
8546

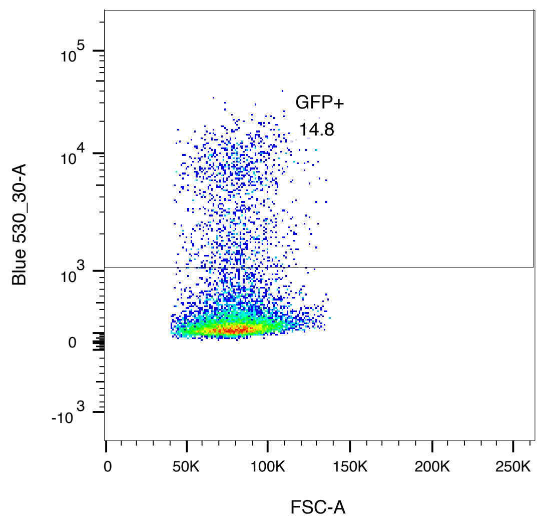

-_E2_008.fcs
Single Cells
8327

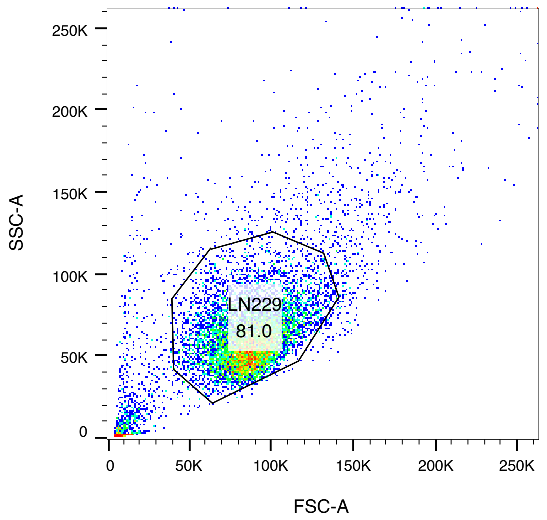

-_C3_2_015.fcs
Ungated
10000

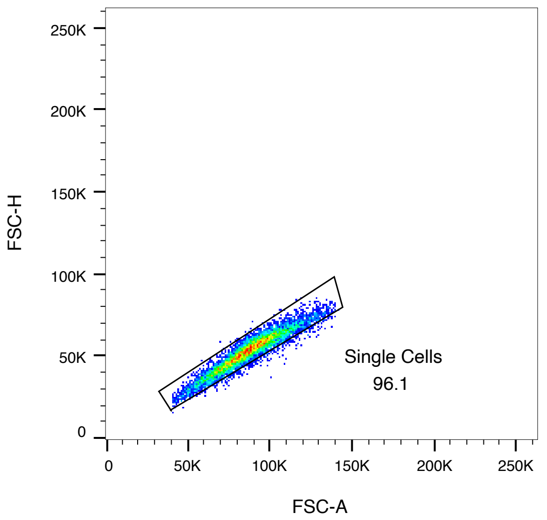

-_C3_2_015.fcs
LN229
8096

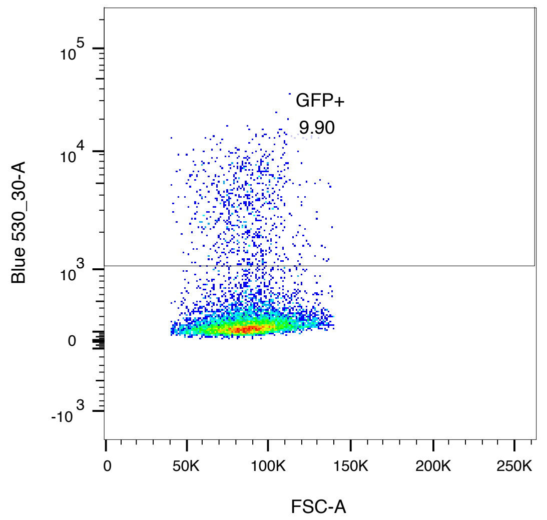

-_C3_2_015.fcs
Single Cells
7777

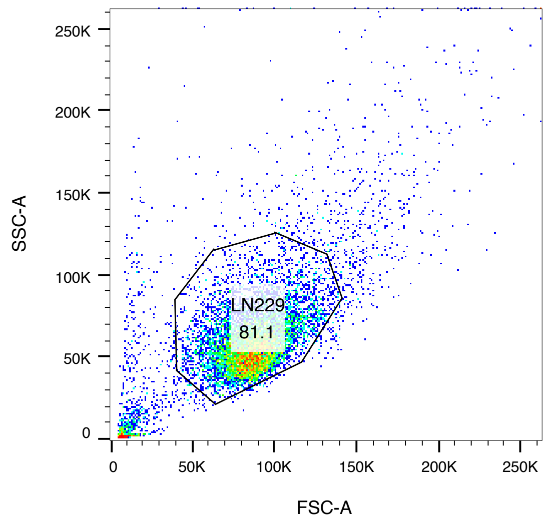

-_C3_014.fcs
Ungated
10000

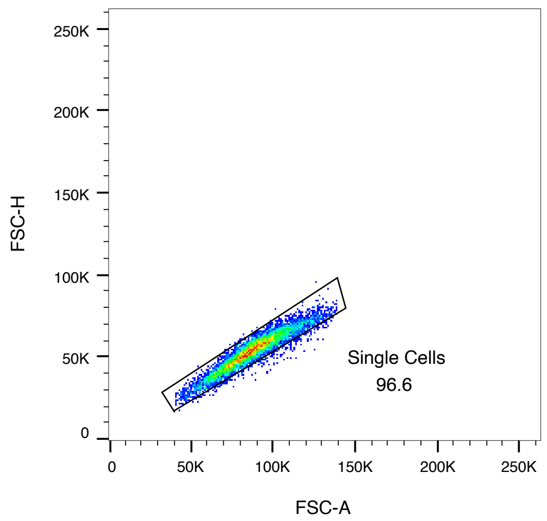

-_C3_014.fcs
LN229
8110

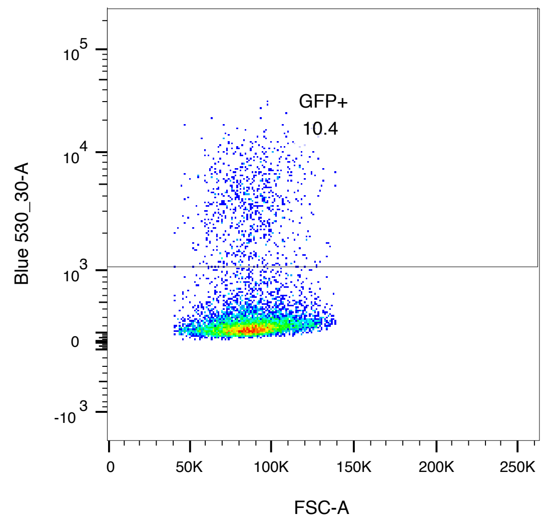

-_C3_014.fcs
Single Cells
7835

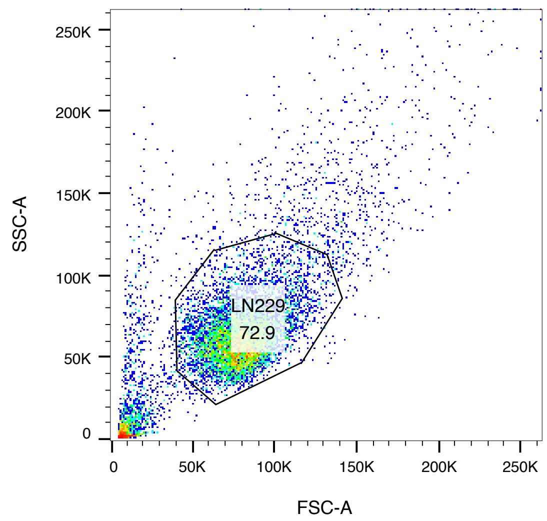

-_M3_2_011.fcs
Ungated
10000

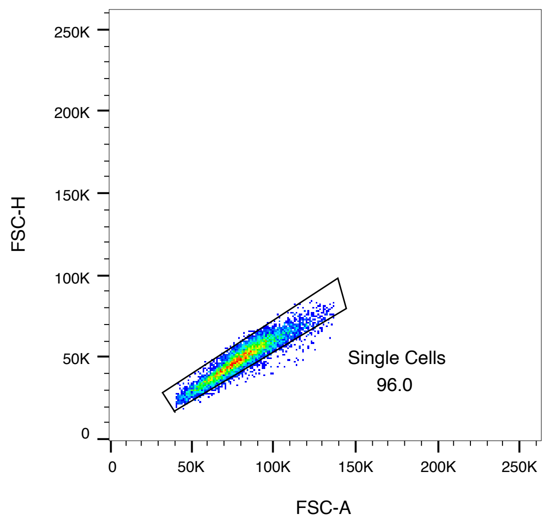

-_M3_2_011.fcs
LN229
7292

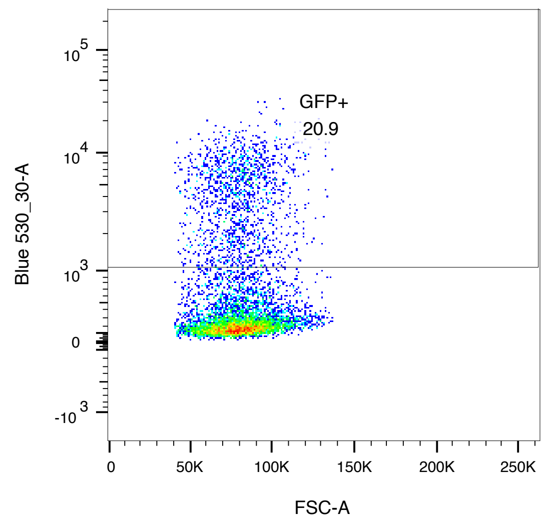

-_M3_2_011.fcs
Single Cells
6997

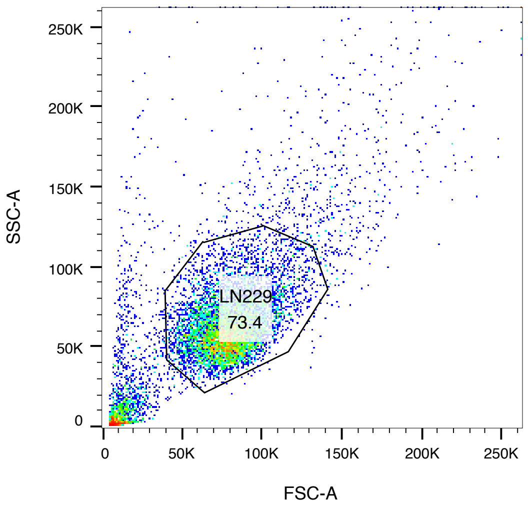

-_M3_010.fcs
Ungated
10000

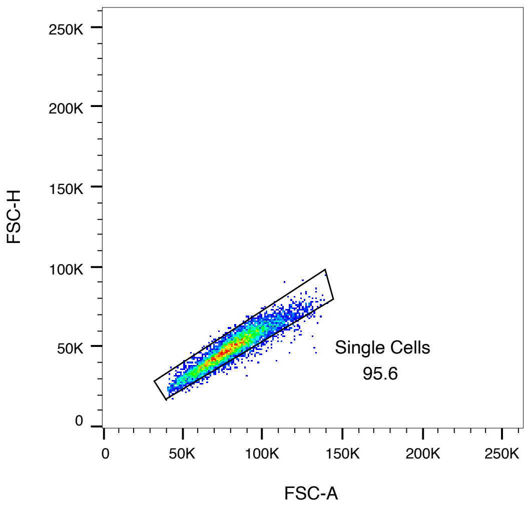

-_M3_010.fcs
LN229
7340

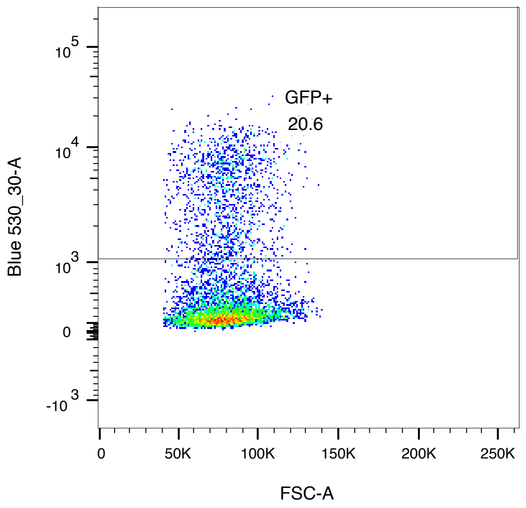

-_M3_010.fcs
Single Cells
7019

S5C Fig

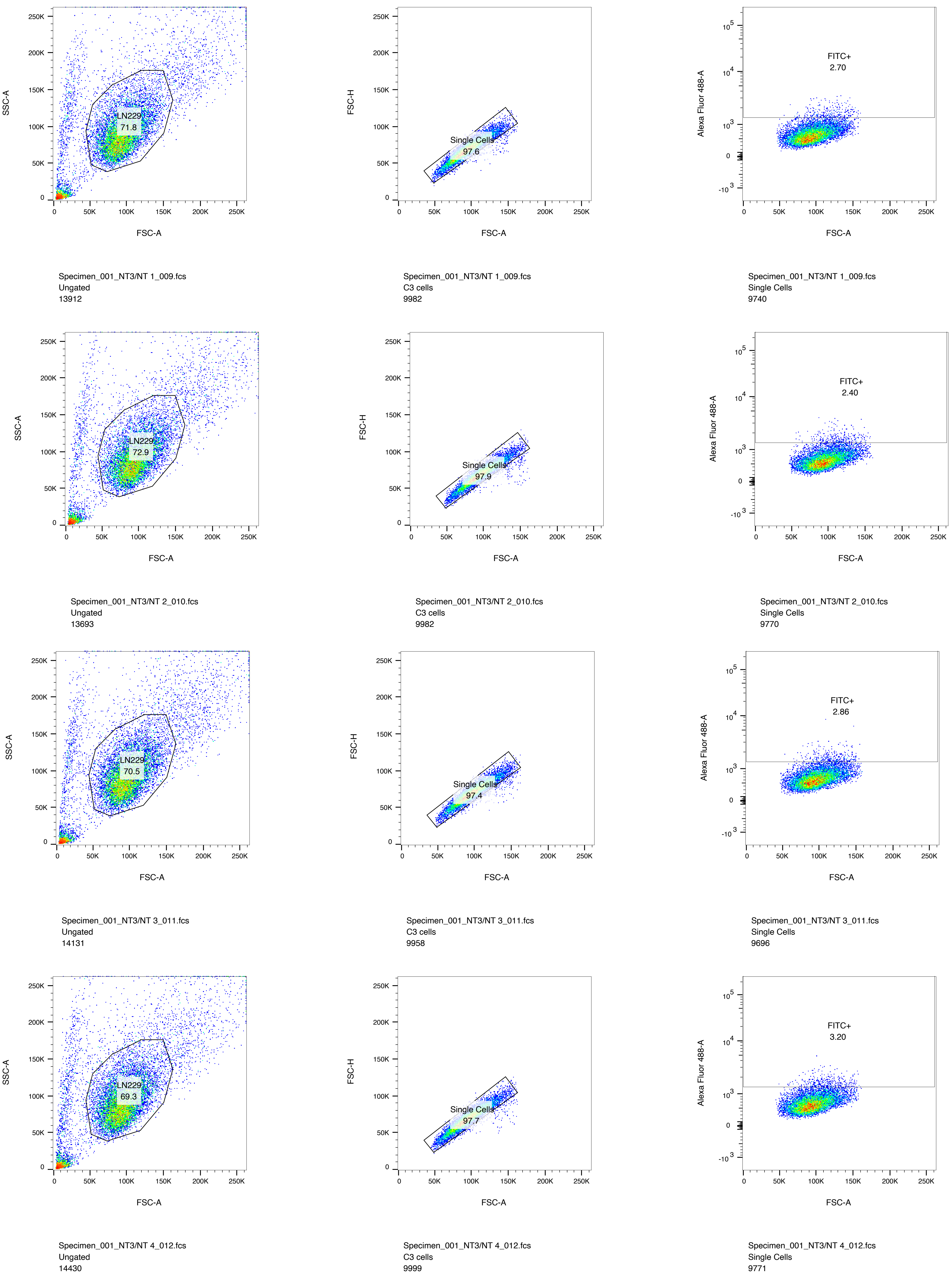

SSC-A
FSC-A
LN229
71.8
Specimen_001_NT3/NT 1_009.fcs
Ungated
13912
FSC-H
FSC-A
Single Cells
97.6
Specimen_001_NT3/NT 1_009.fcs
C3 cells
9982
Alexa Fluor 488-A
FSC-A
FITC+
2.70
Specimen_001_NT3/NT 1_009.fcs
Single Cells
9740
LN229
72.9
Specimen_001_NT3/NT 2_010.fcs
Ungated
13693
Single Cells
97.9
Specimen_001_NT3/NT 2_010.fcs
C3 cells
9982
FITC+
2.40
Specimen_001_NT3/NT 2_010.fcs
Single Cells
9770
LN229
70.5
Specimen_001_NT3/NT 3_011.fcs
Ungated
14131
Single Cells
97.4
Specimen_001_NT3/NT 3_011.fcs
C3 cells
9958
FITC+
2.86
Specimen_001_NT3/NT 3_011.fcs
Single Cells
9696
LN229
69.3
Specimen_001_NT3/NT 4_012.fcs
Ungated
14430
Single Cells
97.7
Specimen_001_NT3/NT 4_012.fcs
C3 cells
9999
FITC+
3.20
Specimen_001_NT3/NT 4_012.fcs
Single Cells
9771

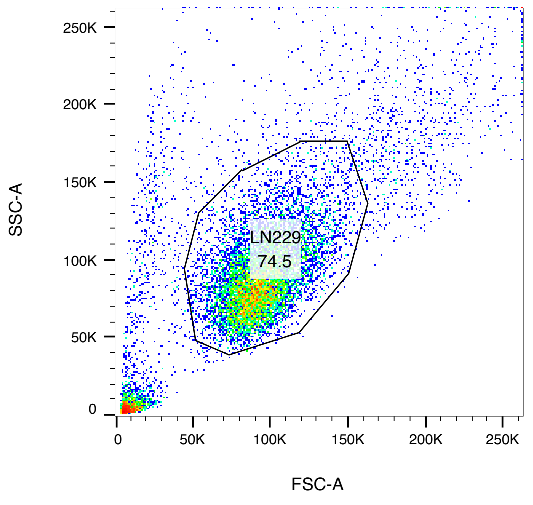

Specimen_001_NT NT 5_013.fcs
Ungated
13359

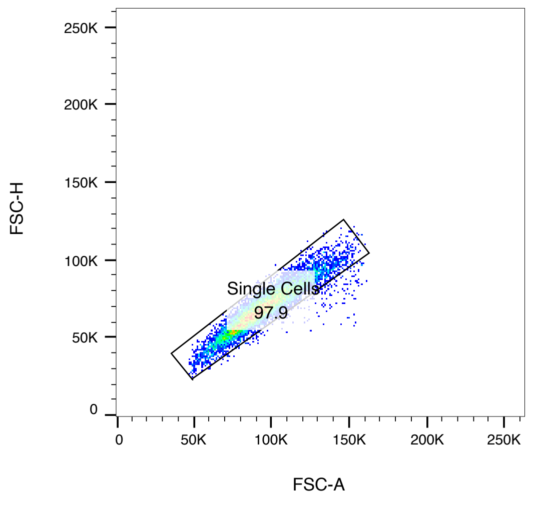

Specimen_001_NT NT 5_013.fcs
C3 cells
9950

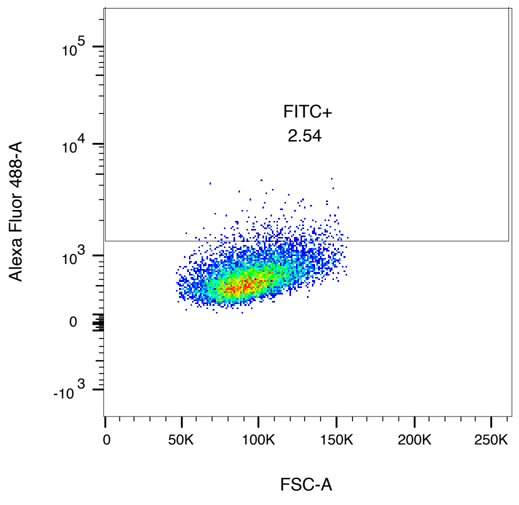

Specimen_001_NT NT 5_013.fcs
Single Cells
9746

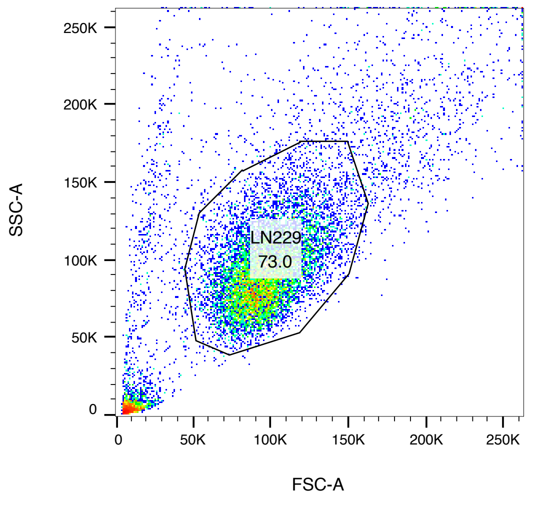

Specimen_001_NT3/NT 6_014.fcs
Ungated
13657

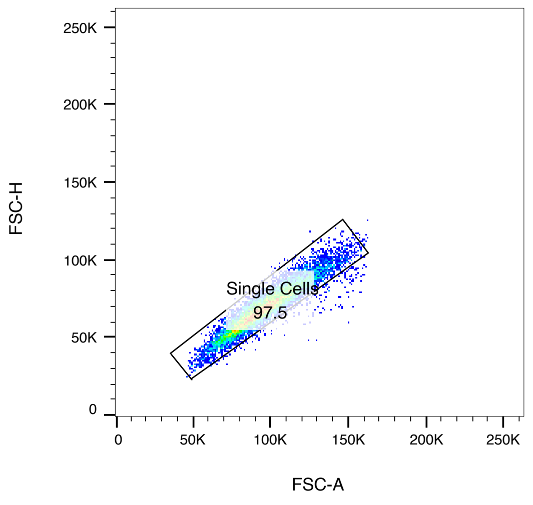

Specimen_001_NT3/NT 6_014.fcs
C3 cells
9971

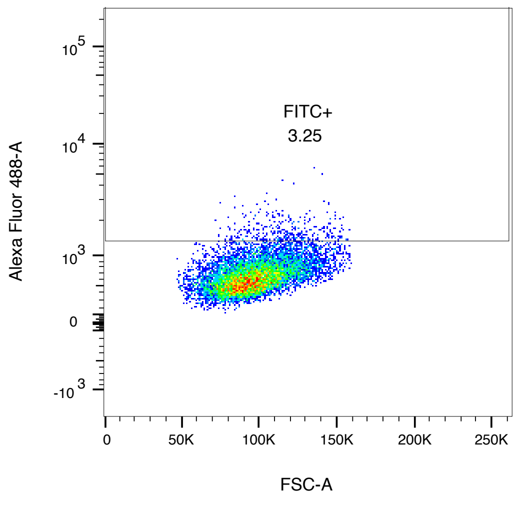

Specimen_001_NT3/NT 6_014.fcs
Single Cells
9726

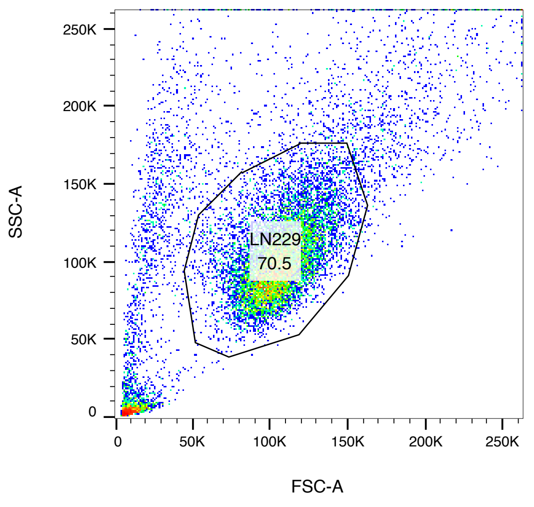

Specimen_001_NT3/HNRNPK 1_015.fcs
Ungated
14287

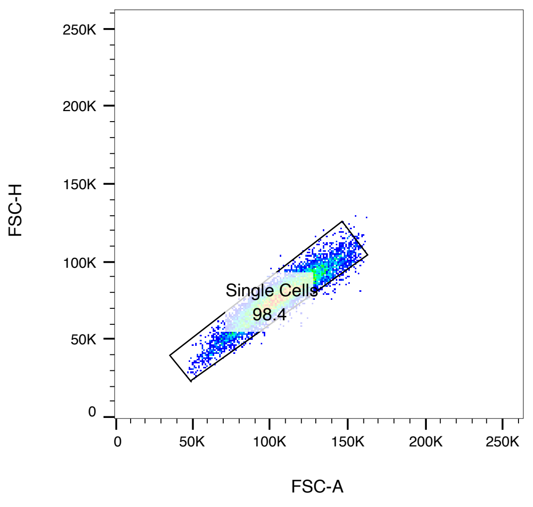

Specimen_001_NT3/HNRNPK 1_015.fcs
C3 cells
10075

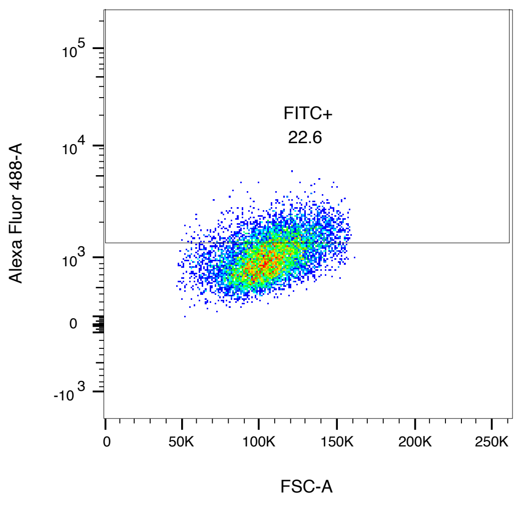

Specimen_001_NT3/HNRNPK 1_015.fcs
Single Cells
9918

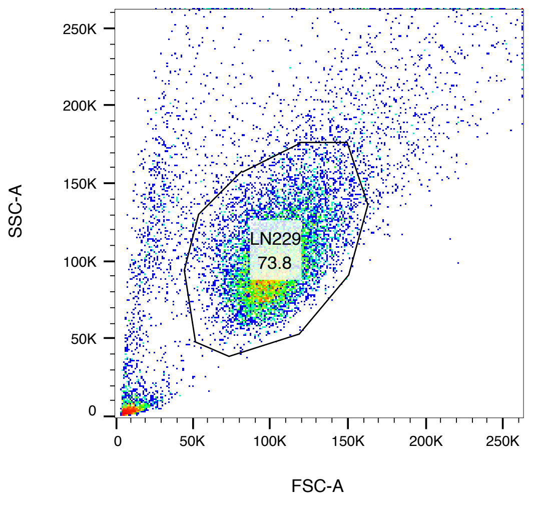

Specimen_001_NT3/HNRNPK 2_016.fcs
Ungated
13626

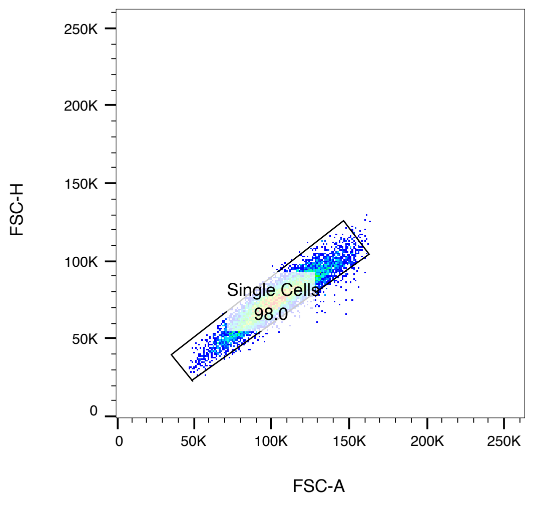

Specimen_001_NT3/HNRNPK 2_016.fcs
C3 cells
10051

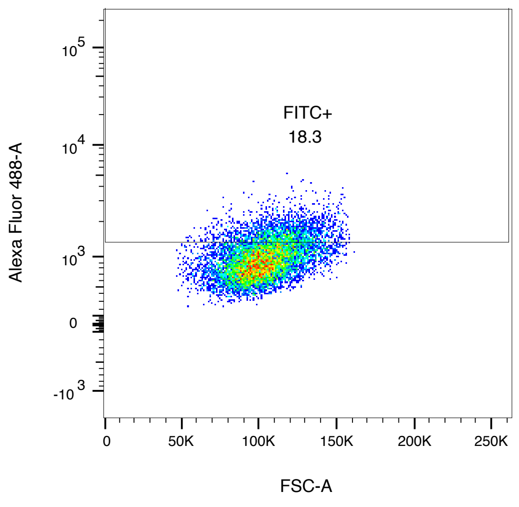

Specimen_001_NT3/HNRNPK 2_016.fcs
Single Cells
9848

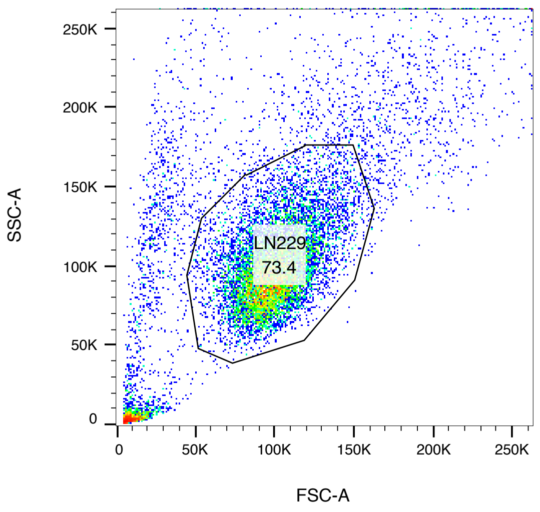

Specimen_001_NT3/HNRNPK 3_017.fcs
Ungated
13724

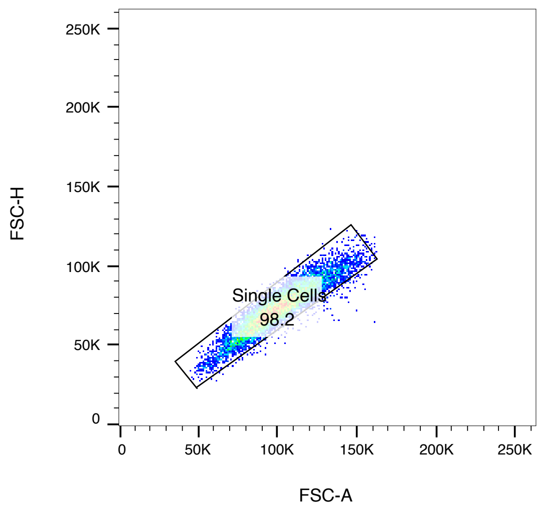

Specimen_001_NT3/HNRNPK 3_017.fcs
C3 cells
10080

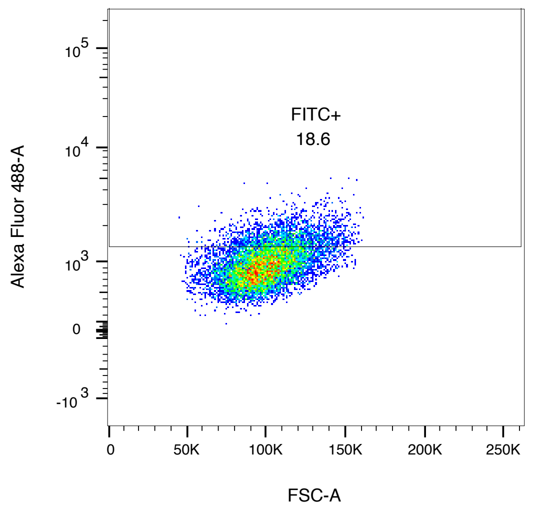

Specimen_001_NT3/HNRNPK 3_017.fcs
Single Cells
9899

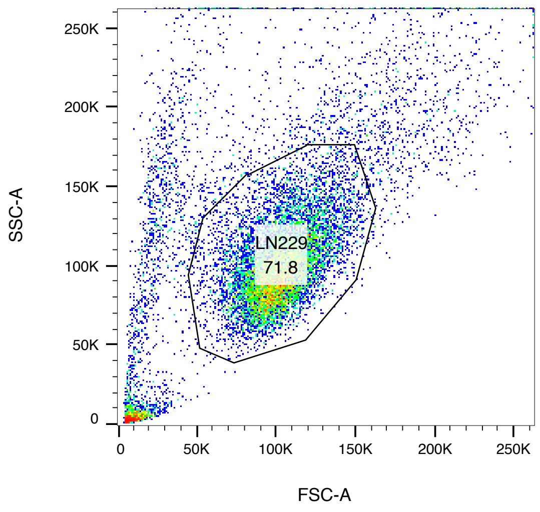

Specimen_001_NT3/HNRNPK 4_018.fcs
Ungated
13997

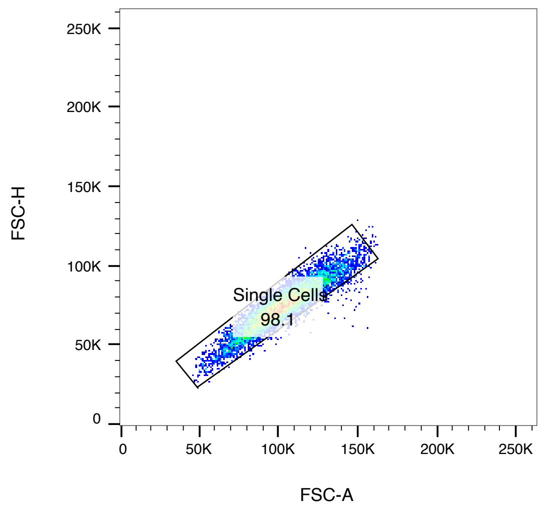

Specimen_001_NT3/HNRNPK 4_018.fcs
C3 cells
10054

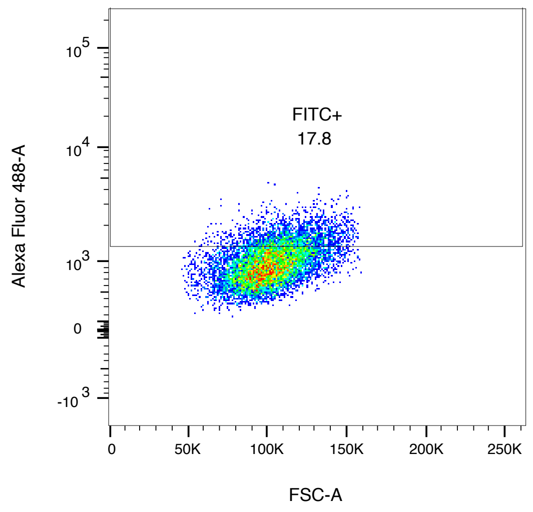

Specimen_001_NT3/HNRNPK 4_018.fcs
Single Cells
9862

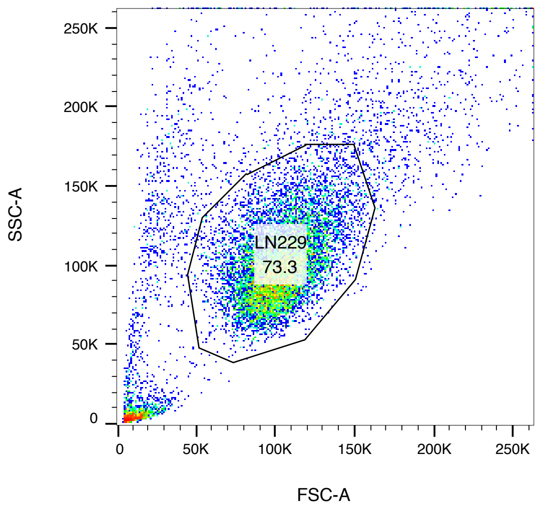

Specimen_001_NT3/HNRNPK 5_019.fcs
Ungated
13721

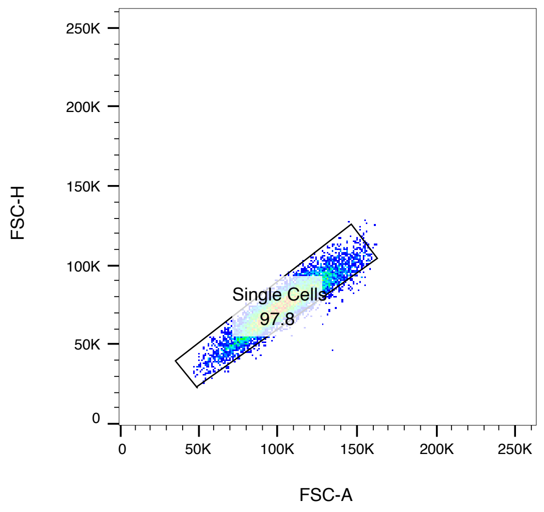

Specimen_001_NT3/HNRNPK 5_019.fcs
C3 cells
10058

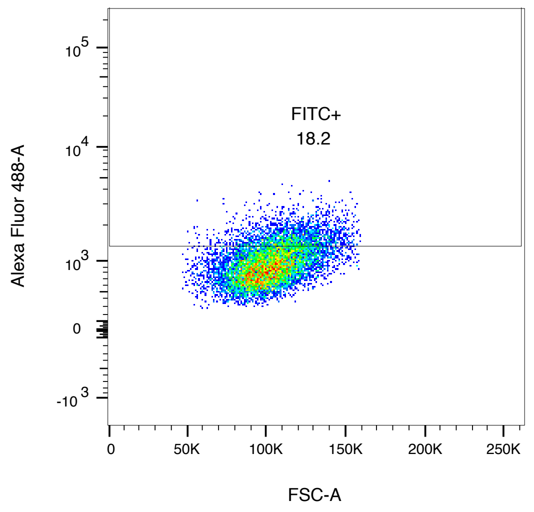

Specimen_001_NT3/HNRNPK 5_019.fcs
Single Cells
9838

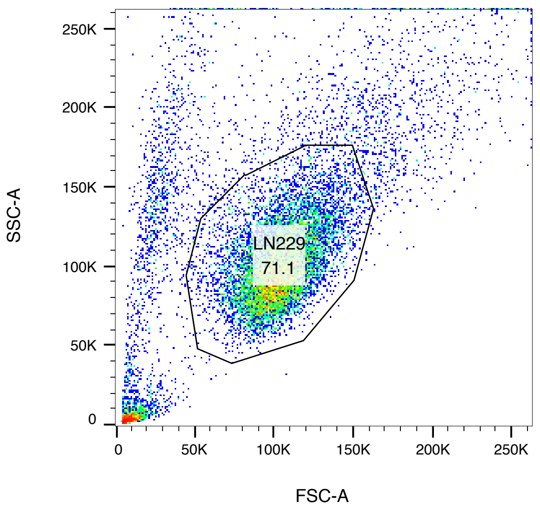

Specimen_001_NT3/HNRNPK 6_020.fcs
Ungated
14159

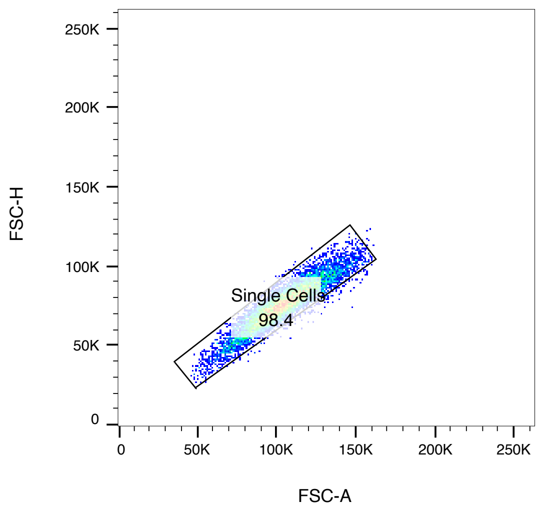

Specimen_001_NT3/HNRNPK 6_020.fcs
C3 cells
10060

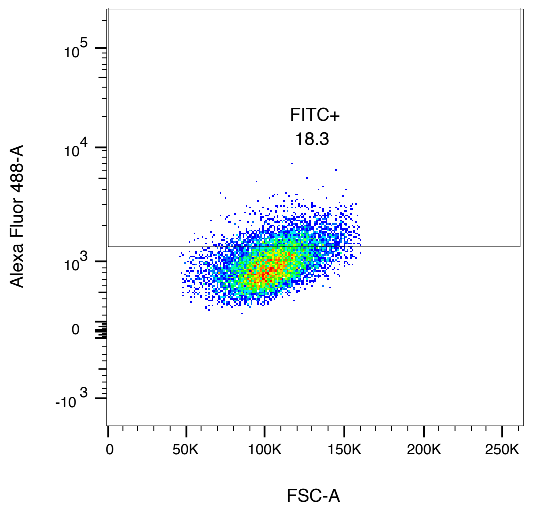

Specimen_001_NT3/HNRNPK 6_020.fcs
Single Cells
9901

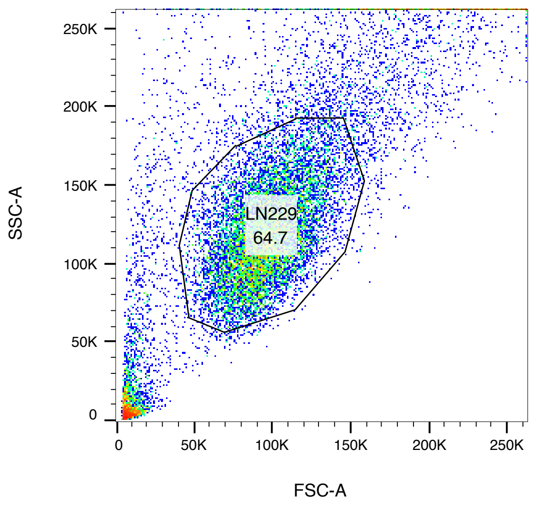

Specimen_001_TFAP2C/NT 1_021.fcs
Ungated
17078

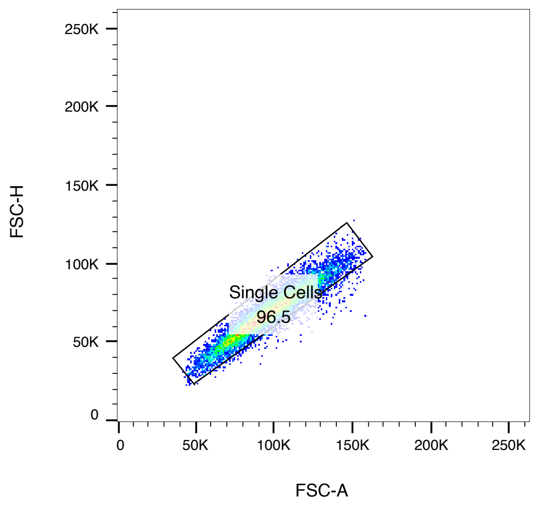

Specimen_001_TFAP2C/NT 1_021.fcs
C3 cells
11046

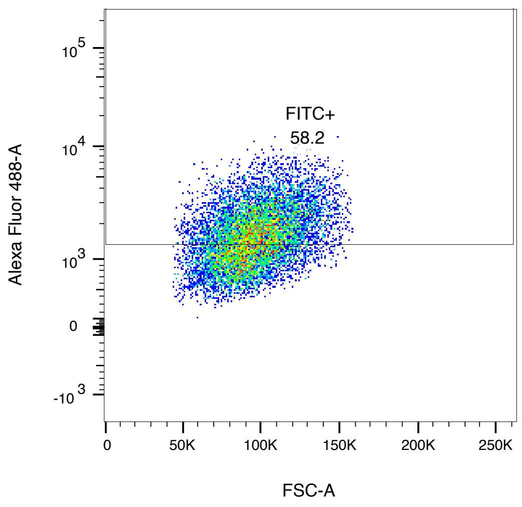

Specimen_001_TFAP2C/NT 1_021.fcs
Single Cells
10656

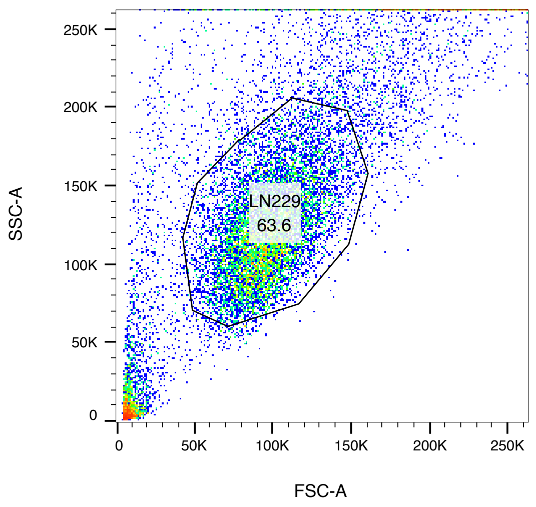

Specimen_001_TFAP2C/NT 2_022.fcs
Ungated
16297

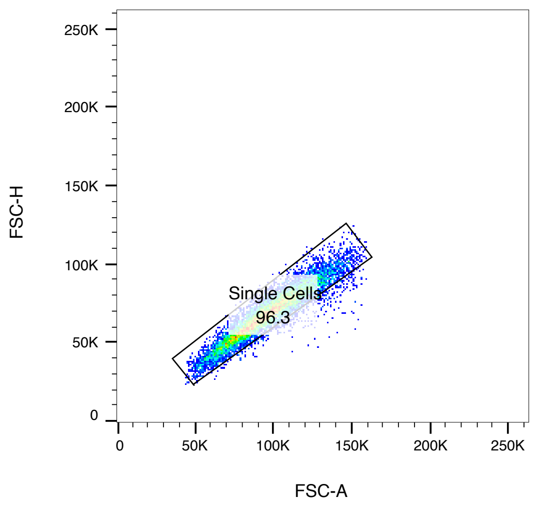

Specimen_001_TFAP2C/NT 2_022.fcs
C3 cells
10372

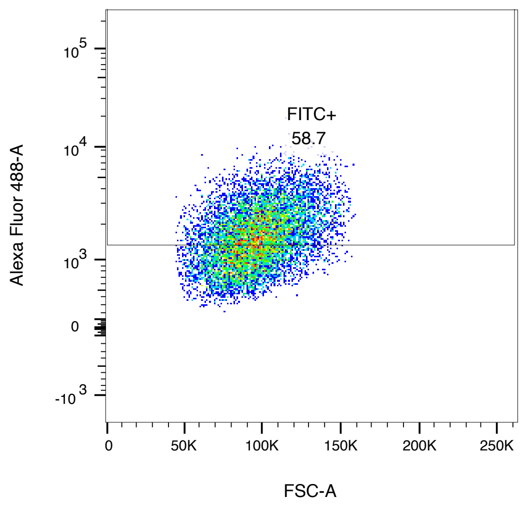

Specimen_001_TFAP2C/NT 2_022.fcs
Single Cells
9988

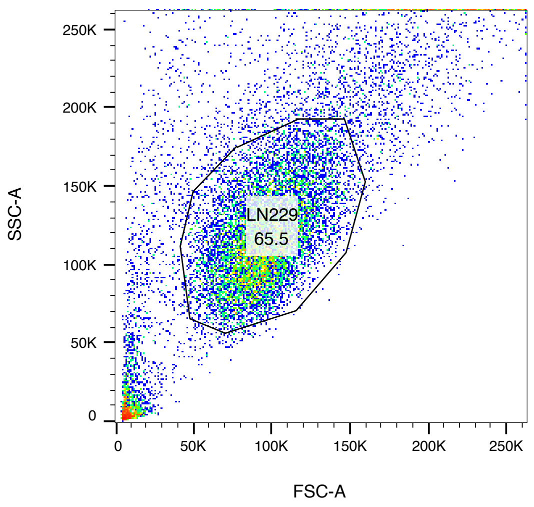

Specimen_001_TFAP2C/NT 3_023.fcs
Ungated
15523

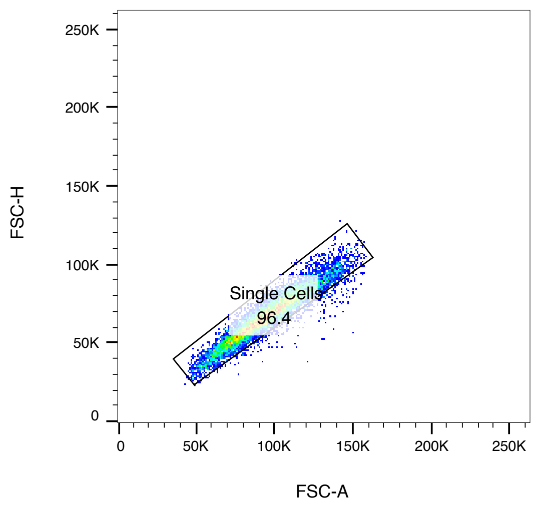

Specimen_001_TFAP2C/NT 3_023.fcs
C3 cells
10174

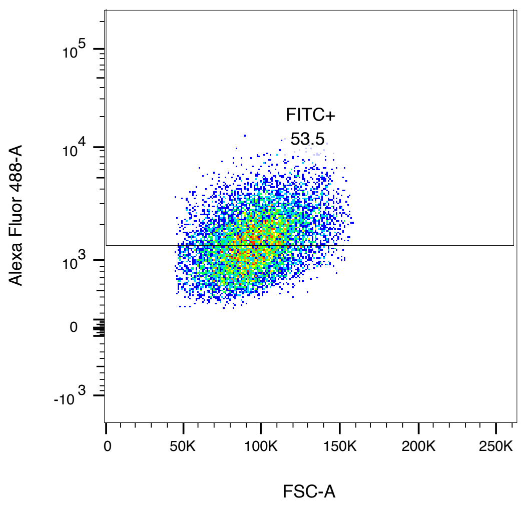

Specimen_001_TFAP2C/NT 3_023.fcs
Single Cells
9810

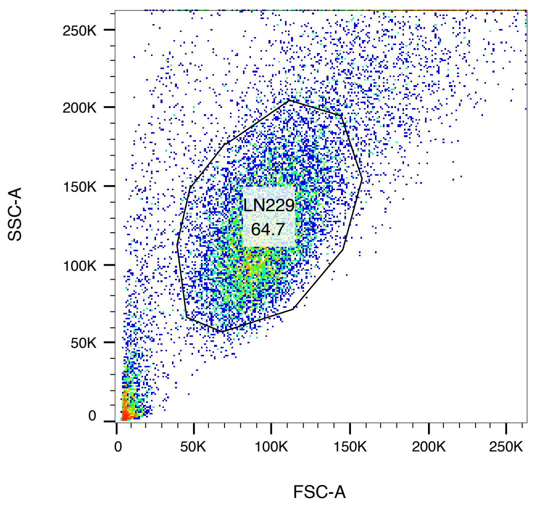

Specimen_001_TFAP2C/NT 4_024.fcs
Ungated
16071

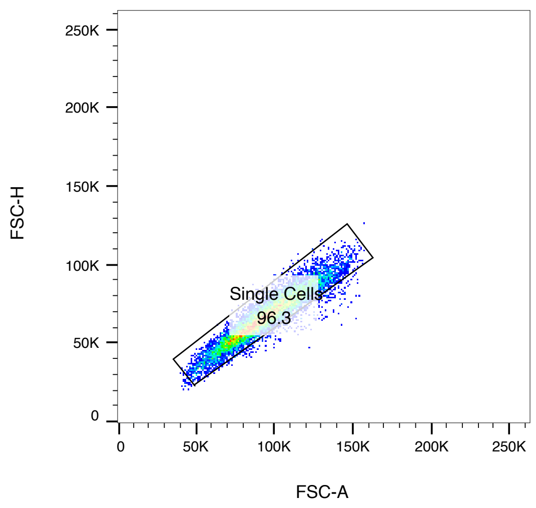

Specimen_001_TFAP2C/NT 4_024.fcs
C3 cells
10390

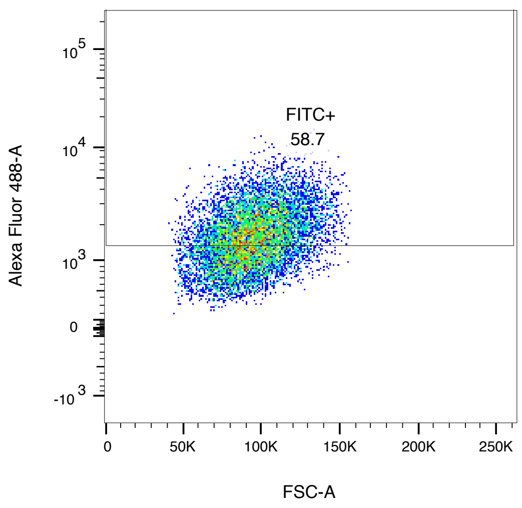

Specimen_001_TFAP2C/NT 4_024.fcs
Single Cells
10009

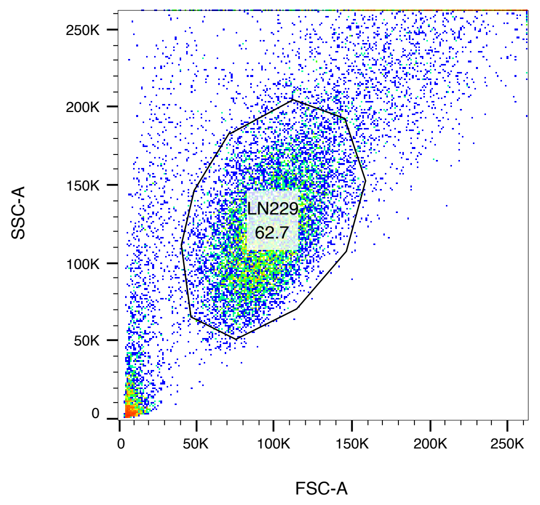

Specimen_001_TFAP2C/NT 5_025.fcs
Ungated
16670

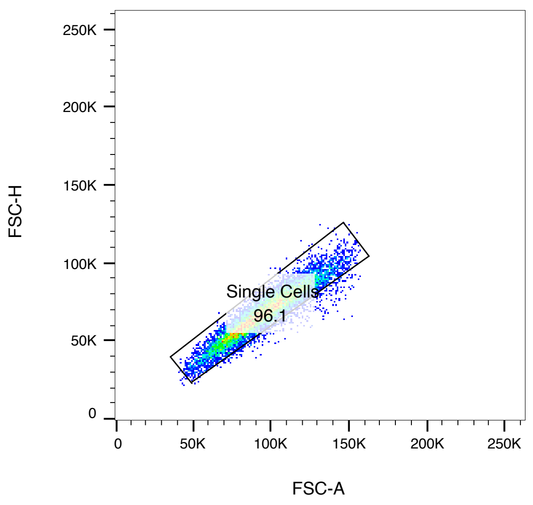

Specimen_001_TFAP2C/NT 5_025.fcs
C3 cells
10456

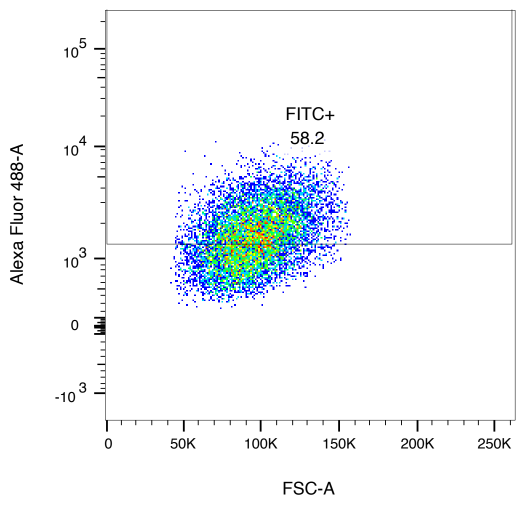

Specimen_001_TFAP2C/NT 5_025.fcs
Single Cells
10046

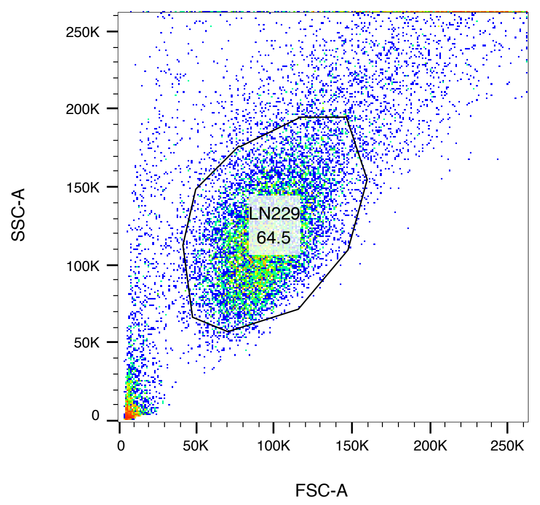

Specimen_001_TFAP2C/NT 6_026.fcs
Ungated
15792

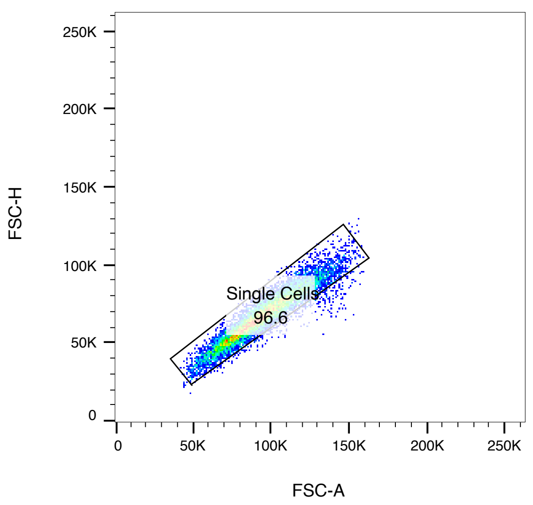

Specimen_001_TFAP2C/NT 6_026.fcs
C3 cells
10189

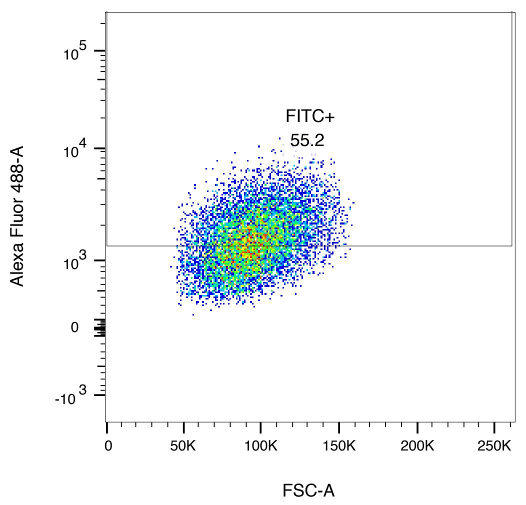

Specimen_001_TFAP2C/NT 6_026.fcs
Single Cells
9838

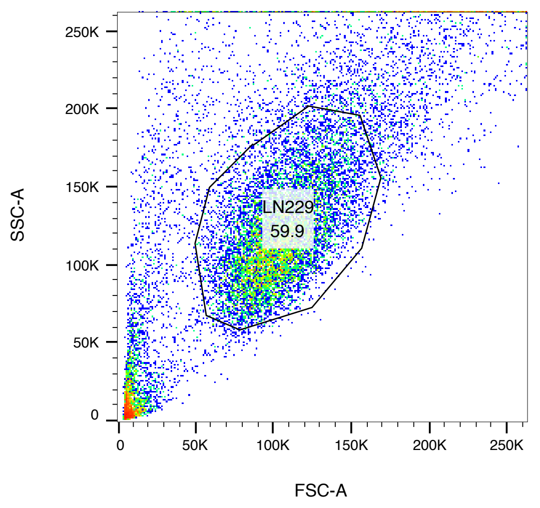

Specimen_001_TFAP2C/HNRNPK 1_027.fcs
Ungated
17072

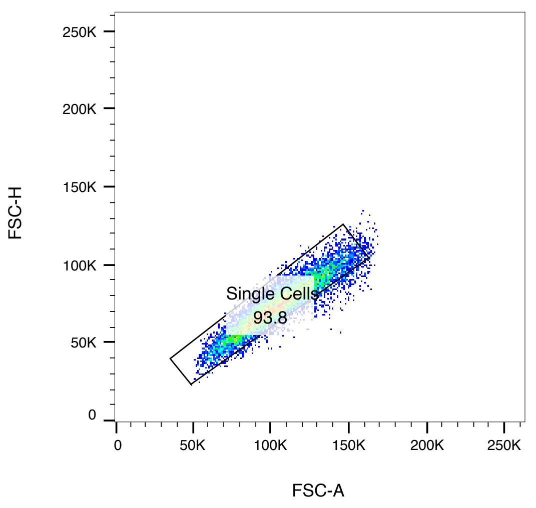

Specimen_001_TFAP2C/HNRNPK 1_027.fcs
C3 cells
10231

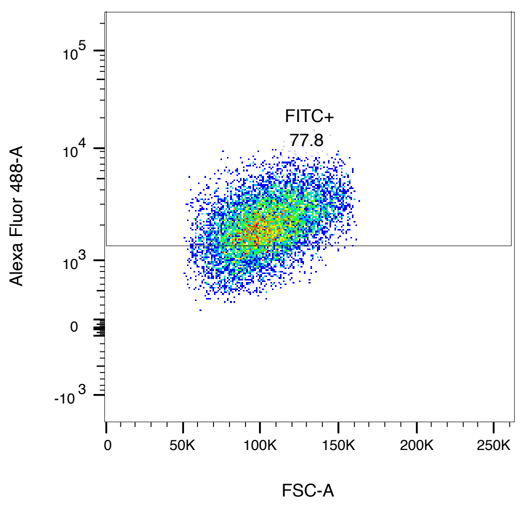

Specimen_001_TFAP2C/HNRNPK 1_027.fcs
Single Cells
9592

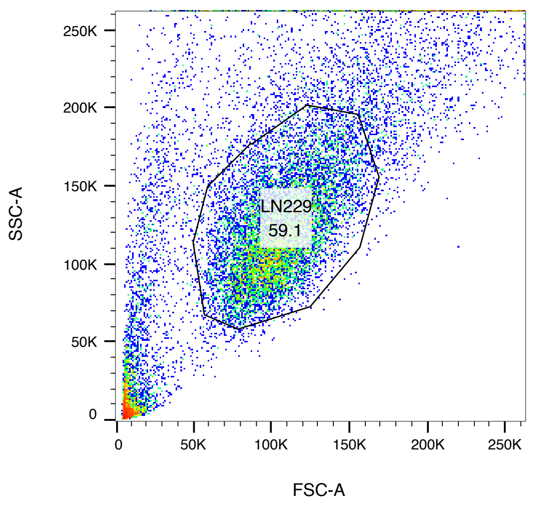

Specimen_001_TFAP2C/HNRNPK 2_028.fcs
Ungated
17346

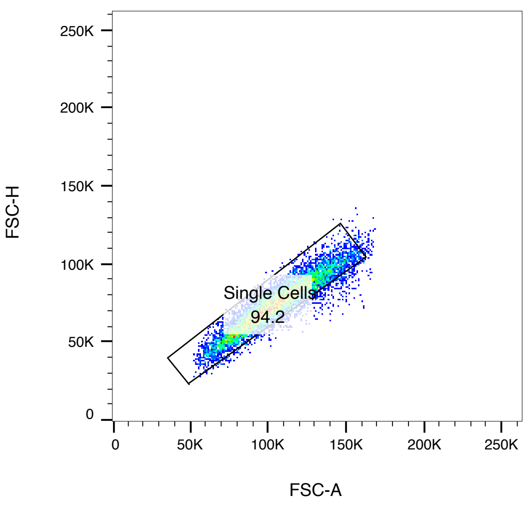

Specimen_001_TFAP2C/HNRNPK 2_028.fcs
C3 cells
10249

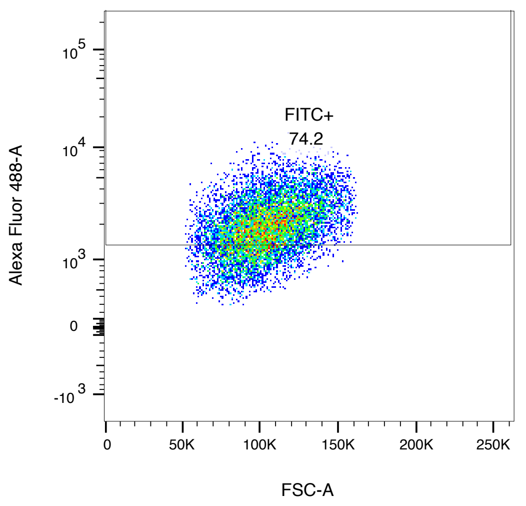

Specimen_001_TFAP2C/HNRNPK 2_028.fcs
Single Cells
9654

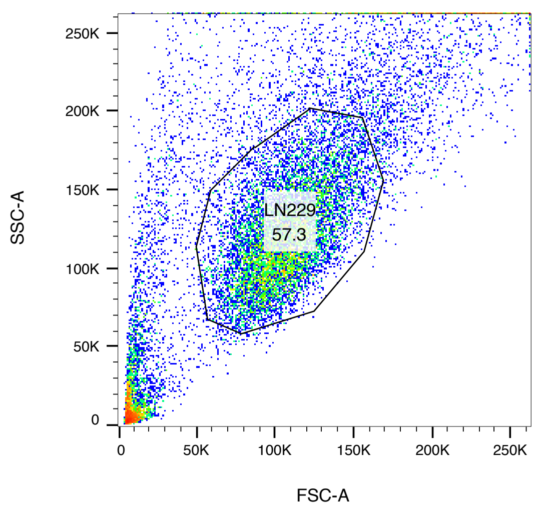

Specimen_001_TFAP2C/HNRNPK 3_029.fcs
Ungated
17883

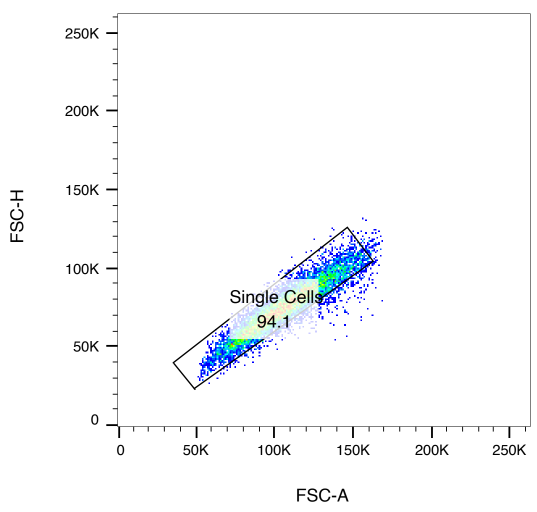

Specimen_001_TFAP2C/HNRNPK 3_029.fcs
C3 cells
10254

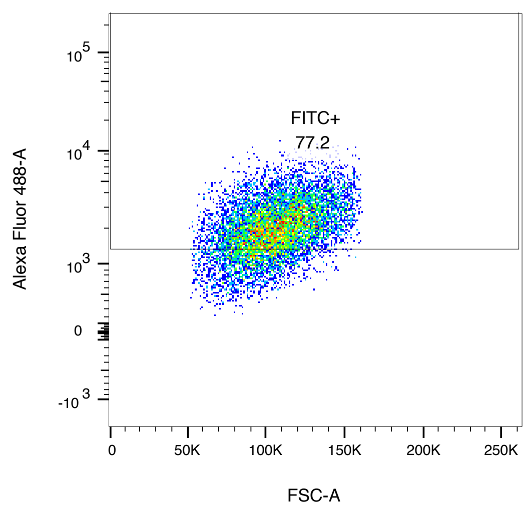

Specimen_001_TFAP2C/HNRNPK 3_029.fcs
Single Cells
9646

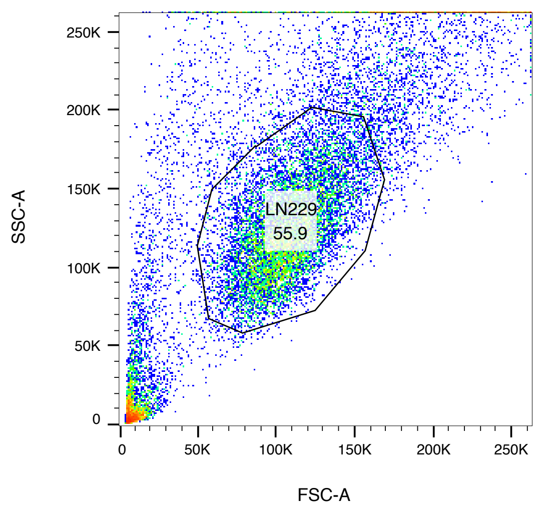

Specimen_001_TFAP2C/HNRNPK 4_030.fcs
Ungated
18474

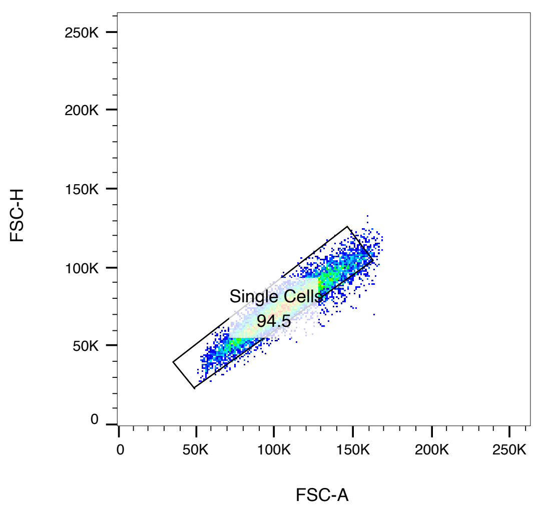

Specimen_001_TFAP2C/HNRNPK 4_030.fcs
C3 cells
10329

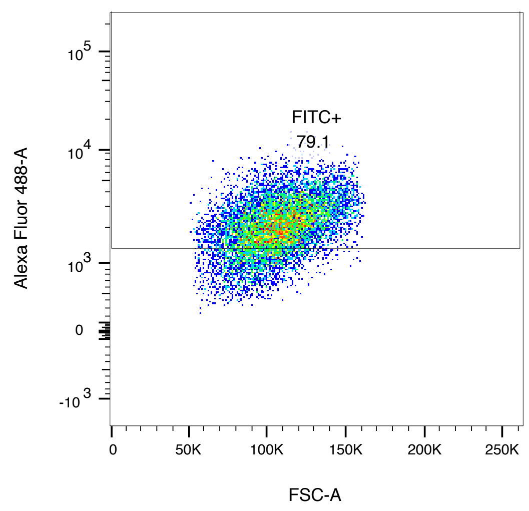

Specimen_001_TFAP2C/HNRNPK 4_030.fcs
Single Cells
9757

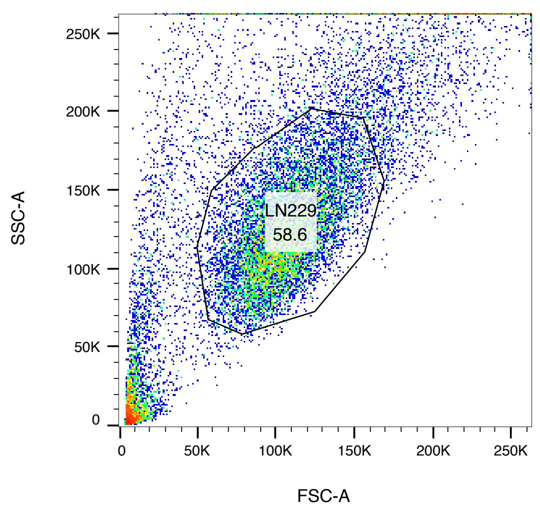

Specimen_001_TFAP2C/HNRNPK 5_031.fcs
Ungated
17627

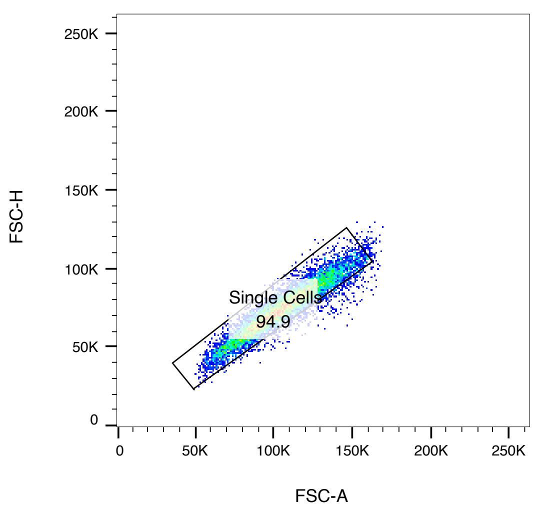

Specimen_001_TFAP2C/HNRNPK 5_031.fcs
C3 cells
10327

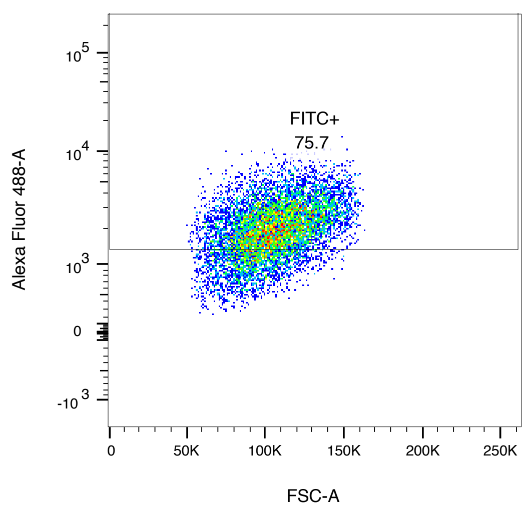

Specimen_001_TFAP2C/HNRNPK 5_031.fcs
Single Cells
9797

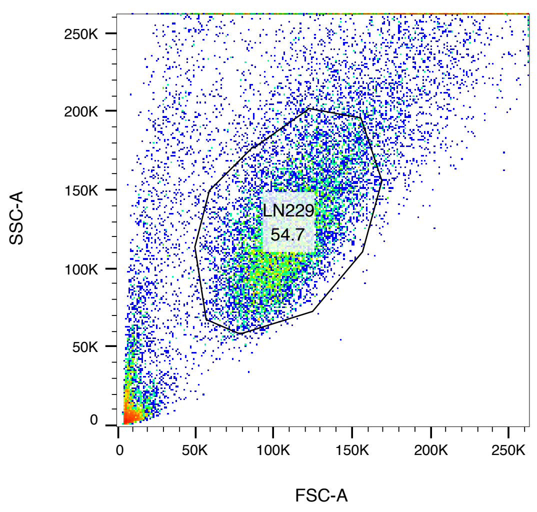

Specimen_001_TFAP2C/HNRNPK 6_032.fcs
Ungated
18928

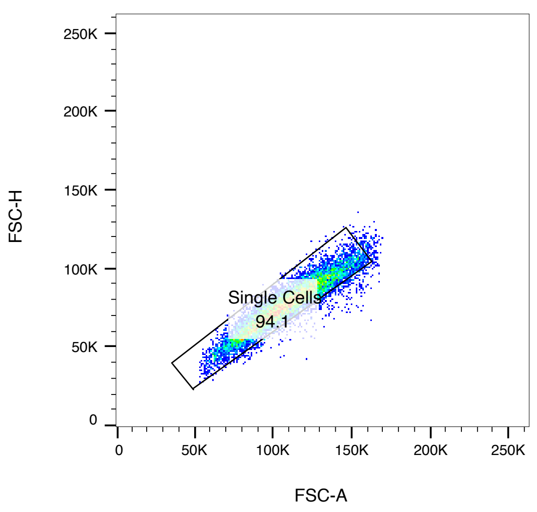

Specimen_001_TFAP2C/HNRNPK 6_032.fcs
C3 cells
10362

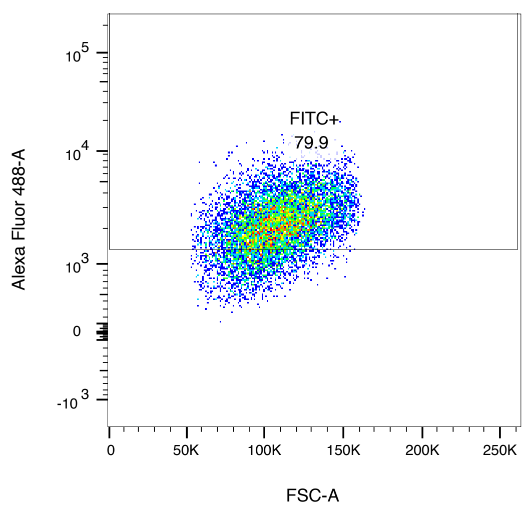

Specimen_001_TFAP2C/HNRNPK 6_032.fcs
Single Cells
9748

S7E Fig

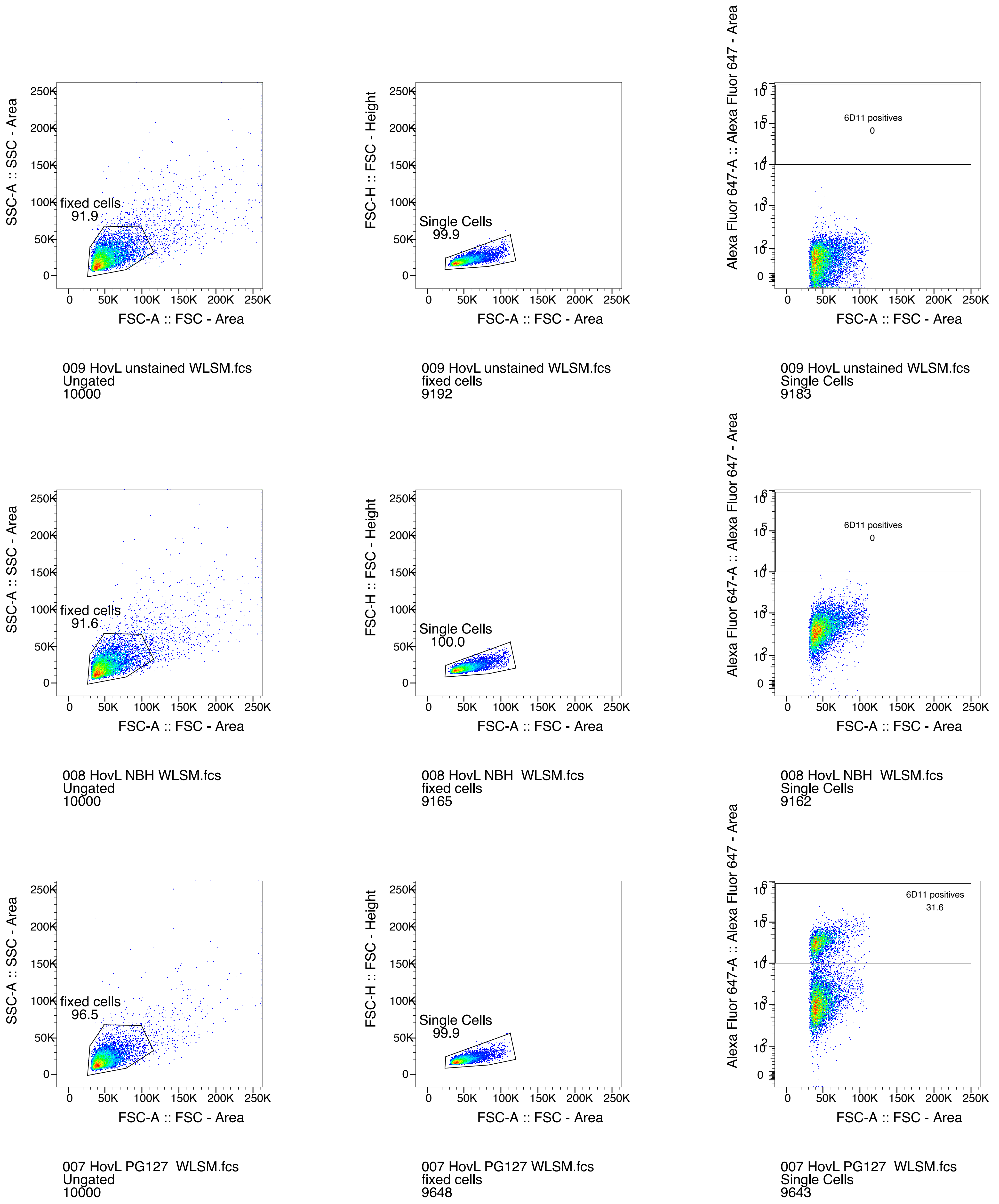
SSC-A :: SSC - Area
FSC-A :: FSC - Area
FSC-H :: FSC - Height
Alexa Fluor 647-A :: Alexa Fluor 647 - Area
fixed cells
91.9
Single Cells
99.9
6D11 positives
0
009 HovL unstained WLSM.fcs
Ungated
10000
009 HovL unstained WLSM.fcs
fixed cells
9192
009 HovL unstained WLSM.fcs
Single Cells
9183
fixed cells
91.6
Single Cells
100.0
6D11 positives
0
008 HovL NBH WLSM.fcs
Ungated
10000
008 HovL NBH WLSM.fcs
fixed cells
9165
008 HovL NBH WLSM.fcs
Single Cells
9162
fixed cells
96.5
Single Cells
99.9
6D11 positives
31.6
007 HovL PG127 WLSM.fcs
Ungated
10000
007 HovL PG127 WLSM.fcs
fixed cells
9648
007 HovL PG127 WLSM.fcs
Single Cells
9643
