# Supplementary material for: Prion propagation is controlled by a hierarchical network involving the nuclear Tfap2c and hnRNP K factors and the cytosolic mTORC1 complex
Source: PLoS Pathog. 2026 Apr 20;22(4):e1014056. doi: 10.1371/journal.ppat.1014056 (PMC13108870; doi:10.1371/journal.ppat.1014056)
Supplement: Attachment — Submitted filename: 155075_1_rebuttal_3662851_tp52z2.pdf [file ppat.1014056.s018.pdf]

Full Revision

Manuscript number: RC-2025-02904
Corresponding author(s): Adriano Aguzzi

[Please use this template only if the submitted manuscript should be considered by the affiliate journal as a full revision in response to the points raised by the reviewers.

*If you wish to submit a preliminary revision with a revision plan, please use our "Revision Plan" template. **It is important to use the appropriate template to clearly inform the editors of your intentions.**]*

1. General Statements [optional]

This section is optional. Insert here any general statements you wish to make about the goal of the study or about the reviews.

We are grateful for the reviewers' constructive comments and suggestions, which contributed to improving our manuscript. We are pleased to see that our work was described as an "interesting manuscript in which a lot of work has been undertaken". We are also encouraged by the fact that the experiments were considered "on the whole well done, carefully documented, and support most of the conclusions drawn," and that our findings were viewed as providing "mechanistic insight into how HNRNPK modulates prion propagation" and potentially offering "new mechanical insight of hnRNPK function and its interaction with TFAP2C."
We conducted several new experiments and revised specific sections of the manuscript, as detailed below in the point-by-point response in this letter.

2. Point-by-point description of the revisions

This section is mandatory. Please insert a point-by-point reply describing the revisions that were already carried out and included in the transferred manuscript.

Reviewer #1 (Evidence, reproducibility and clarity (Required)):

The paper by Sellitto describes studies to determine the mechanism by which hnRNPK modulates the propagation of prion. The authors use cell models lacking HNRNPK, which is lethal, in a CRISPR screen to identify genes that suppress lethality. Based on this screen to 2 different cell lines, gene termed Tfap2C emerged as a candidate for interaction with HNRNPK. The show that Tfap2C counteracts the actions of HNRNPK with respect to prion propagation. Cells lacking

HNRNPK show increased PrPSc levels. Overexpression of Tfap2C suppesses PrPSc levels. These effects on PrPSc are independent of PrPC levels. By RNAseq analysis, the authors hone in on metabolic pathways regulated by HNRPNK and Tfap2C, then follow the data to autophagy regulation by mTor. Ultimately, the authors show that short-term treatments of these cell models with mTor inhibitors causes increased accumulation of PrPSc. The authors conclude that the loss of HNRNPK leads to a reduced energy metabolism causing mTor inhibition, which is reduces translation by dephosphorylation of S6.

Major comments:

1) Fig H and I, Fig 3L. The interaction between Tfap2C and HNRNPK is pretty weak. The interaction may not be consequential. The experiment seems to be well controlled, yielding limited interaction. The co-ip was done in PBS with no detergent. The authors indicate that the cells were mechanically disrupted. Since both of these are DNA binding proteins, is it possible that the observed interaction is due to the proximity on DNA that is linking the 2 proteins, including a DNAase treatment would clarify.

We agree that the observed co-IP between Tfap2c and hnRNP K is weak (previous Fig. 2H-I, Supp. Fig. 3L now shifted in Supp. Fig. 4C-E), and we have now highlighted this in the relevant section of the manuscript to reflect this observation better.
Importantly, the co-IP was performed using endogenous proteins without overexpression or tagging, which can sometimes artificially enhance protein-protein interactions. However, we acknowledge that the use of a detergent-free lysis buffer and mechanical disruption alone may have limited nuclear protein extraction and solubilization, potentially contributing to the low co-IP signal.
To address the reviewer's concerns and clarify whether the observed interaction could be DNA-mediated, we repeated the co-IP experiments under low-detergent conditions and included benzonase nuclease treatment to digest nucleic acids (Fig. 2H-I). DNA digestion was confirmed by agarose gel electrophoresis (Supp. Fig. 4F-G). Additionally, we performed the reciprocal IPs using both hnRNP K and Tfap2c antibodies (Fig. 2H-I). Although the level of co-immunoprecipitation remains modest, these updated experiments continue to demonstrate a specific co-immunoprecipitation between Tfap2c and hnRNP K, independent of DNA bridging. These additional controls and experimental refinements strengthen the validity of our findings. These results are also attached here for your convenience.

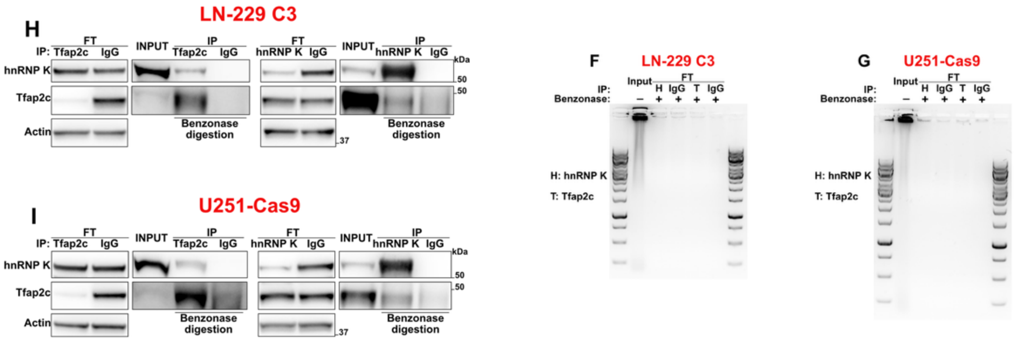

2) Supplemental Fig 5B - The western blot images for pAMPK don't really look like a 2 fold increase in phosphorylation in HNRNPK deletion.

We thank the reviewer for raising this point. We re-examined the original pAMPK western blot (previously Supp. Fig. 5B; now presented as Supp. Fig. 6B) and confirmed the reported results. We note that the overall loading is not perfectly uniform across lanes (as suggested by the actin signal), which may affect the visual impression of band intensity. However, the phosphorylation change reported in the manuscript is based on the pAMPK/total AMPK ratio, which accounts for differences in AMPK expression and accurately reflects relative phosphorylation levels. To further address this concern, we performed three additional independent experiments. These new data reproduce the increase in pAMPK/AMPK upon HNRNPK deletion and are now included in the revised Supplementary Fig. 6B, together with the updated quantification. The new blot and the quantification are also attached here for your convenience.

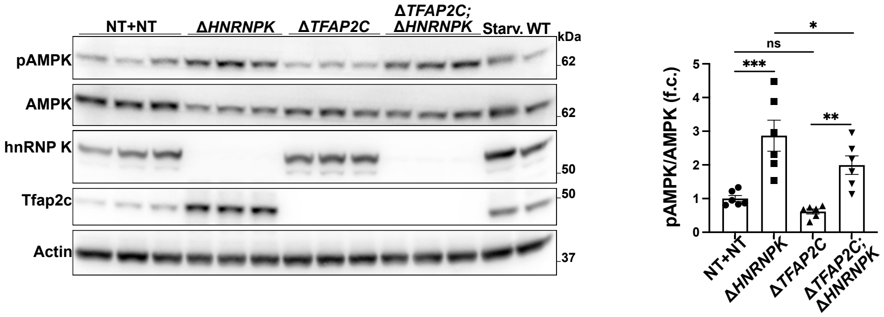

3) Fig. 5A - I don't think it is proper to do statistics on an of 2.

We believe the reviewer's comment refers to Fig. 5B, as Fig. 5A already has sufficient replication. We have now added two additional replicates, bringing the total to four. The updated statistical analysis corroborates our initial results. The new quantification is provided in the revised manuscript (Fig. 5B) along with the new blot (Supp. Fig. 6C). Both data are also attached here for your convenience.

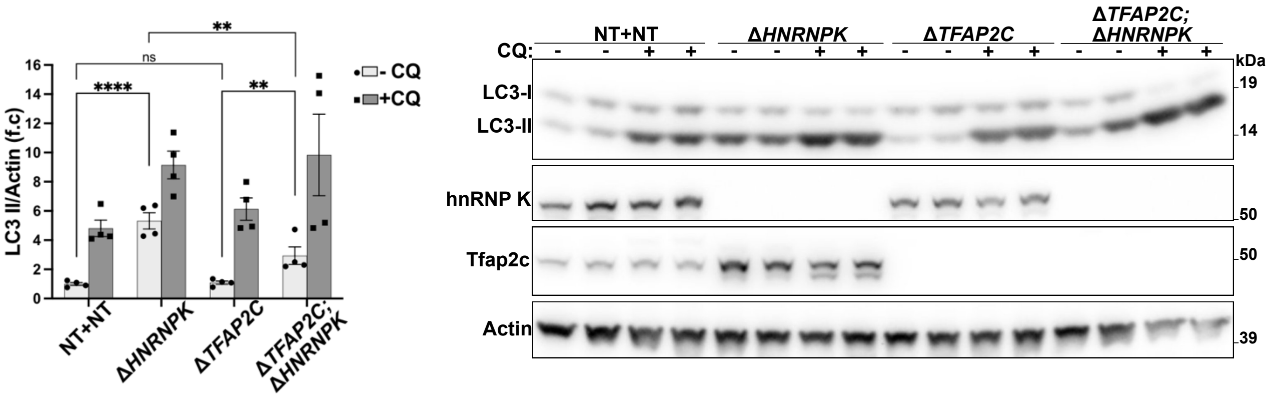

4) Fig 6D. The data look a bit more complicated than described in the text. At 7 days, compared to 2 days, it looks like there is a decrease in % cells positive for 6D11. Is there clearance of PrPSc or proliferation of un-infected cells?

We have now reworded our text in the results paragraph as follows:
"These data show that TFAP2C overexpression and HNRNPK downregulation bidirectionally regulate prion levels in cell culture."
We have now also included the following comments in the discussion section:
"However, prion propagation relies on a combination of intracellular PrP^{Sc} seeding and amplification, as well as intercellular spread, which together contribute to the maintenance and expansion of infected cells within the cultured population. In this study, we were limited in our ability to dissect which specific steps of the prion life cycle are affected by TFAP2C. We also cannot fully exclude the possibility that TFAP2C overexpression influenced the relative proliferation of prion-infected versus uninfected cells in the PG127-infected HovL culture, thereby contributing to the observed reduction in the percentage of $6D11^+$ cells and overall $6D11^+$ fluorescence. However, we did not observe any signs of cell death, growth impairment, or increased proliferation under TFAP2C overexpression in PG127-infected HovL cells compared to NBH controls (data not shown). This suggests that a negative selective pressure on infected cells or a proliferative advantage of uninfected cells is unlikely in this context".

5) The authors might consider a different order of presenting the data. Fig 6 could follow Fig. 2 before the mechanistic studies in Figs 3-5.

We believe that the current order of presenting the data is more appropriate. The first part of the manuscript focuses on the genetic and functional interactions between hnRNP K and its partners, particularly TFAP2C, which is a critical point for understanding the broader context before delving into the mechanistic studies involving prion-infected cells.

6) The authors use SEM throughout the paper and while this is often used, there has been some interest in using StdDev to show the full scope of variability.

We chose to use SEM as it reflects the precision of the mean, which is central to our statistical comparisons. As the reviewer notes, this is a common and appropriate practice. To address variability, almost all graphs already include individual data points, which provide a direct visual representation of data spread. To further enhance clarity, we have now included StdDev in the Supplementary Source Data table of the revised manuscript.

Discussion:

The discrepancy between short-term and long-term treatments with mTor inhibitors is only briefly mentioned with a bit of a hand-waving explanation. The authors may need a better explanation.

We have now integrated a more detailed explanation in the discussion section of the revised manuscript as follows:

"Previous studies showed that mTORC1/2 inhibition and autophagy activation generally reduce, rather than increase, PrP^{Sc} aggregation (79, 80). The reason for this discrepancy remains unclear and may be multifactorial. First, most prior studies were based on long-term mTOR inhibition, whereas our work examined acute inhibition, mimicking the time frame of HNRNPK and TFAP2C manipulation. Acute inhibition may trigger transient metabolic or signaling shifts that differ from adaptive changes associated with mTOR chronic inhibition, potentially overriding autophagy's effects on prion propagation. Additionally, while previous works were primarily conducted in murine in vivo models, our study focused on a human cell system propagating ovine prions. Differences in species background, model complexity (e.g., interactions between different cell types), and prion strain variability, as certain strains exhibit distinct responses to autophagy and mTOR modulation (https://doi.org/10.1371/journal.pone.0137958), likely contributed to the observed differences".

Minor comments:

Page 12 - no mention of chloroquine in the text or related data.
Page 12 - Supp. Fig. E - should be 5E

We thank the reviewer for pointing this out. We have now better highlighted the use of chloroquine in Fig. 5B (see reviewer #1 - Point 3 – Major comments) and in the text as follows:
"Furthermore, in the presence of chloroquine, LC3-II levels rose almost proportionally across all conditions (Fig. 5B), suggesting that the effects of HNRNPK and TFAP2C on autophagy occur at the level of autophagosome formation, rather than autophagosome-lysosome fusion and degradation."

We have corrected the reference to Supp. Fig. 5E.

Reviewer #1 (Significance (Required)):

The study provides mechanistic insight into how HNRNPK modulates prion propagation. The paper is limited to cell models, and the authors note that long term treatment with mTor inhibitors reduced PrPSc levels in an in vivo model.

The primary audience will be other prion researchers. There may be some broader interest in the mTor pathway and the role of HNRNPK in other neurodegenerative diseases.

Reviewer #2 (Evidence, reproducibility and clarity (Required)):

The manuscript "Prion propagation is controlled by a hierarchical network involving the nuclear Tfap2c and hnRNP K factors and the cytosolic mTORC1 complex" by Sellitto et al aims to examine how heterogenous nuclear ribonucleoprotein K (hnRNPK), limits pion propagation. They perform a synthetic - viability CRISPR- ablation screen to identify epistatic interactors of HNRNPK. They found that deletion of Transcription factor AP-2g (TFAP2C) suppressed the death of hnRNP-K depleted LN-229 and U-251 MG cells whereas its overexpression hypersensitized them to hnRNP K loss. Moreover, HNRNPK ablation decreased cellular ATP, downregulated genes related to lipid and glucose metabolism and enhanced autophagy. Simultaneous deletion of TFAP2C reversed these effects, restored transcription and alleviated energy deficiency. They state that HNRNPK and TFAP2C are linked to mTOR signalling and observe that HNRNPK ablation inhibits mTORC1 activity through downregulation of mTOR and Rptor while TFAP2C overexpression enhances mTORC1 downstream functions. In prion infected cells, TFAP2C activation reduced prion levels and countered the increased prion propagation due to HNRNPK suppression. Pharmacological inhibition of mTOR also elevated prion levels and partially mimicked the effects of HNRNPK silencing. They state their study identifies TFAP2C as a genetic interactor of HNRNPK and implicates their roles in mTOR metabolic regulation and establishes a causative link between these activities and prion propagation.
This is an interesting manuscript in which a lot of work has been undertaken. The experiments are on the whole well done, carefully documented and support most of the conclusions drawn. However, there are places where it was quite difficult to read as some of the important results are in the supplementary Figures and it was necessary to go back and forth between the Figs in the main body of the paper and the supplementary Figs. There are also Figures in the supplementary which should have been presented in the main body of the paper. These are indicated in our comments below.
We have the following questions /points:

Major comments:

1) A plasmid harbouring four guide RNAs driven by four distinct constitutive promoters is used for targetting HNRNPK- is there a reason for using 4 guides- is it simply to obtain maximal editing - in their experience is this required for all genes or specific to HNRNPK?

The use of four guide RNAs driven by distinct promoters is chosen to maximize editing efficiency for HNRNPK. As previously demonstrated by J. A. Yin et al. (Ref. 32), this system provides better efficiency for gene knockout (or activation). For HNRNPK, achieving full knockout was crucial for observing a complete lethal phenotype, which made the four guide RNAs approach fundamental. However, other knockout systems, while potentially less efficient, have been shown to work well in other circumstances. We have now included this explanation in the revised manuscript as follows:
"We employed a plasmid harboring quadruple non-overlapping single-guide RNAs (qgRNAs), driven by four distinct constitutive promoters, to target the human HNRNPK gene <u>and maximize editing efficiency</u> in polyclonal LN-229 and U-251 MG cells stably expressing Cas9 (32)."

2) Is there a minimal amount of Cas9 required for editing?

We did not observe a correlation between Cas9 levels and activity, yet the C3 clone was the one with higher Cas9 expression and higher activity (Supp. Fig. 1A-B). We agree that comments about the amount of Cas9 expression may be misleading here. Thus, in the first result paragraph of the revised manuscript, we have now modified the text "*we isolated by limiting dilutions LN-229 clones expressing high Cas9 levels*" to "*we isolated by limiting dilutions LN-229 single-cell clones expressing Cas9*".

3) It is stated that cell death is delayed in U251-MG cells compared to LN-229-C3 cells- why? Also, why use glioblastoma cells other than that they have high levels of HNRNPK? Would neuroblastoma cells be more appropriate if they are aiming to test for prion propagation?

As shown in Fig. 1A, U251-MG cells reached complete cell death at day 13, while LN-229 C3 reached it already at day 10. The percentage of viable U251-MG cells is higher (statistically significant) than LN-229 C3 cells at all time points before day 13, when both lines show complete death. The underlying reasons for this partial and relative resistance are probably multiple, but we clearly showed in Fig. 2 that TFAP2C differential expression is one modulator of cell sensitivity to HNRNPK ablation.

We selected glioblastoma cells because their high expression of HNRNPK was essential for developing our synthetic lethality screen strategy, and we have now clarified it in the revised manuscript as follows:

"As model systems, we chose the human glioblastoma-derived LN-229 and U-251 MG cell lines, which express high levels of HNRNPK (2, 3), a key factor for optimizing our synthetic lethality screen."

While neuroblastoma cells might be more relevant in terms of prion neurotoxicity, glial cells, despite their resistance to prion toxicity, are fully capable of propagating prions. Prion propagation in glial cells has been shown to play crucial roles in mediating prion-dependent neuronal loss in a non-autonomous manner (see 10.1111/bpa.13056). This makes glioblastoma cells a valuable model for studying prion propagation (that is the focus of our study), despite the lack of direct toxicity (which is not the focus of our study). We have now added this explanation to the revised manuscript as follows:
"Therefore, we continued our experiments using LN-229 cells, which provide a relevant model for studying prions, as glial cells can propagate prions and contribute to prion-induced neuronal loss through non-cell-autonomous mechanisms."

4) Human CRISPR Brunello pooled library- does the Brunello library use constructs which have four independent guide RNAs as used for the silencing of HNRPNK?

No, the Human CRISPR Brunello pooled library does not use constructs with four independent guide RNAs (qgRNAs). Instead, each gene is targeted by 4 different single-guide RNAs (sgRNAs), each expressed on a separate plasmid. We have now clarified this in the main text of the revised manuscript as follows:
"To identify functionally relevant epistatic interactors of HNRNPK, we conducted a whole-genome ablation screen in LN-229 C3 cells using the Human CRISPR Brunello pooled library (33), which targets 19,114 genes with an average of four distinct sgRNAs per gene, each expressed by a separate plasmid (total = 76,441 sgRNA plasmids)."

5) To rank the 763 enriched genes, they multiply the -log10FDR with their effect size - is this a standard step that is normally undertaken?

The approach of ranking hits using the product of effect size and statistical significance is a well-established method in CRISPR screening studies. This strategy has been explicitly used in high-impact work by Martin Kampmann and others (see https://doi.org/10.1371/journal.pgen.1009103 and https://doi.org/10.1016/j.neuron.2019.07.014 as references). We have now added both references to the revised manuscript.

6) The 32 genes selected- they were ablated individually using constructs with one guide RNA or four guide RNAs?

The 32 genes selected were ablated individually using constructs with quadruple-guide RNAs (qgRNAs), as this approach was intended to maximize editing efficiency for each gene. We have now clarified this in the main text of the revised manuscript as follows:
"We ablated each gene individually using qgRNAs and then deleted HNRNPK."

7) The identified targets were also tested in U251-MG cells and nine were confirmed but the percent viability was variable - is the variability simply a reflection of the different cell line?

The variability in percent viability observed in U251-MG cells likely reflects the inherent differences between cell lines, which can contribute to varying levels of susceptibility to gene ablation, even for the same targets. We have now highlighted these small differences in the main text of the revised manuscript as follows:
"We confirmed a total of 9 hits (Fig. 1H), including the ELPs gene IKBAKP and the transcription factor TFAP2C, the two strongest hits identified in LN-229 C3 cells. However, in the U251-Cas9 the rescue effect did not always fall within the exact range observed in LN-229 C3 cells, likely due to intrinsic differences between the two cell lines."

8) The two strongest hits were IKBAKP and TFAP2C. As TFAP2C is a transcription factor - is it known to modulate expression of any of the genes that were identified to be perturbed in the screen? Moreover, it is stated that it regulates expression of several lncRNAs- have the authors looked at expression of these lncRNAs- is the expression affected- can modulation of expression of these lncRNAs modulate the observed phenotypic effects and also some of the targets they have identified in the screen?

While TFAP2C is a transcription factor known to regulate the expression of several genes and lncRNAs, we did not identify any of its known target genes among the hits of our screen. However, our RNA-seq data and RT-qPCR (data not shown) indicate that the expression of lncRNA MALAT1 and NEAT1 (reported to interact with both HNRNPK and TFAP2C; ref 37, 41, 47) is strongly affected by HNRNPK ablation and to a lesser extent by TFAP2C deletion. However, the double deletion condition does not appear to change these lncRNA levels beyond what is observed with HNRNPK ablation alone. Therefore, we concluded that these changes do not play a primary role in the phenotypic effects observed in our study. Thus, although interesting, we believe that the description of such observations goes beyond the scope of this manuscript and the relevance of this work.

9) As both HNRNPK and TFAP2C modulate glucose metabolism, the authors have chosen to explore the epistatic interaction. This is most reasonable.

We do not have further comments on this point.

10) The orthogonal assay to confirm that deletion of TFAP2C supresses cell death upon removing HNRNPK- was this done using a single guide RNA or multiple guides - is there a level of suppression required to observe rescue? Interestingly ablation of HNRNPK increases TFAP2C expression in LN-229-C3 whereas in U251-Cas9 cells HNRNPK ablation has the opposite effect- both RNA and protein levels of TFAP2C are decreased - is this the cause of the smaller protective effect of TFAP2C deletion in this cell line?

TFAP2C deletion was performed using quadruple-guide RNAs (gqRNAs). We have clarified this point by addressing the reviewer #2's point 6 in "Major comments".
We did not directly test the threshold of TFAP2C inhibition required to suppress HNRNPK ablation-induced cell death. We did not exclude that other effectors may take a role in the smaller protective effect of TFAP2C deletion in the U251-Cas9 cells, however, multiple lines of evidence from our study suggest that TFAP2C expression levels influence cellular sensitivity to HNRNPK loss:
1) Both LN-229 C3 and U251-Cas9 cells are less sensitive to HNRNPK ablation upon TFAP2C deletion (Fig. 1G-H, Fig. 2A-B, Supp. Fig.3A-B).
2) We observed a correlation between endogenous TFAP2C levels and HNRNPK ablation sensitivity. U251-Cas9 cells, where TFAP2C expression is reduced upon HNRNPK ablation (in contrast to LN-229 C3 cells, where HNRNPK ablation leads to an increase in TFAP2C expression) (Fig. 2C-F), are a) less sensitive to HNRNPK deletion than LN-229 C3 (Fig. 1A, 2A-B) and b) the protective effect of TFAP2C deletion is less pronounced than in LN-229 C3 (Fig. 1G-H, Fig. 2A-B, Supp. Fig.3A-B).
3) TFAP2C overexpression experiments (Fig. 2G) establish a causal relationship to the former correlation: TFAP2C overexpression increased U251-Cas9 sensitivity to HNRNPK ablation.
As clearly mentioned in the manuscript, we believe that, taken together, these findings strongly demonstrate a causal role for TFAP2C in modulating sensitivity to HNRNPK loss. Thus, despite the differences in the expression, the proposed viability interaction between TFAP2C and HNRNPK is conserved across cell lines.
To further strengthen our conclusions, we have now added LN-229 C3 TFAP2C overexpression in Fig. 2G (also attached below for your convenience). As for the U251-Cas9, LN-229 C3 cells show increased sensitivity to HNRNPK ablation upon TFAP2C overexpression.

Review COMMONS

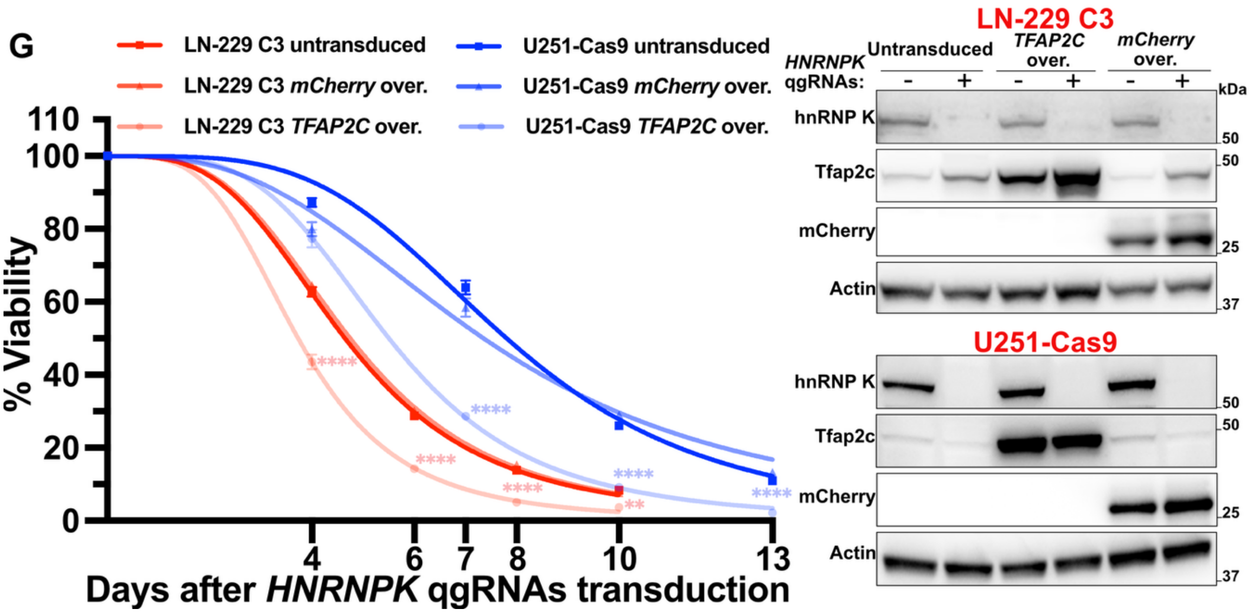

11) Nuclear localisation studies indicate that the HNRNPK and TFAP2C proteins colocalise in the nucleus however the co-IP data is not convincing- although appropriate controls are present, the level of interaction is very low - the amount of HNRNPK pulled down by TFAP2C is really very low in the LN-229C3 cells and even lower in the U251-Cas9 cells. Have they undertaken the reciprocal co-IP expt?

We rephrased our text to better highlight this as also mentioned in our response to reviewer #1 (Point 1 - Major comments). However, as also noted by the reviewer, the experiments included all the relevant controls. Thus, the results are solid and confirm a degree of co-immunoprecipitation (although weak). As detailed in our response to reviewer #1 (Point 1 - Major comments), to strengthen our conclusion, we have now repeated the experiment in low-detergent conditions and used benzonase nuclease for DNA digestion. We also have performed the reciprocal experiment as suggested by the reviewer, confirming the initial results. In our opinion, these additional experiments support the conclusion that Tfap2c and hnRNP K co-immunoprecipitate through a weak, but direct, interaction.

12) They state that LN-229 C3 ΔTFAP2C and U251-Cas9 ΔTFAP2C were only mildly resistant to the apoptotic action of staurosporin Fig 3E and F - I accept they have undertaken the stats which support their statement that at high concentrations of staurosporin the LN-229 C3 ΔTFAP2C cells are less sensitive but the U251-Cas9 ΔTFAP2C decreased sensitivity is hard to believe. Has this been replicated? I agree that HNRNPK deletion causes apoptosis in both LN-229 C3 and U251-Cas9 cells and this is blocked by Z-VAD-FMK - however the block is not complete- the max viability for HNRNPK deletion in LN-229 C3 cells is about 40% whereas for U251-Cas9 cells it is

about 30% - does this suggest that cells are being lost by another pathway. Have they tested concentrations higher than 10nM?

The experiments in FIG. 3E-F have been replicated four times, as stated in the figure legend. We agree that TFAP2C plays a limited role in response to staurosporine-induced apoptosis, particularly in U251-Cas9 cells. To ensure clarity, we have now modified our previous sentence as follows:
"LN-229 C3$^{\Delta TFAP2C}$ cells were only mildly resistant to the apoptotic action of staurosporine, and U251-Cas9$^{\Delta TFAP2C}$ showed even lower and minimal recovery (Fig. 3E-F). These results indicate that TFAP2C plays a limited role in apoptosis regulation and suggest that its suppressive effect on HNRNPK essentiality is not mediated through direct modulation of apoptosis but rather through upstream processes that eventually converge on it."

The incomplete blockade of apoptosis by Z-VAD-FMK suggests that HNRNPK ablation may activate alternative, non-caspase-mediated cell death pathways. Regarding this point, we decided to not test Z-VAD-FMK above 10 nM as we noted that the rescue effect at the lowest concentration (2nM) was not proportionally increasing at higher concentrations, suggesting we already reached saturation. We have now added and clarified these observations in the revised manuscript as follows:
"Z-VAD-FMK decreased cell death consistently and significantly in LN-229 C3 and U251-Cas9 cells transduced with HNRNPK ablation qgRNAs (Fig. 3C-D), confirming that HNRNPK deletion promotes cell apoptosis. However, we observed that viability recovery plateaued already at the lowest concentration (2 nM) without further increase at higher doses, suggesting a saturation effect. This indicates that while caspase inhibition alleviates part of the cell death, HNRNPK loss triggers additional mechanisms beyond apoptosis".

Following the suggestion of the reviewer, we have now also tested two higher concentrations of Z-VAD (20 and 50nM) in LN-229 cells. At these concentrations, we observed a slight decrease in cell viability in the NT condition, with a rescue effect in the HNRNPK-ablated cells comparable to what was observed at 2-10nM Z-VAD. For this reason, we did not include these data in the revised manuscript, and we attached them here for transparency.

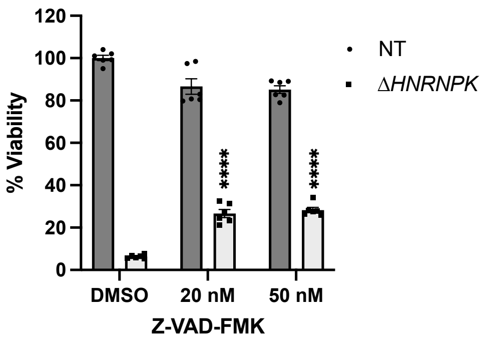

13) The RNA-seq comparisons- the authors use log2 FC <0.5 upregulated or genes downregulated by a similar amount- this is a very low cut off and would include essentially minimal changes in expression - not convinced of the significance of such low-level changes.

We used a log2 FC threshold of >0.5 and < -0.5, not <0.5 (there was a typo in the main text, which we have now fixed). This threshold represents moderate but still relevant changes (over 40% relative difference) in individual gene expression. Such a threshold (or even lower, <-0.25 and >0.25) is commonly used in RNA-seq studies to capture biologically relevant shifts (e.g., https://doi.org/10.1371/journal.ppat.1012552; https://doi.org/10.1371/journal.ppat.1008653; https://doi.org/10.1016/j.neuron.2025.03.008; https://doi.org/10.15252/embj.2022112338). We complemented this analysis with Gene Set Enrichment Analysis (GSEA) to assess coordinated changes in biological/genetic pathways, ensuring that our conclusions are not based on isolated, minor expression changes nor on arbitrary thresholds. Finally, to enhance our result robustness, we applied False Discovery Rate (FDR) statistics, which is more stringent than a p-value cutoff. We hope this clarification strengthens the reviewer's confidence in the significance of the observed changes.

14) It is stated" Accordingly, we observed increased AMPK phosphorylation (pAMPK) upon ablation of HNRNPK, which was consistently reduced in LN-229 C3ΔTFAP2C cells (Supp. Fig. 5B). LN-229 C3ΔTFAP2C; ΔHNRNPK cells also showed a partial reduction of pAMPK relative to LN-229 C3ΔHNRNPK cells (Supp. Fig. 5B). These results suggest that hnRNP K depletion causes an energy shortfall, leading to cell death.
I am not totally convinced by the data presented in this Fig. The authors have quantified the band intensity and present the ratio of pAMPK to AMPK. Please note that the actin levels are variable across the samples - did they normalise the data using the actin level before undertaking the comparisons? Also, if the authors think this is an important point which supports their conclusion, then it should be in the main body of the paper rather than the supplementary. If AMPK is being phosphorylated, this should lead to activation of the metabolic check point which involves p53 activation by phosphorylation. Activated p53 would turn on p21CIP1 which is a very sensitive indicator of p53 activation.

We also refer the reviewer to our response to reviewer #1 (Point 2 – Major comments). We understand the point of the reviewer as pAMPK/Actin (absolute AMPK phosphorylation) may provide additional context regarding the downstream effects of AMPK activation, which, however, is not the primary scope of our experiment. We believe that in our specific case, a) the pAMPK/AMPK ratio is the most appropriate metric, as it reflects the energy status of the cell (ATP/AMP levels), which was our main point to assess in this experiment, and b) phospho-protein/total protein is the standard approach for quantifying phosphorylation ratio. For completeness, we have now included pAMPK/Actin quantifications in Supp. Fig. 6B of the revised manuscript (also attached below). pAMPK/Actin levels follow the same trend of pAMPK/AMPK in HNRNPK and TFAP2C single ablations. The pAMPK/AMPK partial rescue in HNRNPK;TFAP2C double ablation relative to HNRNPK single deletion is instead not observed at pAMPK/Actin level.

We have now added the pAMPK/Actin quantification and this observation to the revised manuscript as follows:

"*Accordingly, we observed increased AMPK phosphorylation (pAMPK/AMPK ratio and pAMPK/Actin) upon ablation of HNRNPK, with a trend toward reduction in LN-229 C3$^{\Delta TFAP2C}$ cells (Supp. Fig. 6B). LN-229 C3$^{\Delta TFAP2C;\Delta HNRNPK}$ cells also showed a reduction of pAMPK/AMPK ratio relative to LN-229 C3$^{\Delta HNRNPK}$ cells, although absolute AMPK phosphorylation (pAMPK/Actin) remained high (Supp. Fig. 6B).*"

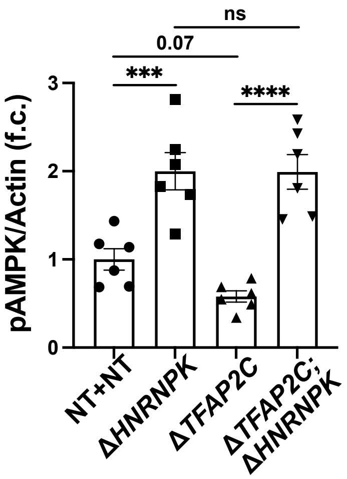

We prefer to keep the AMPK blots in Supplementary Fig. 6B, as we believe the main take-home message of the manuscript should remain centered on mTORC1 activity.

15) We also do not understand why the mTOR Suppl. Fig. 5E is not in the main body of the paper. It's clear that RNA and protein levels of mTOR were downregulated in LN-229 C3ΔHNRNPK cells but were partially rebalanced by the ΔTFAP2C- however the ΔTFAP2C;ΔHNRNPK double deletion levels are only slightly higher than the ΔHNRNPK - they are not at the level NT or even ΔTFAP2C (Fig. 4C, Supp. Fig. 5E).

We moved the mTOR blot to Fig.5D of the revised manuscript. About the low rescue effect, this is in line with all the other observations where a full rescue of the effects of HNRNPK ablation is never achieved, but is only partial. As suggested by reviewer #3 (Figure 5 – Point 2), we have now added RT-qPCR in Fig.5C, which corroborates these data.

16) The authors state: "Deletion of HNRNPK diminished the highly phosphorylated forms of 4EBP1, which instead were preserved in both LN-229 C3ΔTFAP2C and LN-229 C3ΔTFAP2C;ΔHNRNPK cells (Fig. 5C). Similarly, the S6 phosphorylation ratio was reduced in LN-229 C3ΔHNRNPK cells and was restored in the ΔTFAP2C;ΔHNRNPK double-ablated cells (Fig. 5C)."

WE are not convinced that p4EBP1 is preserved in the LN-229 C3ΔTFAP2C cells - there is a very faint band which is at a lower level than the band in the LN-229 C3ΔHNRNPK cells. However, when both HNRNPK and TFAP2C were ablated, the p4EBP1 band is clear cut. I agree with the quantitation that deletion of HNRNPK and TFAP2C both reduce the level of 4EBP1 - the reduction is greater with TFAP2 but when both are deleted together the levels of 4EBP1 are higher and p4EBP1 is clearly present. In quantifying the S6 and pS6 levels, did the authors consider the actin levels- they present a ratio of the pS6 to S6. I may be lacking some understanding but why is the ratio of pS6/S6 being calculated. Is the level of pS6 not what is important - phosphorylation of S6 should lead it to being activated and thus it's the actual level of pS6 that is important, not the ratio to the non-phosphorylated protein.

In Fig. 5C, the three-band pattern of 4EBP1 is clearly visible in the NT+NT or WT condition, with the top band representing the highest phosphorylation state. Upon HNRNPK deletion, this top band almost completely disappears, mimicking the effect of our starvation control (Starv.). This top band remains clearly visible in both TFAP2C-ablated and double-ablated cells, supporting our conclusion. In our original text, we referred to the "highly phosphorylated forms" of 4EBP1, which might have caused some confusion, suggesting we were evaluating the two top bands. We are specifically referring only to the very top band (high p4EBP1), which represents the most highly phosphorylated form of 4EBP1. This is the relevant phosphorylated form to focus on, as it is the only one that disappears in the starvation control (Starv.) or upon mTORC1/2 inhibition with Torin-1 (Fig. 7B).
To better clarify these points, we have now more clearly indicated the "high p4EBP1" band with an asterisk in Fig. 5E, added quantification of high p4EBP1/4EBP1, and rephrased the text as follows:
"Deletion of HNRNPK diminished the highest phosphorylated form of 4EBP1 (high p4EBP1, marked with an asterisk), mimicking the effect observed in starved cells (Starv.). This high p4EBP1 band was preserved in both LN-229 C3$^{\Delta TFAP2C}$ and LN-229 C3$^{\Delta TFAP2C;\Delta HNRNPK}$ cells (Fig. 5C).".

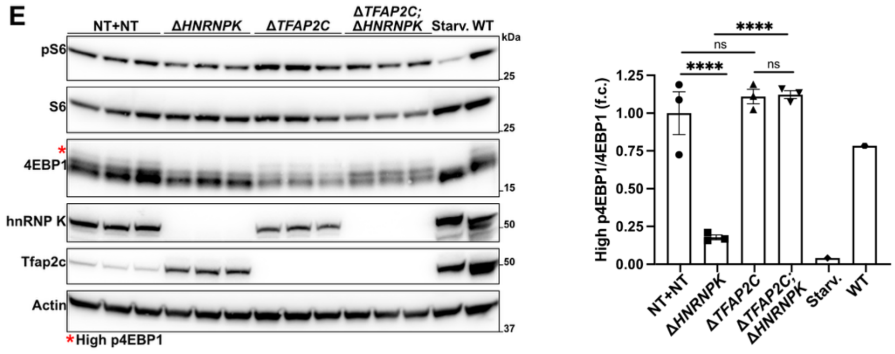

Regarding pS6 quantification, we added pS6/Actin quantification in Supp. Fig. 6E and F of the revised manuscript, also attached here for your convenience.

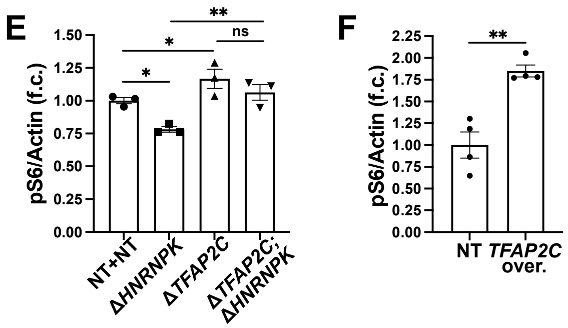

17) When determining ATP levels, do they control for cell number? HNRNPK depletion results in lower ATP levels, co-deletion of TFAP2C rescues this. But this could be because there is less cell-death? So, more cells express ATP. Have they controlled for relative numbers of cells.

As described in the Materials and Methods, we normalized ATP levels to total protein content, which is a standard approach for this type of quantification (see DOI:10.1038/nature19312).

18) The construction of the HovL cell line that propagate ovine prions - very few details are provided of the susceptibility of the cell line to PG127 prions.

As with other prion-infected cell lines, HovL cells do not exhibit any specific growth defects, susceptibilities, or phenotypes beyond their ability to propagate prions. This is consistent with established observations in prion research, where immortalized cell lines (and in general in vitro cultures) normally do not show cytotoxicity upon prion infection and, therefore, are used as models for prion propagation rather than for prion toxicity (see https://doi.org/10.1111/jnc.14956 for reference).
We now expanded the relevant section, including technical and conceptual details in the main text of the revised manuscript as follows:
"As reported for other ovinized cell models (*66*), HovL cells were susceptible to infection by the PG127 strain of ovine prions and capable of sustaining chronic prion propagation, as shown by proteinase K (PK)-digested western blot and by detection of PrP^{Sc} using the anti-PrP antibody 6D11, which selectively stains prion-infected cells after fixation and guanidinium treatment (*67*) (Supp. Fig. 7C-E). Consistent with most prion-propagating cell lines (*68*), HovL cells did not exhibit specific growth defects, susceptibilities, or overt phenotypes beyond their ability to propagate prions."

19) It is stated that HRNPK depletion from HovL cells increases PrpSC as determined by 6D11 fluorescence, but in the manuscript HRNPK depletion results in cell death. How does this come together?

As explicitly stated in the main text and shown in Fig.6-7, HNRNPK is downregulated (via siRNAs) in the prion experiments rather than fully deleted (via CRISPR) as in the first part of the manuscript. As shown in Supp. Fig. 8B, this downregulation does not affect cell viability within the experimental time window. Therefore, the observed increase in PrP^{Sc} levels upon HNRNPK downregulation, as determined by western blot and 6D11 staining, is independent of any potential cell death effects. Moreover, the same siRNA downregulation approach was used by M. Avar et al. (Ref. 26) in comparable experiments, yielding similar outcomes.

20) They show that mTOR inhibition mimics the effect of HNRNPK deletion, why didn't they overexpress mTOR and see if that rescues this? This would indicate a causal relationship.

We appreciate the reviewer's suggestion. We agree that the proposed rescue strategy would be the best approach to indicate a causal relationship. However, we linked the activity of the mTORC1 complex (and not only that of mTOR) to prion propagation. Overexpression of only mTOR would not restore mTORC1 full function, as Rptor would still be downregulated in the context of HNRNPK siRNA silencing (Fig. 7A and Supp. Fig. 8E). Moreover, our RNA-seq data (Supp. Table 5) from HNRNPK ablation indicate the downregulation of other mTORC1 components (namely Pras40 (AKT1S1) and mLST8). Therefore, the rescue of the mTORC1 activity by an overexpression strategy would be a very challenging approach. Given these complexities, to infer causality, we used mTORC1 inhibition (via rapamycin and Torin1) to mimic the effects of HNRNPK downregulation in reducing mTORC1 activity (FIG. 7B).
For clarification, we have now highlighted in Fig. 4C that HNRNPK downregulates also AKT1S1 and mLST8, other than mTOR and Rptor (also attached below), and we have discussed this in the main text as well. We also have clarified in the revised manuscript (where we sometimes inadvertently referred to it as just mTOR inhibition) that the observed effects are due to mTORC1 inhibition, and not simply mTOR inhibition.

C

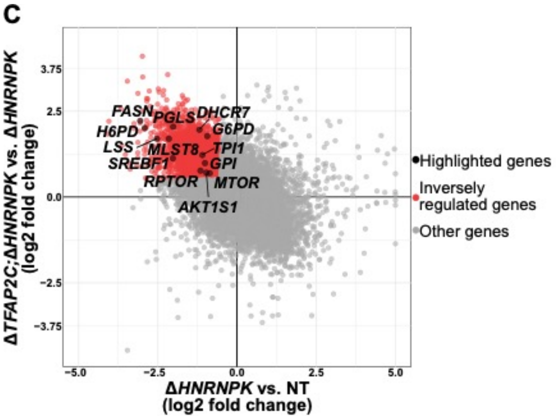

21) Flow cytometric data: supplementary Fig of Fig6d. - when they are looking at fixed cells the gating strategy for cells results in the inclusion of a lot of debris. The gate needs to be moved and

Review COMMONS

be more specific to ensure results are interpreted properly. Same with the singlet gating. It's not tight enough, they include doublets as well which will skew their data. The gating strategy needs to be regated.

We have reanalyzed the flow cytometry data in Fig. 6D with a more stringent gating approach to better exclude debris and ensure proper singlet selection. We confirm that there is no change in the final interpretation of the results after applying the updated gating strategy.

Reviewer #2 (Significance (Required)):

The manuscript "Prion propagation is controlled by a hierarchical network involving the nuclear Tfap2c and hnRNP K factors and the cytosolic mTORC1 complex" by Sellitto et al aims to examine how heterogenous nuclear ribonucleoprotein K (hnRNPK), limits pion propagation. They perform a synthetic - viability CRISPR- ablation screen to identify epistatic interactors of HNRNPK. They found that deletion of Transcription factor AP-2g (TFAP2C) suppressed the death of hnRNP-K depleted LN-229 and U-251 MG cells whereas its overexpression hypersensitized them to hnRNP K loss. Moreover, HNRNPK ablation decreased cellular ATP, downregulated genes related to lipid and glucose metabolism and enhanced autophagy. Simultaneous deletion of TFAP2C reversed these effects, restored transcription and alleviated energy deficiency.

Reviewer #3 (Evidence, reproducibility and clarity (Required)):

Summary:
Using a CRISPR-based high throughput abrasion assay, Sellitto et al. identified a list of genes that improve cell viability when deleted in hnRNP K knockout cells. Tfap2c, a transcription factor, was identified as a candidate with potential overlap with a hnRNP K function like modulating glucose metabolism. The deletion of Tfap2c in hnRNP K-deletion background prevented caspase-dependent apoptosis observed in hnRNP K single-deletion cells. Further analysis of bulk RNA-seq in hnRNP K/TFAP2C single- and double-deletion cells revealed the impairment in cellular ATP level. Accordingly, activation of AMPK led to perturbed autophagy in hnRNP K deleted cells. Moreover, the reduction and/or inactivation of the downstream mTOR protein resulted in the reduced phosphorylation of S6. Conversely, the phosphorylation of S6 and E4BP1 can be increased by TFAP2C overexpression. Finally, the pharmacological inhibition of the mTOR pathway increased the PrPSC level. This is an interesting paper potentially providing new mechanical insight of hnRNPK function and its interaction with TFAP2C. However, inconsistencies in TFAP2C expression across cell lines and conflicting mechanistic interpretations complicate conclusions. Co-IP experiments suggested hnRNP K and Tfap2c may interact, though further validation is needed. Several figures require additional clarification, statistical analysis, or experimental validation to strengthen conclusions.

Major comments:

1) Different responses of the TFAP2C expression level to deletion of hnRNPK in the two cell lines (LN-229 C3 and U251-Cas9) should be more adequately addressed. The manuscript focuses on the interaction between hnRNPK and TFAP2C, yet the hnRNPK deletion causes different changes in TFAP2C level in two different lines. Furthermore, in studies where the mechanistic link between hnRNPK and TFAP2C is being investigated, only results from the LN-229 line are presented (Figure 4-7). Thus, it is not clear whether these mechanisms also apply to another line, U251-Cas9, where hnRNPK deletion has the opposite effect on the TFAP1C level. Thus, key experiments should be performed in both lines.

The opposite effects of hnRNPK ablation on TFAP2C expression between LN-229 C3 and U251-Cas9 cells likely reflect intrinsic differences between the two cell lines. However, the viability interaction between hnRNPK and TFAP2C is conserved in both cell models (Fig. 1G-H, 2A-B, Supp. Fig. 3A-B), suggesting that shared molecular functions at the interface of this interaction exist across the lines. In fact, we believe that the opposite effect of hnRNPK ablation on TFAP2C expression in the two lines strengthens (rather than weakens) our model by highlighting how TFAP2C expression modulates cellular sensitivity to HNRNPK ablation, as detailed in our response to Reviewer #2 (Point 10 - Major comments).
Regarding the mechanistic studies presented in FIG. 4-7, our initial goal in using two cell lines was to validate the functional viability interaction between HNRNPK and TFAP2C, as identified in our screening (performed in LN-229 C3 cells). After confirming this interaction, we chose to focus only on LN-229 C3 (beginning with RNA-seq analysis, which then led to subsequent mechanistic studies), as this provided the necessary foundation to investigate prion propagation in HovL cells (derived from LN-229). As a U251 model propagating prions does not exist, we are technically limited in performing prion experiments only in HovL and we do not believe that conducting additional experiments in U251 cells would add substantial value to our work or further our investigation.
We hope this explanation clarifies our rationale and addresses the reviewer's concerns.

2) Although a lot of data are presented, it is not clear how deletion of the TFAP2C reverses the toxicity caused by deletion of hnRNPK. Specifically, the first half of the paper seems to suggest an opposite mechanism than the second half of the paper. In Figure 2-4, the authors suggest a model that TFAP2C deletion has the opposite effect of hnRNPK deletion, thus rescuing toxicity. However, in Figure 5-6, it is suggested TFAP2C overexpression has the opposite effect of hnRNPK deletion. This two opposite effect of TFAP2C make it difficult to understand the models that the authors are proposing. Please also see below comment 2 for Figure 5.

We respectfully disagree with the notion that the first and second halves of the manuscript propose contradictory mechanisms.
In Fig. 2–4, we describe the phenotypic rescue of cell viability upon TFAP2C deletion in hnRNPK-deficient cells. At this stage, we are not proposing a specific molecular mechanism but simply

observing a rescue of viability and highlighting underlying transcriptional differences. There is no implication of an opposite molecular mechanism involving the individual activities of hnRNPK and TFAP2C; rather, we focused on the broader effect of TFAP2C deletion on the viability of HNRNPK-lacking cells. In Fig. 5, we isolated a partial mechanism underlying this interaction. We state that: *"These data specify a role for TFAP2C in promoting mTORC1-mediated cell anabolism and suggest that its overexpression might hypersensitize cells to HNRNPK ablation by depleting the already limited ATP available, thus making its deletion advantageous"*. In the discussion, we now further reviewed our explanation: *"HNRNPK deletion might cause a metabolic impairment leading to a nutritional crisis and a catabolic shift, whereas TFAP2C activation could promote mTORC1 anabolic functions. Thus, Tfap2c removal may rewire the bioenergetic needs of cells by modulating the mTORC1 signaling and augmenting their resilience to metabolic stress like the one induced by HNRNPK ablation"*. Therefore, we propose that TFAP2C expression might be particularly detrimental in hnRNPK-deficient cells, as it could push the cell into an anabolic biosynthetic state, further depleting energy stores that the cell is attempting to conserve in response to hnRNPK depletion. Removal of TFAP2C alleviates this metabolic strain. In our view, there is no contradiction between our observations.
We hope this explanation clarifies our rationale and resolves any perceived inconsistency in our model. To further enhance the understanding of our interpretations, we have now also added (in substitution of Fig. 5E of the original manuscript) a graphical scheme (Fig. 5G of the revised manuscript) to visually explain and illustrate our model (attached below for your convenience).

G

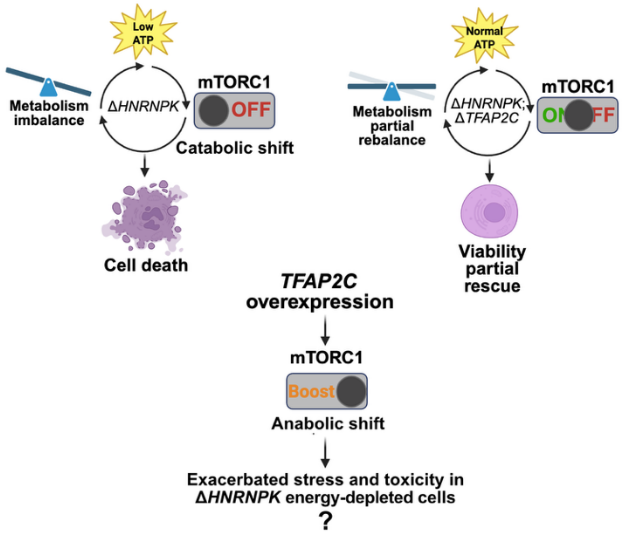

3) Similar to the point above, the first half of the paper focuses on hnRNPK deletion-induced toxicity (Fig. 1-5), while the second half of the paper focuses on hnRNPK deletion-induced PrPSC level (Fig. 6-7). The mechanistic link between these two downstream effects of hnRNPK deletion is not clear and thus, it is difficult to understand the reason that hnRNPK deletion-induced toxicity can be rescued by TFAP2C deletion, while hnRNPK deletion-induced PrPSC level increase can be rescued by TFAP2C overexpression.

Our study is not aimed at comparing viability and prion propagation as interconnected phenotypes but rather at identifying molecular processes regulated by the HNRNPK-TFAP2C interaction. Our study identifies mTORC1 activity as a molecular process at the interface of the HNRNPK-TFAP2C. HNRNPK knockout (or knockdown, which does not affect viability, and therefore is used in the prion section of the manuscript) tones mTORC1 activity down, while TFAP2C overexpression enhances it. This finding suggested an explanation for the viability interaction we observed (see reply to reviewer #3 - Point 2 -Major comments) and it provided a partial mechanism (mTORC1 activity) to explain the effect of HNRNPK knockdown and TFAP2C overexpression on prions.
We hope this clarification addresses the reviewer's concern.

Abstract.

1) Please rephrase and clarify "We linked HNRNPK and TFAP2C interaction to mTOR signaling..." by distinguishing functional, genetic, and direct (molecule-to-molecule) interactions.

1) We have now clarified it in the text of the revised manuscript as follows:
"We linked HNRNPK and TFAP2C functional and genetic interaction to mTOR signaling, observing that HNRNPK ablation inhibited mTORC1 activity through downregulation of mTOR and Rptor, while TFAP2C overexpression enhanced mTORC1 downstream functions."

2) A sentence reads, "...HNRNPK ablation inhibited mTORC1 activity through downregulation of mTOR and Rptor," although the downregulation of Rptor is observed only at the RNA level. The change in Rptor protein expression level is not reported in the manuscript. Please consider adding an experiment to address this or rephrase the sentence.

2) We have now added the experiment in Supp. Fig. 9A of the revised manuscript. The blot shows that hnRNP K depletion reduces both mTOR and Rptor protein levels. *"hnRNP K depletion inhibited mTORC1 activity through downregulation of mTOR and Rptor".*

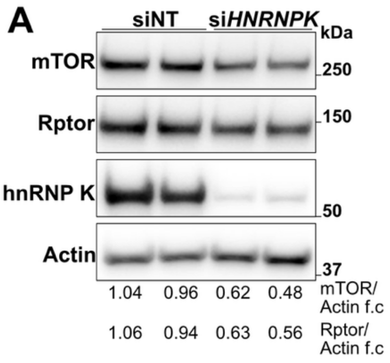

Figure 2.

1. H and I. Co-IP experiments were done using anti-TFAP2C antibody to the bead. Although the TFAP2C bands show robust signals on the blots, indicating successful enrichment of the protein, hnRNP K bands are very faint. Has the experiment been done by conjugating the hnRNP K antibody to the beads instead? Was the input lysate enriched in the nuclear fraction? Did the lysis buffer include nuclease (if so, please indicate in the figure legend and the methods section)? Addressing these would make the argument, "We also observed specific co-immunoprecipitation of hnRNP K and Tfap2c in LN-229 C3 and U251-Cas9 cells (Fig. 2H-I, Supp. Fig. 3L), suggesting that the two proteins form a complex inside the nucleus" stronger, providing information on potential direct binding.

1. We refer the reviewer to our response to reviewers #1 and #2 regarding the weak interaction, the nuclease treatment, and the HNRNPK IP (reviewer #1 Point 1 and reviewer #2 Point 11 - Major comments). As for the co-IP input, it was not enriched in the nuclear fraction, but as shown in Supp. Fig. 4A-B hnRNPK and Tfap2c are exclusively nuclear.

Figure 3.

1. C and D. Please add a sentence in the figure legend explaining which means the multiple comparisons were made between (DMSO vs each drug concentration?). Graphing individual data points instead of bars would also be helpful and more informative. Please discuss the lack of dose dependency.

1. We have now added information about the comparison in the figure legend (*"Multiple comparison was made between Z-VAD-FMK and DMSO treatments in ΔHNRNPK cells."*), modified the graph to show the individual data points (attached below for your convenience), and expanded the discussion as detailed for reviewer #2 (Point 14 – Major comments). (For completeness, we have also modified Supp. FIG. 5F to show individual data points, and we have combined the graphs (the DMSO control was shared across treatments)).

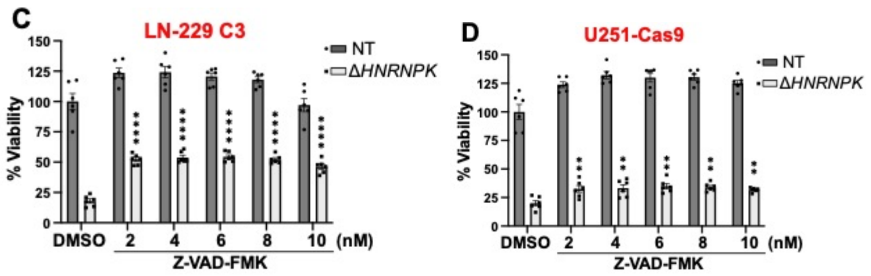

Supplemental Figure 4. (Now shifted in Supplemental Figure 5)

1. A. Although the trend can be observed, the deletion of hnRNP K does not significantly reduce the GPX4 protein level in LN-229 C3. Therefore, the following statement requires more data points and additional statistical analysis to be accurate: "In LN-229 C3 and U251-Cas9 cells, the deletion of HNRNPK reduced the protein level of GPX4, whereas TFAP2C deletion increased it (Supp. Fig. 4A-B)."

2. A and B. The results are confusing, considering the previous report cited (ref 49) shows an increase in GPX4 with TFAP2C. It may be possible that the deletion of TFAP2C upregulates the expression of proteins with similar functions (e.g., Sp1). If this is the case, the changes in GPX4 expression observed here are a consequence of TFAP2C deletion and may not "suggest a role for HNRNPK and TFAP2C in balancing the protein levels of GPX4."

1. We agree with the reviewer that in LN-229 C3 cells the reduction of GPX4 protein levels upon HNRNPK deletion did not reach statistical significance in our initial Western blot analysis. To address this concern, we performed six additional independent experiments and repeated the statistical analysis. Although the trend toward reduced GPX4 protein levels remained consistent, statistical significance was still not achieved ($p > 0.05$). Importantly, this trend is supported by our RNA-seq dataset (Supplementary Table 5), which shows decreased GPX4 expression upon HNRNPK deletion. We have now revised the text to more accurately reflect the experimental observations and to avoid overstating the effect in LN-229 C3 cells as follows:
"In LN-229 C3 and U251-Cas9 cells, deletion of HNRNPK was associated with reduced glutathione peroxidase 4 (GPX4) protein abundance (although not statistically significant in LN-229 C3; $p \approx 0.08$), whereas deletion of TFAP2C increased it (Supp. Fig. 5A-B)."
The six new experimental replicas have been added to the uncropped western blot section.

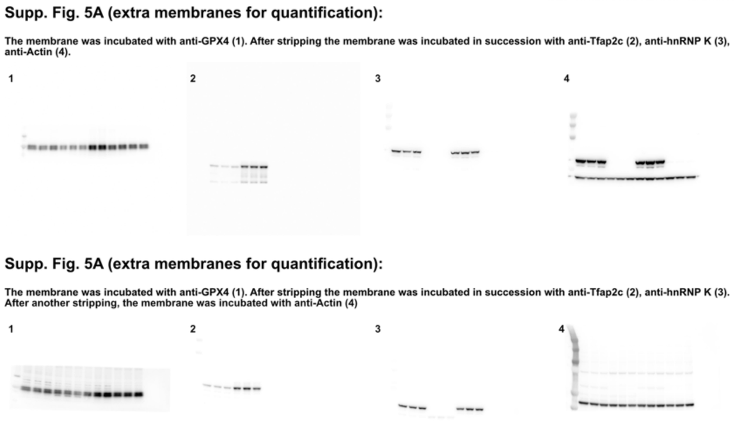

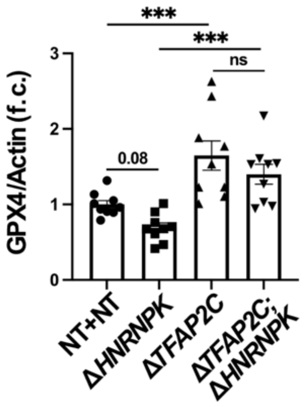

2. Concerning the potential role of TFAP2C deletion in upregulating proteins with similar functions, we recognize the reviewer's perspective. However, our primary focus is on the observed trends rather than a definitive mechanistic conclusion. We clarified our wording to acknowledge this

possibility while maintaining the relevance of our findings within the broader context of hnRNPK and TFAP2C interactions.
"This last result was interesting as a previous study reported that Tfap2c enhances GPX4 expression (51). Thus, the observed increase upon TFAP2C deletion suggests additional layers of regulation, potentially involving compensatory mechanisms."

Supplemental Figure 5. (Now shifted in Supplemental Figure 6)

1. B. To obtain statistical significance and strengthen the conclusion, more repeated Western blot experiments can be done to quantify the pAMPK/AMPK ratio.

We included three more experiments as detailed in our response to reviewer #1 (Point 2 – Major comments) and reviewer #2 (Point 14 – Major comments).

Figure 5.

1. B. I believe statistical analysis with two replicates or less is not recommended. Although the assay is robust, and the blot is convincing, please consider adding more replicates if the blot is to be quantified and statistically analyzed.

2. "Interestingly, RNA and protein levels of mTOR were downregulated in LN-229 C3ΔHNRNPK cells but were partially rebalanced by the ΔTFAP2C;ΔHNRNPK double deletion (Fig. 4C, Supp. Fig. E)." The statement is based on a slight difference at the protein level between the single deletion and the double deletion, as well as the observation from the bulk RNA-seq data. mTOR (and Rptor) mRNA level can be assessed by RT-qPCR to validate and further support the existing data. It is also curious why deletion of TFAP2C alone, also induced decrease in mTOR, but double deletion rescued mTOR level slightly compared to deletion of HNRNPK alone.

3. C. The main text refers to the changes in the level of phosphorylated E4BP1, stating, "Deletion of HNRNPK diminished the highly phosphorylated forms of 4EBP1, which instead were preserved in both LN-229 C3ΔTFAP2C and LN-229 C3ΔTFAP2C;ΔHNRNPK cells (Fig. 5C)." However, the quantification was done on the total E4BP1, which may be because separating pE4BP1 and E4BP1 bands on a blot is challenging. Please consider using phospho-E4BP1 specific antibody or rephrase the sentence mentioned above. The current data suggest the single- and double-deletion of hnRNP K/TFAP2C affect the overall stability of E4BP1, which may be a correlation and not due to the mTOR activity as claimed in "We conclude that HNRNPK and TFAP2C play an essential role in co-regulating cell metabolism homeostasis by influencing mTOR and AMPK activity and expression." How does the cap-dependent translation (or total protein level) change in TFAP2C deleted and overexpressing cells?

1. We added two additional experiments as detailed in our response to reviewer #1 (Point 3 – Major comment).

2. Deletion of TFAP2C does not decrease mTOR levels as shown from the quantification in Fig. 5D. To further support our results, we have now included RT-qPCR in FIG. 5C as suggested by the reviewer. Data are also attached here for your convenience.

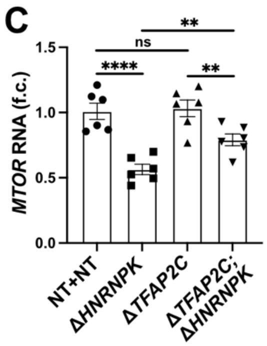

3. Regarding the assessment of phosphorylated 4EBP1, we think we achieved a clear separation of the differently phosphorylated forms of 4EBP1 in our blots, and we have now added the quantification for High p4EBP1/4EBP1 in Fig. 5E (see also our response to reviewer #2 Point 16 – Major comments). The quantification of total 4EBP1 represents an additional dataset, and we do not claim that 4EBP1 stability is affected by HNRNPK and TFAP2C directly through mTOR, which could be, in fact, correlative. We claim that HNRNPK and TFAP2C modulate mTORC1 and AMPK metabolic signaling as shown by the changed phosphorylation of 4EBP1, S6, AMPK, and ULK1 (Fig. 5C-E, Supp. FIG. 6B, D) and by the regulation of autophagy (Fig. 5B, Supp. Fig. 6C); we did not directly check cap-dependent translation.
We have now rephrased our text to ensure clarity as follows:
"We conclude that HNRNPK and TFAP2C play a role in co-regulating mTORC1 and AMPK expression, signaling, and activity."

Figure 6.

1. A. Did the sihnRNP K increase the TFAP2C level?

2. A and C. Are the total PrP levels lower in TFAP2C overexpressing cells compared to mCherry cells when they are infected?

3. D. Do the TFAP2C protein levels differ between 2-day+72-h and 7-day+96-h?

1. Yes, it does. We have now provided the quantification in Fig. 6A, C, and Supp. Fig. 8A (also attached below for your convenience).

2. We have now provided the quantification in Fig. 6A and Supp. Fig. 8A. The total PrP does not change in TFAP2C overexpressing cells. Total PrP consists of both PK-resistant PrP (PrP^{Sc}) and PK-sensitive PrP (PrP^{C} plus potential other intermediate species), with PrP^{Sc} typically present at much lower levels. In our model, PrP^{C} is exogenously expressed at high levels via a vector and remains constant across conditions (Fig. 6C and Supp. Fig. 8C). As a result, any changes in PrP^{Sc} may not necessarily reflect on total PrP levels.

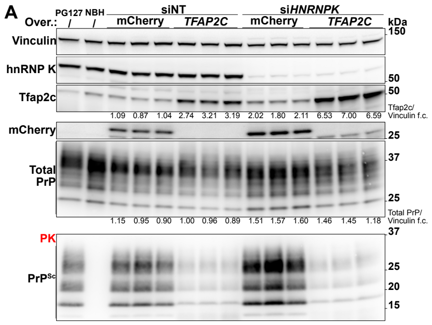

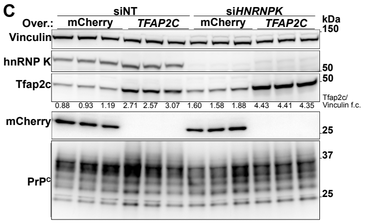

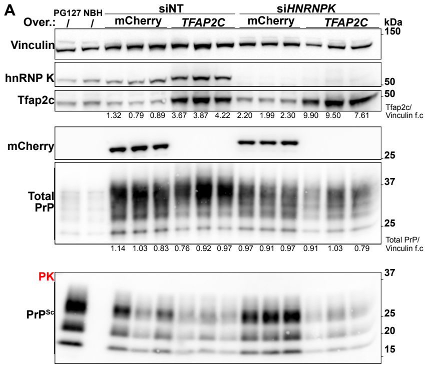

3. No, there is no statistically significant change. We have now added a representative western blot and the quantification of 3 independent replicates in Supp. Fig. 8D. The other two western blots are only shown in the uncropped western blots section. This dataset is also attached here for your convenience.

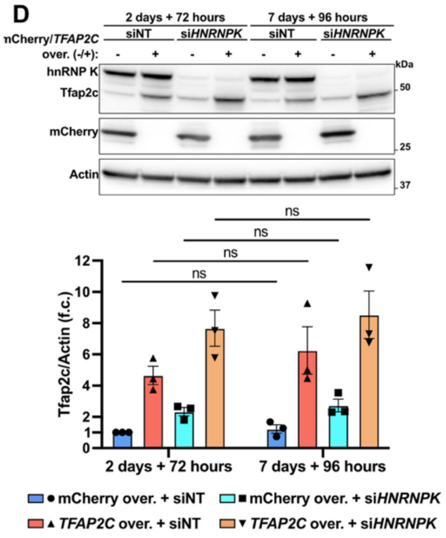

Supp. Fig. 8D:

The membrane was cut below 37 kDa (1 and 2). Membrane 1 (35-250 kDa) was incubated with anti-Tfap2c and anti-hnRNP K (1). Membrane 2 (10-35 kDa) was incubated anti-mCherry (2). Membrane 1 was following stripped and incubated with anti-Actin (3). Shown are three experimental repeats. Only the first row of images was used as the representative figure in the main text.

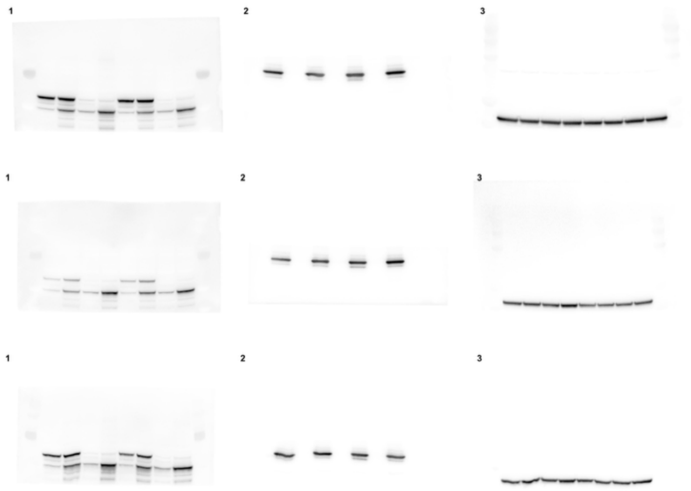

Figure 7.

1. I agree with the latter half of the statement: "These findings suggest that HNRNPK influences prion propagation at least in part through mTORC1 signaling, although additional mechanisms may be involved." The first half requires careful rephrasing since (A) Independent of the background siRNA treatment, TFAP2C overexpression by itself can modulate PrPSC level as seen in Fig 6A and B, (B) Although the increase in TFAP2C level is observed with the hnRNP K deletion (Fig 1; LN-229 C3), sihnRNP K treatment may or may not influence the TFAP2C level (Fig 6; quantified data not provided), and (C) In the sihnRNP K-treated cells, E4BP1 level is increased compared to the siNT-treated cells, which was not observed hnRNP K-deleted cells. Discussions and additional experiments (e.g., mTOR knockdown) addressing these points would be helpful.

A, B) We respectfully disagree with the possibility that HNRNPK downregulation may increase prion propagation via TFAP2C upregulation. As shown in Fig. 6A-B, D and in Supp. Fig. 8A, TFAP2C overexpression reduces, rather than increases, prion levels. Therefore, it would be inconsistent to suggest that HNNRPK siRNA promotes prion propagation through TFAP2C upregulation (quantification is now provided, see reviewer #3 – Figure 6 – Point 1). C) Concerning 4EBP1 levels, we have quantified the total 4EBP1 (also attached below) and expanded the discussion on potential discrepancies between HNRNPK knockout and knockdown, as the former affects cell viability, while the latter does not. However, as explained also in the previous reply to reviewer #3 – Figure 5 – Point 3, our focus is on the highly phosphorylated band of 4EBP1 (High p4EBP1), which is the direct target of mTORC1 activity. In both the hnRNPK knockout LN-229 C3 (Fig. 5E) and knockdown HovL models (Fig. 7B), phosphorylation of 4EBP1, along with phosphorylation of S6, is clearly reduced (we have now included quantification for Fig. 7B), reinforcing our conclusion that mTORC1 activity is affected by hnRNPK depletion. As the reviewer noted, we do not claim that mTORC1 is the sole mediator of hnRNPK's effect on prion regulation. However, we think that our interpretation of a potential and partial role of mTORC1 inhibition in the effect of HNRNPK downregulation on prion propagation is in line with the data presented in Fig. 6-7 and Supp. Fig. 8-9. For further clarification, we expanded the text according to the new experiments and analysis, and we added mTOR and Raptor siRNA knockdown (Supp. Fig.9C) to further support our conclusions (also attached below for your convenience).

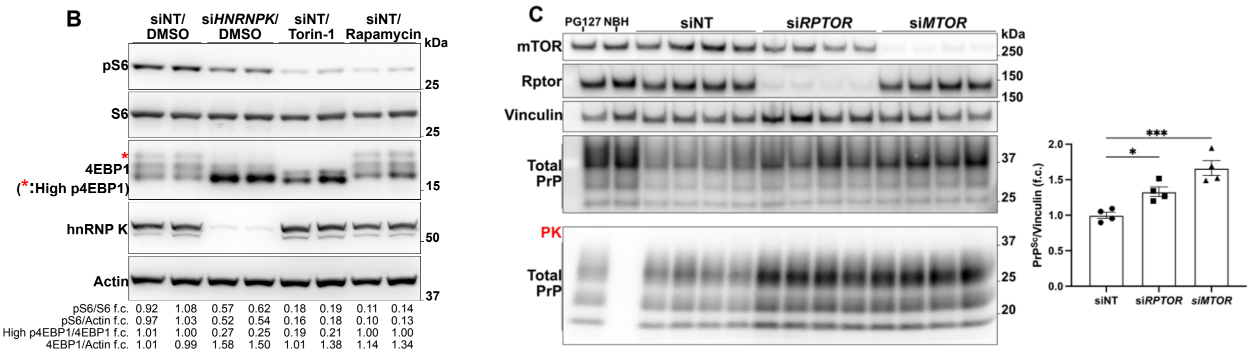

Minor comments:

1. Please clarify "independent cultures." Does this mean technical replicates on the same cell culture plate but different wells or replicated experiments on different days?

We have now clarified in each figure legend. “Individually treated wells” means different parental cultures grown and treated separately on the same day. n represents independent experiments on different days.

2. Fig 2G. Please explain how the sigmoidal curves were fitted to the data points under the materials and methods section.
2. Fig 3E and F. Please refer to the comment on Fig 2G above.

We have now added the explanation in Materials and Methods as follows:
“Curve Fitting
For sigmoidal curve fitting, we used GraphPad Prism (version X, GraphPad Software). Data in Figure 2G were fitted using nonlinear regression with a least squares regression model. For Figures 3E and 3F, data fitting was performed using an asymmetric sigmoidal model with five parameters (5PL) and log-transformed X-values (log[concentration]).”

3. Fig S3 F/H. Quantification of gel bands would be helpful when comparing protein expression changes after different treatments, as band intensities look different across.

We have now added the quantifications in Supp. FIG. 3D-H (attached below for your convenience). They confirm that there are no significant differences in the means of the normalized values.

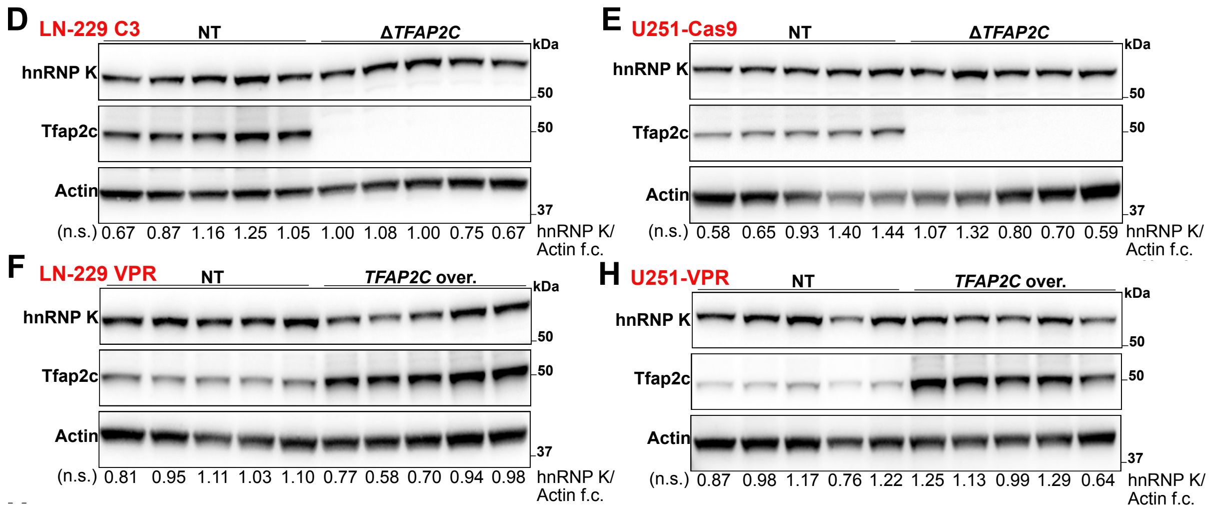

4. Supp Fig 5C and F. These panels can be combined with the corresponding panels in main Figure 5 if space allows so that the readers do not have to flip pages between the main text and Supplemental material.

We have now combined the panels. Previous Supp. FIG. 5C and F are now shown in FIG. 6C and E, respectively.

Reviewer #3 (Significance (Required)):

This is an interesting paper potentially providing new mechanical insight of hnRNPK function and its interaction with TFAP2C. It is also important to understand how hnRNPK deletion induces prion propagation and develop methods to mitigate its spread. However, inconsistencies in TFAP2C expression across cell lines and conflicting mechanistic interpretations complicate conclusions. I have expertise in RNA-binding protein, cell biology, and prion disease.
